# Exploring the Pharmacological Mechanism of Liuwei Dihuang Decoction for Diabetic Retinopathy: A Systematic Biological Strategy-Based Research

**DOI:** 10.1155/2021/5544518

**Published:** 2021-08-02

**Authors:** Mengxia Yuan, Qi He, Zhiyong Long, Xiaofei Zhu, Wang Xiang, Yonghe Wu, Shibin Lin

**Affiliations:** ^1^Shantou University Medical College, Shantou University, Shantou, Guangdong, China; ^2^Joint Shantou International Eye Center of Shantou University and the Chinese University of Hong Kong, Shantou City, Guangdong Province, China; ^3^Hunan University of Chinese Medicine Affiliated People's Hospital of Ningxiang City, Ningxiang City, Hunan Province, China

## Abstract

**Objective:**

To explore the pharmacological mechanism of Liuwei Dihuang decoction (LDD) for diabetic retinopathy (DR).

**Methods:**

The potential targets of LDD were predicted by PharmMapper. GeneCards and other databases were used to collect DR genes. Cytoscape was used to construct and analyze network DR and LDD's network, and DAVID was used for Gene Ontology (GO) and pathway enrichment analysis. Finally, animal experiments were carried out to verify the results of systematic pharmacology.

**Results:**

Five networks were constructed and analyzed: (1) diabetic retinopathy genes' PPI network; (2) compound-compound target network of LDD; (3) LDD-DR PPI network; (4) compound-known target network of LDD; (5) LDD known target-DR PPI network. Several DR and treatment-related targets, clusters, signaling pathways, and biological processes were found. Animal experiments found that LDD can improve the histopathological changes of the retina. LDD can also increase erythrocyte filtration rate and decrease the platelet adhesion rate (*P* < 0.05) and decrease MDA and TXB2 (*P* < 0.05). Compared with the model group, the retinal VEGF and HIF-1*α* expression in the LDD group decreased significantly (*P* < 0.05).

**Conclusion:**

The therapeutic effect of LDD on DR may be achieved by interfering with the biological processes (such as response to insulin, glucose homeostasis, and regulation of angiogenesis) and signaling pathways (such as insulin, VEGF, HIF-1, and ErbB signaling pathway) related to the development of DR that was found in this research.

## 1. Introduction

Diabetic retinopathy (DR) is a microvascular complication of diabetes [1], which is the most common cause of blindness in working-age adults [2, 3]. Its pathogenesis is hyperglycemia-induced endothelial thinning and impaired internal barrier function, leading to retinopathy and dysfunction [1]. The current global DR prevalence rate is estimated at 126 million of the 382 million diabetic patients [3]. Meanwhile, people with DR also have a greater risk of developing other cardiovascular complications, such as diabetic cardiomyopathy and diabetic nephropathy [4, 5]. Therefore, the prevention of DR and reduction of DR-caused visual impairment are currently hot topics in medical research.

Currently, the treatment strategies of DR mainly include the following: control of systemic risk factors (glucose, blood pressure, and serum lipids), ophthalmic administration, and laser surgery. For nonproliferative diabetic retinopathy (NPDR), the medical treatment can delay its progression, ameliorate visual performance, and reduce the adverse events of laser treatment [6]. For severe NPDR and proliferative retinopathy (PDR), the panretinal photocoagulation can prevent visual impairment but cannot improve vision [7]. At present, complementary and alternative medicine (CAM) is gradually being applied to the prevention and treatment of DR. As an important part of CAM, traditional Chinese medicine (TCM) is receiving more and more attention in the prevention and treatment of DR [8, 9]. Compared with conventional treatment regimens, the long-term application of TCM has less toxicity and side effects [10–12]. Therefore, TCM is becoming more and more popular among patients with DR [13].

**Figure 1:**
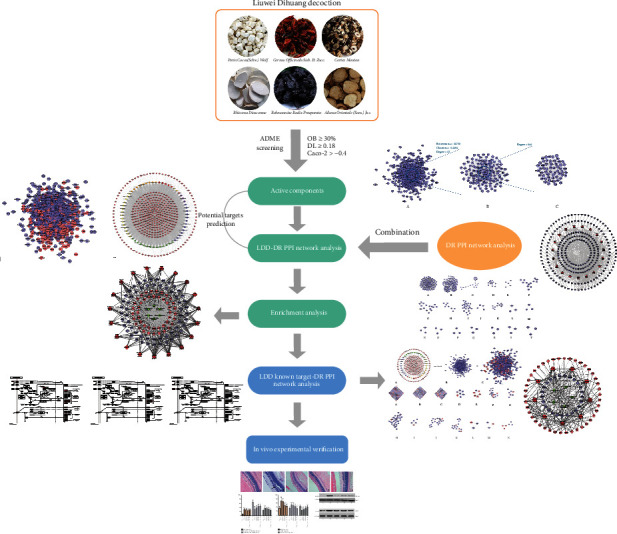
Research process.

Liuwei Dihuang decoction (LDD) is a classical TCM herbal formula consisting of six herbs: Rehmanniae Radix Praeparata (Shu Di Huang), *Cornus officinalis* Sieb. et Zucc. (Shan Zhu Yu), Rhizoma Dioscoreae (Shan Yao), *Alisma orientale* (Sam.) Juz. (Ze Xie), Cortex Moutan (Mu Dan Pi), and *Poria cocos* (Schw.) Wolf. (Fu Ling), and the ratio of which is 8 : 4 : 4 : 3 : 3 : 3 [14]. In the past few centuries, LDD has been widely used in the treatment of diabetes (“Xiao Ke Zheng” in TCM) and diabetic complications [14]. Meanwhile, latest systematic review and mata-analysis showed that LDD combined with conventional treatment regimens can improve the treatment of type 2 diabetes compared with the control group [15]. Another clinical study showed that LDD combined with Ginkgo Leaf Tablets can reduce the incidence of DR [14]. Therefore, LDD may have a therapeutic effect on DR. However, the mechanism of LDD in the treatment of DR is not clear. With the rapid development of bioinformatics, systematic biological approach has become a way to study multicomponent drugs for the treatment of multitarget diseases, indicating that the mechanism of TCM herbal formula can be studied by means of “multidrug-multicomponent-multitarget-multipathway” [16–19]. Hence, we utilized the systematic biological approach to uncover the mechanism of LDD on DR in this study. The research processes are shown in Figure 1.

## 2. Materials and Methods

### 2.1. LDD's Compound Prediction

The TCMSP [20] (http://lsp.nwsuaf.edu.cn) was employed to collect the compounds of LDD. The database contains all the drugs included in the 2010 edition of the Chinese Pharmacopoeia, as well as the pharmacokinetic properties, drug similarity, intestinal epithelial permeability, blood-brain barrier, and water solubility of natural compounds involving oral bioavailability (OB). OB, Caco-2 parameters, drug-likeness (DL), and half-life (HL) are important indicators for evaluating whether a compound can be developed into a drug [21]. In this study, compounds with OB ≥ 30%, Caco-2 > −0.4, and DL ≥ 0.18 were selected as candidate components for subsequent target prediction [22–24]. After screening, the following compounds were obtained: (2R)-2-[(5R,10S,13R,14R,16R,17R)-16-hydroxy-3-keto-4,4,10,13,14-pentamethyl-1,2,5,6,12,15,16,17-octahyddrocyclopenta[a]phenanthren-17-yl]-5-isopropyl-hex-5-enoic acid(MOL000285), (−)-taxifolin, (+)-catechin, (2R)-2-[(3S,5R,10S,13R,14R,16R,17R)-3,16-dihydroxy-4,4,10,13,14-pentamethyl-2,3,5,6,12,15,16,17-octahydro-1H-cyclopenta[a]phenanthren-17-yl]-5-isopropyl-hex-5-enoic acid (MOL000280), (2R)-2-[(3S,5R,10S,13R,14R,16R,17R)-3,16-dihydroxy-4,4,10,13,14-pentamethyl-2,3,5,6,12,15,16,17-octahydro-1H-cyclopenta[a]phenanthren-17-yl]-6-methylhept-5-enoic acid (MOL000273), 2,6,10,14,18-pentamethylicosa-2,6,10,14,18-pentaene (MOL005481), 3,4-dehydrolycopen-16-al, 3beta-hydroxy-24-methylene-8-lanostene-21-oic acid (MOL000287), 4-O-methylpaeoniflorin, 7,9(11)-dehydropachymic acid, AIDS180907, alisol B, alisol B23 acetate, alisol C, beta-sitosterol, campesterol, cerevisterol, CLR, cornudentanone, dehydroeburicoic acid, denudatin B, diop, dioscoreside C, diosgenin, doradexanthin, ergosta-7,22E-dien-3beta-ol, ergosterol peroxide, ethyl linolenate, ethyl oleate (NF), hancinol, hancinone C, hederagenin, hydroxygenkwanin, isofucosterol, kadsurenone, kaempferol, lanosta-8,24-dien-3-ol,3-acetate, mairin, malkangunin, mandenol, methylcimicifugoside, mudanpioside H, pachymic acid, paeonidanin, paeoniflorin, piperlonguminine, poricoic acid A, poricoic acid B, poricoic acid C, poriferast-5-en-3beta-ol, quercetin, sitosterol, stigmasterol, telocinobufagin, tetrahydroalstonine, trametenolic acid, and ZINC02816192.

Since the application of biological models to predict LDD compounds has limitations [25], in order to avoid missing active compounds during the prescreening process, we searched a large number of references and selected oral absorbable compounds with pharmacological activity. Combined with relevant references [26–29], the following compounds are included: stachyose, rehmannioside D, catalpol, acteoside, and rehmannioside A.

### 2.2. LDD's Potential and Known Targets and Diabetic Retinopathy Gene Collection

The structure of each compound was collected from PubChem (https://pubchem.ncbi.nlm.nih.gov) with “sdf” format files. Then, those files were input into PharmMapper (http://lilab.ecust.edu.cn/pharmmapper/) to predict the targets of each compound [30]. The known targets were collected from the TCMSP [21]. The DR-related genes were collected from the OMIM (http://omim.org/) database and GeneCards (http://www.genecards.org) [22–24, 31]. The OMIM database is the database that catalogues all known diseases with a genetic component [31]. The genes with relevance score ≥5 were selected for sequence research. The UniProt database (https://www.Uniprot.org/) is used to query the official gene symbol of target proteins and DR genes, and the biological species is set to *Homo sapiens* (human). The official gene symbol and other information of the potential targets, known targets, and DR genes are shown in Tables S1–S3.

### 2.3. Network Construction and Analysis Methods

The protein-protein interaction (PPI) data of LDD targets and DR genes were collected from the STRING database (http://string-db.org/) and the IntAct database (http://www.ebi.ac.uk/intact/) [22–24, 32, 33]. The node interaction type is default. The Cytoscape 3.7.0 software (http://cytoscape.org/) was utilized to construct and analyze the networks [34]. The closely connected part of the target in the PPI network is considered to be the main part of the biological function, namely, clusters [34]. The clusters of the PPI network were detected by MCODE (a plug-in of Cytoscape). The DAVID database (https://david-d.ncifcrf.gov, ver. 6.8) was utilized for pathway and Gene Ontology (GO) enrichment analysis [35].

### 2.4. Experimental Materials

#### 2.4.1. Experimental Anima

50 male New Zealand rabbits, weighing 1.7–2.0 kg, were provided by the Laboratory Animal Institute of Shantou University Medical College, license number: SYXK (Guang) 2014-0019 and raised in the Laboratory Animal Center (SPF level) of Shantou University Medical College. Animal experiments have been approved by the Animal Ethics Committee of Shantou University Medical College and performed in accordance with the guidelines for the care and use of experimental animals.

#### 2.4.2. Experimental Drugs

LDD is composed of Rehmanniae Radix Praeparata (Shu Di Huang), *Cornus officinalis* Sieb. et Zucc. (Shan Zhu Yu), Rhizoma Dioscoreae (Shan Yao), *Alisma orientale* (Sam.) Juz. (Ze Xie), Cortex Moutan (Mu Dan Pi), and *Poria cocos* (Schw.) Wolf. (Fu Ling), and the ratio of which is 8 : 4 : 4 : 3 : 3 : 3 [14]. The herbal medicines were purchased from the pharmaceutical factory of Beijing Tongrentang Technology Development Co., Ltd. (National Pharmaceutical Standard: Z19993068; lot number: K161101Z00). The dose for clinical patients is 18 g/d. The dose converted to rabbits was 0.84 g/kg, and twice the dose of LDD (1.68 g/kg) was used in the LDD high-dose group.

#### 2.4.3. Reagents and Instruments

Malondialdehyde (MDA), thromboxane B2 (TXB2), and 6-keto-prostaglandin F1*α* (6-keto-PGF1*α*) ELISA kits were purchased from the US R&D company. Alloxan was purchased from Sigma Corporation. MK3 type microplate reader was purchased from Thermo Company of America. The 5417R low-temperature high-speed centrifuge was purchased from the German company Eppendorf. The inverted fluorescence microscope was purchased from Olympus Corporation of Japan. The manual rotary microtome was purchased from the German company Leica. Electrophoresis tank, electrophoresis instrument, and gel imaging analysis system were purchased from Bio-Rad Inc. Ultra-micro nucleic acid protein analyzer was purchased from BioDrop Inc. RIPA Lysis Solution (Cat. No. 89900) and BCA Kit (Cat. No. 23234) were purchased from Thermo. Horseradish peroxidase labeled goat anti-rabbit secondary antibody (Cat. No. SV0002) was purchased from Wuhan Boster Company. Anti-rabbit VEGF primary antibody (Cat. No. ab1316) and anti-rabbit HIF-1*α* primary antibody (Cat. No. ab179843) were purchased from Abcam Inc.

### 2.5. Experimental Methods

#### 2.5.1. Animal Modeling

Healthy New Zealand rabbits were divided into two groups: normal control group (8) and model group (42). The model group received intravenous injection of alloxan 200 mg/kg. The normal control group was given the same dose of 0.9% sodium chloride solution. After 48 hours, the fasting blood glucose value was measured. If the blood glucose value was above 11.1 mmol/L, the model was considered successful. Thereafter, alloxan was injected on the 20th, 40th, and 60th days of the experiment to maintain the hyperglycemic status of New Zealand rabbits. On the 61st day, the fasting blood glucose value was detected as the final blood glucose concentration, and 40 New Zealand rabbits with a blood glucose value >20 mg/mL were selected for the next grouping and administration.

#### 2.5.2. Animal Grouping and Administration

40 New Zealand rabbits were divided into 5 groups: normal control group (8 rabbits), model group (8 rabbits), positive control group (8 rabbits), LDD high-dose group (8 rabbits), and LDD low-dose group (8 rabbits). LDD high- and low-dose groups were given LDD 1.68 and 0.84 g of crude drug/kg, respectively, and the positive control group was given calcium dobesilate 5.8 mg/kg. The normal control group and the model group were given the same amount of distilled water once a day for 30 days.

#### 2.5.3. Blood Index Detection

One hour after the end of the last administration, the blood glucose value was measured, and then New Zealand rabbits were anesthetized with 3% pentobarbital sodium 3 ml/kg to collect blood samples. The right eye retina was stored in 4% paraformaldehyde solution for pathological detection, and the left eye retina was frozen in liquid nitrogen. The erythrocyte filtration rate, platelet adhesion rate, and globulin dissolution time of New Zealand rabbit plasma in each group were detected, respectively.

#### 2.5.4. Pathomorphological Observation

The retinal tissues fixed in 4% paraformaldehyde solution were taken, sliced after embedding in conventional paraffin, observed and filmed under the microscope after HE staining, and the pathological damage of New Zealand rabbit retinal tissues in each group was analyzed.

#### 2.5.5. Enzyme-Linked Immunosorbent Assay (ELISA)

Cryopreserved retinal tissue was homogenized on ice, centrifuged at 4°C, 12000 r/min for 15 min, and the supernatant was taken. The MDA, TXB2, and 6-keto-PGF1*α* of retinas were tested in strict accordance with the instructions of the ELISA kit content.

#### 2.5.6. Retina HIF-1*α* and VEGF Protein Expression Detection by Western Blot

The cryopreserved retinal tissue was added to RIPA lysate to prepare tissue protein extract, and the protein content was determined by the BCA method. The protein expression levels of HIF-1*α* and VEGF in the retina were detected according to the Western Blot experimental procedures, and the relative protein expression levels were calculated based on the ratio of the corresponding gray values of the target protein and the internal reference protein.

### 2.6. Statistical Analysis

SPSS19.0 software was used for data analysis. The difference between groups was tested by Dunnett's post-test. The measurement data were expressed by *x* ± *s*, with *α* = 0.05 as the test level.

## 3. Results

### 3.1. Diabetic Retinopathy Network Analysis

#### 3.1.1. Diabetic Retinopathy Network

**Figure 2:**
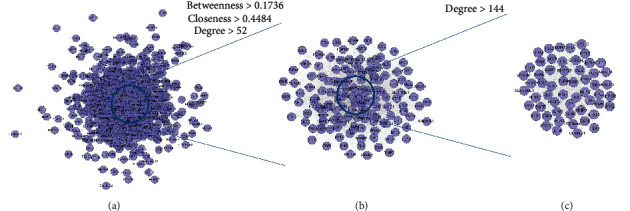
Diabetic retinopathy network.

This network consists of 738 genes nodes and 18996 edges (Figure 2). The targets are arranged in descending order of degree. The top 10 targets are as follows: ALB (381 edges), INS (339 edges), VEGFA (336 edges), IL6 (321 edges), GAPDH (320 edges), AKT1 (308 edges), TP53 (300 edges), TNF (298 edges), EGF (266 edges), JUN (258 edges), and so on (Figure 2(c)).

#### 3.1.2. Clusters of DR Network

**Figure 3:**
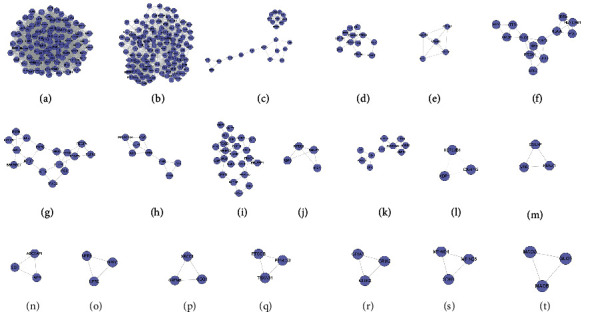
Cluster of the DR network (A, B, C,… represent cluster 1, 2, 3,…).

The DR PPI network was further analyzed by Cytoscape's plug-in MCODE, and 20 clusters were obtained (Table 1 and Figure 3). The genes in clusters were input into DAVID to perform GO enrichment analysis.

Cluster 19 did not return any human's biological processes. Cluster 10, 11, 12, 13, 14, 15, 16, 17, 18, and 20 did not return DR-related biological processes. The cluster 1 was taken as the example.

As mentioned above, cluster 1 gets GO:0000165, GO:0000187, GO:0014068, GO:0032735, GO:0042346, GO:0043066, GO:0048010, GO:0050796, GO:0051770, GO:0070371, GO:0070374, GO:0071347, GO:0071456, GO:1904707, etc. (Table S4).

#### 3.1.3. Pathway of DR Network

**Figure 4:**
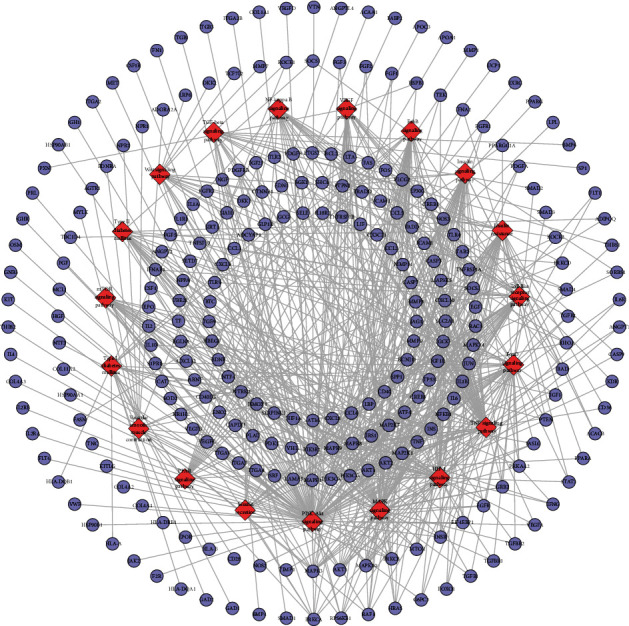
Pathway of the DR network (red diamonds represent pathways, and blue circles represent DR genes).

The genes in the DR PPI network were input into DAVID to perform signaling pathway analysis (Figure 4). The results showed that the DR-related signaling pathways may include the following: PI3K-Akt signaling pathway, MAPK signaling pathway, HIF-1 signaling pathway, TNF signaling pathway, FoxO signaling pathway, and so on (Table S5).

### 3.2. Compound-Compound Target Network Analysis

**Figure 5:**
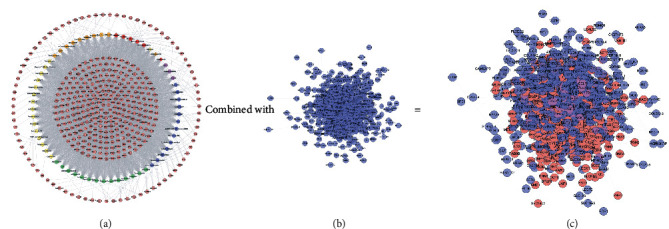
(a) Compound-compound target network of LDD (pink hexagons represent compound targets; red, orange, yellow, green, blue, and purple circles represent Rehmanniae Radix Praeparata, Cortex Moutan, *Poria cocos* (Schw.) Wolf., Rhizoma Dioscoreae, *Cornus officinalis* Sieb. et Zucc., and *Alisma orientale* (Sam.) Juz., resp.; red diamonds represent common compound of Rehmanniae Radix Praeparata, Rhizoma Dioscoreae, and *Cornus officinalis* Sieb. et Zucc.; orange diamonds represent common compound of *Cornus officinalis* Sieb. et Zucc. and Cortex Moutan; yellow diamonds represent common compound of *Cornus officinalis* Sieb. et Zucc. and Rehmanniae Radix Praeparata). (b) DR network. (c) LDD-DR network (pink, blue, and purple circles represent compound target, DR genes, and LDD-DR target, resp.).

This network is constructed by 429 nodes (62 compound nodes and 367 compound target nodes) and 8222 edges. The targets near the center are regulated by more compounds than peripheral targets. For example, AKR1B1, BACE1, CA2, CDK2, GSTP1, HSP90AA1, and LCK can be controlled by all of the compounds, while NOS3, PITPNA, PLA2G10, PLK1, and PNPO are regulated by only one compound (Figure 5(a)).

### 3.3. LDD-DR Network Analysis

#### 3.3.1. LDD-DR Network

This network contains 1015 nodes and 26867 edges. Two hundred and seventy-nine (279) nodes are compound targets, 79 nodes are LDD-DR targets, and 657 nodes are DR genes (Figure 5(c)).

#### 3.3.2. Clusters of LDD-DR Network

**Figure 6:**
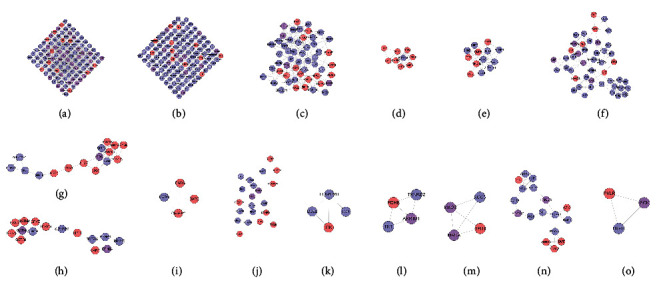
Cluster of the LDD-DR network (A, B, C, D,… represent cluster 1, 2, 3, 4,…). Pink, blue, and purple circles represent compound target, DR genes, and LDD-DR target, resp.

Twenty-one clusters were collected after analyzed by MCODE. The genes and targets in each cluster were input into DAVID for GO enrichment analysis (Table 2 and Figure 6).

Cluster 4, 9, and 11 did not return DR-related biological processes. The cluster 1 was taken as the example.

Cluster 1 gets a lot of biological processes, including GO:0000165, GO:0006006, GO:0006954, GO:0007179, GO:0007223, GO:0009749, GO:0010574, GO:0014066, GO:0032869, GO:0035924, GO:0043410, GO:0045429, GO:0045765, GO:0045766, GO:0045907, GO:0045909, GO:0070374, GO:0071347, GO:0071560, GO:0090370, GO:1904707, and GO:1990314 (Table S6).

#### 3.3.3. Pathway of LDD-DR Network

**Figure 7:**
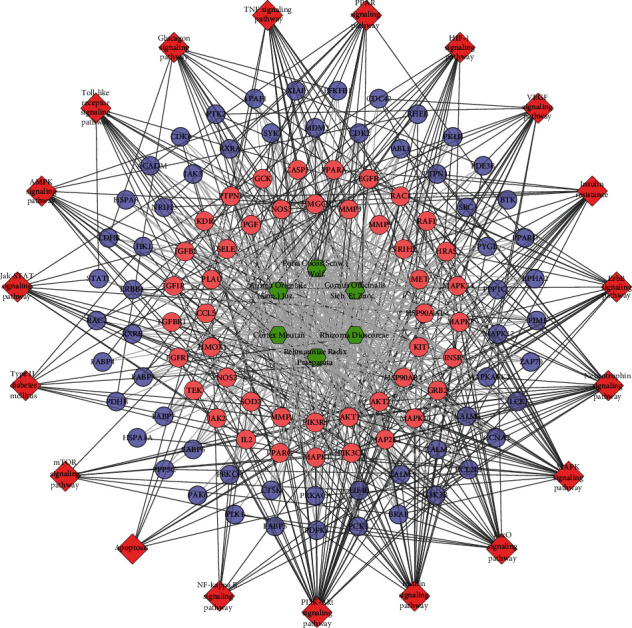
Pathway of the LDD-DR network. Blue circles represent compound targets. Pink circles represent LDD-DR targets. Red diamonds represent signaling pathways. Green hexagons represent herb. Black lines represent relationships of signaling pathways and compound targets. Light lines represent relationships of herb and compound target.

The genes in DR PPI network were input into DAVID to perform signaling pathway analysis (Figure 7). The results showed that the LDD may regulate the PI3K-Akt signaling pathway, insulin signaling pathway, FoxO signaling pathway, MAPK signaling pathway, etc. so as to treat DR (Table S7). Meanwhile, the compounds of Cortex Moutan totally regulate 92 targets (which is the most), while that of Rehmanniae Radix Praeparata regulate 88 targets. The compounds of *Cornus officinalis* Sieb. Et Zucc. and Rhizoma Dioscoreae regulate 82 targets. *Poria cocos* (Schw.) Wolf.'s compounds control 74 targets, and the compounds of *Alisma orientale* (Sam.) Juz. control 59 targets. This suggests that Cortex Moutan and Rehmanniae Radix Praeparata play a major role in LDD.

### 3.4. LDD Known Target-DR PPI Network Analysis

#### 3.4.1. LDD Known Target-DR PPI Network Construction

**Figure 8:**
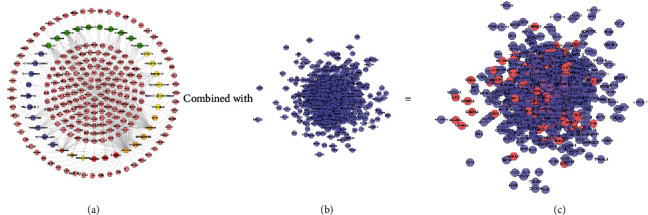
(a) Compound-known target network of LDD (pink hexagons represent compound targets; red, orange, yellow, green, and blue circles represent Rehmanniae Radix Praeparata, Cortex Moutan, *Poria cocos* (Schw.) Wolf., Rhizoma Dioscoreae, and *Cornus officinalis* Sieb. et Zucc., resp.; red diamonds represent common compound of Rehmanniae Radix Praeparata, Rhizoma Dioscoreae, and *Cornus officinalis* Sieb. et Zucc.; orange diamonds represent common compound of *Cornus officinalis* Sieb. et Zucc. and Cortex Moutan; yellow diamonds represent common compound of *Cornus officinalis* Sieb. et Zucc. and Rehmanniae Radix Praeparata). (b) DR network. (c) LDD known target-DR network (pink, blue, and purple circles represent known target, DR genes, and known-DR target, resp.).

The compound-known target network contains 242 nodes (37 compound nodes and 205 known compound target nodes) and 460 edges (Figure 8(a)). The LDD known target and DR genes were input into Cytoscape to construct LDD known target-DR network, which was composed of 856 nodes and 24369 edges (Figure 8(c)). This network is a confirmation of the previous prediction network.

#### 3.4.2. Cluster and Pathway of LDD Known Target-DR Network

**Figure 9:**
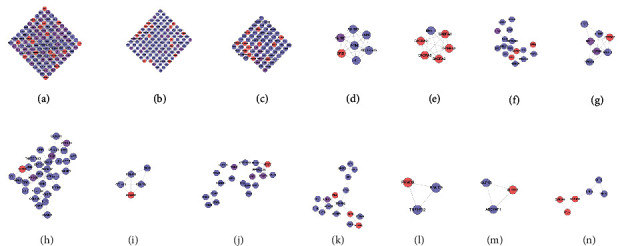
Cluster of the LDD known target-DR network (A, B, C, D … represent cluster 1, 2, 3, 4,…). Pink, blue, and purple circles represent known target, DR genes, and known-DR target, resp.

The LDD known target-DR network was further analyzed by Cytoscape's plug-in MCODE, and 17 clusters were obtained (Table 3 and Figure 9). Most of them are the same as that in the clusters of the LDD-DR network, verifying the LDD's effects indirectly.

Cluster 4 and 8 did not return DR-related biological processes. The cluster 1 was taken as the example.

Clusters 1 gets GO:0000165, GO:0000187, GO:0016525, GO:0017015, GO:0019229, GO:0030512, GO:0045766, GO:0045907, GO:0045909, GO:0046627, GO:0046628, GO:0048010, GO:0050727, GO:0050729, GO:0050796, GO:0070374, GO:0071363, etc. The details of each cluster and biological process are shown in Table S8.

**Figure 10:**
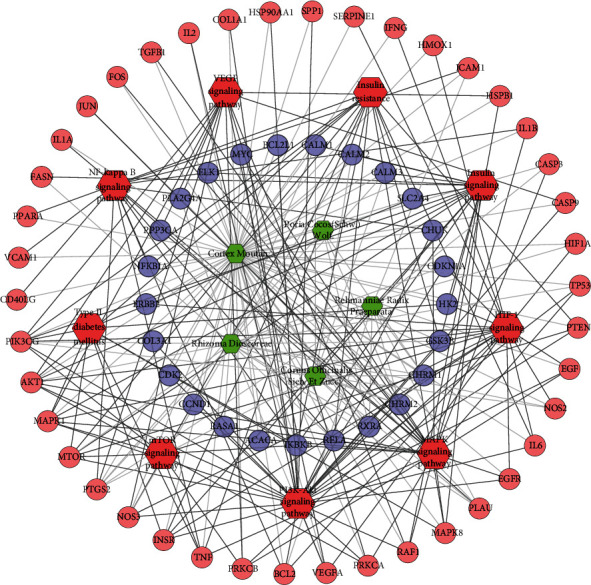
Pathway of the LDD known target-DR network. Blue circles represent known target. Pink circles represent known-DR targets. Red diamonds represent signaling pathway. Green hexagons represent herb. Black lines represent relationships of signaling pathways and known target. Light lines represent relationships of herb and compound target.

Meanwhile, nine DR-related pathways were obtained after the pathway enrichment analysis (Figure 10). Some of the signaling pathways are the same as pathways in Figures 3 and 6, suggesting that these pathways may be the crucial pathways in DR's development. The details of each signaling pathways are shown in Table S9.

**Figure 11:**
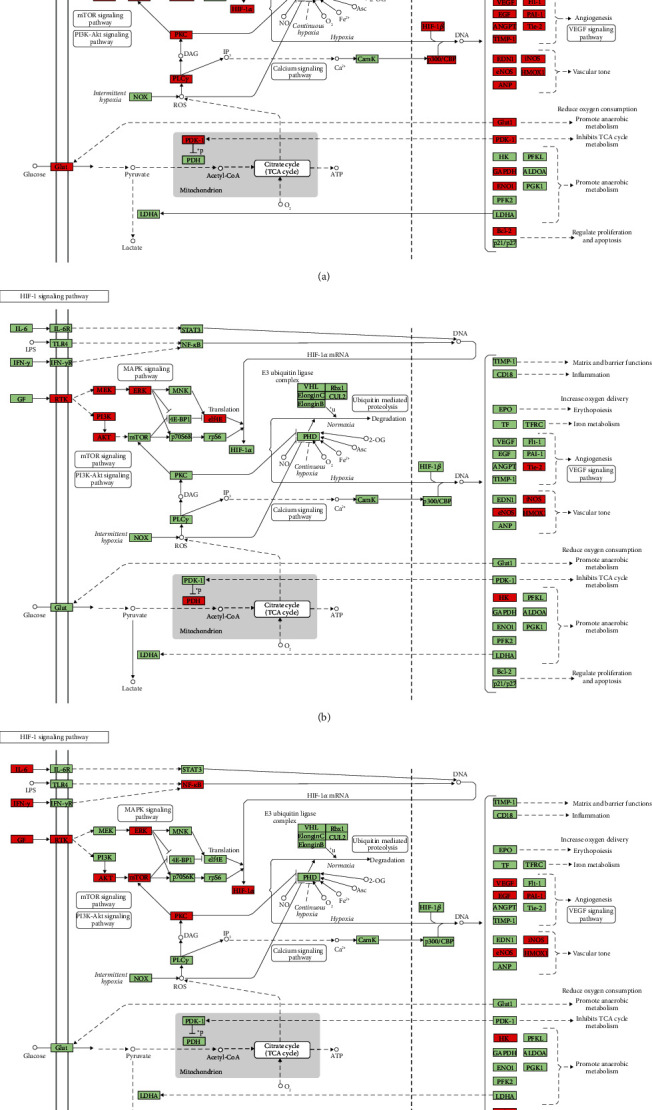
HIF signaling pathway adapted from KEGG (ID: hsa04066): (a) the DR genes were signed in red; (b) the predicted targets and DR genes were signed in red; (c) the known targets and genes were signed in red.

Since the HIF-1 signaling pathway is very important in DR's development and treatment (Figures 4, 7, and 10), the potential targets and DR genes are shown in Figure 10 (red squares) as an example. It can be found that some of the red squares in Figure 11(b) overlap with those in Figure 11(a), which further demonstrates the reliability of the predicted network.

### 3.5. Blood Test Results

**Figure 12:**
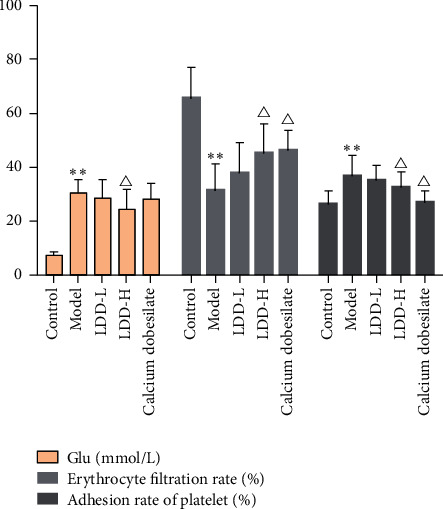
Blood test results (LDD-L: LDD low-dose group; LDD-H: LDD high-dose group; compared with the normal control group, ^*∗∗*^*P* < 0.01; compared with the model group, ^Δ^*P* < 0.05).

Compared with the normal control group, the blood glucose level of the model group was significantly increased (*P* < 0.01), the erythrocyte filtration rate was significantly decreased (*P* < 0.01), and the platelet adhesion rate was significantly increased (*P* < 0.05). Compared with the model group, the blood glucose value of the LDD high-dose group was significantly reduced (*P* < 0.05), the erythrocyte filtration rate of the positive control group and the LDD high-dose group was significantly increased (*P* < 0.05), and the platelet adhesion rate in the positive control group and LDD high-dose group was significantly reduced (*P* < 0.05) (Figure 12).

### 3.6. Pathomorphological Observation

**Figure 13:**
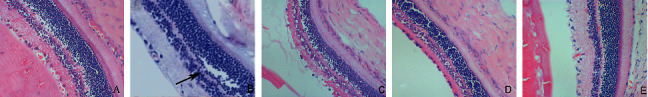
Pathomorphological observation (HE staining, X400). (a) Normal control group; (b) model group; (c) positive control group; (d) LDD high-dose group; (e) LDD low-dose group. Black arrow shows the lesion.

In the normal control group, the layers of the retina of the rabbit were clear, the cells were arranged neatly, and no obvious abnormalities were found. In the model group, the retinal network structure of the rabbit was loose, the arrangement of the inner and outer granular layers was disordered, the capillary wall was obviously twisted, interstitial edema, and the stenosis of the arteriolar lumen. In the positive control group and the LDD high-dose group, the rabbit cells were basically neatly arranged with obvious layers, the capillary lumen was normal, and there were no edema and vacuoles. In the LDD low-dose group, the arrangement of retinal cells was basically clear and regular, and tissue edema was reduced (Figure 13).

### 3.7. Expression of MDA, TXB2, and 6-Keto-PGF1*α*

**Figure 14:**
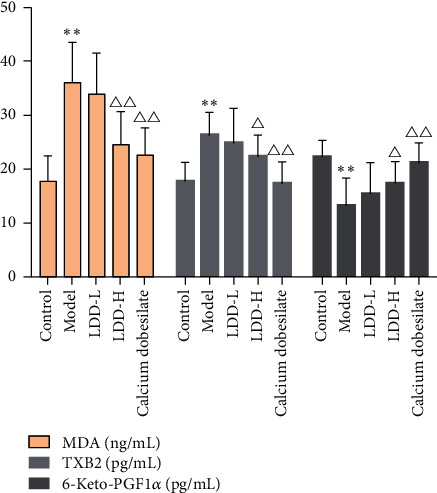
The content of MDA, TXB2, and 6-keto-PGF1*α* (LDD-L: LDD low-dose group; LDD-H: LDD high-dose group; compared with the normal control group, ^*∗∗*^*P* < 0.01; compared with the model group, ^Δ^*P* < 0.05,  ^ΔΔ^*P* < 0.01).

In the LDD low-dose group, the arrangement of retinal cells was basically clear and regular, and tissue edema was reduced. Compared with the normal control group, the content of 6-keto-PGF1*α* in the retina of the model group decreased significantly (*P* < 0.01), and the content of MDA and TXB2 increased significantly (*P* < 0.01 or *P* < 0.05). Compared with the model group, the content of 6-keto-PGF1*α* in the positive control group and LDD high-dose group increased significantly (*P* < 0.01 or *P* < 0.05), and the content of MDA and TXB2 in the positive control group and LDD high-dose group decreased significantly (*P* < 0.01 or *P* < 0.05) (Figure 14).

### 3.8. Expression of HIF-1*α* and VEGF

**Figure 15:**
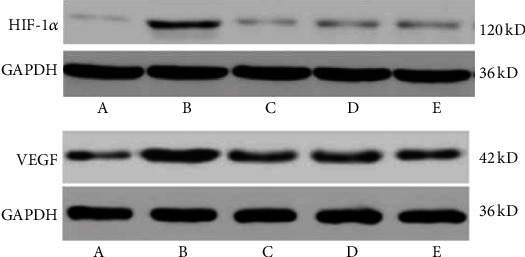
Expression of HIF-1*α* and VEGF. A: normal control group; B: model group; C: positive control group; D: LDD high-dose group; E: LDD low-dose group.

Compared with the normal control group, the expression of VEGF in the retina of the model group increased significantly (*P* < 0.01). Compared with the model group, the expression of retinal VEGF in the LDD high-dose group and the positive control group decreased significantly (*P* < 0.01), and the low-dose group had a downward trend, but there was no statistical difference (*P* > 0.05). Compared with the normal control group, the expression of HIF-1*α* in the retina of the model group was significantly increased (*P* < 0.01). Compared with the model group, the expression of HIF-1*α* in the retina in the LDD high-dose group and the positive control group was significantly reduced (*P* < 0.01), and the protein expression in the low-dose group had a downward trend, but there was no statistical significance (*P* > 0.05) (Table 4 and Figure 15).

## 4. Discussion

Three main networks (DR gene PPI network, LDD-DR PPI network, and LDD known target-DR PPI network) were constructed and analyzed in this research. The first network uncovered the possible mechanism of DR. The second network explored the potential mechanism of LDD treating DR. The third network confirmed the feasibility of the second network.

The occurrence and development of DR is closely related to the occurrence of diabetes. Long-term hyperglycemia can trigger a series of diabetes-related small vessel diseases, wherein the retinal blood vessel lesions play a crucial role in DR [36]. The hyperglycemia-associated signaling pathways and biological processes were also found in the DR gene PPI network, such as hsa04931, hsa04910, hsa04930, hsa04940, GO:0009749, GO:0006006, GO:0046326, and GO:0050796. Hyperglycemia can upregulate MMP-9 expression in vascular endothelial cells by lowering H4K20me3, increasing Ac-H3K9, and promoting the recruitment of NF-*κ*B subunit p65 at the promoter [37]. Transient hyperglycemia induces sustained transcriptional activation of the NF-*κ*B subunit p65 and is associated with H3K4 and H3K9 modification [38]. The NF-*κ*B-related pathway and biological processes were also found in the DR gene PPI network (hsa04064, GO:0043123, GO:0051092, etc.). Besides hyperglycemia, the oxidative stress result from hyperglycemia is also a major factor of vascular injury in diabetic cardiovascular complications, which may mediate diabetic microvascular leakage and retinal inflammatory cell infiltration by partially activating the classical *β*-catenin/Wnt signaling pathway [39], such as hsa04310 and GO:0007223 in the DR network. Clinical and experimental studies showed that diabetic abnormal metabolism caused by hyperglycemia can lead to imbalance of oxidation and antioxidant system, increase peroxide production, and reduce oxidative substance clearance, which eventually result in oxidative stress [40, 41]. The level of reactive oxy gen species (ROS) in the retina and its capillary cells is elevated in patients with diabetes. Such excessive ROS further damages mitochondrial DNA (mtDNA) and protein, which eventually forms a vicious circle, leading to diabetic retinal oxidative stress injury [42]. Oxidative stress-related biological processes were also found in DR networks: GO:0071456, GO:0000302, GO:0055114, GO:0042554, and so on. In the LDD-DR network, several treatment-related signaling pathways and biological processes have also been discovered. According to the enrichment results of the LDD-DR network, LDD may improve hyperglycemia (regulating hsa04910, hsa04931, hsa04930, GO:0006006, GO:0042593, GO:0009749, etc.) and oxidative stress (regulating GO:0019430, GO:0034599, GO:0032930, GO:2000378, GO:1901687, etc.). LDD can also regulate the NF-*κ*B related (hsa04064, GO:0042346, GO:0007249, etc.) and Wnt-associated (GO:0007223) pathway and biological processes.

In the DR network, several endothelial progenitor cells (EPCs) play a critical role in the repair of vascular injury in DR; when they are dysfunctional, they will affect the repair of vascular endothelial damage; hence, the EPCs' dysfunction is also an important factor in the progression of DR [43]. Some angiogenesis and retinal degeneration-related pathways and biological processes were also discovered in the DR network, such as hsa04066, GO:0045766, GO:0043536, GO:0046666, and GO:0001895. In DR, the histopathology of retinal blood vessels is the death of Rouget cells and endothelial cells; such vascular degeneration leads to retinal tissue ischemia, hypoxia and insufficient nutrient supply, blood-retinal barrier destruction, and massive release of inflammatory factors [44–47]. These pathological changes stimulate residual vascular leakage and even pathological neovascularization. Therefore, the repair and reconstruction of damaged vascular is the key to preventing the development of NPDR to PDR [44–47]. Recent studies showed that extracellular signal HIF-1a participates in the regulation of endothelial progenitor cell aggregation and adhesion to the retinal ischemic injury region through the CXCR4/SDF-1 pathway [48]; however, in the diabetic microenvironment, especially in patients with DR, the number of EPCs is significantly reduced, impairing the function of migration, adhesion, and aggregation [49, 50]. This research also found that LDD may affect the progress of angiogenesis and retinal degeneration through controlling the related pathway and processes such as hsa04722, hsa04210, GO:0043066, hsa04066, GO:0045765, and GO:0043537.

Inflammation also plays an important role in the pathogenesis of DR. The inflammation-related pathways were found in the DR network: hsa04668, hsa04068, hsa04620, GO:0006954, GO:0032757, GO:0032755, GO:0032611, etc. Currently, DR is considered to be a low-grade inflammatory disease [51]. In animal experiments, the levels of proinflammatory cytokines in the retina of diabetic animals increase, and inhibition of TNF-*α* activity plays a beneficial role in the prevention of early DR [52]. Clinical studies showed that levels of proinflammatory cytokines in vitreous humor in patients with PDR are elevated, which is associated with the severity and progression of retinal damage [53–58]. Studies showed that the incidence of DR in patients treated with salicylate for rheumatoid arthritis in the early stage of DR was reduced [59], which further confirmed the close relationship between diabetic microvascular complications and inflammation. This research found that LDD may control the inflammation in DR through hsa04068, hsa04668, GO:0032715, GO:0050728, and so on. In addition, vascular endothelial growth factor (VEGF) levels are significantly increased in the ocular tissues of patient with diabetes [59], as was found in the DR network: hsa04370, GO:0048010, GO:0010575, and GO:0038084. Another study showed that the intravitreal injection of anti-VEGF agents can inhibit the transport of leukocytes in the retina, suggesting that anti-VEGF therapy also has a therapeutic effect on retinal inflammation [60]. According to the enrichment outcomes of the LDD-DR network, LDD may inhibit DR by regulating the VEGF-related pathway and processes: hsa04370, GO:0035924, GO:0010574, GO:0030949, etc.

From a microscopic perspective, DR is associated with multiple signaling pathways. The inflammation of DR may involve in VEGF signaling pathway (hsa04370 in Table S5), NF-*κ*B signaling pathway (hsa04064 in Table S5), AMPK/mTOR signaling pathway (hsa04010 and hsa04150 in Table S5), PI3K/Akt signaling pathway (hsa04151 in Table S5), Notch/PTEN/Akt signaling pathway, and Wnt/*β*-catenin signaling pathway (hsa04310 in Table S5) [61–65]. These signaling pathways and related biological processes have also been discovered in the enrichment analysis of our DR networks (see Tables S4 and S5). Studies showed that inhibition of NF-*κ*B signaling pathway-mediated expression of inflammatory factors by catechins can improve DR, while enhancement of autophagy and AMPK/mTOR signaling by berberine can attenuate rat retinal Müller cell apoptosis resulting from long-term high glucose stimulation [61]. Another study showed that the p38-MAPK signaling pathway is activated in the microvascular disease retinopathy of the streptozotocin-induced diabetic rat model [62]. From the perspective of MicroRNA, MicroRNA-15b can regulate IRS-1 via the Wnt/*β*-catenin pathway in rat DR [63], and inhibition of microRNA-495 attenuates high glucose-induced apoptosis in retinal ganglion cells by modulating the Notch/PTEN/Akt signaling pathway. Meanwhile, new research reports that high uric acid can promote retinal inflammation and increase the activity of Notch signaling pathways based on high glucose [65]. LDD may achieve therapeutic effects by regulating them and related biological processes (see Tables S6 and S7).

Moreover, the development and progression of DR are also associated with another systemic factors such as hypertension and hypercholesterolemia [41], as was discovered in the DR network: hsa04270, GO:1904707, GO:0045907, GO:0008203, etc. They promote the complex pathological process of DR together with the above factors. This research also found that LDD may improve hypertension (GO:0008217, GO:0045909, GO:1904707, etc.) and hypercholesterolemia (0090370, GO:0006635, GO:0033344, GO:0008203, etc.) through regulating associated pathways and biological processes.

Additionally, experimental studies have shown that LDD can target PPAR signaling pathways well [66], which is consistent with the findings of this study. Current studies have shown that some signaling pathways play an important role in the pathogenesis of DR by inhibiting retinal leukocyte arrest and leakage caused by diabetes [67], in which PPAR-*γ* activators exert anti-inflammatory, antioxidative, and antiproliferative effects in retinal cells and so on [68–71]. LDD also reduces H_2_O_2_-induced phosphorylation of p38 MAPK and p44/42 MAPKs (Erk1/2) [72] (see https://www.ncbi.nlm.nih.gov/pmc/articles/PMC5609941/figure/F3/). In experiments in which LDD prolonged the lifespan of *C. elegans* and mice, microarray data showed that the longevity effect of LDD was attributed to the regulation of innate immune response, proteolysis, lipid metabolism, and oxidative stress [73]. Vascular remodeling plays an important role in atherosclerosis in the retina. This study found that LDD can regulate vascular remodeling in the retina of DR, and recent studies have shown that LDD inhibits proliferation in angiotensin II (Ang II)-treated VSMC and induces cell cycle arrest in a concentration-dependent manner [74]; the study also found that LDD at a concentration of 12 *μ*g/ml inhibits Ang II-stimulated VSMC migration and actin reorganization significantly [74]. In vitro studies showed that pretreatment with LDD-containing serum can attenuate Hcy-induced apoptosis in HUVECs [75] LDD-containing serum significantly upregulated NO release and eNOS activity in HUVEC human umbilical vein endothelial cells (HUVECs) [75]. In addition, LDD-containing serum optimized the balance between Bax and Bcl-2 and attenuated intracellular ROS production in HUVECs cells [75].

## 5. Conclusion

The therapeutic effect of LDD on DR may be achieved by interfering with the abovementioned biological processes (such as response to insulin, glucose homeostasis, regulation of angiogenesis, and inflammatory response) and signaling pathways (such as insulin signaling pathway, VEGF signaling pathway, HIF-1 signaling pathway, and ErbB signaling pathway) related to the development and progression of DR.

## Figures and Tables

**Table 1:** Cluster of the DR network.

Cluster	Score	Nodes	Edges	Gene
1	72.172	94	3356	PIK3CA, FGF2, KIT, ITGA2, CASP9, IFNG, KITLG, MMP1, CYCS, F3, FASLG, PIK3CG, IL8, TNF, PTGS2, CCL2, AR, HRAS, TF, IL13, IGF1, JAK2, MAPK14, IL1B, HSPA4, GAPDH, NOS2, ICAM1, EGR1, MAPK9, BDNF, TNFRSF1A, CASP1, MMP9, IL10, PLG, TIMP1, NGF, IL6, TNFSF10, IL4, TP53, BCL2, ALB, IGF1R, VCAM1, TLR4, NFKB1, MAP2K1, CCL5, EDN1, LEP, CXCL12, CXCR4, MAPK1, PPARG, PARP1, SPP1, MAP2K7, MTOR, CASP3, CAT, FLT1, VEGFA, ANXA5, KDR, PCNA, AKT1, BAX, CSF1, CD40, TGFB1, CD40LG, INS, STAT3, JUN, MAPK3, FOS, MAPK8, EPO, MMP2, SERPINE1, CTNNB1, SELE, TLR2, CREB1, IL2, F2, NOS3, APOE, EGFR, ACE, ACTB, EGF
2	24.721	123	1508	MMRN1, EP300, CDH5, FGB, SERPINA4, CCR2, SST, SSTR2, CD79A, RHOA, GNB3, NPY, IL18, TMSB4X, CNR1, APLNR, APLN, KNG1, MMP3, PDGFRB, CTGF, HBEGF, HMOX1, AHSG, PTGER3, SSTR1, LIF, HSP90AA1, ITGAM, INSR, IRS1, BIRC5, ADIPOQ, PXN, GAS6, MMP14, CCL4, MPO, HBA1, FOXO1, VTN, ECM1, CSF1R, SERPINF2, PGF, SOCS1, AGTR2, BDKRB2, SMAD4, MET, APOH, HGF, ELN, RPS6KB1, CCL3, HCAR2, PLAU, REN, AGT, AGTR1, TLR9, HRG, VEGFC, NPY2R, SHC1, GRB2, TIMP3, FAS, CDKN3, SP1, PF4, PLAT, VIM, TNFRSF1B, RAF1, MCL1, PTEN, CD36, SELL, PRL, SOCS3, IL1R1, TGFB2, SELP, SSTR3, ANGPT1, GFAP, TEK, PROS1, VEGFB, HSPB1, HIF1A, IL6R, CYBB, FIGF, TXN, ATM, CRP, SIRT1, CCR5, HNF4A, SOD2, IL1A, SMAD2, BDKRB1, CORT, IGF2, AKT2, CXCR3, CXCL5, CXCL10, F8, C5AR1, VWF, A2M, C5, S1PR1, IL17A, SPARC, CTLA4, SMAD3, GGT1, CXCL9
3	5.818	23	64	PRKCD, RAC1, F2RL2, TJP1, OCLN, EDNRB, GAST, ITGAL, ITGB2, UTS2, TGFBR1, NOX1, OPN4, GSR, EDN3, MLN, EDNRA, SOD1, EDN2, NFE2L2, CYBA, F2RL1, ITGAV
4	4.727	12	26	TXNRD2, SIRT6, PRDX1, EPHB4, TXN2, TKT, H6PD, ACACB, AKR1B1, GLUL, GDE1, PPM1D
5	4.5	5	9	COL4A3, COL1A1, NID1, COL4A2, COL4A1
6	3.833	13	23	HLA-DRB1, TTR, IRF8, P4HB, ENO2, PML, HLA-A, HPX, PDIA3, SOD3, AMBP, PRDX4, GPX1
7	3.733	16	28	ATF4, PPARA, IGFBP1, PRKCB, LPL, ITGB1, FGFR2, PDGFB, THBS1, RETN, SERPINC1, PTPN1, APOB, FN1, PDGFA, POMC
8	3.714	8	13	UCP1, GHRL, PPARGC1A, CNTF, UCP2, FASN, GHR, EPOR
9	3.545	23	39	PIK3R1, FGFR1, ITGA2B, GCG, TNC, MAP3K5, FADD, EIF4EBP1, MMP7, CASP7, FGF1, AKT3, TIMP2, IL2RA, ANGPT2, GDNF, VMP1, PRKCA, ETS1, OSM, BMP4, HSPA5, IGFBP3
10	3.333	4	5	ABCA4, RS1, RPE65, RBP3
11	3.333	10	15	HP, ADRB3, ARNT, ADRB1, ADORA2A, APOC3, EPAS1, GLP1R, CP, LPA
12	3	3	3	XBP1, CASP12, HSP90B1
13	3	3	3	VDR, RUNX1, BGLAP
14	3	3	3	IAPP, ADCYAP1, SCT
15	3	3	3	NPR1, NPPC, NPR2
16	3	3	3	GSTM1, OGG1, XRCC1
17	3	3	3	TBXAS1, PTGES3, PTGDS
18	3	3	3	GRIA2, GRIK2, GRIA1
19	3	3	3	SDHB, MT-ND5, MT-ND4
20	3	3	3	MAOA, GLO1, MAOB

**Table 2:** Cluster of the LDD-DR network.

Cluster	Score	Nodes	Edges	Targets and genes
1	79.229	110	4318	PTEN, HMOX1, TLR4, MAPK14, IL17A, ACTB, GAPDH, IL4, IL10, CAT, HPGDS, HSPA4, MMP2, JUN, IFNG, NFKB1, MAP2K7, MAP2K1, CTNNB1, MDM2, IL1B, CXCR4, MAPK8, PCNA, PARP1, CASP3, CALM1, CALM2, CALM3, MAPK1, MAPK3, PTGS2, MAPK9, HSP90AA1, BCL2L1, NGF, CREB1, F3, IRS1, BCL2, LEP, STAT3, CD40LG, EGF, TNFSF10, CCL5, BAX, PLG, TF, IGF1R, MTOR, PIK3CG, KIT, HRAS, KITLG, FASLG, EGR1, ANXA5, RHOA, SPP1, AR, CASP1, TP53, CSF1, AKT1, TLR2, VIM, F2, GRB2, CDK2, CASP9, SRC, ESR1, ACE, EPO, TNFRSF1A, ICAM1, JAK2, ALB, PIK3CA, IGF1, FOS, CCL2, INS, IL13, FGF2, CYCS, NOS3, VCAM1, SERPINE1, BIRC5, CXCL12, MMP9, EGFR, KDR, TNF, SHC1, TGFB1, EDN1, PPARG, IL6, REN, CDC42, TIMP1, ABL1, SELE, IL2, BDNF, NR3C1, APOE
2	23.271	119	1373	HIF1A, BDKRB2, INSR, SELP, PIK3R1, CXCL10, NPY, TIMP3, TXN, XIAP, HBEGF, PGR, SSTR1, APLN, AHSG, BDKRB1, APLNR, IL1R1, PDGFRB, AKT2, IGF2, PF4, EP300, PTPN11, CNR1, CASP7, GGT1, SERPINA1, IL18, HNF4A, CYBB, CD40, HBA1, PDGFB, HSPA5, TNFRSF1B, CTLA4, STAT1, GAS6, PRKCA, PLAU, ALDOA, SIRT1, PXN, RPS6KB1, IL1A, ELN, VEGFB, CXCL9, RAF1, CCR2, S1PR1, SOCS3, CSF1R, CCL4, FOXO1, LIF, CCR5, APOH, CFD, HRG, SELL, IL2RA, VEGFC, CDKN3, FAS, PRL, SP1, SMAD3, PTGER3, MMP1, GNB3, KNG1, TMSB4X, CDK6, CORT, SERPINF2, C5AR1, MPO, PTK2, LCK, HCAR2, GSK3B, ITGAM, SMAD4, MCL1, HGF, CXCL5, SMAD2, CTGF, F8, PROS1, MET, FGB, A2M, MMP3, C5, TEK, TGFB2, AGTR1, AGTR2, FGG, NPY2R, SSTR3, SERPINA4, MMRN1, GFAP, VTN, ATM, SPARC, TLR9, ITGA2, CRP, CXCR3, SSTR2, HSPB1, NOS2, ECM1, VWF
3	7.154	53	186	GCG, JAK3, ANGPT1, HSPA8, MMP14, HSPA1A, HCK, CSK, THBS1, SOCS1, TIMP2, RAC2, EIF4E, PLAT, CD79A, SRF, ENG, APAF1, BMP4, PTPN1, SYK, ERBB4, IL6R, TNC, MMP13, SOD2, ELANE, MAP3K5, CD36, MMP7, PDGFA, AKT3, FADD, PRKCB, EIF4EBP1, FLT1, ETS1, PGF, AGT, CDH5, TJP1, MAPK12, IFNA2, FGFR1, FGF1, IGFBP3, CCL3, KAT2B, GJA1, FN1, ADIPOQ, BMP2, ITGA2B
4	6.5	9	26	NME2, ENTPD5, NT5M, IMPDH1, CANT1, LDHB, GMPR, GMPR2, APRT
5	5.833	13	35	ALDH2, TKTL1, MTHFR, GPI, UMPS, DHFR, G6PD, ACACB, ALDH1A1, IMPDH2, TXN2, SIRT6, PPP5C
6	4.756	46	107	EDN3, CTSS, PPARA, LPL, ANGPT2, GSR, OSM, BGLAP, F2RL2, F2RL1, CD209, SST, NOX4, BTK, UTS2, IGFBP1, MLN, IL2RB, S100B, EPOR, MAPK10, LYZ, CNTF, PRKCD, RAC1, RETN, FASN, OPN4, EDNRA, VDR, EDNRB, ITGAV, ITGB1, TNFRSF11B, CA2, SOD1, EDN2, NOX1, LGALS3, GHR, PRKCQ, APOB, HMGB1, CYBA, FLT4, ZAP70
7	4.4	16	33	MTAP, SRR, SIRT5, AHCY, RHO, SDS, GLP1R, SIRT3, PYGL, AMY2A, SCT, ARF4, CBS, ADCYAP1, AMY1B, AMY1C
8	4.286	15	30	EPHB3, CRYZ, EPHB4, GART, GSTO1, HDAC8, KIF11, GSTA3, PPM1D, GSTM2, TCF7L2, GSTM1, ADH1B, GSTA1, SUV39H1
9	4	4	6	CRABP2, RARG, RXRG, RARB
10	3.9	21	39	FKBP1A, NQO1, AMBP, IRF8, RUNX1, TYMS, RAB5A, MME, GPX1, PRKACA, MTHFD1, RAB11A, ENO2, PRDX1, RARA, HLA-A, PDIA3, PML, PRDX4, TPI1, LAMP1
11	3.333	4	5	CD28, CD4, ITK, HLA-DRB1
12	3.333	4	5	PDHB, TKT, AKR1B1, TXNRD2
13	3.333	4	5	MAOA, AOC3, TPH1, MAOB
14	3.25	17	26	CD163, APOC3, AURKA, HMGCR, DUT, ADAM17, GLI1, GC, PRKAA2, APOA1, JAG1, ELAVL1, HPX, NAMPT, ATIC, CP, CCNA2
15	3	3	3	GCK, H6PD, PKLR
16	3	3	3	ACAA1, ACADM, ACAT1
17	2.833	13	17	CASP12, NRP1, TGFBR1, ITGA4, CTSB, XBP1, COL18A1, HSP90AB1, HSP90B1, ITGB3, ITGAL, PDPK1, ATF4
18	2.727	12	15	CYP2C8, GDF15, CARM1, GSTZ1, CYP2C19, RND3, SULT2A1, NR1H2, RXRA, TGFBR2, CYP2C9, ACVRL1

**Table 3:** Cluster of the LDD known target-DR network.

Cluster	Score	Nodes	Edges	Targets and genes
1	83.468	110	4549	EPO, CDK2, INS, ACTB, ALB, MAPK14, IGF1, HGF, FGF2, MMP9, GAPDH, CXCL12, IL4, KDR, HSPA4, EDN1, CTNNB1, TIMP1, MAP2K1, PCNA, CXCR4, ITGA2, BDNF, PTGS2, MAP2K7, MAPK9, PIK3CG, CD40, APOE, MAPK3, TLR4, CREB1, CALM1, IL17A, ESR1, HSP90AA1, CAT, CALM2, CALM3, AR, ICAM1, BCL2, CASP3, TNFSF10, F2, NFKB1, RELA, AKT1, NR3C1, TP53, LEP, VEGFA, CDKN1A, MTOR, NGF, EGR1, KIT, HRAS, FASLG, STAT3, ANXA5, BAX, NOS3, CCL5, PLG, MAPK8, TNF, JUN, RHOA, STAT1, TF, IGF1R, KITLG, VCAM1, CASP1, CCND1, BCL2L1, EGFR, CASP9, FOS, IL10, EGF, MMP2, MAPK1, CSF1, NFKBIA, RB1, IL6, CASP8, VIM, TLR2, RAF1, MYC, ERBB2, IL1B, CCL2, BIRC5, IL8, TGFB1, JAK2, IL2, PIK3CA, IFNG, CYCS, TNFRSF1A, PTEN, PARP1, IL13, SPP1, CD40LG
2	24.435	116	1405	ADRA2C, CHRM4, MMRN1, ACE, ATM, OPRD1, SSTR2, SOD2, BDKRB2, FLT1, SST, CTGF, CXCR3, ECM1, TGFB2, TEK, SSTR1, BDKRB1, REN, GFAP, SPARC, AKT2, PDGFRB, HBEGF, EP300, TLR9, AHSG, POMC, NPY, MAP3K5, SELP, NOS2, IRS1, GSK3B, GGT1, TIMP3, IL18, PGR, HNF4A, APLN, PXN, APLNR, HBA1, CTLA4, IL1R1, CCR2, CNR1, PF4, VEGFB, SOCS3, HIF1A, S1PR1, GAS6, RPS6KB1, CHRM2, CSF1R, HRG, OPRM1, PPARG, SIRT1, FOXO1, ELN, CCR5, CDKN3, CDK1, APOH, MMP1, CXCL9, HMOX1, SELE, FAS, SMAD3, INSR, MMP3, PLAU, PRL, C5AR1, SP1, SERPINF2, GNB3, CAV1, TMSB4X, KNG1, F3, CORT, GRB2, PTGER3, HCAR2, HSPB1, CCNB1, SMAD4, MCL1, SERPINE1, MPO, SMAD2, IL1A, ITGAM, FGB, F8, PROS1, CXCL2, A2M, CXCL11, SHC1, CRP, CXCL5, MET, IGF2, ERBB3, IRF1, C5, ADRA2A, SSTR3, AGTR2, NPY2R, SERPINA4
3	12.149	68	407	IKBKB, GCG, CDH5, EDNRB, F2RL2, IFNA2, GAST, EDNRA, CCL4, IL2RA, MLN, PIK3R1, SLC2A4, CCL3, TXN, FN1, PGF, ITGA2B, AGTR1, EDN3, SELL, VTN, OPN4, FGF1, MMP14, SOCS1, BMP4, HMGB1, HSPA5, CASP7, ADIPOQ, PRKCA, VWF, GJA1, F2R, ANGPT1, MMP7, ENG, PLAT, CD79A, EIF4EBP1, CD36, EDN2, F2RL1, NCF1, FADD, PDGFB, THBS1, IL6R, TNC, TIMP2, CHRM3, TNFRSF1B, CHUK, CHRM1, ETS1, CYBB, RUNX2, IGFBP3, ADRA1B, ADRA1D, LIF, FGFR1, AKT3, HTR2A, UTS2, ADRA1A, CHRM5
4	7	7	21	ADORA2A, DRD1, IAPP, SCT, ADM, ADRB3, ADRB1
5	6	6	15	GABRA3, GABRA2, GABRA6, GABRA5, GABRA1, BEST1
6	4.75	17	38	HNF1A, CALR, HLA-A, CTSD, H6PD, GPX1, P4HB, GCK, HK2, PRKAA2, LAMP1, TXN2, DPP4, HSP90AB1, HMGCR, PRDX4, ACACA
7	4.333	7	13	XRCC1, GSTP1, GSTM1, SIRT3, ALDH2, GSTM2, GSTT1
8	4.065	32	63	PRKCB, ATF4, SLC2A1, TJP1, RETN, PRKCD, APOB, TOP2A, YY1, ANGPT2, LPL, IGFBP1, ITGB1, PTPN1, COL18A1, AGT, PDGFA, FASN, OSM, BGLAP, TNFRSF11B, CXCL10, FLT4, VEGFC, FGFR2, GHR, IL2RB, CX3CR1, VDR, ADAM17, SRF, CX3CL1
9	4	5	8	COL4A3, COL4A1, COL4A2, NID1, COL3A1
10	3.474	20	33	ITGB2, ITGAV, PPARA, NOX4, RAC1, PPARGC1A, ITGB3, ITGAL, NOX1, APOA1, ELAVL1, NCOA1, THBD, NRP1, UCP2, CYBA, OCLN, TGFBR1, SOD1, SERPINC1
11	3.375	17	27	NCOA2, AKR1B1, HPX, RXRA, MTHFR, SOD3, CP, RARA, APOC3, GLUL, PIM1, SIRT6, TXNRD2, TKT, ACACB, LPA, CARM1
12	3	3	3	PSMD3, PSMD9, TNFSF12
13	3	3	3	ADCYAP1, GLP1R, ADRB2
14	2.8	6	7	NPPB, NPPA, NPR1, CYP1A1, POR, CYP3A4

**Table 4:** Expression of HIF-1*α* and VEGF.

Group	HIF-1a	VEGF
Normal control group	0.20 ± 0.03	0.38 ± 0.04
Model group	0.41 ± 0.06^*∗*^	0.62 ± 0.07^*∗*^
Positive control group	0.25 ± 0.04^#^	0.42 ± 0.05^#^
LDD high-dose group	0.29 ± 0.04^#^	0.44 ± 0.05^#^
LDD low-dose group	0.30 ± 0.05^#^	0.50 ± 0.07^#^

Tips: compared with the normal control group, ^*∗*^*P* < 0.05; compared with the model group, ^#^*P* < 0.05

## Data Availability

The data used to support the findings of this study are included within the article and the supplementary information files.

## Supplementary Materials

Supplementary Materials Table S1: compound targets for each compounds. Table S2: known targets for each compounds. Table S3: DR genes. Table S4: enrichment analysis of clusters based on Gene Ontology (GO) annotation of DR PPI network. Table S5: pathway enrichment analysis of DR PPI network. Table S6: enrichment analysis of clusters based on Gene Ontology (GO) annotation of LDD-DR PPI network. Table S7: pathway enrichment analysis of LDD-DR PPI network. Table S8: enrichment analysis of clusters based on Gene Ontology (GO) annotation of LDD known target-DR network. Table S9: pathway enrichment analysis of LDD known target-DR network.
