# Corrigendum to “Exploring the Pharmacological Mechanism of Liuwei Dihuang Decoction for Diabetic Retinopathy: A Systematic Biological Strategy-Based Research”

**DOI:** 10.1155/2022/9790813

**Published:** 2022-01-05

**Authors:** Mengxia Yuan, Qi He, Zhiyong Long, Xiaofei Zhu, Wang Xiang, Yonghe Wu, Shibin Lin

**Affiliations:** ^1^Joint Shantou International Eye center of Shantou University and the Chinese University of Hong Kong, Shantou City, Guangdong Province, China; ^2^Shantou University Medical College, Shantou University, Shantou, Guangdong, China; ^3^Hunan University of Chinese Medicine Affiliated People's Hospital of Ningxiang City, Ningxiang City, Hunan Province, China

In the article titled “Exploring the Pharmacological Mechanism of Liuwei Dihuang Decoction for Diabetic Retinopathy: A Systematic Biological Strategy-Based Research” [1], the order of affiliations has been corrected as above. The authors have also requested the following correction to the disclosure statement.

Disclosure

Mengxia Yuan, Shibin Lin, Qi He, and Zhiyong Long are to be considered the co-first authors. Shibin Lin is the first corresponding author because he supervised the study, and Joint Shantou International Eye Center of Shantou University and the Chinese University of Hong Kong is the first corresponding address.
