# Supplementary material for: Exploring the Pharmacological Mechanism of Liuwei Dihuang Decoction for Diabetic Retinopathy: A Systematic Biological Strategy-Based Research
Source: Evid Based Complement Alternat Med. 2021 Aug 2;2021:5544518. doi: 10.1155/2021/5544518 (PMC8356007; doi:10.1155/2021/5544518)
Supplement: Supplementary Materials — Table S1: compound targets for each compounds. Table S2: known targets for each compounds. Table S3: DR genes. Table S4: enrichment analysis of clusters based on Gene Ontology (GO) annotation of DR PPI network. Table S5: pathway enrichment analysis of DR PPI network. Table S6: enrichment analysis of clusters based on Gene Ontology (GO) annotation of LDD-DR PPI network. Table S7: pathway enrichment analysis of LDD-DR PPI network. Table S8: enrichment analysis of clusters based on Gene Ontology (GO) annotation of LDD known target-DR network. Table S9: pathway enrichment analysis of LDD known target-DR network. [file 5544518.f1.zip › 5544518.f1/Table S1.pdf]

Table S1 Compound targets for each compounds

Compounds	Compounds Targets
(-)-taxifolin	HRAS
(-)-taxifolin	UCK2
(-)-taxifolin	HSD17B1
(-)-taxifolin	HCK
(-)-taxifolin	PDE4D
(-)-taxifolin	HSP90AA1
(-)-taxifolin	FGFR1
(-)-taxifolin	DCPS
(-)-taxifolin	PDPK1
(-)-taxifolin	CDK6
(-)-taxifolin	CCNA2
(-)-taxifolin	VDR
(-)-taxifolin	CDK2
(-)-taxifolin	DCK
(-)-taxifolin	SRC
(-)-taxifolin	GSTP1
(-)-taxifolin	NT5M
(-)-taxifolin	MME
(-)-taxifolin	GPI
(-)-taxifolin	ADH
(-)-taxifolin	GART
(-)-taxifolin	AKR1B1
(-)-taxifolin	ESR1
(-)-taxifolin	BACE1
(-)-taxifolin	CBR1
(-)-taxifolin	METAP2
(-)-taxifolin	TAP1
(-)-taxifolin	PDE5A
(-)-taxifolin	CA2
(-)-taxifolin	GSTT2
(-)-taxifolin	KIT
(-)-taxifolin	GMPR
(-)-taxifolin	EIF4E
(-)-taxifolin	RAP2A
(-)-taxifolin	CTSK
(-)-taxifolin	GSTA3
(-)-taxifolin	DAPK1
(-)-taxifolin	KDR
(-)-taxifolin	INSR
(-)-taxifolin	APAF1
(-)-taxifolin	MAPK14
(-)-taxifolin	AR
(-)-taxifolin	F2
(-)-taxifolin	GSTM2
(-)-taxifolin	LCK
(-)-taxifolin	RAC1

(-)-taxifolin	AK1
(-)-taxifolin	PNMT
(-)-taxifolin	PCK1
(-)-taxifolin	PTPN1
(-)-taxifolin	DTYMK
(-)-taxifolin	HINT1
(-)-taxifolin	JAK2
(-)-taxifolin	NDST1
(-)-taxifolin	GSK3B
(-)-taxifolin	EPHA2
(-)-taxifolin	MET
(-)-taxifolin	ABO
(-)-taxifolin	ELANE
(-)-taxifolin	AMY1A
(-)-taxifolin	AMY1B
(-)-taxifolin	AMY1C
(-)-taxifolin	MTAP
(-)-taxifolin	FKBP1A
(-)-taxifolin	IL2
(-)-taxifolin	SORD
(-)-taxifolin	TEK
(-)-taxifolin	AKR1C2
(-)-taxifolin	NMNAT1
(-)-taxifolin	AURKA
(-)-taxifolin	UMPS
(-)-taxifolin	PDE4B
(-)-taxifolin	RFK
(-)-taxifolin	CASP3
(-)-taxifolin	DHODH
(-)-taxifolin	RARA
(-)-taxifolin	PFKFB1
(-)-taxifolin	ADK
(-)-taxifolin	RAB5A
(-)-taxifolin	SULT2B1
(-)-taxifolin	CASP1
(-)-taxifolin	PYGL
(-)-taxifolin	PRKACA
(-)-taxifolin	HPRT1
(-)-taxifolin	SHBG
(-)-taxifolin	RAB11A
(-)-taxifolin	BTK
(-)-taxifolin	FKBP3
(-)-taxifolin	GSTZ1
(-)-taxifolin	ITPKA
(-)-taxifolin	ARL5B
(-)-taxifolin	UAP1
(-)-taxifolin	HNMT
(-)-taxifolin	CHEK1

(-)-taxifolin	HEXB
(-)-taxifolin	GSTA1
(-)-taxifolin	BHMT
(-)-taxifolin	FGG
(-)-taxifolin	GMPR2
(-)-taxifolin	HK1
(-)-taxifolin	F11
(-)-taxifolin	SULT1A1
(-)-taxifolin	RAN
(-)-taxifolin	PIM1
(-)-taxifolin	CHIT1
(-)-taxifolin	BST1
(-)-taxifolin	PLAU
(-)-taxifolin	CDC42
(-)-taxifolin	DHFR
(-)-taxifolin	OAT
(-)-taxifolin	MAPK10
(-)-taxifolin	CTSF
(-)-taxifolin	SULT1E1
(-)-taxifolin	HDAC8
(-)-taxifolin	CLEC4M
(-)-taxifolin	FKBP1B
(-)-taxifolin	SPR
(-)-taxifolin	ABL1
(-)-taxifolin	DUT
(-)-taxifolin	TPSB2
(-)-taxifolin	REN
(-)-taxifolin	APRT
(-)-taxifolin	RAB9A
(-)-taxifolin	RAB9B
(-)-taxifolin	SSE1
(-)-taxifolin	GSR
(-)-taxifolin	MMP3
(-)-taxifolin	NR1I2
(-)-taxifolin	HSD11B1
(-)-taxifolin	RNASE3
(-)-taxifolin	ARL5A
(-)-taxifolin	LGALS3
(-)-taxifolin	IMPDH2
(-)-taxifolin	CBS
(-)-taxifolin	GSTM1
(-)-taxifolin	PLA2G2A
(-)-taxifolin	WARS
(-)-taxifolin	SHMT1
(+)-catechin	HRAS
(+)-catechin	UCK2
(+)-catechin	HSD11B1
(+)-catechin	GART

(+)-catechin	HSD17B1
(+)-catechin	MME
(+)-catechin	HSP90AA1
(+)-catechin	PRKACA
(+)-catechin	VDR
(+)-catechin	ABO
(+)-catechin	NR3C1
(+)-catechin	SHBG
(+)-catechin	GSTP1
(+)-catechin	DCK
(+)-catechin	PTPN1
(+)-catechin	BACE1
(+)-catechin	ESRRG
(+)-catechin	GSK3B
(+)-catechin	PNMT
(+)-catechin	PDPK1
(+)-catechin	FGFR1
(+)-catechin	ELANE
(+)-catechin	MAPK14
(+)-catechin	AKR1C3
(+)-catechin	PDE4B
(+)-catechin	CCNA2
(+)-catechin	PDE4D
(+)-catechin	KDR
(+)-catechin	CDK2
(+)-catechin	LCK
(+)-catechin	ESR1
(+)-catechin	AKR1B1
(+)-catechin	ZAP70
(+)-catechin	GSR
(+)-catechin	INSR
(+)-catechin	SRC
(+)-catechin	NR1H4
(+)-catechin	FKBP1A
(+)-catechin	AKR1C2
(+)-catechin	CRABP2
(+)-catechin	RAP2A
(+)-catechin	BTK
(+)-catechin	SORD
(+)-catechin	CA2
(+)-catechin	SELE
(+)-catechin	PCK1
(+)-catechin	HCK
(+)-catechin	PLA2G10
(+)-catechin	BRAF
(+)-catechin	JAK2
(+)-catechin	MAPKAPK2
(+)-catechin	MMP8

(+)-catechin	ARL5B
(+)-catechin	AR
(+)-catechin	GSTM2
(+)-catechin	MMP12
(+)-catechin	BHMT
(+)-catechin	MMP3
(+)-catechin	TGM3
(+)-catechin	CASP3
(+)-catechin	GSTM1
(+)-catechin	RAB5A
(+)-catechin	APRT
(+)-catechin	NR3C2
(+)-catechin	MAPK10
(+)-catechin	CDK6
(+)-catechin	HPRT1
(+)-catechin	MET
(+)-catechin	FECH
(+)-catechin	GPI
(+)-catechin	RAB11A
(+)-catechin	METAP2
(+)-catechin	SULT2A1
(+)-catechin	CHEK1
(+)-catechin	JAK3
(+)-catechin	THRB
(+)-catechin	KIF11
(+)-catechin	DPEP1
(+)-catechin	CBR1
(+)-catechin	THRA
(+)-catechin	AURKA
(+)-catechin	FGG
(+)-catechin	CES1
(+)-catechin	MAOB
(+)-catechin	F2
(+)-catechin	NR1H2
(+)-catechin	GMPR
(+)-catechin	PPP1CC
(+)-catechin	GSTA3
(+)-catechin	CTSB
(+)-catechin	NR1H3
(+)-catechin	MTAP
(+)-catechin	GSTA1
(+)-catechin	ESR2
(+)-catechin	IMPA1
(+)-catechin	EIF4E
(+)-catechin	EPHB4
(+)-catechin	XIAP
(+)-catechin	TGFB2
(+)-catechin	PLA2G2A

(+)-catechin	REN
(+)-catechin	CASP1
(+)-catechin	CTSK
(+)-catechin	PIM1
(+)-catechin	RARG
(+)-catechin	HPGDS
(+)-catechin	LCN2
(+)-catechin	TTR
(+)-catechin	IGF1R
(+)-catechin	IL2
(+)-catechin	PGR
(+)-catechin	MAP2K1
(+)-catechin	DHODH
(+)-catechin	PYGL
3,4-Dehydrolycopen-1	TTR
3,4-Dehydrolycopen-1	TTPA
3,4-Dehydrolycopen-1	PPP1CC
3,4-Dehydrolycopen-1	HNMT
3,4-Dehydrolycopen-1	TEK
3,4-Dehydrolycopen-1	RBP4
3,4-Dehydrolycopen-1	MET
3,4-Dehydrolycopen-1	METAP2
3,4-Dehydrolycopen-1	RXRA
3,4-Dehydrolycopen-1	PROCR
3,4-Dehydrolycopen-1	ALB
3,4-Dehydrolycopen-1	VDR
3,4-Dehydrolycopen-1	CRABP2
3,4-Dehydrolycopen-1	MAOB
3,4-Dehydrolycopen-1	RXRB
3,4-Dehydrolycopen-1	HSD17B1
3,4-Dehydrolycopen-1	RARB
3,4-Dehydrolycopen-1	LSS
3,4-Dehydrolycopen-1	CYP2C8
3,4-Dehydrolycopen-1	GSTP1
3,4-Dehydrolycopen-1	MAP2K1
3,4-Dehydrolycopen-1	RARG
3,4-Dehydrolycopen-1	PCTP
3,4-Dehydrolycopen-1	MAPK14
3,4-Dehydrolycopen-1	IL2
3,4-Dehydrolycopen-1	PPARD
3,4-Dehydrolycopen-1	CASP3
3,4-Dehydrolycopen-1	RORA
3,4-Dehydrolycopen-1	HSD11B1
3,4-Dehydrolycopen-1	ESR2
3,4-Dehydrolycopen-1	SEC14L2
3,4-Dehydrolycopen-1	THRB
3,4-Dehydrolycopen-1	BACE1
3,4-Dehydrolycopen-1	SULT2B1

3,4-Dehydrolycopen-1 TRAPPC3
3,4-Dehydrolycopen-1 FABP3
3,4-Dehydrolycopen-1 NR1I2
3,4-Dehydrolycopen-1 DPP4
3,4-Dehydrolycopen-1 FABP7
3,4-Dehydrolycopen-1 AR
3,4-Dehydrolycopen-1 F2
3,4-Dehydrolycopen-1 HSP90AA1
3,4-Dehydrolycopen-1 BRAF
3,4-Dehydrolycopen-1 PTPN1
3,4-Dehydrolycopen-1 REN
3,4-Dehydrolycopen-1 NR1H4
3,4-Dehydrolycopen-1 HNF4G
3,4-Dehydrolycopen-1 AKR1B1
3,4-Dehydrolycopen-1 MDM2
3,4-Dehydrolycopen-1 KIT
3,4-Dehydrolycopen-1 EPHB4
3,4-Dehydrolycopen-1 HDAC8
3,4-Dehydrolycopen-1 NR1I3
3,4-Dehydrolycopen-1 FABP6
3,4-Dehydrolycopen-1 PDE4D
3,4-Dehydrolycopen-1 ABL1
3,4-Dehydrolycopen-1 CDK2
3,4-Dehydrolycopen-1 FECH
3,4-Dehydrolycopen-1 CHEK1
3,4-Dehydrolycopen-1 FGFR1
3,4-Dehydrolycopen-1 MMP12
3,4-Dehydrolycopen-1 PDE4B
3,4-Dehydrolycopen-1 NR3C1
3,4-Dehydrolycopen-1 CA2
3,4-Dehydrolycopen-1 DHODH
3,4-Dehydrolycopen-1 CTSK
3,4-Dehydrolycopen-1 MMP3
3,4-Dehydrolycopen-1 GSTA1
3,4-Dehydrolycopen-1 PRKACA
3,4-Dehydrolycopen-1 MAPK10
3,4-Dehydrolycopen-1 FNTA
3,4-Dehydrolycopen-1 F10
3,4-Dehydrolycopen-1 PPARG
3,4-Dehydrolycopen-1 NR1H2
3,4-Dehydrolycopen-1 BLVRB
3,4-Dehydrolycopen-1 HPGDS
3,4-Dehydrolycopen-1 ITGAL
3,4-Dehydrolycopen-1 FKBP1A
3,4-Dehydrolycopen-1 PLA2G2A
3,4-Dehydrolycopen-1 PGR
3,4-Dehydrolycopen-1 GRB2
3,4-Dehydrolycopen-1 HMGCR

3,4-Dehydrolycopen-1 ESR1
3,4-Dehydrolycopen-1 ADK
3,4-Dehydrolycopen-1 KDR
3,4-Dehydrolycopen-1 MAPK1
3,4-Dehydrolycopen-1 WAS
3,4-Dehydrolycopen-1 KIF11
3,4-Dehydrolycopen-1 MMP2
3,4-Dehydrolycopen-1 SHBG
3,4-Dehydrolycopen-1 PTPN11
3,4-Dehydrolycopen-1 SULT2A1
3,4-Dehydrolycopen-1 ADH
3,4-Dehydrolycopen-1 GC
3,4-Dehydrolycopen-1 MMP8
3,4-Dehydrolycopen-1 JAK3
3,4-Dehydrolycopen-1 PIM1
3,4-Dehydrolycopen-1 TGFBR1
3,4-Dehydrolycopen-1 PPARA
3,4-Dehydrolycopen-1 LCK
3,4-Dehydrolycopen-1 PARP1
3,4-Dehydrolycopen-1 NR1H3
3,4-Dehydrolycopen-1 CASP1
3,4-Dehydrolycopen-1 CALM1
3,4-Dehydrolycopen-1 CALM2
3,4-Dehydrolycopen-1 CALM3
3,4-Dehydrolycopen-1 PSAP
3,4-Dehydrolycopen-1 SERPINA1
3,4-Dehydrolycopen-1 AKR1C2
3,4-Dehydrolycopen-1 RARA
3,4-Dehydrolycopen-1 SYK
3,4-Dehydrolycopen-1 EGFR
3,4-Dehydrolycopen-1 TGM3
3,4-Dehydrolycopen-1 DPEP1
3,4-Dehydrolycopen-1 THRA
3,4-Dehydrolycopen-1 MAPKAPK2
3,4-Dehydrolycopen-1 PDPK1
3,4-Dehydrolycopen-1 ERBB4
3,4-Dehydrolycopen-1 GM2A
3,4-Dehydrolycopen-1 SULT1E1
3,4-Dehydrolycopen-1 PRKCQ
3,4-Dehydrolycopen-1 MMP13
3,4-Dehydrolycopen-1 ERRA
3,4-Dehydrolycopen-1 ADAM17
3,4-Dehydrolycopen-1 PIK3R1
3,4-Dehydrolycopen-1 ABO
3,4-Dehydrolycopen-1 SRC
3,4-Dehydrolycopen-1 CTSB
3,4-Dehydrolycopen-1 ZAP70
3,4-Dehydrolycopen-1 BCL2L1

3,4-Dehydrolycopen-1	MME
3,4-Dehydrolycopen-1	LCN2
3,4-Dehydrolycopen-1	ITK
4-O-methylpaeoniflori	VDR
4-O-methylpaeoniflori	MME
4-O-methylpaeoniflori	TTR
4-O-methylpaeoniflori	AR
4-O-methylpaeoniflori	TTPA
4-O-methylpaeoniflori	RXRB
4-O-methylpaeoniflori	MAP2K1
4-O-methylpaeoniflori	PRKACA
4-O-methylpaeoniflori	FGFR1
4-O-methylpaeoniflori	HMGCR
4-O-methylpaeoniflori	BACE1
4-O-methylpaeoniflori	HSD17B1
4-O-methylpaeoniflori	AKR1C3
4-O-methylpaeoniflori	METAP2
4-O-methylpaeoniflori	MMP3
4-O-methylpaeoniflori	MET
4-O-methylpaeoniflori	CASP3
4-O-methylpaeoniflori	HSP90AA1
4-O-methylpaeoniflori	DHODH
4-O-methylpaeoniflori	FKBP1A
4-O-methylpaeoniflori	RBP4
4-O-methylpaeoniflori	KDR
4-O-methylpaeoniflori	CA2
4-O-methylpaeoniflori	RARG
4-O-methylpaeoniflori	SHBG
4-O-methylpaeoniflori	PPP1CC
4-O-methylpaeoniflori	MAPK10
4-O-methylpaeoniflori	ALB
4-O-methylpaeoniflori	RORA
4-O-methylpaeoniflori	MAPK14
4-O-methylpaeoniflori	CRABP2
4-O-methylpaeoniflori	MAOB
4-O-methylpaeoniflori	GSTA1
4-O-methylpaeoniflori	BRAF
4-O-methylpaeoniflori	THRB
4-O-methylpaeoniflori	RARA
4-O-methylpaeoniflori	GSTP1
4-O-methylpaeoniflori	MMP12
4-O-methylpaeoniflori	LCK
4-O-methylpaeoniflori	DPEP1
4-O-methylpaeoniflori	CDK2
4-O-methylpaeoniflori	F2
4-O-methylpaeoniflori	NR1I2
4-O-methylpaeoniflori	RARB
4-O-methylpaeoniflori	CASP1

4-O-methylpaeoniflori	CYP2C9
4-O-methylpaeoniflori	ERBB4
4-O-methylpaeoniflori	HSD11B1
4-O-methylpaeoniflori	AKR1B1
4-O-methylpaeoniflori	FABP7
4-O-methylpaeoniflori	NR1H2
4-O-methylpaeoniflori	KIT
4-O-methylpaeoniflori	PDE5A
4-O-methylpaeoniflori	TGFB2
4-O-methylpaeoniflori	NR3C1
4-O-methylpaeoniflori	PTPN1
4-O-methylpaeoniflori	NR1H3
4-O-methylpaeoniflori	PDE4D
4-O-methylpaeoniflori	SYK
4-O-methylpaeoniflori	DCK
4-O-methylpaeoniflori	REN
4-O-methylpaeoniflori	PDE4B
4-O-methylpaeoniflori	FABP3
4-O-methylpaeoniflori	PDPK1
4-O-methylpaeoniflori	ELANE
4-O-methylpaeoniflori	CTSK
4-O-methylpaeoniflori	SULT1E1
4-O-methylpaeoniflori	RXRA
4-O-methylpaeoniflori	PLA2G2A
4-O-methylpaeoniflori	PGR
4-O-methylpaeoniflori	PPARG
4-O-methylpaeoniflori	CHEK1
4-O-methylpaeoniflori	MMP13
4-O-methylpaeoniflori	PROCR
4-O-methylpaeoniflori	ESR1
4-O-methylpaeoniflori	AKR1C2
4-O-methylpaeoniflori	DPP4
4-O-methylpaeoniflori	PPARD
4-O-methylpaeoniflori	FECH
4-O-methylpaeoniflori	FABP6
4-O-methylpaeoniflori	KIF11
4-O-methylpaeoniflori	SULT2A1
4-O-methylpaeoniflori	PNMT
4-O-methylpaeoniflori	TRAPPC3
4-O-methylpaeoniflori	TGM3
4-O-methylpaeoniflori	LTA4H
4-O-methylpaeoniflori	CCNA2
4-O-methylpaeoniflori	ABL1
4-O-methylpaeoniflori	NR3C2
4-O-methylpaeoniflori	PYGL
4-O-methylpaeoniflori	NR1H4
4-O-methylpaeoniflori	F10
4-O-methylpaeoniflori	ABO

4-O-methylpaeoniflori	IL2
4-O-methylpaeoniflori	PCTP
4-O-methylpaeoniflori	GART
4-O-methylpaeoniflori	CTSS
4-O-methylpaeoniflori	AKR1C1
4-O-methylpaeoniflori	FKBP3
4-O-methylpaeoniflori	GCK
4-O-methylpaeoniflori	MAPK1
4-O-methylpaeoniflori	CTNNA1
4-O-methylpaeoniflori	INSR
4-O-methylpaeoniflori	CTSB
4-O-methylpaeoniflori	HNF4G
4-O-methylpaeoniflori	ADK
4-O-methylpaeoniflori	ADAM17
4-O-methylpaeoniflori	MDM2
4-O-methylpaeoniflori	NQO1
4-O-methylpaeoniflori	EIF4E
7,9(11)-dehydropachy	TTR
7,9(11)-dehydropachy	RBP4
7,9(11)-dehydropachy	CRABP2
7,9(11)-dehydropachy	HSD11B1
7,9(11)-dehydropachy	HSD17B1
7,9(11)-dehydropachy	PPP1CC
7,9(11)-dehydropachy	TTPA
7,9(11)-dehydropachy	NR1I2
7,9(11)-dehydropachy	RXRA
7,9(11)-dehydropachy	FABP6
7,9(11)-dehydropachy	RORA
7,9(11)-dehydropachy	SHBG
7,9(11)-dehydropachy	DHODH
7,9(11)-dehydropachy	MAOB
7,9(11)-dehydropachy	PTPN1
7,9(11)-dehydropachy	VDR
7,9(11)-dehydropachy	CA2
7,9(11)-dehydropachy	RARB
7,9(11)-dehydropachy	RARG
7,9(11)-dehydropachy	LSS
7,9(11)-dehydropachy	AKR1C2
7,9(11)-dehydropachy	SULT2B1
7,9(11)-dehydropachy	MAP2K1
7,9(11)-dehydropachy	MAPK14
7,9(11)-dehydropachy	CCNA2
7,9(11)-dehydropachy	METAP2
7,9(11)-dehydropachy	NR3C2
7,9(11)-dehydropachy	CHEK1
7,9(11)-dehydropachy	BACE1
7,9(11)-dehydropachy	BRAF
7,9(11)-dehydropachy	F10

7,9(11)-dehydropachy AKR1C3
7,9(11)-dehydropachy HNMT
7,9(11)-dehydropachy CYP2C8
7,9(11)-dehydropachy MMP3
7,9(11)-dehydropachy RARA
7,9(11)-dehydropachy AR
7,9(11)-dehydropachy LTA4H
7,9(11)-dehydropachy HSP90AA1
7,9(11)-dehydropachy NR3C1
7,9(11)-dehydropachy ALB
7,9(11)-dehydropachy FABP7
7,9(11)-dehydropachy PPARD
7,9(11)-dehydropachy PDPK1
7,9(11)-dehydropachy THRB
7,9(11)-dehydropachy PROCR
7,9(11)-dehydropachy PCTP
7,9(11)-dehydropachy IL2
7,9(11)-dehydropachy MAPK10
7,9(11)-dehydropachy HMGCR
7,9(11)-dehydropachy GSTA1
7,9(11)-dehydropachy FECH
7,9(11)-dehydropachy SEC14L2
7,9(11)-dehydropachy PDE4D
7,9(11)-dehydropachy RXRB
7,9(11)-dehydropachy FABP3
7,9(11)-dehydropachy F2
7,9(11)-dehydropachy MET
7,9(11)-dehydropachy FKBP1A
7,9(11)-dehydropachy REN
7,9(11)-dehydropachy NR1H2
7,9(11)-dehydropachy TRAPPC3
7,9(11)-dehydropachy CASP3
7,9(11)-dehydropachy CDK2
7,9(11)-dehydropachy ABO
7,9(11)-dehydropachy TGFBR1
7,9(11)-dehydropachy DPEP1
7,9(11)-dehydropachy NR1H4
7,9(11)-dehydropachy ESR1
7,9(11)-dehydropachy MMP12
7,9(11)-dehydropachy LCK
7,9(11)-dehydropachy ADK
7,9(11)-dehydropachy AKR1B1
7,9(11)-dehydropachy ABL1
7,9(11)-dehydropachy SULT2A1
7,9(11)-dehydropachy MMP2
7,9(11)-dehydropachy GC
7,9(11)-dehydropachy ELANE
7,9(11)-dehydropachy PLA2G2A

7,9(11)-dehydropachy GSK3B
7,9(11)-dehydropachy PDE4B
7,9(11)-dehydropachy MMP13
7,9(11)-dehydropachy UCK2
7,9(11)-dehydropachy KIF11
7,9(11)-dehydropachy PSAP
7,9(11)-dehydropachy ESR2
7,9(11)-dehydropachy CASP1
7,9(11)-dehydropachy AURKA
7,9(11)-dehydropachy DPP4
7,9(11)-dehydropachy GRB2
7,9(11)-dehydropachy KDR
7,9(11)-dehydropachy CTSK
7,9(11)-dehydropachy HDAC8
7,9(11)-dehydropachy TEK
7,9(11)-dehydropachy ADH
7,9(11)-dehydropachy SULT1E1
7,9(11)-dehydropachy TGM3
7,9(11)-dehydropachy PRKACA
7,9(11)-dehydropachy PGR
7,9(11)-dehydropachy HPGDS
7,9(11)-dehydropachy BLVRB
7,9(11)-dehydropachy ERBB4
7,9(11)-dehydropachy CTSB
7,9(11)-dehydropachy ADAM17
7,9(11)-dehydropachy CTSS
7,9(11)-dehydropachy INSR
7,9(11)-dehydropachy MMP8
7,9(11)-dehydropachy SYK
7,9(11)-dehydropachy WAS
7,9(11)-dehydropachy MTAP
7,9(11)-dehydropachy PRKCQ
7,9(11)-dehydropachy GSTP1
7,9(11)-dehydropachy TGFB2
7,9(11)-dehydropachy MAPK8
7,9(11)-dehydropachy NR1H3
7,9(11)-dehydropachy ZAP70
7,9(11)-dehydropachy GM2A
7,9(11)-dehydropachy PARP1
7,9(11)-dehydropachy MAPKAPK2
7,9(11)-dehydropachy MDM2
7,9(11)-dehydropachy ITK
7,9(11)-dehydropachy NQO1
7,9(11)-dehydropachy PTPN11
7,9(11)-dehydropachy FGFR1
7,9(11)-dehydropachy EIF4E
7,9(11)-dehydropachy HCK
7,9(11)-dehydropachy PLAU

7,9(11)-dehydropachy	SRC
7,9(11)-dehydropachy	HNF4G
Acteoside	UCK2
Acteoside	ABO
Acteoside	GSTP1
Acteoside	HRAS
Acteoside	SIRT5
Acteoside	INSR
Acteoside	DAPK1
Acteoside	LCK
Acteoside	AHCY
Acteoside	GP1BA
Acteoside	PFKFB1
Acteoside	SELE
Acteoside	GSTM2
Acteoside	RAB5A
Acteoside	BACE1
Acteoside	BST1
Acteoside	SPR
Acteoside	PCK1
Acteoside	DTYMK
Acteoside	SULT2B1
Acteoside	EEA1
Acteoside	RAP2A
Acteoside	CA2
Acteoside	ESR2
Acteoside	ADAM33
Acteoside	CDC42
Acteoside	HNMT
Acteoside	HCK
Acteoside	HADH
Acteoside	EIF4E
Acteoside	PNMT
Acteoside	UAP1
Acteoside	GSK3B
Acteoside	MAPK14
Acteoside	VDR
Acteoside	CASP3
Acteoside	HSD17B1
Acteoside	PKLR
Acteoside	SERPINA1
Acteoside	SRC
Acteoside	ELANE
Acteoside	APAF1
Acteoside	HSD11B1
Acteoside	CRABP2
Acteoside	LGALS3
Acteoside	GSTT2

Acteoside	DDX39B
Acteoside	NT5M
Acteoside	GMPR2
Acteoside	STAT1
Acteoside	BTK
Acteoside	NOS2
Acteoside	HINT1
Acteoside	ANG
Acteoside	GPI
Acteoside	CDK2
Acteoside	RNASE2
Acteoside	MTAP
Acteoside	CHIT1
Acteoside	PIM1
Acteoside	RFK
Acteoside	ACAT1
Acteoside	HPRT1
Acteoside	CD209
Acteoside	PDE4B
Acteoside	SHBG
Acteoside	AMY1A
Acteoside	AMY1B
Acteoside	AMY1C
Acteoside	DHFR
Acteoside	CHEK1
Acteoside	IMPDH2
Acteoside	FGG
Acteoside	GSTA1
Acteoside	PDE4D
Acteoside	THRA
Acteoside	AMY2A
Acteoside	GSTA3
Acteoside	PPARG
Acteoside	CTSK
Acteoside	F2
Acteoside	HSP90AA1
Acteoside	DPEP1
Acteoside	SRM
Acteoside	BIRC7
Acteoside	RAC1
Acteoside	ARL5B
Acteoside	MAPK10
Acteoside	GMPR
Acteoside	FKBP1A
Acteoside	MME
Acteoside	AURKA
Acteoside	ADH
Acteoside	TGM3

Acteoside	DUT
Acteoside	IMPDH1
Acteoside	ZAP70
Acteoside	DCPS
Acteoside	GSTM1
Acteoside	GLO1
Acteoside	CCL5
Acteoside	AKR1B1
Acteoside	ALDOA
Acteoside	AKR1C2
Acteoside	PLAU
Acteoside	MMP3
Acteoside	TTR
Acteoside	TAP1
Acteoside	PTPN1
Acteoside	AKT1
Acteoside	ARG2
Acteoside	ADK
Acteoside	RND3
Acteoside	PDPK1
Acteoside	WARS
Acteoside	DCK
Acteoside	PYGL
Acteoside	ME2
Acteoside	GCK
Acteoside	TEK
Acteoside	BHMT
Acteoside	MAPKAPK2
Acteoside	TTPA
Acteoside	PAPSS1
Acteoside	ARL5A
Acteoside	SULT2A1
Acteoside	HMGCR
Acteoside	METAP2
Acteoside	GSTZ1
Acteoside	PPP1CC
Acteoside	ATIC
Acteoside	ESRRG
Acteoside	KIF11
Acteoside	LDHB
Acteoside	REG1A
Acteoside	PDE5A
Acteoside	MMP12
Acteoside	TNK2
Acteoside	OAT
Acteoside	RARA
Acteoside	NMNAT1
Acteoside	CTSS

Acteoside	LYZ
Acteoside	MAPK12
Acteoside	SULT1A1
Acteoside	HDAC8
Acteoside	GRB2
Acteoside	REN
Acteoside	KIT
Acteoside	BCAT2
Acteoside	ALB
Acteoside	NME2
Acteoside	MIF
Acteoside	CLK1
Acteoside	CDK7
Acteoside	AMD1
Acteoside	GNPDA1
Acteoside	GNPDA2
Acteoside	ESR1
Acteoside	DOT1L
Acteoside	CCNA2
Acteoside	HK1
Acteoside	MMP9
Acteoside	SDS
Acteoside	FABP6
Acteoside	CES1
Acteoside	F11
Acteoside	MAN1B1
Acteoside	PAH
Acteoside	TPSB2
Acteoside	JAK3
AIDS180907	HSD17B1
AIDS180907	TTR
AIDS180907	CRABP2
AIDS180907	TTPA
AIDS180907	BACE1
AIDS180907	HMGCR
AIDS180907	LSS
AIDS180907	PDPK1
AIDS180907	VDR
AIDS180907	METAP2
AIDS180907	MET
AIDS180907	TEK
AIDS180907	MAOB
AIDS180907	UCK2
AIDS180907	MAPK14
AIDS180907	THRB
AIDS180907	CA2
AIDS180907	PROCR
AIDS180907	RORA

AIDS180907	PCTP
AIDS180907	CHEK1
AIDS180907	LCK
AIDS180907	NR1I2
AIDS180907	PDE4D
AIDS180907	F2
AIDS180907	REN
AIDS180907	AKR1B1
AIDS180907	CASP1
AIDS180907	DHODH
AIDS180907	RBP4
AIDS180907	NT5M
AIDS180907	GSTA1
AIDS180907	F10
AIDS180907	SULT2B1
AIDS180907	TRAPPC3
AIDS180907	GSR
AIDS180907	HSD11B1
AIDS180907	RARG
AIDS180907	SRC
AIDS180907	MME
AIDS180907	SHBG
AIDS180907	PCK1
AIDS180907	ALB
AIDS180907	TGFBR1
AIDS180907	PTPN1
AIDS180907	FABP6
AIDS180907	ESRRG
AIDS180907	RARA
AIDS180907	KDR
AIDS180907	KIT
AIDS180907	RXRA
AIDS180907	ESR1
AIDS180907	PPP1CC
AIDS180907	MAP2K1
AIDS180907	FKBP1A
AIDS180907	MMP3
AIDS180907	KIF11
AIDS180907	AR
AIDS180907	PRKACA
AIDS180907	S100A9
AIDS180907	CDK2
AIDS180907	SYK
AIDS180907	XIAP
AIDS180907	IL2
AIDS180907	BLVRB
AIDS180907	ADK
AIDS180907	MDM2

AIDS180907	SULT2A1
AIDS180907	MMP8
AIDS180907	ABO
AIDS180907	LTA4H
AIDS180907	ELANE
AIDS180907	MMP12
AIDS180907	ITGAL
AIDS180907	WAS
AIDS180907	PPARG
AIDS180907	ERBB4
AIDS180907	THRA
AIDS180907	BRAF
AIDS180907	CTSK
AIDS180907	HPGDS
AIDS180907	PDE4B
AIDS180907	FECH
AIDS180907	ADH
AIDS180907	CASP3
AIDS180907	ADAM33
AIDS180907	HNF4G
AIDS180907	ABL1
AIDS180907	PGF
AIDS180907	HEXB
AIDS180907	GSTP1
AIDS180907	CYP2C9
AIDS180907	HSP90AA1
AIDS180907	SEC14L2
AIDS180907	TYMS
AIDS180907	RARB
AIDS180907	MMP2
AIDS180907	GM2A
AIDS180907	MAPK10
AIDS180907	AKR1C2
AIDS180907	ESR2
AIDS180907	GRB2
AIDS180907	NR3C1
AIDS180907	DAPK1
AIDS180907	GSTT2
AIDS180907	PPARD
AIDS180907	PPARA
AIDS180907	AURKA
AIDS180907	HCK
AIDS180907	GSTM2
AIDS180907	JAK3
AIDS180907	PLK1
AIDS180907	ANXA5
AIDS180907	AMY2A
AIDS180907	INSR

AIDS180907	PARP1
AIDS180907	CDK5R1
AIDS180907	ADH1C
AIDS180907	ADAM17
AIDS180907	GSK3B
AIDS180907	PAK7
AIDS180907	PTPN11
AIDS180907	AKR1C3
AIDS180907	DPP4
AIDS180907	MAPKAPK2
AIDS180907	PLA2G2A
AIDS180907	CALM1
AIDS180907	CALM2
AIDS180907	CALM3
Alisol B	RORA
Alisol B	MAOB
Alisol B	HSD17B1
Alisol B	UCK2
Alisol B	CRABP2
Alisol B	TTR
Alisol B	RARG
Alisol B	VDR
Alisol B	SULT2A1
Alisol B	HSD11B1
Alisol B	FKBP1A
Alisol B	KDR
Alisol B	PRKACA
Alisol B	PPP1CC
Alisol B	SHBG
Alisol B	PGR
Alisol B	RXRB
Alisol B	SULT2B1
Alisol B	PLA2G2A
Alisol B	NR1I2
Alisol B	AR
Alisol B	THRB
Alisol B	RBP4
Alisol B	MAP2K1
Alisol B	CHEK1
Alisol B	NR3C2
Alisol B	HSP90AA1
Alisol B	RXRA
Alisol B	HNF4G
Alisol B	THRA
Alisol B	TTPA
Alisol B	NR3C1
Alisol B	ANXA5
Alisol B	AKR1C3

Alisol B	ALB
Alisol B	PGF
Alisol B	PPARA
Alisol B	PCTP
Alisol B	FABP3
Alisol B	LTA4H
Alisol B	HPGDS
Alisol B	CDK2
Alisol B	GSTP1
Alisol B	PROCR
Alisol B	GSTA1
Alisol B	METAP2
Alisol B	BACE1
Alisol B	CTSK
Alisol B	ADK
Alisol B	FECH
Alisol B	PDE4B
Alisol B	CCNA2
Alisol B	HNMT
Alisol B	ADAM17
Alisol B	CA2
Alisol B	MMP3
Alisol B	TGM3
Alisol B	NR1H2
Alisol B	TRAPPC3
Alisol B	FABP6
Alisol B	MMP2
Alisol B	ESRRG
Alisol B	HCK
Alisol B	JAK3
Alisol B	DHODH
Alisol B	FABP7
Alisol B	AKR1C2
Alisol B	RARB
Alisol B	REN
Alisol B	FNTA
Alisol B	MET
Alisol B	ITGAL
Alisol B	MMP12
Alisol B	LCK
Alisol B	NR1H3
Alisol B	F2
Alisol B	ABL1
Alisol B	ESR1
Alisol B	ITK
Alisol B	PDPK1
Alisol B	PTPN1
Alisol B	SRC

Alisol B	FGFR1
Alisol B	JAK2
Alisol B	HMGCR
Alisol B	MTAP
Alisol B	GSK3B
Alisol B	PRKCQ
Alisol B	ABO
Alisol B	MAPK14
Alisol B	GC
Alisol B	HDAC8
Alisol B	PDE4D
Alisol B	AKR1B1
Alisol B	LCN2
Alisol B	GRB2
Alisol B	CTSS
Alisol B	DPP4
Alisol B	GM2A
Alisol B	MAPK1
Alisol B	MAPKAPK2
Alisol B	DPEP1
Alisol B	CALM1
Alisol B	CALM2
Alisol B	CALM3
Alisol B	PARP1
Alisol B	NR1I3
Alisol B	KIF11
Alisol B	TEK
Alisol B	ADH
Alisol B	HSP90AB1
Alisol B	PSAP
Alisol B	IL2
Alisol B	LSS
Alisol B	CYP2C8
Alisol B	WAS
Alisol B	MMP13
Alisol B	CASP3
Alisol B	MDM2
Alisol B	ESR2
Alisol B	PIM1
Alisol B	RARA
Alisol B	INSR
Alisol B	BCAT2
Alisol B	CTSB
Alisol B	ADH1C
Alisol B	SULT1E1
Alisol B	SYK
Alisol B	AKR1C1
Alisol B	F10

Alisol B23 acetate	HNMT
Alisol B23 acetate	RXRB
Alisol B23 acetate	FABP3
Alisol B23 acetate	HSD17B1
Alisol B23 acetate	CRABP2
Alisol B23 acetate	FKBP1A
Alisol B23 acetate	TTR
Alisol B23 acetate	RXRA
Alisol B23 acetate	GSTP1
Alisol B23 acetate	SHBG
Alisol B23 acetate	NR3C2
Alisol B23 acetate	THRA
Alisol B23 acetate	AKR1B1
Alisol B23 acetate	FABP6
Alisol B23 acetate	MMP3
Alisol B23 acetate	HNF4G
Alisol B23 acetate	RBP4
Alisol B23 acetate	TTPA
Alisol B23 acetate	FABP7
Alisol B23 acetate	PDE4B
Alisol B23 acetate	MAOB
Alisol B23 acetate	PTPN1
Alisol B23 acetate	NR1I2
Alisol B23 acetate	RARG
Alisol B23 acetate	PPARA
Alisol B23 acetate	PPP1CC
Alisol B23 acetate	CDK2
Alisol B23 acetate	AR
Alisol B23 acetate	ABO
Alisol B23 acetate	PCTP
Alisol B23 acetate	PLA2G2A
Alisol B23 acetate	METAP2
Alisol B23 acetate	GSTA1
Alisol B23 acetate	NR3C1
Alisol B23 acetate	BACE1
Alisol B23 acetate	ALB
Alisol B23 acetate	CTSK
Alisol B23 acetate	SEC14L2
Alisol B23 acetate	THRB
Alisol B23 acetate	CA2
Alisol B23 acetate	MAP2K1
Alisol B23 acetate	PPARG
Alisol B23 acetate	PROCR
Alisol B23 acetate	TGM3
Alisol B23 acetate	PIK3R1
Alisol B23 acetate	HSD11B1
Alisol B23 acetate	RARB
Alisol B23 acetate	HSP90AA1

Alisol B23 acetate	PGR
Alisol B23 acetate	CTNNA1
Alisol B23 acetate	TRAPPC3
Alisol B23 acetate	NR1H2
Alisol B23 acetate	F2
Alisol B23 acetate	VDR
Alisol B23 acetate	SULT2A1
Alisol B23 acetate	F10
Alisol B23 acetate	AKR1C3
Alisol B23 acetate	CCNA2
Alisol B23 acetate	MMP2
Alisol B23 acetate	CASP1
Alisol B23 acetate	RORA
Alisol B23 acetate	ADK
Alisol B23 acetate	ESR1
Alisol B23 acetate	TGFBR1
Alisol B23 acetate	SULT1E1
Alisol B23 acetate	PGF
Alisol B23 acetate	F7
Alisol B23 acetate	TEK
Alisol B23 acetate	IL2
Alisol B23 acetate	MET
Alisol B23 acetate	CASP3
Alisol B23 acetate	JAK3
Alisol B23 acetate	HMGCR
Alisol B23 acetate	FNTA
Alisol B23 acetate	DHODH
Alisol B23 acetate	ADH
Alisol B23 acetate	GSK3B
Alisol B23 acetate	DCK
Alisol B23 acetate	MDM2
Alisol B23 acetate	PDE4D
Alisol B23 acetate	MAPKAPK2
Alisol B23 acetate	MAPK14
Alisol B23 acetate	MME
Alisol B23 acetate	LCK
Alisol B23 acetate	MMP12
Alisol B23 acetate	PRKACA
Alisol B23 acetate	KDR
Alisol B23 acetate	AKR1C2
Alisol B23 acetate	ESR2
Alisol B23 acetate	GRB2
Alisol B23 acetate	ITGAL
Alisol B23 acetate	ERBB4
Alisol B23 acetate	RARA
Alisol B23 acetate	FECH
Alisol B23 acetate	DPP4
Alisol B23 acetate	NR1H3

Alisol B23 acetate	FGFR1
Alisol B23 acetate	ADH1C
Alisol B23 acetate	DPEP1
Alisol B23 acetate	ANXA5
Alisol B23 acetate	REN
Alisol B23 acetate	CALM1
Alisol B23 acetate	CALM2
Alisol B23 acetate	CALM3
Alisol B23 acetate	PRKCQ
Alisol B23 acetate	AKR1C1
Alisol B23 acetate	KIT
Alisol B23 acetate	ZAP70
Alisol B23 acetate	MMP13
Alisol B23 acetate	LCN2
Alisol B23 acetate	NQO1
Alisol B23 acetate	SORD
Alisol B23 acetate	CTSS
Alisol B23 acetate	HPGDS
Alisol B23 acetate	BRAF
Alisol B23 acetate	GC
Alisol B23 acetate	S100A9
Alisol B23 acetate	CYP2C8
Alisol B23 acetate	MTHFD1
Alisol B23 acetate	ELANE
Alisol B23 acetate	PDE3B
Alisol B23 acetate	HDAC8
Alisol B23 acetate	CYP2C9
Alisol B23 acetate	SRC
Alisol B23 acetate	CTSB
Alisol B23 acetate	SULT2B1
Alisol B23 acetate	PSAP
Alisol B23 acetate	LTA4H
Alisol B23 acetate	WAS
Alisol B23 acetate	BCL2L1
Alisol B23 acetate	BIRC7
Alisol B23 acetate	CES1
Alisol B23 acetate	SYK
Alisol B23 acetate	PDE5A
Alisol B23 acetate	ITK
Alisol B23 acetate	ADAM17
Alisol B23 acetate	BCAT2
Alisol B23 acetate	MTAP
Alisol B23 acetate	PIM1
Alisol B23 acetate	ESRRG
Alisol B23 acetate	INSR
Alisol C	VDR
Alisol C	TTR
Alisol C	AR

Alisol C	FGFR1
Alisol C	HSP90AA1
Alisol C	CRABP2
Alisol C	KDR
Alisol C	TTPA
Alisol C	PPP1CC
Alisol C	FKBP1A
Alisol C	FABP6
Alisol C	HMGCR
Alisol C	TGFB2
Alisol C	BACE1
Alisol C	PDE4B
Alisol C	PCTP
Alisol C	MMP3
Alisol C	FABP3
Alisol C	RBP4
Alisol C	RXRA
Alisol C	SHBG
Alisol C	HSD17B1
Alisol C	CA2
Alisol C	PTPN1
Alisol C	PROCR
Alisol C	AKR1B1
Alisol C	RARB
Alisol C	SULT2A1
Alisol C	NR1I2
Alisol C	PRKACA
Alisol C	MAPK14
Alisol C	MAOB
Alisol C	METAP2
Alisol C	CASP3
Alisol C	ADK
Alisol C	GSTA1
Alisol C	RXRB
Alisol C	ADAM17
Alisol C	ALB
Alisol C	SULT2B1
Alisol C	TGM3
Alisol C	CHEK1
Alisol C	NR3C1
Alisol C	F2
Alisol C	REN
Alisol C	FABP7
Alisol C	GART
Alisol C	PDPK1
Alisol C	CASP1
Alisol C	MET
Alisol C	TRAPPC3

Alisol C	HSD11B1
Alisol C	MAP2K1
Alisol C	RARA
Alisol C	BRAF
Alisol C	IL2
Alisol C	HPGDS
Alisol C	ESRRG
Alisol C	RARG
Alisol C	FECH
Alisol C	ESR2
Alisol C	PDE4D
Alisol C	RORA
Alisol C	NR1I3
Alisol C	HNMT
Alisol C	NR1H2
Alisol C	ITGAL
Alisol C	MMP12
Alisol C	SULT1E1
Alisol C	MMP13
Alisol C	SEC14L2
Alisol C	PCK1
Alisol C	TGFBR1
Alisol C	LSS
Alisol C	DHODH
Alisol C	NR1H3
Alisol C	HCK
Alisol C	AKR1C2
Alisol C	PLA2G2A
Alisol C	HDAC8
Alisol C	PGR
Alisol C	ABL1
Alisol C	TEK
Alisol C	MME
Alisol C	CTSK
Alisol C	MMP2
Alisol C	GRB2
Alisol C	DPEP1
Alisol C	MTAP
Alisol C	GSTP1
Alisol C	SYK
Alisol C	JAK2
Alisol C	THRB
Alisol C	ABO
Alisol C	CDK2
Alisol C	MAPK10
Alisol C	WAS
Alisol C	GSK3B
Alisol C	BIRC7

Alisol C	ESR1
Alisol C	CCNA2
Alisol C	CTSB
Alisol C	PPARG
Alisol C	LTA4H
Alisol C	BCAT2
Alisol C	NR1H4
Alisol C	ITK
Alisol C	PDE3B
Alisol C	F10
Alisol C	CALM1
Alisol C	CALM2
Alisol C	CALM3
Alisol C	ERRA
Alisol C	AKR1C3
Alisol C	KIF11
Alisol C	THRA
Alisol C	ZAP70
Alisol C	MMP9
Alisol C	MMP8
Alisol C	DTYMK
Alisol C	LCK
Alisol C	EPHB4
Alisol C	PIM1
Alisol C	ELANE
Alisol C	PPARA
Alisol C	PDE5A
Alisol C	ACADM
Alisol C	MDM2
Alisol C	TYMS
Alisol C	AURKA
Alisol C	ADH1C
Beta-sitosterol	RORA
Beta-sitosterol	MAP2K1
Beta-sitosterol	TTR
Beta-sitosterol	CYP2C8
Beta-sitosterol	TTPA
Beta-sitosterol	CRABP2
Beta-sitosterol	HSD17B1
Beta-sitosterol	VDR
Beta-sitosterol	RBP4
Beta-sitosterol	RARG
Beta-sitosterol	MAOB
Beta-sitosterol	CA2
Beta-sitosterol	PCTP
Beta-sitosterol	ADK
Beta-sitosterol	RXRA
Beta-sitosterol	GSTA1

Beta-sitosterol	MAPK14
Beta-sitosterol	MAPK10
Beta-sitosterol	PDPK1
Beta-sitosterol	FABP6
Beta-sitosterol	PPP1CC
Beta-sitosterol	PRKACA
Beta-sitosterol	NR3C1
Beta-sitosterol	RARB
Beta-sitosterol	HNMT
Beta-sitosterol	LSS
Beta-sitosterol	ALB
Beta-sitosterol	FGFR1
Beta-sitosterol	RXRB
Beta-sitosterol	HDAC8
Beta-sitosterol	HPGDS
Beta-sitosterol	SULT2A1
Beta-sitosterol	FKBP1A
Beta-sitosterol	BACE1
Beta-sitosterol	PROCR
Beta-sitosterol	NR1I2
Beta-sitosterol	KDR
Beta-sitosterol	ITGAL
Beta-sitosterol	MAPK1
Beta-sitosterol	FECH
Beta-sitosterol	DHODH
Beta-sitosterol	SULT2B1
Beta-sitosterol	METAP2
Beta-sitosterol	IL2
Beta-sitosterol	HSD11B1
Beta-sitosterol	CASP3
Beta-sitosterol	WAS
Beta-sitosterol	TEK
Beta-sitosterol	MMP2
Beta-sitosterol	MMP3
Beta-sitosterol	GSTP1
Beta-sitosterol	TRAPPC3
Beta-sitosterol	PDE4D
Beta-sitosterol	PTPN1
Beta-sitosterol	BRAF
Beta-sitosterol	GC
Beta-sitosterol	MMP13
Beta-sitosterol	ESR2
Beta-sitosterol	CALM1
Beta-sitosterol	CALM2
Beta-sitosterol	CALM3
Beta-sitosterol	NR1I3
Beta-sitosterol	FABP3
Beta-sitosterol	AR

Beta-sitosterol	FABP7
Beta-sitosterol	KIF11
Beta-sitosterol	AKR1C3
Beta-sitosterol	SHBG
Beta-sitosterol	MMP12
Beta-sitosterol	CDK2
Beta-sitosterol	NR1H4
Beta-sitosterol	F2
Beta-sitosterol	NR1H2
Beta-sitosterol	MDM2
Beta-sitosterol	THRB
Beta-sitosterol	SEC14L2
Beta-sitosterol	PPARG
Beta-sitosterol	PPARD
Beta-sitosterol	ADH
Beta-sitosterol	AKR1B1
Beta-sitosterol	HMGCR
Beta-sitosterol	HNF4G
Beta-sitosterol	LCK
Beta-sitosterol	CHEK1
Beta-sitosterol	RARA
Beta-sitosterol	DPP4
Beta-sitosterol	PDE4B
Beta-sitosterol	ADAM17
Beta-sitosterol	SYK
Beta-sitosterol	GRB2
Beta-sitosterol	PLA2G2A
Beta-sitosterol	CCNA2
Beta-sitosterol	BLVRB
Beta-sitosterol	PTPN11
Beta-sitosterol	REN
Beta-sitosterol	S100A9
Beta-sitosterol	AKR1C2
Beta-sitosterol	HSP90AA1
Beta-sitosterol	ABO
Beta-sitosterol	TGM3
Beta-sitosterol	NR1H3
Beta-sitosterol	TGFBR1
Beta-sitosterol	SULT1E1
Beta-sitosterol	HSP90AB1
Beta-sitosterol	F10
Beta-sitosterol	MET
Beta-sitosterol	GART
Beta-sitosterol	ERRA
Beta-sitosterol	ESR1
Beta-sitosterol	PDE5A
Beta-sitosterol	ABL1
Beta-sitosterol	DPEP1

Beta-sitosterol	EGFR
Beta-sitosterol	PGR
Beta-sitosterol	NQO1
Beta-sitosterol	EPHB4
Beta-sitosterol	NR3C2
Beta-sitosterol	MMP8
Beta-sitosterol	CTSK
Beta-sitosterol	GLO1
Beta-sitosterol	TGFB2
Beta-sitosterol	JAK3
Beta-sitosterol	AKR1C1
Beta-sitosterol	ESRRG
Beta-sitosterol	FNTA
Beta-sitosterol	CASP1
Beta-sitosterol	KIT
Beta-sitosterol	BCAT2
Campesterol	RORA
Campesterol	RBP4
Campesterol	TTR
Campesterol	MAOB
Campesterol	TTPA
Campesterol	CRABP2
Campesterol	MAP2K1
Campesterol	HSD17B1
Campesterol	NR3C2
Campesterol	PPP1CC
Campesterol	VDR
Campesterol	ALB
Campesterol	GSTA1
Campesterol	SULT2B1
Campesterol	GRB2
Campesterol	RARG
Campesterol	SHBG
Campesterol	MAPK14
Campesterol	RARB
Campesterol	HNF4G
Campesterol	SEC14L2
Campesterol	HDAC8
Campesterol	THRB
Campesterol	PPARD
Campesterol	FABP6
Campesterol	FECH
Campesterol	RXRB
Campesterol	GSTP1
Campesterol	RARA
Campesterol	METAP2
Campesterol	NR1H3
Campesterol	CA2

Campesterol	HSD11B1
Campesterol	RXRA
Campesterol	MAPK10
Campesterol	EGFR
Campesterol	HPGDS
Campesterol	PPARG
Campesterol	TRAPPC3
Campesterol	NR1I2
Campesterol	PCTP
Campesterol	AR
Campesterol	CASP3
Campesterol	NR1H2
Campesterol	ADK
Campesterol	F2
Campesterol	KDR
Campesterol	GLO1
Campesterol	PROCR
Campesterol	LSS
Campesterol	NR1H4
Campesterol	MMP12
Campesterol	PGR
Campesterol	HSP90AA1
Campesterol	MAPK1
Campesterol	FABP7
Campesterol	CDK2
Campesterol	DPP4
Campesterol	AKR1B1
Campesterol	MMP3
Campesterol	HNMT
Campesterol	ESR2
Campesterol	FABP3
Campesterol	HMGCR
Campesterol	CYP2C8
Campesterol	DHODH
Campesterol	NR3C1
Campesterol	PIK3R1
Campesterol	ABL1
Campesterol	SULT2A1
Campesterol	MET
Campesterol	BACE1
Campesterol	ADH
Campesterol	TEK
Campesterol	CHEK1
Campesterol	MMP13
Campesterol	CALM1
Campesterol	CALM2
Campesterol	CALM3
Campesterol	DPEP1

Campesterol	CYP2C9
Campesterol	PTPN1
Campesterol	PTPN11
Campesterol	IL2
Campesterol	EPHB4
Campesterol	LCK
Campesterol	GART
Campesterol	ADAM17
Campesterol	MMP2
Campesterol	ZAP70
Campesterol	PDE4B
Campesterol	KIF11
Campesterol	PLA2G2A
Campesterol	GSK3B
Campesterol	SYK
Campesterol	PDPK1
Campesterol	REN
Campesterol	PRKCQ
Campesterol	PRKACA
Campesterol	MAPKAPK2
Campesterol	ITK
Campesterol	ERRA
Campesterol	ESR1
Campesterol	F10
Campesterol	FGFR1
Campesterol	GC
Campesterol	SERPINA1
Campesterol	HSP90AB1
Campesterol	SULT1E1
Campesterol	PPARA
Campesterol	KIT
Campesterol	JAK3
Campesterol	PDE4D
Campesterol	ERBB4
Campesterol	LCN2
Campesterol	BRAF
Campesterol	NR1I3
Campesterol	GM2A
Campesterol	ITGAL
Campesterol	FNTA
Campesterol	NQO1
Campesterol	MDM2
Campesterol	WAS
Campesterol	AKR1C1
Campesterol	FKBP1A
Campesterol	CTSB
Campesterol	CCNA2
Campesterol	BLVRB

Campesterol	TGFBR1
Catalpol	TGM3
Catalpol	HRAS
Catalpol	UCK2
Catalpol	DCK
Catalpol	HSP90AA1
Catalpol	ZAP70
Catalpol	GCK
Catalpol	JAK2
Catalpol	HMGCR
Catalpol	EEA1
Catalpol	HSD17B1
Catalpol	DHODH
Catalpol	ADAM33
Catalpol	CDK2
Catalpol	FECH
Catalpol	GSTP1
Catalpol	ELANE
Catalpol	PDPK1
Catalpol	MTAP
Catalpol	FKBP1A
Catalpol	FKBP1B
Catalpol	HNMT
Catalpol	MMP3
Catalpol	CDK5R1
Catalpol	MAPKAPK2
Catalpol	GSTM2
Catalpol	CTSK
Catalpol	ABO
Catalpol	PDE4B
Catalpol	F2
Catalpol	CTSB
Catalpol	GSK3B
Catalpol	NR3C1
Catalpol	AR
Catalpol	VDR
Catalpol	UAP1
Catalpol	BCAT2
Catalpol	PNMT
Catalpol	AKR1B1
Catalpol	PDE4D
Catalpol	S100A9
Catalpol	BACE1
Catalpol	SRC
Catalpol	LSS
Catalpol	RARG
Catalpol	MME
Catalpol	RFK

Catalpol	GART
Catalpol	DAPK1
Catalpol	IGF1R
Catalpol	AKR1C3
Catalpol	AURKA
Catalpol	METAP2
Catalpol	NR1H4
Catalpol	SHBG
Catalpol	PTPN1
Catalpol	RAP2A
Catalpol	LTA4H
Catalpol	CCNA2
Catalpol	FKBP3
Catalpol	KAT2B
Catalpol	PIM1
Catalpol	AMD1
Catalpol	DUT
Catalpol	RAC1
Catalpol	GSTA1
Catalpol	REN
Catalpol	CASP3
Catalpol	PPP1CC
Catalpol	KIF11
Catalpol	AKT1
Catalpol	NQO1
Catalpol	FOLH1
Catalpol	PRKACA
Catalpol	SORD
Catalpol	CHEK1
Catalpol	ARHGAP1
Catalpol	CTSS
Catalpol	CYP2C9
Catalpol	MAPK14
Catalpol	TPSB2
Catalpol	ALB
Catalpol	CASP1
Catalpol	HSD11B1
Catalpol	CA2
Catalpol	CTNNA1
Catalpol	PAH
Catalpol	PCK1
Catalpol	TYMS
Catalpol	GSTM1
Catalpol	CBR1
Catalpol	LCK
Catalpol	OAT
Catalpol	ITK
Catalpol	HSP90AB1

Catalpol	MAP2K1
Catalpol	EGFR
Catalpol	SULT1A1
Catalpol	PIK3R1
Catalpol	FGFR1
Catalpol	KDR
Catalpol	NOS2
Catalpol	ABL1
Catalpol	HDAC8
Catalpol	BTK
Catalpol	JAK3
Catalpol	OTC
Catalpol	SDS
Catalpol	TPH1
Catalpol	HCK
Catalpol	PADI4
Catalpol	DTYMK
Catalpol	F11
Catalpol	EIF4E
Catalpol	GLO1
Catalpol	ADH
Catalpol	GP1BA
Catalpol	PDE5A
Catalpol	SELE
Cerevisterol	RORA
Cerevisterol	TTR
Cerevisterol	TTPA
Cerevisterol	HSD17B1
Cerevisterol	CRABP2
Cerevisterol	RBP4
Cerevisterol	MAP2K1
Cerevisterol	TRAPPC3
Cerevisterol	RXRB
Cerevisterol	FECH
Cerevisterol	VDR
Cerevisterol	RARB
Cerevisterol	FABP7
Cerevisterol	CYP2C8
Cerevisterol	DPP4
Cerevisterol	PROCR
Cerevisterol	FABP6
Cerevisterol	MAOB
Cerevisterol	METAP2
Cerevisterol	EPHB4
Cerevisterol	PPP1CC
Cerevisterol	SULT2B1
Cerevisterol	PDPK1
Cerevisterol	HPGDS

Cerevisterol	CA2
Cerevisterol	HNMT
Cerevisterol	PLA2G2A
Cerevisterol	PTPN1
Cerevisterol	ALB
Cerevisterol	ESR2
Cerevisterol	MAPK10
Cerevisterol	THRB
Cerevisterol	NR1H2
Cerevisterol	F2
Cerevisterol	RARG
Cerevisterol	RARA
Cerevisterol	MAPK14
Cerevisterol	PRKACA
Cerevisterol	AKR1B1
Cerevisterol	MET
Cerevisterol	GSTA1
Cerevisterol	FABP3
Cerevisterol	SHBG
Cerevisterol	TEK
Cerevisterol	RXRA
Cerevisterol	NR3C1
Cerevisterol	BACE1
Cerevisterol	HNF4G
Cerevisterol	MAPK1
Cerevisterol	MMP12
Cerevisterol	DHODH
Cerevisterol	LCK
Cerevisterol	NR1H4
Cerevisterol	PDE4D
Cerevisterol	LSS
Cerevisterol	MMP13
Cerevisterol	AR
Cerevisterol	SEC14L2
Cerevisterol	GSTP1
Cerevisterol	GSTT2
Cerevisterol	GRB2
Cerevisterol	SULT2A1
Cerevisterol	PPARD
Cerevisterol	AKR1C2
Cerevisterol	ADH
Cerevisterol	PTPN11
Cerevisterol	HSD11B1
Cerevisterol	PGR
Cerevisterol	KIF11
Cerevisterol	SULT1E1
Cerevisterol	F10
Cerevisterol	HDAC8

Cerevisterol	ELANE
Cerevisterol	HSP90AA1
Cerevisterol	KDR
Cerevisterol	IL2
Cerevisterol	ADAM17
Cerevisterol	GSK3B
Cerevisterol	MMP3
Cerevisterol	MMP2
Cerevisterol	HMGCR
Cerevisterol	CHEK1
Cerevisterol	CASP3
Cerevisterol	ADK
Cerevisterol	MDM2
Cerevisterol	DPEP1
Cerevisterol	CALM1
Cerevisterol	CALM2
Cerevisterol	CALM3
Cerevisterol	ERBB4
Cerevisterol	PCTP
Cerevisterol	ESR1
Cerevisterol	CDK2
Cerevisterol	FGFR1
Cerevisterol	LTA4H
Cerevisterol	ABL1
Cerevisterol	FKBP1A
Cerevisterol	CTSK
Cerevisterol	SRC
Cerevisterol	PIM1
Cerevisterol	REN
Cerevisterol	BLVRB
Cerevisterol	WAS
Cerevisterol	ABO
Cerevisterol	NR1I2
Cerevisterol	HSP90AB1
Cerevisterol	TGFBR1
Cerevisterol	NR1H3
Cerevisterol	GSR
Cerevisterol	PPARA
Cerevisterol	SERPINA1
Cerevisterol	PDE4B
Cerevisterol	BRAF
Cerevisterol	PPARG
Cerevisterol	KIT
Cerevisterol	MTAP
Cerevisterol	MAOA
Cerevisterol	CCNA2
Cerevisterol	EGFR
Cerevisterol	AKR1C3

Cerevisterol	NR3C2
Cerevisterol	NQO1
Cerevisterol	CASP1
Cerevisterol	SYK
Cerevisterol	GC
Cerevisterol	ZAP70
Cerevisterol	MAPKAPK2
CLR	RORA
CLR	TTR
CLR	RBP4
CLR	MAOB
CLR	CRABP2
CLR	TTPA
CLR	HSD17B1
CLR	PPP1CC
CLR	HPGDS
CLR	NR3C2
CLR	FGFR1
CLR	MAP2K1
CLR	RARG
CLR	ALB
CLR	VDR
CLR	SEC14L2
CLR	SULT2B1
CLR	MAPK14
CLR	SHBG
CLR	HSD11B1
CLR	AKR1B1
CLR	FKBP1A
CLR	FABP6
CLR	RXRB
CLR	FECH
CLR	TRAPPC3
CLR	CYP2C8
CLR	LSS
CLR	AKR1C2
CLR	RXRA
CLR	MMP12
CLR	NR3C1
CLR	CHEK1
CLR	PCTP
CLR	CA2
CLR	NR1H4
CLR	HNF4G
CLR	FABP7
CLR	PPARG
CLR	THRB
CLR	RARB

CLR	MMP3
CLR	CDK2
CLR	PRKACA
CLR	CASP3
CLR	GSTA1
CLR	HNMT
CLR	PPARD
CLR	ESR2
CLR	HSP90AA1
CLR	FABP3
CLR	F2
CLR	ADH
CLR	IL2
CLR	MAPK10
CLR	PROCR
CLR	TEK
CLR	METAP2
CLR	GRB2
CLR	MAPK1
CLR	MTAP
CLR	RARA
CLR	DHODH
CLR	GC
CLR	ABL1
CLR	PTPN1
CLR	PTPN11
CLR	HDAC8
CLR	MET
CLR	NR1H2
CLR	AR
CLR	GLO1
CLR	DPEP1
CLR	PGR
CLR	ADK
CLR	NR1I2
CLR	NR1H3
CLR	ABO
CLR	HMGCR
CLR	TGM3
CLR	ERRA
CLR	SULT2A1
CLR	SULT1E1
CLR	KIF11
CLR	CASP1
CLR	PDE4D
CLR	BACE1
CLR	EPHB4
CLR	ITGAL

CLR	CALM1
CLR	CALM2
CLR	CALM3
CLR	SERPINA1
CLR	PDE4B
CLR	REN
CLR	PDPK1
CLR	GSTP1
CLR	F10
CLR	WAS
CLR	ZAP70
CLR	MMP2
CLR	LCK
CLR	GSK3B
CLR	MDM2
CLR	ERBB4
CLR	CCNA2
CLR	KDR
CLR	DPP4
CLR	HSP90AB1
CLR	PRKCQ
CLR	MAPKAPK2
CLR	NR1I3
CLR	LCN2
CLR	JAK3
CLR	MMP13
CLR	ESR1
CLR	CYP2C9
CLR	GM2A
CLR	ITK
CLR	ADAM17
CLR	PLA2G2A
CLR	TGFBR1
CLR	PPARA
CLR	S100A9
CLR	BLVRB
CLR	PIK3R1
Cornudentanone	CRABP2
Cornudentanone	TTR
Cornudentanone	MAOB
Cornudentanone	RBP4
Cornudentanone	MAPK14
Cornudentanone	FKBP1A
Cornudentanone	LSS
Cornudentanone	RXRA
Cornudentanone	AKR1C3
Cornudentanone	MMP3
Cornudentanone	ADH

Cornudentanone	TTPA
Cornudentanone	CA2
Cornudentanone	VDR
Cornudentanone	FABP3
Cornudentanone	RXRB
Cornudentanone	BRAF
Cornudentanone	RARB
Cornudentanone	AKR1B1
Cornudentanone	PTPN1
Cornudentanone	SULT2B1
Cornudentanone	BACE1
Cornudentanone	METAP2
Cornudentanone	ESR1
Cornudentanone	HSD17B1
Cornudentanone	AR
Cornudentanone	HPGDS
Cornudentanone	MAPK10
Cornudentanone	MET
Cornudentanone	NR1I2
Cornudentanone	ALB
Cornudentanone	FGFR1
Cornudentanone	HSP90AA1
Cornudentanone	APAF1
Cornudentanone	MAP2K1
Cornudentanone	CASP1
Cornudentanone	NR3C1
Cornudentanone	GSTT2
Cornudentanone	CHEK1
Cornudentanone	PPP1CC
Cornudentanone	HNF4G
Cornudentanone	PDE4D
Cornudentanone	AKR1C2
Cornudentanone	RARG
Cornudentanone	CCNA2
Cornudentanone	FGG
Cornudentanone	LCK
Cornudentanone	HSD11B1
Cornudentanone	RORA
Cornudentanone	MME
Cornudentanone	HSP90AB1
Cornudentanone	GSTA1
Cornudentanone	THRB
Cornudentanone	CDK2
Cornudentanone	TRAPPC3
Cornudentanone	ABO
Cornudentanone	SULT2A1
Cornudentanone	PLA2G2A
Cornudentanone	CTSK

Cornudentanone	RARA
Cornudentanone	HNMT
Cornudentanone	HMGCR
Cornudentanone	REN
Cornudentanone	PDPK1
Cornudentanone	FABP6
Cornudentanone	F2
Cornudentanone	TGM3
Cornudentanone	KIF11
Cornudentanone	PROCR
Cornudentanone	MMP12
Cornudentanone	ELANE
Cornudentanone	ITK
Cornudentanone	PCTP
Cornudentanone	BHMT
Cornudentanone	PGR
Cornudentanone	GSTP1
Cornudentanone	ITGAL
Cornudentanone	EPHB4
Cornudentanone	OAT
Cornudentanone	DPEP1
Cornudentanone	SHBG
Cornudentanone	CYP2C8
Cornudentanone	PIM1
Cornudentanone	FECH
Cornudentanone	PRKCQ
Cornudentanone	HCK
Cornudentanone	DHODH
Cornudentanone	INSR
Cornudentanone	PPARA
Cornudentanone	MTAP
Cornudentanone	ESRRG
Cornudentanone	SRC
Cornudentanone	PRKACA
Cornudentanone	BTK
Cornudentanone	RFK
Cornudentanone	SEC14L2
Cornudentanone	JAK2
Cornudentanone	ADK
Cornudentanone	FABP7
Cornudentanone	EGFR
Cornudentanone	TEK
Cornudentanone	PTPN11
Cornudentanone	DCK
Cornudentanone	GSK3B
Cornudentanone	KDR
Cornudentanone	GM2A
Cornudentanone	TGFBR1

Cornudentanone	GLO1
Cornudentanone	PDE4B
Cornudentanone	IL2
Cornudentanone	MAPK1
Cornudentanone	ADAM17
Cornudentanone	THRA
Cornudentanone	BIRC7
Cornudentanone	ESR2
Cornudentanone	PCK1
Cornudentanone	TGFB2
Cornudentanone	BLVRB
Cornudentanone	SERPINA1
Cornudentanone	BST1
Cornudentanone	KIT
Cornudentanone	NR1H2
Cornudentanone	DPP4
Cornudentanone	MMP13
Cornudentanone	UCK2
Cornudentanone	PPARD
Cornudentanone	GRB2
Cornudentanone	HDAC8
Cornudentanone	MDM2
Cornudentanone	GC
Cornudentanone	DTYMK
Dehydroeburicoic acid	TTR
Dehydroeburicoic acid	RBP4
Dehydroeburicoic acid	RORA
Dehydroeburicoic acid	CRABP2
Dehydroeburicoic acid	HSD17B1
Dehydroeburicoic acid	MAP2K1
Dehydroeburicoic acid	PPP1CC
Dehydroeburicoic acid	PTPN1
Dehydroeburicoic acid	TTPA
Dehydroeburicoic acid	RARB
Dehydroeburicoic acid	F2
Dehydroeburicoic acid	MAOB
Dehydroeburicoic acid	GART
Dehydroeburicoic acid	DPEP1
Dehydroeburicoic acid	MET
Dehydroeburicoic acid	VDR
Dehydroeburicoic acid	GSTA1
Dehydroeburicoic acid	HSD11B1
Dehydroeburicoic acid	CDK2
Dehydroeburicoic acid	AKR1B1
Dehydroeburicoic acid	ESR2
Dehydroeburicoic acid	MMP3
Dehydroeburicoic acid	RARG
Dehydroeburicoic acid	FECH

Dehydroeburicoic aci	CYP2C8
Dehydroeburicoic aci	AKR1C2
Dehydroeburicoic aci	LSS
Dehydroeburicoic aci	METAP2
Dehydroeburicoic aci	LTA4H
Dehydroeburicoic aci	AKR1C3
Dehydroeburicoic aci	RXRB
Dehydroeburicoic aci	MAPK1
Dehydroeburicoic aci	MAPK10
Dehydroeburicoic aci	BACE1
Dehydroeburicoic aci	CASP3
Dehydroeburicoic aci	MAPK14
Dehydroeburicoic aci	AR
Dehydroeburicoic aci	PRKACA
Dehydroeburicoic aci	CA2
Dehydroeburicoic aci	SHBG
Dehydroeburicoic aci	THRB
Dehydroeburicoic aci	ALB
Dehydroeburicoic aci	DHODH
Dehydroeburicoic aci	LCK
Dehydroeburicoic aci	SULT2B1
Dehydroeburicoic aci	HNF4G
Dehydroeburicoic aci	PROCR
Dehydroeburicoic aci	HSP90AA1
Dehydroeburicoic aci	FABP6
Dehydroeburicoic aci	PDE4B
Dehydroeburicoic aci	ABL1
Dehydroeburicoic aci	ABO
Dehydroeburicoic aci	CHEK1
Dehydroeburicoic aci	PDE4D
Dehydroeburicoic aci	HPGDS
Dehydroeburicoic aci	ADH
Dehydroeburicoic aci	PDPK1
Dehydroeburicoic aci	NR1I2
Dehydroeburicoic aci	PCTP
Dehydroeburicoic aci	NR3C1
Dehydroeburicoic aci	FABP7
Dehydroeburicoic aci	NR3C2
Dehydroeburicoic aci	NR1H2
Dehydroeburicoic aci	RARA
Dehydroeburicoic aci	IL2
Dehydroeburicoic aci	SULT2A1
Dehydroeburicoic aci	HNMT
Dehydroeburicoic aci	HDAC8
Dehydroeburicoic aci	ADAM17
Dehydroeburicoic aci	GSTT2
Dehydroeburicoic aci	NR1H4
Dehydroeburicoic aci	FABP3

Dehydroeburicoic acid	REN
Dehydroeburicoic acid	CALM1
Dehydroeburicoic acid	CALM2
Dehydroeburicoic acid	CALM3
Dehydroeburicoic acid	BRAF
Dehydroeburicoic acid	ADK
Dehydroeburicoic acid	TEK
Dehydroeburicoic acid	CTSB
Dehydroeburicoic acid	GRB2
Dehydroeburicoic acid	PTPN11
Dehydroeburicoic acid	PGR
Dehydroeburicoic acid	CASP1
Dehydroeburicoic acid	MMP12
Dehydroeburicoic acid	CTSS
Dehydroeburicoic acid	MMP2
Dehydroeburicoic acid	TRAPPC3
Dehydroeburicoic acid	PPARD
Dehydroeburicoic acid	EPHB4
Dehydroeburicoic acid	RXRA
Dehydroeburicoic acid	SORD
Dehydroeburicoic acid	ESR1
Dehydroeburicoic acid	GSTP1
Dehydroeburicoic acid	BCAT2
Dehydroeburicoic acid	NR1H3
Dehydroeburicoic acid	KDR
Dehydroeburicoic acid	HCK
Dehydroeburicoic acid	OAT
Dehydroeburicoic acid	MME
Dehydroeburicoic acid	FKBP1A
Dehydroeburicoic acid	TYMS
Dehydroeburicoic acid	PLA2G2A
Dehydroeburicoic acid	GSK3B
Dehydroeburicoic acid	SRC
Dehydroeburicoic acid	MTAP
Dehydroeburicoic acid	ANXA5
Dehydroeburicoic acid	BCL2L1
Dehydroeburicoic acid	THRA
Dehydroeburicoic acid	MTHFD1
Dehydroeburicoic acid	BIRC7
Dehydroeburicoic acid	SEC14L2
Dehydroeburicoic acid	PPARG
Dehydroeburicoic acid	GLO1
Dehydroeburicoic acid	MMP8
Dehydroeburicoic acid	MDM2
Dehydroeburicoic acid	DPP4
Dehydroeburicoic acid	ERBB4
Dehydroeburicoic acid	TGM3
Dehydroeburicoic acid	ZAP70

Dehydroeburicoic acid	CYP2C9
Dehydroeburicoic acid	BLVRB
Dehydroeburicoic acid	IGF1R
Dehydroeburicoic acid	PRKCQ
Dehydroeburicoic acid	TAP1
Dehydroeburicoic acid	SULT1E1
Dehydroeburicoic acid	KIT
Dehydroeburicoic acid	WAS
Denudatin B	PDPK1
Denudatin B	CRABP2
Denudatin B	THRA
Denudatin B	FGFR1
Denudatin B	CA2
Denudatin B	MAOB
Denudatin B	FKBP1A
Denudatin B	GSTA3
Denudatin B	ELANE
Denudatin B	PPP1CC
Denudatin B	TTR
Denudatin B	METAP2
Denudatin B	AKR1B1
Denudatin B	RARA
Denudatin B	PDE4D
Denudatin B	AKR1C3
Denudatin B	MAPK10
Denudatin B	GSTA1
Denudatin B	RARG
Denudatin B	LTA4H
Denudatin B	PLA2G2A
Denudatin B	RARB
Denudatin B	PTPN1
Denudatin B	ADH
Denudatin B	DHODH
Denudatin B	LCK
Denudatin B	F2
Denudatin B	RORA
Denudatin B	HSD17B1
Denudatin B	VDR
Denudatin B	CDK2
Denudatin B	OTC
Denudatin B	NT5M
Denudatin B	SRC
Denudatin B	RBP4
Denudatin B	MAPK14
Denudatin B	THRB
Denudatin B	BACE1
Denudatin B	SULT2B1
Denudatin B	INSR

Denudatin B	HMGCR
Denudatin B	HSP90AA1
Denudatin B	CHEK1
Denudatin B	HPGDS
Denudatin B	REN
Denudatin B	MMP3
Denudatin B	PPARA
Denudatin B	SORD
Denudatin B	SHBG
Denudatin B	RXRB
Denudatin B	NR1I2
Denudatin B	EGFR
Denudatin B	KDR
Denudatin B	GSTM2
Denudatin B	ABO
Denudatin B	NR1H3
Denudatin B	MAP2K1
Denudatin B	TGFBR1
Denudatin B	HDAC8
Denudatin B	DPEP1
Denudatin B	PROCR
Denudatin B	PDE5A
Denudatin B	HSD11B1
Denudatin B	PRKACA
Denudatin B	AR
Denudatin B	HNMT
Denudatin B	BRAF
Denudatin B	GLO1
Denudatin B	ALB
Denudatin B	ESR1
Denudatin B	ESRRG
Denudatin B	OAT
Denudatin B	MMP12
Denudatin B	ACE
Denudatin B	HRAS
Denudatin B	MAPK1
Denudatin B	GSK3B
Denudatin B	FABP6
Denudatin B	SYK
Denudatin B	KIF11
Denudatin B	NR3C2
Denudatin B	GSTM1
Denudatin B	CASP3
Denudatin B	TTPA
Denudatin B	CTSK
Denudatin B	FECH
Denudatin B	PPARG
Denudatin B	ESR2

Denudatin B	KIT
Denudatin B	GSTP1
Denudatin B	NQO1
Denudatin B	SULT1E1
Denudatin B	EPHB4
Denudatin B	PNMT
Denudatin B	CCNA2
Denudatin B	GSTZ1
Denudatin B	MET
Denudatin B	ADAM17
Denudatin B	RXRA
Denudatin B	TEK
Denudatin B	BIRC7
Denudatin B	PDE4B
Denudatin B	CBR1
Denudatin B	CYP2C9
Denudatin B	HNF4G
Denudatin B	TRAPPC3
Denudatin B	MMP8
Denudatin B	PTPN11
Denudatin B	RFK
Denudatin B	SEC14L2
Denudatin B	CALM1
Denudatin B	CALM2
Denudatin B	CALM3
Denudatin B	PCK1
Denudatin B	LCN2
Denudatin B	PIK3CG
Denudatin B	CASP1
Denudatin B	MMP13
Denudatin B	MMP2
Denudatin B	DPP4
Denudatin B	HCK
Denudatin B	DUT
Denudatin B	RAP2A
Denudatin B	BLVRB
Denudatin B	CYP2C8
Denudatin B	CTSF
Denudatin B	IL2
Denudatin B	AMY2A
Denudatin B	PGR
Denudatin B	PCTP
Denudatin B	NR1H4
Diop	AKR1B1
Diop	MAPK10
Diop	METAP2
Diop	F2
Diop	LTA4H

Diop	VDR
Diop	MET
Diop	CRABP2
Diop	MMP12
Diop	FKBP1A
Diop	RARG
Diop	TTPA
Diop	BACE1
Diop	PPP1CC
Diop	MMP3
Diop	MAPK14
Diop	DHODH
Diop	MAOB
Diop	TGM3
Diop	TTR
Diop	RBP4
Diop	RXRB
Diop	PROCR
Diop	TGFB2
Diop	PCTP
Diop	CASP1
Diop	GLO1
Diop	HSP90AA1
Diop	PDPK1
Diop	HMGCR
Diop	ALB
Diop	DPP4
Diop	AR
Diop	MME
Diop	HSD11B1
Diop	PLA2G2A
Diop	CA2
Diop	RORA
Diop	HNMT
Diop	HDAC8
Diop	F10
Diop	KIF11
Diop	BCAT2
Diop	FABP3
Diop	RARB
Diop	MMP13
Diop	MAP2K1
Diop	ESR2
Diop	PPARD
Diop	TEK
Diop	PTPN1
Diop	KDR
Diop	FGFR1

Diop	PARP1
Diop	NR1I2
Diop	FABP7
Diop	AKR1C3
Diop	MMP8
Diop	NR3C1
Diop	NR1H2
Diop	ELANE
Diop	WAS
Diop	RXRA
Diop	AKR1C2
Diop	ABL1
Diop	PIM1
Diop	LSS
Diop	BRAF
Diop	THRB
Diop	PCK1
Diop	SEC14L2
Diop	PPARG
Diop	DPEP1
Diop	ABO
Diop	ADAM17
Diop	IL2
Diop	NR1H4
Diop	CDK2
Diop	BLVRB
Diop	RFK
Diop	EGFR
Diop	GM2A
Diop	SHBG
Diop	GSTP1
Diop	MMP2
Diop	HCK
Diop	LCK
Diop	GSTA1
Diop	EPHX2
Diop	KIT
Diop	CASP3
Diop	TRAPPC3
Diop	PPARA
Diop	CTSB
Diop	PDE4D
Diop	GSK3B
Diop	SORD
Diop	PDE4B
Diop	XIAP
Diop	ADAM33
Diop	REN

Diop	RARA
Diop	PYGL
Diop	CYP2C8
Diop	HSD17B1
Diop	DUT
Diop	HNF4G
Diop	PRKACA
Diop	SERPINA1
Diop	INSR
Diop	MAPK1
Diop	CTSK
Diop	AMD1
Diop	SULT2A1
Diop	FECH
Diop	CTSS
Diop	DCPS
Diop	ADK
Diop	TGFBR1
Diop	BHMT
Dioscoreside C	TTR
Dioscoreside C	TTPA
Dioscoreside C	HSD17B1
Dioscoreside C	RBP4
Dioscoreside C	VDR
Dioscoreside C	CRABP2
Dioscoreside C	MAOB
Dioscoreside C	RARB
Dioscoreside C	MET
Dioscoreside C	RXRA
Dioscoreside C	RORA
Dioscoreside C	TRAPPC3
Dioscoreside C	CA2
Dioscoreside C	AR
Dioscoreside C	NR3C2
Dioscoreside C	LSS
Dioscoreside C	HSP90AA1
Dioscoreside C	NR1I2
Dioscoreside C	PTPN1
Dioscoreside C	AKR1C2
Dioscoreside C	METAP2
Dioscoreside C	NR3C1
Dioscoreside C	ELANE
Dioscoreside C	SEC14L2
Dioscoreside C	FABP6
Dioscoreside C	SULT2B1
Dioscoreside C	MAP2K1
Dioscoreside C	PPP1CC
Dioscoreside C	F2

Dioscoreside C	HPGDS
Dioscoreside C	HSD11B1
Dioscoreside C	KDR
Dioscoreside C	MAPK14
Dioscoreside C	PDE4D
Dioscoreside C	RARG
Dioscoreside C	DHODH
Dioscoreside C	SULT2A1
Dioscoreside C	SHBG
Dioscoreside C	ABO
Dioscoreside C	PRKACA
Dioscoreside C	FKBP1A
Dioscoreside C	RXRB
Dioscoreside C	MMP3
Dioscoreside C	BACE1
Dioscoreside C	PCTP
Dioscoreside C	RARA
Dioscoreside C	FECH
Dioscoreside C	FABP7
Dioscoreside C	FABP3
Dioscoreside C	ESR2
Dioscoreside C	LCK
Dioscoreside C	THRB
Dioscoreside C	CYP2C8
Dioscoreside C	AKR1B1
Dioscoreside C	NR1H3
Dioscoreside C	GSTP1
Dioscoreside C	REN
Dioscoreside C	PPARG
Dioscoreside C	TGM3
Dioscoreside C	CHEK1
Dioscoreside C	KIT
Dioscoreside C	IL2
Dioscoreside C	GSK3B
Dioscoreside C	GSTA1
Dioscoreside C	ALB
Dioscoreside C	CDK2
Dioscoreside C	FGFR1
Dioscoreside C	HDAC8
Dioscoreside C	MMP12
Dioscoreside C	CCNA2
Dioscoreside C	MTHFD1
Dioscoreside C	EPHB4
Dioscoreside C	GSTT2
Dioscoreside C	F10
Dioscoreside C	NQO1
Dioscoreside C	BRAF
Dioscoreside C	CALM1

Dioscoreside C	CALM2
Dioscoreside C	CALM3
Dioscoreside C	DPEP1
Dioscoreside C	EGFR
Dioscoreside C	HNMT
Dioscoreside C	NR1H4
Dioscoreside C	CASP1
Dioscoreside C	PLA2G2A
Dioscoreside C	MME
Dioscoreside C	ESR1
Dioscoreside C	TEK
Dioscoreside C	PDE4B
Dioscoreside C	GCK
Dioscoreside C	MTAP
Dioscoreside C	PIM1
Dioscoreside C	AKR1C3
Dioscoreside C	ADH
Dioscoreside C	MAPKAPK2
Dioscoreside C	CASP3
Dioscoreside C	PROCR
Dioscoreside C	DHFR
Dioscoreside C	ZAP70
Dioscoreside C	CTSK
Dioscoreside C	SYK
Dioscoreside C	ABL1
Dioscoreside C	PDPK1
Dioscoreside C	PGR
Dioscoreside C	PRKCQ
Dioscoreside C	PPARA
Dioscoreside C	ADK
Dioscoreside C	JAK2
Dioscoreside C	PPARD
Dioscoreside C	MMP2
Dioscoreside C	MAPK1
Dioscoreside C	CBR1
Dioscoreside C	MAOA
Dioscoreside C	ERBB4
Dioscoreside C	MAPK10
Dioscoreside C	HNF4G
Dioscoreside C	MMP8
Dioscoreside C	JAK3
Dioscoreside C	FNTA
Dioscoreside C	ERRA
Dioscoreside C	ADH1C
Dioscoreside C	ITK
Dioscoreside C	THRA
Dioscoreside C	NR1H2
Dioscoreside C	CES1

Dioscoreside C	MMP13
Dioscoreside C	GRB2
Dioscoreside C	SERPINA1
Dioscoreside C	HMGCR
Dioscoreside C	TGFBR1
Dioscoreside C	INSR
Dioscoreside C	DPP4
Diosgenin	TTR
Diosgenin	RBP4
Diosgenin	CRABP2
Diosgenin	HSD17B1
Diosgenin	SHBG
Diosgenin	MAP2K1
Diosgenin	MAPK14
Diosgenin	NR1I2
Diosgenin	TTPA
Diosgenin	RARG
Diosgenin	RXRB
Diosgenin	RXRA
Diosgenin	PCTP
Diosgenin	RORA
Diosgenin	SEC14L2
Diosgenin	CA2
Diosgenin	RARB
Diosgenin	MAOB
Diosgenin	AR
Diosgenin	ALB
Diosgenin	PPP1CC
Diosgenin	NR3C2
Diosgenin	PTPN1
Diosgenin	PPARG
Diosgenin	VDR
Diosgenin	FKBP1A
Diosgenin	NR1H2
Diosgenin	ADK
Diosgenin	SULT2A1
Diosgenin	NR1H4
Diosgenin	HDAC8
Diosgenin	ADH
Diosgenin	PROCR
Diosgenin	PDE4B
Diosgenin	BACE1
Diosgenin	PDE4D
Diosgenin	CYP2C8
Diosgenin	F2
Diosgenin	ESR1
Diosgenin	THRB
Diosgenin	REN

Diosgenin	HSD11B1
Diosgenin	HMGCR
Diosgenin	TRAPPC3
Diosgenin	DHODH
Diosgenin	FABP6
Diosgenin	MMP3
Diosgenin	BRAF
Diosgenin	CTNNA1
Diosgenin	MMP2
Diosgenin	GSTA1
Diosgenin	IL2
Diosgenin	JAK2
Diosgenin	MET
Diosgenin	MMP13
Diosgenin	HNMT
Diosgenin	SULT1E1
Diosgenin	AKR1B1
Diosgenin	GCK
Diosgenin	SULT2B1
Diosgenin	GC
Diosgenin	PDPK1
Diosgenin	FABP7
Diosgenin	PGR
Diosgenin	TGM3
Diosgenin	MMP12
Diosgenin	DPEP1
Diosgenin	NR1H3
Diosgenin	GSTP1
Diosgenin	METAP2
Diosgenin	MTHFD1
Diosgenin	ADAM17
Diosgenin	CALM1
Diosgenin	CALM2
Diosgenin	CALM3
Diosgenin	FECH
Diosgenin	HNF4G
Diosgenin	NR3C1
Diosgenin	GSK3B
Diosgenin	KDR
Diosgenin	NQO1
Diosgenin	TGFB2
Diosgenin	PRKACA
Diosgenin	HCK
Diosgenin	CHEK1
Diosgenin	ERBB4
Diosgenin	MAPK1
Diosgenin	MTAP
Diosgenin	GRB2

Diosgenin	FABP3
Diosgenin	HPGDS
Diosgenin	RARA
Diosgenin	ABO
Diosgenin	KIF11
Diosgenin	PTPN11
Diosgenin	CFD
Diosgenin	DPP4
Diosgenin	CASP1
Diosgenin	LCK
Diosgenin	HSP90AA1
Diosgenin	PPARD
Diosgenin	AKR1C3
Diosgenin	F10
Diosgenin	ESR2
Diosgenin	AKR1C1
Diosgenin	LSS
Diosgenin	TEK
Diosgenin	TGFBR1
Diosgenin	ADH1C
Diosgenin	ABL1
Diosgenin	AMD1
Diosgenin	PPARA
Diosgenin	BHMT
Diosgenin	CDK2
Diosgenin	MAPK10
Diosgenin	PNMT
Diosgenin	CASP3
Diosgenin	S100A9
Diosgenin	PIK3R1
Diosgenin	MDM2
Diosgenin	MAPKAPK2
Diosgenin	FGFR1
Diosgenin	BMP2
Diosgenin	PSAP
Diosgenin	GART
Diosgenin	BCL2L1
Diosgenin	BLVRB
Diosgenin	EPHX2
Diosgenin	PLA2G2A
Diosgenin	PIK3CG
Diosgenin	ITGAL
Diosgenin	PPP5C
Diosgenin	ELANE
Diosgenin	JAK3
Diosgenin	PRKCQ
Diosgenin	PDE3B
Diosgenin	TYMS

Doradexanthin	TTR
Doradexanthin	TTPA
Doradexanthin	CYP2C8
Doradexanthin	PPP1CC
Doradexanthin	VDR
Doradexanthin	METAP2
Doradexanthin	ALB
Doradexanthin	RXRB
Doradexanthin	FABP7
Doradexanthin	RBP4
Doradexanthin	RARB
Doradexanthin	CRABP2
Doradexanthin	FABP3
Doradexanthin	HSD11B1
Doradexanthin	MAPK14
Doradexanthin	RORA
Doradexanthin	DHODH
Doradexanthin	MAOB
Doradexanthin	HSD17B1
Doradexanthin	CASP3
Doradexanthin	NR3C1
Doradexanthin	CDK2
Doradexanthin	MDM2
Doradexanthin	REN
Doradexanthin	THRB
Doradexanthin	CYP2C9
Doradexanthin	MAPK10
Doradexanthin	MAP2K1
Doradexanthin	BACE1
Doradexanthin	PROCR
Doradexanthin	FECH
Doradexanthin	GSTP1
Doradexanthin	AKR1C2
Doradexanthin	NR1I2
Doradexanthin	RXRA
Doradexanthin	MMP12
Doradexanthin	SEC14L2
Doradexanthin	GSTA1
Doradexanthin	EGFR
Doradexanthin	AKR1C3
Doradexanthin	AR
Doradexanthin	DPP4
Doradexanthin	CASP1
Doradexanthin	CA2
Doradexanthin	IL2
Doradexanthin	ESR1
Doradexanthin	PCTP
Doradexanthin	RARG

Doradexanthin	LCK
Doradexanthin	TRAPPC3
Doradexanthin	PRKACA
Doradexanthin	F2
Doradexanthin	HNMT
Doradexanthin	HNF4G
Doradexanthin	SULT2B1
Doradexanthin	TEK
Doradexanthin	FABP6
Doradexanthin	SULT1E1
Doradexanthin	KIT
Doradexanthin	PTPN1
Doradexanthin	CTSK
Doradexanthin	KDR
Doradexanthin	CHEK1
Doradexanthin	HDAC8
Doradexanthin	NR1H2
Doradexanthin	MET
Doradexanthin	AKR1B1
Doradexanthin	FKBP1A
Doradexanthin	ESR2
Doradexanthin	SERPINA1
Doradexanthin	ADK
Doradexanthin	BRAF
Doradexanthin	PGR
Doradexanthin	PDPK1
Doradexanthin	LSS
Doradexanthin	HPGDS
Doradexanthin	NR1I3
Doradexanthin	NR1H4
Doradexanthin	NR3C2
Doradexanthin	MMP2
Doradexanthin	CCNA2
Doradexanthin	SHBG
Doradexanthin	PPARD
Doradexanthin	HMGCR
Doradexanthin	ITGAL
Doradexanthin	NR1H3
Doradexanthin	PLA2G2A
Doradexanthin	GRB2
Doradexanthin	EPHB4
Doradexanthin	KIF11
Doradexanthin	PSAP
Doradexanthin	GSTT2
Doradexanthin	MMP3
Doradexanthin	PDE4D
Doradexanthin	ABL1
Doradexanthin	GC

Doradexanthin	ADH
Doradexanthin	F10
Doradexanthin	DPEP1
Doradexanthin	SULT2A1
Doradexanthin	ADH1C
Doradexanthin	ABO
Doradexanthin	THRA
Doradexanthin	GLO1
Doradexanthin	ERBB4
Doradexanthin	HSP90AA1
Doradexanthin	PPARG
Doradexanthin	MMP13
Doradexanthin	BLVRB
Doradexanthin	PDE4B
Doradexanthin	WAS
Doradexanthin	PARP1
Doradexanthin	CALM1
Doradexanthin	CALM2
Doradexanthin	CALM3
Doradexanthin	RARA
Doradexanthin	ELANE
Doradexanthin	ESRRG
Doradexanthin	TNNC1
Doradexanthin	PPARA
Doradexanthin	ADAM17
Doradexanthin	MAPK1
Doradexanthin	PTPN11
Doradexanthin	TGFBR1
Doradexanthin	FNTA
Doradexanthin	GSK3B
Doradexanthin	MAPKAPK2
Doradexanthin	FGFR1
Doradexanthin	ZAP70
Doradexanthin	SRC
Doradexanthin	SYK
Doradexanthin	ITK
Ergosta-7,22E-dien-3b	RORA
Ergosta-7,22E-dien-3b	TTR
Ergosta-7,22E-dien-3b	CRABP2
Ergosta-7,22E-dien-3b	TTPA
Ergosta-7,22E-dien-3b	RBP4
Ergosta-7,22E-dien-3b	MAOB
Ergosta-7,22E-dien-3b	HSD17B1
Ergosta-7,22E-dien-3b	VDR
Ergosta-7,22E-dien-3b	MAP2K1
Ergosta-7,22E-dien-3b	SEC14L2
Ergosta-7,22E-dien-3b	SULT2B1
Ergosta-7,22E-dien-3b	MAPK14

Ergosta-7,22E-dien-3ł NR3C2
Ergosta-7,22E-dien-3ł SHBG
Ergosta-7,22E-dien-3ł RXRB
Ergosta-7,22E-dien-3ł ALB
Ergosta-7,22E-dien-3ł RARB
Ergosta-7,22E-dien-3ł METAP2
Ergosta-7,22E-dien-3ł PPP1CC
Ergosta-7,22E-dien-3ł FECH
Ergosta-7,22E-dien-3ł LSS
Ergosta-7,22E-dien-3ł CASP3
Ergosta-7,22E-dien-3ł CA2
Ergosta-7,22E-dien-3ł FABP6
Ergosta-7,22E-dien-3ł RXRA
Ergosta-7,22E-dien-3ł HPGDS
Ergosta-7,22E-dien-3ł THRB
Ergosta-7,22E-dien-3ł PROCR
Ergosta-7,22E-dien-3ł RARG
Ergosta-7,22E-dien-3ł NR1H3
Ergosta-7,22E-dien-3ł ESR2
Ergosta-7,22E-dien-3ł MET
Ergosta-7,22E-dien-3ł CDK2
Ergosta-7,22E-dien-3ł TRAPPC3
Ergosta-7,22E-dien-3ł HSD11B1
Ergosta-7,22E-dien-3ł GLO1
Ergosta-7,22E-dien-3ł GSTA1
Ergosta-7,22E-dien-3ł MMP3
Ergosta-7,22E-dien-3ł CALM1
Ergosta-7,22E-dien-3ł CALM2
Ergosta-7,22E-dien-3ł CALM3
Ergosta-7,22E-dien-3ł MAPK10
Ergosta-7,22E-dien-3ł FABP7
Ergosta-7,22E-dien-3ł PGR
Ergosta-7,22E-dien-3ł RARA
Ergosta-7,22E-dien-3ł PCTP
Ergosta-7,22E-dien-3ł NR1H2
Ergosta-7,22E-dien-3ł PTPN1
Ergosta-7,22E-dien-3ł AR
Ergosta-7,22E-dien-3ł PPARG
Ergosta-7,22E-dien-3ł ADK
Ergosta-7,22E-dien-3ł AKR1B1
Ergosta-7,22E-dien-3ł ADH
Ergosta-7,22E-dien-3ł MAPK1
Ergosta-7,22E-dien-3ł MMP13
Ergosta-7,22E-dien-3ł KIF11
Ergosta-7,22E-dien-3ł TEK
Ergosta-7,22E-dien-3ł CHEK1
Ergosta-7,22E-dien-3ł HNMT
Ergosta-7,22E-dien-3ł NR1I2

Ergosta-7,22E-dien-3ł GSTP1
Ergosta-7,22E-dien-3ł KDR
Ergosta-7,22E-dien-3ł PIK3R1
Ergosta-7,22E-dien-3ł NR1H4
Ergosta-7,22E-dien-3ł EPHB4
Ergosta-7,22E-dien-3ł HNF4G
Ergosta-7,22E-dien-3ł CYP2C8
Ergosta-7,22E-dien-3ł LCK
Ergosta-7,22E-dien-3ł HSP90AA1
Ergosta-7,22E-dien-3ł BACE1
Ergosta-7,22E-dien-3ł FABP3
Ergosta-7,22E-dien-3ł ESR1
Ergosta-7,22E-dien-3ł DPP4
Ergosta-7,22E-dien-3ł PPARD
Ergosta-7,22E-dien-3ł DHODH
Ergosta-7,22E-dien-3ł GRB2
Ergosta-7,22E-dien-3ł NQO1
Ergosta-7,22E-dien-3ł PRKACA
Ergosta-7,22E-dien-3ł SULT2A1
Ergosta-7,22E-dien-3ł BRAF
Ergosta-7,22E-dien-3ł IL2
Ergosta-7,22E-dien-3ł SULT1E1
Ergosta-7,22E-dien-3ł SYK
Ergosta-7,22E-dien-3ł ITGAL
Ergosta-7,22E-dien-3ł REN
Ergosta-7,22E-dien-3ł HMGCR
Ergosta-7,22E-dien-3ł PDE4B
Ergosta-7,22E-dien-3ł WAS
Ergosta-7,22E-dien-3ł PDE4D
Ergosta-7,22E-dien-3ł HDAC8
Ergosta-7,22E-dien-3ł MTAP
Ergosta-7,22E-dien-3ł PTPN11
Ergosta-7,22E-dien-3ł BLVRB
Ergosta-7,22E-dien-3ł PLA2G2A
Ergosta-7,22E-dien-3ł EGFR
Ergosta-7,22E-dien-3ł GC
Ergosta-7,22E-dien-3ł GSTT2
Ergosta-7,22E-dien-3ł NR3C1
Ergosta-7,22E-dien-3ł ERBB4
Ergosta-7,22E-dien-3ł MMP2
Ergosta-7,22E-dien-3ł F2
Ergosta-7,22E-dien-3ł MMP12
Ergosta-7,22E-dien-3ł ABL1
Ergosta-7,22E-dien-3ł TGM3
Ergosta-7,22E-dien-3ł PDPK1
Ergosta-7,22E-dien-3ł ERRA
Ergosta-7,22E-dien-3ł ADAM17
Ergosta-7,22E-dien-3ł FKBP1A

Ergosta-7,22E-dien-3b	TGFBR1
Ergosta-7,22E-dien-3b	FGFR1
Ergosta-7,22E-dien-3b	MDM2
Ergosta-7,22E-dien-3b	AKR1C1
Ergosta-7,22E-dien-3b	ITK
Ergosta-7,22E-dien-3b	LCN2
Ergosta-7,22E-dien-3b	GSK3B
Ergosta-7,22E-dien-3b	KIT
Ergosta-7,22E-dien-3b	DPEP1
Ergosta-7,22E-dien-3b	NR1I3
Ergosta-7,22E-dien-3b	SERPINA1
Ergosta-7,22E-dien-3b	ZAP70
Ergosta-7,22E-dien-3b	PPARA
Ergosta-7,22E-dien-3b	CASP1
Ergosta-7,22E-dien-3b	CTSK
Ergosta-7,22E-dien-3b	CYP2C9
Ergosta-7,22E-dien-3b	F10
Ergosta-7,22E-dien-3b	GM2A
Ergosta-7,22E-dien-3b	PRKCQ
Ergosta-7,22E-dien-3b	MAPKAPK2
Ergosterol peroxide	TTPA
Ergosterol peroxide	RORA
Ergosterol peroxide	MAOB
Ergosterol peroxide	HSD17B1
Ergosterol peroxide	TTR
Ergosterol peroxide	CRABP2
Ergosterol peroxide	RXRB
Ergosterol peroxide	MAP2K1
Ergosterol peroxide	NR1H3
Ergosterol peroxide	RBP4
Ergosterol peroxide	VDR
Ergosterol peroxide	MAPK14
Ergosterol peroxide	CA2
Ergosterol peroxide	NR1H2
Ergosterol peroxide	ALB
Ergosterol peroxide	MME
Ergosterol peroxide	SULT2A1
Ergosterol peroxide	TRAPPC3
Ergosterol peroxide	METAP2
Ergosterol peroxide	FECH
Ergosterol peroxide	RARB
Ergosterol peroxide	CASP3
Ergosterol peroxide	LSS
Ergosterol peroxide	AR
Ergosterol peroxide	HSD11B1
Ergosterol peroxide	NR1I2
Ergosterol peroxide	PPP1CC
Ergosterol peroxide	FABP7

Ergosterol peroxide	DPP4
Ergosterol peroxide	RXRA
Ergosterol peroxide	RARG
Ergosterol peroxide	SEC14L2
Ergosterol peroxide	NR1H4
Ergosterol peroxide	AKR1C3
Ergosterol peroxide	RARA
Ergosterol peroxide	ADK
Ergosterol peroxide	FABP3
Ergosterol peroxide	HDAC8
Ergosterol peroxide	GSTA1
Ergosterol peroxide	PDPK1
Ergosterol peroxide	MET
Ergosterol peroxide	MAPK1
Ergosterol peroxide	CYP2C8
Ergosterol peroxide	SULT2B1
Ergosterol peroxide	FKBP1A
Ergosterol peroxide	ADAM17
Ergosterol peroxide	MAPK10
Ergosterol peroxide	THRB
Ergosterol peroxide	FABP6
Ergosterol peroxide	PRKACA
Ergosterol peroxide	NR3C1
Ergosterol peroxide	PROCR
Ergosterol peroxide	ESR1
Ergosterol peroxide	AKR1B1
Ergosterol peroxide	HPGDS
Ergosterol peroxide	MDM2
Ergosterol peroxide	GRB2
Ergosterol peroxide	GSTP1
Ergosterol peroxide	HNMT
Ergosterol peroxide	SYK
Ergosterol peroxide	PCTP
Ergosterol peroxide	DHODH
Ergosterol peroxide	REN
Ergosterol peroxide	TEK
Ergosterol peroxide	MMP12
Ergosterol peroxide	PTPN1
Ergosterol peroxide	CALM1
Ergosterol peroxide	CALM2
Ergosterol peroxide	CALM3
Ergosterol peroxide	CDK2
Ergosterol peroxide	BRAF
Ergosterol peroxide	KIT
Ergosterol peroxide	HSP90AA1
Ergosterol peroxide	S100A9
Ergosterol peroxide	F2
Ergosterol peroxide	ABL1

Ergosterol peroxide	SHBG
Ergosterol peroxide	BACE1
Ergosterol peroxide	PPARD
Ergosterol peroxide	ESR2
Ergosterol peroxide	MMP3
Ergosterol peroxide	MMP2
Ergosterol peroxide	PRKCQ
Ergosterol peroxide	GC
Ergosterol peroxide	CTSB
Ergosterol peroxide	PLA2G2A
Ergosterol peroxide	ZAP70
Ergosterol peroxide	GSK3B
Ergosterol peroxide	KIF11
Ergosterol peroxide	JAK3
Ergosterol peroxide	ERRA
Ergosterol peroxide	LCK
Ergosterol peroxide	KDR
Ergosterol peroxide	ITK
Ergosterol peroxide	MAPKAPK2
Ergosterol peroxide	IL2
Ergosterol peroxide	HNF4G
Ergosterol peroxide	F10
Ergosterol peroxide	PDE4D
Ergosterol peroxide	PDE5A
Ergosterol peroxide	PTPN11
Ergosterol peroxide	PDE4B
Ergosterol peroxide	FGFR1
Ergosterol peroxide	GLO1
Ergosterol peroxide	HMGCR
Ergosterol peroxide	CHEK1
Ergosterol peroxide	GSTT2
Ergosterol peroxide	CCNA2
Ergosterol peroxide	GART
Ergosterol peroxide	PIK3R1
Ergosterol peroxide	NQO1
Ergosterol peroxide	MTAP
Ergosterol peroxide	PGR
Ergosterol peroxide	THRA
Ergosterol peroxide	BLVRB
Ergosterol peroxide	ABO
Ergosterol peroxide	WAS
Ergosterol peroxide	TGM3
Ergosterol peroxide	ADH
Ergosterol peroxide	HSP90AB1
Ergosterol peroxide	TGFBR1
Ergosterol peroxide	PPARA
Ergosterol peroxide	NR1I3
Ergosterol peroxide	NR3C2

Ergosterol peroxide	CASP1
Ergosterol peroxide	ELANE
Ergosterol peroxide	ESRRG
Ergosterol peroxide	PPARG
Ergosterol peroxide	ITGAL
Ergosterol peroxide	EPHB4
Ergosterol peroxide	CTSK
Ethyl linolenate	TTR
Ethyl linolenate	CRABP2
Ethyl linolenate	RBP4
Ethyl linolenate	FABP7
Ethyl linolenate	CA2
Ethyl linolenate	MAOB
Ethyl linolenate	RORA
Ethyl linolenate	TTPA
Ethyl linolenate	LSS
Ethyl linolenate	ALB
Ethyl linolenate	ADH
Ethyl linolenate	MAP2K1
Ethyl linolenate	PROCR
Ethyl linolenate	AR
Ethyl linolenate	HSD11B1
Ethyl linolenate	FABP3
Ethyl linolenate	MAPK14
Ethyl linolenate	CYP2C8
Ethyl linolenate	RXRA
Ethyl linolenate	AKR1B1
Ethyl linolenate	DHODH
Ethyl linolenate	RARB
Ethyl linolenate	TRAPPC3
Ethyl linolenate	VDR
Ethyl linolenate	RXRB
Ethyl linolenate	REN
Ethyl linolenate	ESR2
Ethyl linolenate	BACE1
Ethyl linolenate	HPGDS
Ethyl linolenate	HSD17B1
Ethyl linolenate	NR1I2
Ethyl linolenate	SHBG
Ethyl linolenate	RARG
Ethyl linolenate	KIF11
Ethyl linolenate	SEC14L2
Ethyl linolenate	HCK
Ethyl linolenate	IL2
Ethyl linolenate	AKR1C2
Ethyl linolenate	METAP2
Ethyl linolenate	PIK3R1
Ethyl linolenate	DPEP1

Ethyl linolenate	HNMT
Ethyl linolenate	PTPN1
Ethyl linolenate	HMGCR
Ethyl linolenate	PPP1CC
Ethyl linolenate	F2
Ethyl linolenate	FABP6
Ethyl linolenate	ITGAL
Ethyl linolenate	ELANE
Ethyl linolenate	CHEK1
Ethyl linolenate	FECH
Ethyl linolenate	LCK
Ethyl linolenate	MMP3
Ethyl linolenate	PDE4B
Ethyl linolenate	HSP90AB1
Ethyl linolenate	MET
Ethyl linolenate	CDK2
Ethyl linolenate	CASP1
Ethyl linolenate	PTPN11
Ethyl linolenate	GSK3B
Ethyl linolenate	ERBB4
Ethyl linolenate	THRB
Ethyl linolenate	MDM2
Ethyl linolenate	F10
Ethyl linolenate	CCNA2
Ethyl linolenate	HNF4G
Ethyl linolenate	AKR1C3
Ethyl linolenate	BLVRB
Ethyl linolenate	SRC
Ethyl linolenate	MAPKAPK2
Ethyl linolenate	PPARA
Ethyl linolenate	TEK
Ethyl linolenate	CTSK
Ethyl linolenate	GSTP1
Ethyl linolenate	HDAC8
Ethyl linolenate	PPARD
Ethyl linolenate	HSP90AA1
Ethyl linolenate	JAK3
Ethyl linolenate	DPP4
Ethyl linolenate	LTA4H
Ethyl linolenate	RFK
Ethyl linolenate	GC
Ethyl linolenate	FGFR1
Ethyl linolenate	PDPK1
Ethyl linolenate	NR3C1
Ethyl linolenate	GLO1
Ethyl linolenate	PCK1
Ethyl linolenate	ESR1
Ethyl linolenate	BRAF

Ethyl linolenate	MMP2
Ethyl linolenate	ZAP70
Ethyl linolenate	GRB2
Ethyl linolenate	PLA2G2A
Ethyl linolenate	MMP12
Ethyl linolenate	KDR
Ethyl linolenate	ERRA
Ethyl linolenate	PDE4D
Ethyl linolenate	FKBP1A
Ethyl linolenate	PCTP
Ethyl linolenate	ITK
Ethyl linolenate	THRA
Ethyl linolenate	SERPINA1
Ethyl linolenate	MIF
Ethyl linolenate	PARP1
Ethyl linolenate	CASP3
Ethyl linolenate	PGR
Ethyl linolenate	ADK
Ethyl linolenate	BCL2L1
Ethyl linolenate	NR1H4
Ethyl linolenate	PRKCQ
Ethyl linolenate	TGM3
Ethyl linolenate	NR1H2
Ethyl linolenate	GCK
Ethyl linolenate	MAPK10
Ethyl linolenate	PRKACA
Ethyl linolenate	GSTA1
Ethyl linolenate	CALM1
Ethyl linolenate	CALM2
Ethyl linolenate	CALM3
Ethyl linolenate	ABO
Ethyl linolenate	WAS
Ethyl linolenate	TGFBR1
Ethyl linolenate	GM2A
Ethyl linolenate	PPARG
Ethyl linolenate	NR1I3
Ethyl linolenate	MAPK1
Ethyl linolenate	INSR
Ethyl linolenate	SULT1E1
Ethyl linolenate	ADAM17
Ethyl linolenate	SULT2B1
Ethyl linolenate	EPHX2
Ethyl oleate (NF)	CRABP2
Ethyl oleate (NF)	TTR
Ethyl oleate (NF)	RBP4
Ethyl oleate (NF)	FABP7
Ethyl oleate (NF)	CYP2C8
Ethyl oleate (NF)	MAOB

Ethyl oleate (NF)	CA2
Ethyl oleate (NF)	TTPA
Ethyl oleate (NF)	TRAPPC3
Ethyl oleate (NF)	ALB
Ethyl oleate (NF)	PROCR
Ethyl oleate (NF)	ADH
Ethyl oleate (NF)	RORA
Ethyl oleate (NF)	MET
Ethyl oleate (NF)	PPARD
Ethyl oleate (NF)	AR
Ethyl oleate (NF)	PDE4D
Ethyl oleate (NF)	VDR
Ethyl oleate (NF)	RXRB
Ethyl oleate (NF)	MMP3
Ethyl oleate (NF)	MAP2K1
Ethyl oleate (NF)	CASP1
Ethyl oleate (NF)	KIF11
Ethyl oleate (NF)	PCTP
Ethyl oleate (NF)	HPGDS
Ethyl oleate (NF)	MAPK14
Ethyl oleate (NF)	RXRA
Ethyl oleate (NF)	ELANE
Ethyl oleate (NF)	FECH
Ethyl oleate (NF)	SEC14L2
Ethyl oleate (NF)	FKBP1A
Ethyl oleate (NF)	PDPK1
Ethyl oleate (NF)	THRA
Ethyl oleate (NF)	REN
Ethyl oleate (NF)	HNF4G
Ethyl oleate (NF)	AKR1C3
Ethyl oleate (NF)	HSD17B1
Ethyl oleate (NF)	HMGCR
Ethyl oleate (NF)	RARG
Ethyl oleate (NF)	RARA
Ethyl oleate (NF)	NR1H2
Ethyl oleate (NF)	ESR2
Ethyl oleate (NF)	FABP6
Ethyl oleate (NF)	FABP3
Ethyl oleate (NF)	RARB
Ethyl oleate (NF)	PTPN1
Ethyl oleate (NF)	PRKACA
Ethyl oleate (NF)	METAP2
Ethyl oleate (NF)	PDE4B
Ethyl oleate (NF)	AKR1B1
Ethyl oleate (NF)	EGFR
Ethyl oleate (NF)	LSS
Ethyl oleate (NF)	AKR1C2
Ethyl oleate (NF)	HSD11B1

Ethyl oleate (NF)	PPP1CC
Ethyl oleate (NF)	PCK1
Ethyl oleate (NF)	MME
Ethyl oleate (NF)	ADAM17
Ethyl oleate (NF)	SULT2B1
Ethyl oleate (NF)	MMP2
Ethyl oleate (NF)	HNMT
Ethyl oleate (NF)	BACE1
Ethyl oleate (NF)	MMP12
Ethyl oleate (NF)	HSP90AA1
Ethyl oleate (NF)	CCNA2
Ethyl oleate (NF)	MAPK10
Ethyl oleate (NF)	BHMT
Ethyl oleate (NF)	IL2
Ethyl oleate (NF)	GRB2
Ethyl oleate (NF)	F2
Ethyl oleate (NF)	NR1I2
Ethyl oleate (NF)	GLO1
Ethyl oleate (NF)	INSR
Ethyl oleate (NF)	PTPN11
Ethyl oleate (NF)	BCAT2
Ethyl oleate (NF)	DPP4
Ethyl oleate (NF)	THRB
Ethyl oleate (NF)	SHBG
Ethyl oleate (NF)	HDAC8
Ethyl oleate (NF)	GSTP1
Ethyl oleate (NF)	HCK
Ethyl oleate (NF)	DPEP1
Ethyl oleate (NF)	NR3C1
Ethyl oleate (NF)	LCK
Ethyl oleate (NF)	CTSK
Ethyl oleate (NF)	ITGAL
Ethyl oleate (NF)	PPARA
Ethyl oleate (NF)	PLA2G2A
Ethyl oleate (NF)	CDK2
Ethyl oleate (NF)	JAK2
Ethyl oleate (NF)	ABO
Ethyl oleate (NF)	CHEK1
Ethyl oleate (NF)	GSK3B
Ethyl oleate (NF)	LTA4H
Ethyl oleate (NF)	SYK
Ethyl oleate (NF)	PRKCQ
Ethyl oleate (NF)	GC
Ethyl oleate (NF)	TEK
Ethyl oleate (NF)	MDM2
Ethyl oleate (NF)	DHODH
Ethyl oleate (NF)	HMOX1
Ethyl oleate (NF)	FGFR1

Ethyl oleate (NF)	KDR
Ethyl oleate (NF)	GSTA1
Ethyl oleate (NF)	ZAP70
Ethyl oleate (NF)	SULT2A1
Ethyl oleate (NF)	NR1H4
Ethyl oleate (NF)	ESR1
Ethyl oleate (NF)	JAK3
Ethyl oleate (NF)	PNMT
Ethyl oleate (NF)	ITK
Ethyl oleate (NF)	BLVRB
Ethyl oleate (NF)	EPHB4
Ethyl oleate (NF)	AKR1C1
Ethyl oleate (NF)	AGXT
Ethyl oleate (NF)	PGR
Ethyl oleate (NF)	SRC
Ethyl oleate (NF)	ADK
Ethyl oleate (NF)	TGM3
Ethyl oleate (NF)	ABL1
Ethyl oleate (NF)	SULT1E1
Ethyl oleate (NF)	CYP2C9
Ethyl oleate (NF)	TGFBR1
Ethyl oleate (NF)	MAPKAPK2
Ethyl oleate (NF)	MMP13
Ethyl oleate (NF)	SERPINA1
Ethyl oleate (NF)	BRAF
Ethyl oleate (NF)	SORD
Ethyl oleate (NF)	WAS
Hancinol	CRABP2
Hancinol	FGFR1
Hancinol	TTR
Hancinol	HSD17B1
Hancinol	MAP2K1
Hancinol	METAP2
Hancinol	MAOB
Hancinol	SULT1E1
Hancinol	PRKACA
Hancinol	VDR
Hancinol	CA2
Hancinol	AKR1C3
Hancinol	FKBP1A
Hancinol	RBP4
Hancinol	NR3C1
Hancinol	SULT2B1
Hancinol	ESRRG
Hancinol	MME
Hancinol	ADAM17
Hancinol	ALB
Hancinol	PPP1CC

Hancinol	FABP7
Hancinol	RARG
Hancinol	HSP90AA1
Hancinol	RXRA
Hancinol	AKR1C2
Hancinol	TTPA
Hancinol	F2
Hancinol	RXRB
Hancinol	GSTA1
Hancinol	PTPN1
Hancinol	MAPK14
Hancinol	FABP3
Hancinol	DHODH
Hancinol	RORA
Hancinol	RARA
Hancinol	AR
Hancinol	SHBG
Hancinol	FABP6
Hancinol	HSD11B1
Hancinol	DPP4
Hancinol	CCNA2
Hancinol	CHEK1
Hancinol	CDK2
Hancinol	RARB
Hancinol	KIT
Hancinol	PDE4D
Hancinol	LCK
Hancinol	THRB
Hancinol	NR1I2
Hancinol	HSP90AB1
Hancinol	MET
Hancinol	LTA4H
Hancinol	GSTP1
Hancinol	PDPK1
Hancinol	HMGCR
Hancinol	IL2
Hancinol	PROCR
Hancinol	PDE4B
Hancinol	PNMT
Hancinol	SRC
Hancinol	BRAF
Hancinol	INSR
Hancinol	TEK
Hancinol	HNF4G
Hancinol	BACE1
Hancinol	PCTP
Hancinol	ESR1
Hancinol	NR1H3

Hancinol	ITGAL
Hancinol	CYP2C8
Hancinol	GSK3B
Hancinol	PGR
Hancinol	ABL1
Hancinol	THRA
Hancinol	MTAP
Hancinol	DPEP1
Hancinol	HNMT
Hancinol	ADH
Hancinol	PTPN11
Hancinol	GC
Hancinol	ABO
Hancinol	NR1H2
Hancinol	FECH
Hancinol	PIM1
Hancinol	PPARG
Hancinol	ADK
Hancinol	HPGDS
Hancinol	MMP12
Hancinol	MMP3
Hancinol	PARP1
Hancinol	CES1
Hancinol	CASP1
Hancinol	ITK
Hancinol	TGM3
Hancinol	PLA2G2A
Hancinol	CYP2C9
Hancinol	SEC14L2
Hancinol	SULT2A1
Hancinol	DCK
Hancinol	AKR1B1
Hancinol	MAPKAPK2
Hancinol	MMP8
Hancinol	NR1H4
Hancinol	GRB2
Hancinol	NR1I3
Hancinol	CTSB
Hancinol	LCN2
Hancinol	ELANE
Hancinol	TGFBR1
Hancinol	TRAPPC3
Hancinol	MDM2
Hancinol	CALM1
Hancinol	CALM2
Hancinol	CALM3
Hancinol	NR3C2
Hancinol	AKR1C1

Hancinol	EGFR
Hancinol	WAS
Hancinol	CTSK
Hancinol	CTNNA1
Hancinol	KDR
Hancinol	MAPK8
Hancinol	LSS
Hancinol	JAK3
Hancinol	HDAC8
Hancinol	MMP2
Hancinol	ZAP70
Hancinol	PPARA
Hancinone C	AKR1C3
Hancinone C	CRABP2
Hancinone C	TTR
Hancinone C	MAP2K1
Hancinone C	GSTA1
Hancinone C	SHBG
Hancinone C	SULT2B1
Hancinone C	THRB
Hancinone C	MAOB
Hancinone C	HSD11B1
Hancinone C	FABP3
Hancinone C	FKBP1A
Hancinone C	AKR1B1
Hancinone C	FABP6
Hancinone C	GSTP1
Hancinone C	RXRA
Hancinone C	RBP4
Hancinone C	RARA
Hancinone C	NR3C1
Hancinone C	HSD17B1
Hancinone C	MAPK14
Hancinone C	CCNA2
Hancinone C	PTPN1
Hancinone C	DHODH
Hancinone C	FECH
Hancinone C	RXRB
Hancinone C	FABP7
Hancinone C	AR
Hancinone C	HSP90AA1
Hancinone C	RORA
Hancinone C	BACE1
Hancinone C	PNMT
Hancinone C	METAP2
Hancinone C	AKR1C1
Hancinone C	ALB
Hancinone C	PPP1CC

Hancinone C	ADH
Hancinone C	CA2
Hancinone C	VDR
Hancinone C	CHEK1
Hancinone C	AMD1
Hancinone C	ABO
Hancinone C	MMP8
Hancinone C	PROCR
Hancinone C	TTPA
Hancinone C	PDE4D
Hancinone C	ABL1
Hancinone C	PDE4B
Hancinone C	CTSK
Hancinone C	MME
Hancinone C	MAPKAPK2
Hancinone C	SERPINA1
Hancinone C	PCK1
Hancinone C	MMP12
Hancinone C	GSTA3
Hancinone C	NR1I2
Hancinone C	RARB
Hancinone C	HMGCR
Hancinone C	HADH
Hancinone C	DPEP1
Hancinone C	MET
Hancinone C	CDK2
Hancinone C	GSK3B
Hancinone C	F2
Hancinone C	SULT1E1
Hancinone C	ESR1
Hancinone C	ELANE
Hancinone C	THRA
Hancinone C	RARG
Hancinone C	GC
Hancinone C	LTA4H
Hancinone C	UCK2
Hancinone C	FGFR1
Hancinone C	PAH
Hancinone C	MMP3
Hancinone C	SEC14L2
Hancinone C	BTK
Hancinone C	PDPK1
Hancinone C	PRKACA
Hancinone C	IL2
Hancinone C	ITK
Hancinone C	AURKA
Hancinone C	HCK
Hancinone C	REN

Hancinone C	SORD
Hancinone C	TGFB2
Hancinone C	DUT
Hancinone C	SRC
Hancinone C	F10
Hancinone C	DTYMK
Hancinone C	ANXA5
Hancinone C	CASP1
Hancinone C	CBS
Hancinone C	TEK
Hancinone C	PTPN11
Hancinone C	DPP4
Hancinone C	CYP2C8
Hancinone C	GCK
Hancinone C	PPARA
Hancinone C	PLA2G2A
Hancinone C	MMP13
Hancinone C	HNMT
Hancinone C	CMA1
Hancinone C	PPARG
Hancinone C	HNF4G
Hancinone C	TRAPPC3
Hancinone C	ADK
Hancinone C	OAT
Hancinone C	AKR1C2
Hancinone C	PGR
Hancinone C	HDAC8
Hancinone C	LSS
Hancinone C	ESR2
Hancinone C	RAP2A
Hancinone C	PIM1
Hancinone C	INSR
Hancinone C	ESRRG
Hancinone C	BST1
Hancinone C	ACE
Hancinone C	CTSS
Hancinone C	KIF11
Hancinone C	DCK
Hancinone C	LCK
Hancinone C	TGM3
Hancinone C	ARL5A
Hancinone C	SULT2A1
Hancinone C	PCTP
Hancinone C	NQO1
Hancinone C	KDR
Hancinone C	SULT1A1
Hancinone C	CASP3
Hancinone C	CDK6

Hancinone C	TGFBR1
Hancinone C	PKLR
Hederagenin	RORA
Hederagenin	TTPA
Hederagenin	TTR
Hederagenin	MAOB
Hederagenin	FABP6
Hederagenin	RBP4
Hederagenin	CRABP2
Hederagenin	CA2
Hederagenin	MAP2K1
Hederagenin	FECH
Hederagenin	MAPK14
Hederagenin	AKR1B1
Hederagenin	ALB
Hederagenin	PROCR
Hederagenin	HSD17B1
Hederagenin	TRAPPC3
Hederagenin	BACE1
Hederagenin	VDR
Hederagenin	LSS
Hederagenin	PTPN1
Hederagenin	PPP1CC
Hederagenin	MET
Hederagenin	CYP2C8
Hederagenin	MAPK1
Hederagenin	SEC14L2
Hederagenin	METAP2
Hederagenin	PLA2G2A
Hederagenin	RARB
Hederagenin	RXRB
Hederagenin	SULT2B1
Hederagenin	RXRA
Hederagenin	CASP3
Hederagenin	HDAC8
Hederagenin	MMP12
Hederagenin	EPHB4
Hederagenin	HSD11B1
Hederagenin	ESR1
Hederagenin	PCTP
Hederagenin	RARG
Hederagenin	TEK
Hederagenin	MMP3
Hederagenin	PIK3R1
Hederagenin	NR1H4
Hederagenin	IL2
Hederagenin	MAPK10
Hederagenin	NR1H2

Hederagenin	AKR1C2
Hederagenin	ADH
Hederagenin	NR3C1
Hederagenin	FABP7
Hederagenin	THRB
Hederagenin	GSTA1
Hederagenin	PTPN11
Hederagenin	HSP90AA1
Hederagenin	CDK2
Hederagenin	ADK
Hederagenin	AKR1C3
Hederagenin	LCK
Hederagenin	GC
Hederagenin	PRKACA
Hederagenin	F2
Hederagenin	REN
Hederagenin	KDR
Hederagenin	PDE4B
Hederagenin	FABP3
Hederagenin	CHEK1
Hederagenin	HSP90AB1
Hederagenin	ITGAL
Hederagenin	HNMT
Hederagenin	MMP2
Hederagenin	HPGDS
Hederagenin	RARA
Hederagenin	PPARD
Hederagenin	GSTP1
Hederagenin	FKBP1A
Hederagenin	HMGCR
Hederagenin	NR1I2
Hederagenin	MAOA
Hederagenin	PDE4D
Hederagenin	ADAM17
Hederagenin	ERBB4
Hederagenin	GSK3B
Hederagenin	BLVRB
Hederagenin	KIF11
Hederagenin	DHODH
Hederagenin	BRAF
Hederagenin	MDM2
Hederagenin	ABL1
Hederagenin	GRB2
Hederagenin	GLO1
Hederagenin	AR
Hederagenin	NR3C2
Hederagenin	CALM1
Hederagenin	CALM2

Hederagenin	CALM3
Hederagenin	MME
Hederagenin	FGFR1
Hederagenin	PPARG
Hederagenin	FNTA
Hederagenin	HNF4G
Hederagenin	NR1H3
Hederagenin	F10
Hederagenin	DCK
Hederagenin	JAK3
Hederagenin	ITK
Hederagenin	WAS
Hederagenin	SULT2A1
Hederagenin	ELANE
Hederagenin	PRKCQ
Hederagenin	NR1I3
Hederagenin	ZAP70
Hederagenin	AKR1C1
Hederagenin	CYP2C9
Hederagenin	SERPINA1
Hederagenin	SYK
Hederagenin	MTAP
Hederagenin	KIT
Hederagenin	DPEP1
Hederagenin	ABO
Hederagenin	LCN2
Hederagenin	ERRA
Hederagenin	SHBG
Hederagenin	EGFR
Hederagenin	DPP4
Hederagenin	ESR2
Hederagenin	ADH1C
Hederagenin	S100A9
Hederagenin	TGFBR1
Hederagenin	MAPKAPK2
Hederagenin	PDPK1
Hydroxygenkwanin	HRAS
Hydroxygenkwanin	UCK2
Hydroxygenkwanin	HSD17B1
Hydroxygenkwanin	CDK6
Hydroxygenkwanin	PDE4D
Hydroxygenkwanin	PRKACA
Hydroxygenkwanin	INSR
Hydroxygenkwanin	PDPK1
Hydroxygenkwanin	FGFR1
Hydroxygenkwanin	SRC
Hydroxygenkwanin	MME
Hydroxygenkwanin	VDR

Hydroxygenkwanin	CRABP2
Hydroxygenkwanin	CTSS
Hydroxygenkwanin	GART
Hydroxygenkwanin	ADH
Hydroxygenkwanin	ESR1
Hydroxygenkwanin	RAB5A
Hydroxygenkwanin	CDK2
Hydroxygenkwanin	CA2
Hydroxygenkwanin	GSTP1
Hydroxygenkwanin	CBR1
Hydroxygenkwanin	METAP2
Hydroxygenkwanin	CTSK
Hydroxygenkwanin	PDE5A
Hydroxygenkwanin	GSK3B
Hydroxygenkwanin	GMPR
Hydroxygenkwanin	TAP1
Hydroxygenkwanin	CASP1
Hydroxygenkwanin	HCK
Hydroxygenkwanin	GSTM2
Hydroxygenkwanin	AKR1C3
Hydroxygenkwanin	SHBG
Hydroxygenkwanin	KAT2B
Hydroxygenkwanin	AR
Hydroxygenkwanin	KDR
Hydroxygenkwanin	HPRT1
Hydroxygenkwanin	ELANE
Hydroxygenkwanin	RARA
Hydroxygenkwanin	HDAC8
Hydroxygenkwanin	BACE1
Hydroxygenkwanin	GSTT2
Hydroxygenkwanin	CASP3
Hydroxygenkwanin	MAPK14
Hydroxygenkwanin	EIF4E
Hydroxygenkwanin	DUT
Hydroxygenkwanin	CCNA2
Hydroxygenkwanin	DHODH
Hydroxygenkwanin	DCPS
Hydroxygenkwanin	ABO
Hydroxygenkwanin	RFK
Hydroxygenkwanin	TEK
Hydroxygenkwanin	PCK1
Hydroxygenkwanin	FKBP1A
Hydroxygenkwanin	SORD
Hydroxygenkwanin	GSTA3
Hydroxygenkwanin	LCK
Hydroxygenkwanin	MTAP
Hydroxygenkwanin	HINT1
Hydroxygenkwanin	GSTZ1

Hydroxygenkwanin	SULT2B1
Hydroxygenkwanin	BIRC7
Hydroxygenkwanin	AKR1C2
Hydroxygenkwanin	RAB11A
Hydroxygenkwanin	GPI
Hydroxygenkwanin	APAF1
Hydroxygenkwanin	HK1
Hydroxygenkwanin	DTYMK
Hydroxygenkwanin	WARS
Hydroxygenkwanin	RAP2A
Hydroxygenkwanin	SIRT5
Hydroxygenkwanin	ARL5A
Hydroxygenkwanin	PTPN1
Hydroxygenkwanin	BST1
Hydroxygenkwanin	APRT
Hydroxygenkwanin	AKR1B1
Hydroxygenkwanin	FKBP3
Hydroxygenkwanin	DAPK1
Hydroxygenkwanin	MET
Hydroxygenkwanin	PNMT
Hydroxygenkwanin	ESR2
Hydroxygenkwanin	CTSF
Hydroxygenkwanin	DDX39B
Hydroxygenkwanin	AURKA
Hydroxygenkwanin	CDK7
Hydroxygenkwanin	NMNAT1
Hydroxygenkwanin	HSP90AA1
Hydroxygenkwanin	ARL5B
Hydroxygenkwanin	FGG
Hydroxygenkwanin	TPSB2
Hydroxygenkwanin	HADH
Hydroxygenkwanin	NT5M
Hydroxygenkwanin	NR1I2
Hydroxygenkwanin	SPR
Hydroxygenkwanin	PDE4B
Hydroxygenkwanin	MMP16
Hydroxygenkwanin	CSNK1G2
Hydroxygenkwanin	DCK
Hydroxygenkwanin	BHMT
Hydroxygenkwanin	LDHB
Hydroxygenkwanin	ATIC
Hydroxygenkwanin	NR3C1
Hydroxygenkwanin	MMP3
Hydroxygenkwanin	AMY1A
Hydroxygenkwanin	AMY1B
Hydroxygenkwanin	AMY1C
Hydroxygenkwanin	RAF1
Hydroxygenkwanin	GSTA1

Hydroxygenkwanin	ALB
Hydroxygenkwanin	SULT1E1
Hydroxygenkwanin	HSD11B1
Hydroxygenkwanin	DPP4
Hydroxygenkwanin	AKT1
Hydroxygenkwanin	UMPS
Hydroxygenkwanin	ANG
Hydroxygenkwanin	F2
Hydroxygenkwanin	TTR
Hydroxygenkwanin	BRAF
Hydroxygenkwanin	DHFR
Hydroxygenkwanin	SYK
Hydroxygenkwanin	HMGCR
Hydroxygenkwanin	CLK1
Hydroxygenkwanin	PIM1
Hydroxygenkwanin	F10
Hydroxygenkwanin	AHCY
Hydroxygenkwanin	MMP8
Hydroxygenkwanin	IVD
Hydroxygenkwanin	RAC1
Hydroxygenkwanin	RAB9A
Hydroxygenkwanin	RAB9B
Hydroxygenkwanin	JAK2
Hydroxygenkwanin	AMY2A
Hydroxygenkwanin	ARSA
Hydroxygenkwanin	NQO1
Hydroxygenkwanin	CRYZ
Hydroxygenkwanin	ACE
Hydroxygenkwanin	OTC
Hydroxygenkwanin	LTA4H
Hydroxygenkwanin	ARHGAP1
Hydroxygenkwanin	GSTM1
Hydroxygenkwanin	PPARA
Hydroxygenkwanin	REN
Hydroxygenkwanin	MAP2K1
Hydroxygenkwanin	OAT
Hydroxygenkwanin	LSS
Hydroxygenkwanin	GSR
Hydroxygenkwanin	KIT
Hydroxygenkwanin	TGFB2
Hydroxygenkwanin	MMP13
Hydroxygenkwanin	CHIT1
Hydroxygenkwanin	MAOB
Hydroxygenkwanin	SELE
Hydroxygenkwanin	NR1H4
Isofucosterol	RORA
Isofucosterol	TTR
Isofucosterol	CRABP2

Isofucosterol	MAOB
Isofucosterol	TTPA
Isofucosterol	RBP4
Isofucosterol	HSD17B1
Isofucosterol	MAP2K1
Isofucosterol	NR3C2
Isofucosterol	GRB2
Isofucosterol	RXRB
Isofucosterol	PPP1CC
Isofucosterol	METAP2
Isofucosterol	MAPK10
Isofucosterol	BACE1
Isofucosterol	ALB
Isofucosterol	SEC14L2
Isofucosterol	SULT2B1
Isofucosterol	SHBG
Isofucosterol	MAPK14
Isofucosterol	RARG
Isofucosterol	VDR
Isofucosterol	HNMT
Isofucosterol	CYP2C8
Isofucosterol	HNF4G
Isofucosterol	RXRA
Isofucosterol	FECH
Isofucosterol	FABP6
Isofucosterol	PPARD
Isofucosterol	CASP3
Isofucosterol	CA2
Isofucosterol	PCTP
Isofucosterol	PPARG
Isofucosterol	TRAPPC3
Isofucosterol	PROCR
Isofucosterol	LCK
Isofucosterol	NR1H4
Isofucosterol	HPGDS
Isofucosterol	AR
Isofucosterol	ADK
Isofucosterol	LSS
Isofucosterol	HSD11B1
Isofucosterol	RARB
Isofucosterol	RARA
Isofucosterol	PDE4D
Isofucosterol	GLO1
Isofucosterol	F2
Isofucosterol	ADH
Isofucosterol	NR1H3
Isofucosterol	EGFR
Isofucosterol	THRB

Isofucosterol	HDAC8
Isofucosterol	PGR
Isofucosterol	KDR
Isofucosterol	PTPN1
Isofucosterol	TEK
Isofucosterol	IL2
Isofucosterol	NR1I2
Isofucosterol	HSP90AA1
Isofucosterol	CDK2
Isofucosterol	ESR2
Isofucosterol	FABP7
Isofucosterol	CALM1
Isofucosterol	CALM2
Isofucosterol	CALM3
Isofucosterol	CHEK1
Isofucosterol	GART
Isofucosterol	AKR1C1
Isofucosterol	PIK3R1
Isofucosterol	MMP3
Isofucosterol	NR1H2
Isofucosterol	EPHB4
Isofucosterol	MET
Isofucosterol	ITGAL
Isofucosterol	GSTA1
Isofucosterol	FABP3
Isofucosterol	AKR1B1
Isofucosterol	PRKACA
Isofucosterol	NR3C1
Isofucosterol	FKBP1A
Isofucosterol	CYP2C9
Isofucosterol	PDPK1
Isofucosterol	ITK
Isofucosterol	ABL1
Isofucosterol	PDE4B
Isofucosterol	PTPN11
Isofucosterol	MMP2
Isofucosterol	GSK3B
Isofucosterol	MMP12
Isofucosterol	ADAM17
Isofucosterol	KIT
Isofucosterol	ZAP70
Isofucosterol	CCNA2
Isofucosterol	ERBB4
Isofucosterol	ESR1
Isofucosterol	SYK
Isofucosterol	PLA2G2A
Isofucosterol	DHODH
Isofucosterol	MDM2

Isofucosterol	PRKCQ
Isofucosterol	GSTP1
Isofucosterol	MAPK1
Isofucosterol	MAPKAPK2
Isofucosterol	JAK3
Isofucosterol	SULT1E1
Isofucosterol	HSP90AB1
Isofucosterol	REN
Isofucosterol	DPEP1
Isofucosterol	MMP13
Isofucosterol	NR1I3
Isofucosterol	KIF11
Isofucosterol	GC
Isofucosterol	WAS
Isofucosterol	FGFR1
Isofucosterol	NQO1
Isofucosterol	BLVRB
Isofucosterol	HMGCR
Isofucosterol	SERPINA1
Isofucosterol	SULT2A1
Isofucosterol	BRAF
Isofucosterol	CASP1
Isofucosterol	CTSB
Isofucosterol	DPP4
Isofucosterol	MAOA
Isofucosterol	PDE5A
Isofucosterol	PPARA
Isofucosterol	TGFBR1
Isofucosterol	S100A9
Kadsurenone	FKBP1A
Kadsurenone	CA2
Kadsurenone	PDE4B
Kadsurenone	CRABP2
Kadsurenone	THRB
Kadsurenone	MME
Kadsurenone	MAOB
Kadsurenone	AKR1C3
Kadsurenone	PPP1CC
Kadsurenone	NR3C1
Kadsurenone	TTR
Kadsurenone	CASP1
Kadsurenone	GSTA1
Kadsurenone	RBP4
Kadsurenone	ELANE
Kadsurenone	RORA
Kadsurenone	GSTP1
Kadsurenone	NR1I2
Kadsurenone	PDPK1

Kadsurenone	AR
Kadsurenone	PDE4D
Kadsurenone	VDR
Kadsurenone	PTPN1
Kadsurenone	MAPK14
Kadsurenone	CCNA2
Kadsurenone	THRA
Kadsurenone	LTA4H
Kadsurenone	PROCR
Kadsurenone	CTSK
Kadsurenone	RARA
Kadsurenone	AKR1C2
Kadsurenone	INSR
Kadsurenone	BACE1
Kadsurenone	KIF11
Kadsurenone	FGFR1
Kadsurenone	METAP2
Kadsurenone	HSP90AA1
Kadsurenone	RXRB
Kadsurenone	KDR
Kadsurenone	MMP3
Kadsurenone	MET
Kadsurenone	BST1
Kadsurenone	PPARG
Kadsurenone	HPGDS
Kadsurenone	NR1H3
Kadsurenone	ALB
Kadsurenone	AKR1B1
Kadsurenone	PRKACA
Kadsurenone	GLO1
Kadsurenone	HMGCR
Kadsurenone	ADH
Kadsurenone	SULT2B1
Kadsurenone	HSD17B1
Kadsurenone	PPARA
Kadsurenone	SORD
Kadsurenone	SRC
Kadsurenone	ESR1
Kadsurenone	F2
Kadsurenone	FECH
Kadsurenone	CDK2
Kadsurenone	MAP2K1
Kadsurenone	DTYMK
Kadsurenone	PCTP
Kadsurenone	HNF4G
Kadsurenone	DPEP1
Kadsurenone	FABP6
Kadsurenone	DHODH

Kadsurenone	DCK
Kadsurenone	SEC14L2
Kadsurenone	DUT
Kadsurenone	GSTM1
Kadsurenone	TGFB2
Kadsurenone	REN
Kadsurenone	ADAM33
Kadsurenone	LCK
Kadsurenone	ABO
Kadsurenone	BIRC7
Kadsurenone	CALM1
Kadsurenone	CALM2
Kadsurenone	CALM3
Kadsurenone	MMP8
Kadsurenone	NQO1
Kadsurenone	BRAF
Kadsurenone	NR1H2
Kadsurenone	MMP12
Kadsurenone	RARB
Kadsurenone	IL2
Kadsurenone	FABP7
Kadsurenone	PLA2G2A
Kadsurenone	PTPN11
Kadsurenone	DPP4
Kadsurenone	HDAC8
Kadsurenone	TYMS
Kadsurenone	MAPK1
Kadsurenone	PDE5A
Kadsurenone	ESR2
Kadsurenone	HSD11B1
Kadsurenone	HNMT
Kadsurenone	GSK3B
Kadsurenone	OTC
Kadsurenone	CTSB
Kadsurenone	LCN2
Kadsurenone	PGR
Kadsurenone	EGFR
Kadsurenone	CTSS
Kadsurenone	SHBG
Kadsurenone	BLVRB
Kadsurenone	MAPK10
Kadsurenone	ADK
Kadsurenone	SYK
Kadsurenone	TRAPPC3
Kadsurenone	CASP3
Kadsurenone	RXRA
Kadsurenone	EIF4E
Kadsurenone	TTPA

Kadsurenone	ABL1
Kadsurenone	ITK
Kadsurenone	IGF1R
Kadsurenone	LSS
Kadsurenone	GRB2
Kadsurenone	CYP2C8
Kadsurenone	FGG
Kadsurenone	FNTA
Kadsurenone	TREM1
Kadsurenone	CDA
Kadsurenone	CBR1
Kadsurenone	XIAP
Kadsurenone	FABP3
Kadsurenone	RARG
Kadsurenone	GSTA3
Kadsurenone	CHEK1
Kadsurenone	PIK3CG
Kaempferol	PDE4D
Kaempferol	UCK2
Kaempferol	HRAS
Kaempferol	VDR
Kaempferol	HSD17B1
Kaempferol	MME
Kaempferol	AKR1B1
Kaempferol	GSTP1
Kaempferol	SPR
Kaempferol	PDPK1
Kaempferol	PCK1
Kaempferol	RFK
Kaempferol	HCK
Kaempferol	EEA1
Kaempferol	CASP1
Kaempferol	SULT2B1
Kaempferol	UAP1
Kaempferol	INSR
Kaempferol	RAB5A
Kaempferol	CTSK
Kaempferol	DCXR
Kaempferol	GSTA1
Kaempferol	GPI
Kaempferol	MAPK14
Kaempferol	IMPA1
Kaempferol	HSP90AA1
Kaempferol	PLAU
Kaempferol	CCNA2
Kaempferol	HEXB
Kaempferol	ABO
Kaempferol	GMPR

Kaempferol	GSTA3
Kaempferol	LCK
Kaempferol	DUT
Kaempferol	CASP3
Kaempferol	PTPN1
Kaempferol	SULT1A1
Kaempferol	BACE1
Kaempferol	AR
Kaempferol	ADK
Kaempferol	CA2
Kaempferol	DAPK1
Kaempferol	BHMT
Kaempferol	DHODH
Kaempferol	TYMS
Kaempferol	PNMT
Kaempferol	SULT2A1
Kaempferol	CBR1
Kaempferol	APAF1
Kaempferol	GSTM2
Kaempferol	GSR
Kaempferol	GSTT2
Kaempferol	FGG
Kaempferol	ADH
Kaempferol	HNMT
Kaempferol	GART
Kaempferol	GALE
Kaempferol	CTSB
Kaempferol	RAC1
Kaempferol	CTSS
Kaempferol	LYZ
Kaempferol	PRKACA
Kaempferol	CES1
Kaempferol	DCPS
Kaempferol	SRC
Kaempferol	SERPINA1
Kaempferol	HDAC8
Kaempferol	XIAP
Kaempferol	STAT1
Kaempferol	BCAT2
Kaempferol	KIT
Kaempferol	PDE4B
Kaempferol	HPRT1
Kaempferol	TGFB2
Kaempferol	NOS2
Kaempferol	HAGH
Kaempferol	NR3C1
Kaempferol	HMGCR
Kaempferol	PPARA

Kaempferol	NMNAT1
Kaempferol	PGR
Kaempferol	HK1
Kaempferol	ESR1
Kaempferol	AKR1C3
Kaempferol	MAP2K1
Kaempferol	HADH
Kaempferol	BIRC7
Kaempferol	PDHB
Kaempferol	ZAP70
Kaempferol	EIF4E
Kaempferol	ESR2
Kaempferol	FABP6
Kaempferol	SULT1E1
Kaempferol	GSK3B
Kaempferol	JAK2
Kaempferol	PYGL
Kaempferol	PPP1CC
Kaempferol	DPP4
Kaempferol	GSTO1
Kaempferol	NR1H4
Kaempferol	BTK
Kaempferol	NQO1
Kaempferol	KDR
Kaempferol	IL2
Kaempferol	MET
Kaempferol	DHFR
Kaempferol	DCK
Kaempferol	BRAF
Kaempferol	MMP3
Kaempferol	CHEK1
Kaempferol	FABP4
Kaempferol	JAK3
Kaempferol	AKT1
Kaempferol	ABL1
Kaempferol	MAPK10
Kaempferol	MAOB
Kaempferol	KAT2B
Kaempferol	SELE
Kaempferol	MAPK8
Kaempferol	GSTM1
Kaempferol	F10
Kaempferol	MAN1B1
Kaempferol	PAH
Kaempferol	ARL5A
Kaempferol	CDK2
Kaempferol	CDK6
Kaempferol	MMP13

Kaempferol	FKBP3
Kaempferol	LGALS2
Kaempferol	SSE1
Kaempferol	HINT1
Kaempferol	MMP12
Kaempferol	METAP2
Kaempferol	MMP8
Kaempferol	RNASE2
Kaempferol	FKBP1A
Kaempferol	TGM3
Kaempferol	F2
Kaempferol	PNP
Kaempferol	RAP2A
Kaempferol	ARHGAP1
Kaempferol	ALB
Kaempferol	SHBG
Kaempferol	TTR
Lanosta-8,24-dien-3-o	RORA
Lanosta-8,24-dien-3-o	TTR
Lanosta-8,24-dien-3-o	FECH
Lanosta-8,24-dien-3-o	FABP6
Lanosta-8,24-dien-3-o	CRABP2
Lanosta-8,24-dien-3-o	RBP4
Lanosta-8,24-dien-3-o	TTPA
Lanosta-8,24-dien-3-o	VDR
Lanosta-8,24-dien-3-o	FABP3
Lanosta-8,24-dien-3-o	HSD17B1
Lanosta-8,24-dien-3-o	MAOB
Lanosta-8,24-dien-3-o	MAP2K1
Lanosta-8,24-dien-3-o	RARG
Lanosta-8,24-dien-3-o	RXRA
Lanosta-8,24-dien-3-o	THRB
Lanosta-8,24-dien-3-o	MAPK14
Lanosta-8,24-dien-3-o	RARB
Lanosta-8,24-dien-3-o	CYP2C8
Lanosta-8,24-dien-3-o	HNF4G
Lanosta-8,24-dien-3-o	PCTP
Lanosta-8,24-dien-3-o	TRAPPC3
Lanosta-8,24-dien-3-o	REN
Lanosta-8,24-dien-3-o	CASP3
Lanosta-8,24-dien-3-o	RXRB
Lanosta-8,24-dien-3-o	METAP2
Lanosta-8,24-dien-3-o	HSD11B1
Lanosta-8,24-dien-3-o	ALB
Lanosta-8,24-dien-3-o	PPP1CC
Lanosta-8,24-dien-3-o	LSS
Lanosta-8,24-dien-3-o	NR1H2
Lanosta-8,24-dien-3-o	FKBP1A

Lanosta-8,24-dien-3-o	NR3C1
Lanosta-8,24-dien-3-o	ADH
Lanosta-8,24-dien-3-o	CA2
Lanosta-8,24-dien-3-o	MAPK10
Lanosta-8,24-dien-3-o	SULT2B1
Lanosta-8,24-dien-3-o	MMP2
Lanosta-8,24-dien-3-o	SEC14L2
Lanosta-8,24-dien-3-o	HSP90AA1
Lanosta-8,24-dien-3-o	FABP7
Lanosta-8,24-dien-3-o	CALM1
Lanosta-8,24-dien-3-o	CALM2
Lanosta-8,24-dien-3-o	CALM3
Lanosta-8,24-dien-3-o	PROCR
Lanosta-8,24-dien-3-o	NR1I2
Lanosta-8,24-dien-3-o	CASP1
Lanosta-8,24-dien-3-o	PLA2G2A
Lanosta-8,24-dien-3-o	SHBG
Lanosta-8,24-dien-3-o	NR1H4
Lanosta-8,24-dien-3-o	GSTA1
Lanosta-8,24-dien-3-o	PRKACA
Lanosta-8,24-dien-3-o	AKR1C2
Lanosta-8,24-dien-3-o	AKR1B1
Lanosta-8,24-dien-3-o	SULT1E1
Lanosta-8,24-dien-3-o	DHODH
Lanosta-8,24-dien-3-o	LCK
Lanosta-8,24-dien-3-o	AR
Lanosta-8,24-dien-3-o	TEK
Lanosta-8,24-dien-3-o	MET
Lanosta-8,24-dien-3-o	ESR2
Lanosta-8,24-dien-3-o	PPARD
Lanosta-8,24-dien-3-o	PTPN1
Lanosta-8,24-dien-3-o	F2
Lanosta-8,24-dien-3-o	PDPK1
Lanosta-8,24-dien-3-o	PPARA
Lanosta-8,24-dien-3-o	MMP3
Lanosta-8,24-dien-3-o	NR3C2
Lanosta-8,24-dien-3-o	HSP90AB1
Lanosta-8,24-dien-3-o	MDM2
Lanosta-8,24-dien-3-o	GSTP1
Lanosta-8,24-dien-3-o	CDK2
Lanosta-8,24-dien-3-o	BACE1
Lanosta-8,24-dien-3-o	CHEK1
Lanosta-8,24-dien-3-o	KIF11
Lanosta-8,24-dien-3-o	MMP12
Lanosta-8,24-dien-3-o	HNMT
Lanosta-8,24-dien-3-o	ITGAL
Lanosta-8,24-dien-3-o	HDAC8
Lanosta-8,24-dien-3-o	ESR1

Lanosta-8,24-dien-3-o AKR1C1
Lanosta-8,24-dien-3-o SULT2A1
Lanosta-8,24-dien-3-o GRB2
Lanosta-8,24-dien-3-o PTPN11
Lanosta-8,24-dien-3-o CCNA2
Lanosta-8,24-dien-3-o PDE4B
Lanosta-8,24-dien-3-o PIK3R1
Lanosta-8,24-dien-3-o HMGCR
Lanosta-8,24-dien-3-o NR1H3
Lanosta-8,24-dien-3-o ABL1
Lanosta-8,24-dien-3-o NQO1
Lanosta-8,24-dien-3-o ADK
Lanosta-8,24-dien-3-o F10
Lanosta-8,24-dien-3-o ITK
Lanosta-8,24-dien-3-o ESRRG
Lanosta-8,24-dien-3-o BLVRB
Lanosta-8,24-dien-3-o MAPK1
Lanosta-8,24-dien-3-o PGR
Lanosta-8,24-dien-3-o IL2
Lanosta-8,24-dien-3-o AURKA
Lanosta-8,24-dien-3-o RARA
Lanosta-8,24-dien-3-o THRA
Lanosta-8,24-dien-3-o NR1I3
Lanosta-8,24-dien-3-o AKR1C3
Lanosta-8,24-dien-3-o DPP4
Lanosta-8,24-dien-3-o FGFR1
Lanosta-8,24-dien-3-o KDR
Lanosta-8,24-dien-3-o GSK3B
Lanosta-8,24-dien-3-o GC
Lanosta-8,24-dien-3-o ADAM17
Lanosta-8,24-dien-3-o WAS
Lanosta-8,24-dien-3-o TGFBR1
Lanosta-8,24-dien-3-o HPGDS
Lanosta-8,24-dien-3-o TGM3
Lanosta-8,24-dien-3-o JAK3
Lanosta-8,24-dien-3-o MMP13
Lanosta-8,24-dien-3-o PDE4D
Lanosta-8,24-dien-3-o PIM1
Lanosta-8,24-dien-3-o ZAP70
Lanosta-8,24-dien-3-o ABO
Lanosta-8,24-dien-3-o MAPKAPK2
Lanosta-8,24-dien-3-o CTSK
Lanosta-8,24-dien-3-o DPEP1
Lanosta-8,24-dien-3-o EGFR
Lanosta-8,24-dien-3-o PRKCQ
Lanosta-8,24-dien-3-o GM2A
Lanosta-8,24-dien-3-o LCN2
Lanosta-8,24-dien-3-o PARP1

Lanosta-8,24-dien-3-o	BRAF
Lanosta-8,24-dien-3-o	CYP2C9
Lanosta-8,24-dien-3-o	PPARG
Mairin	HSD17B1
Mairin	TTPA
Mairin	LSS
Mairin	TTR
Mairin	HSD11B1
Mairin	FABP3
Mairin	CRABP2
Mairin	FKBP1A
Mairin	ALB
Mairin	AR
Mairin	VDR
Mairin	FABP6
Mairin	FABP7
Mairin	RXRB
Mairin	AKR1C2
Mairin	RORA
Mairin	SHBG
Mairin	MMP3
Mairin	RARG
Mairin	RBP4
Mairin	PCTP
Mairin	NR3C1
Mairin	PDPK1
Mairin	PPP1CC
Mairin	THRA
Mairin	RXRA
Mairin	METAP2
Mairin	HDAC8
Mairin	CDK2
Mairin	PDE4B
Mairin	ABL1
Mairin	BHMT
Mairin	THRB
Mairin	GC
Mairin	GSTA1
Mairin	SEC14L2
Mairin	PROCR
Mairin	NR1I2
Mairin	HNF4G
Mairin	HSP90AA1
Mairin	TEK
Mairin	IL2
Mairin	SULT1E1
Mairin	PTPN1
Mairin	GSTP1

Mairin	MAOB
Mairin	CA2
Mairin	LCN2
Mairin	MAPK14
Mairin	WAS
Mairin	FECH
Mairin	SULT2B1
Mairin	HMGCR
Mairin	ABO
Mairin	MAP2K1
Mairin	RARB
Mairin	PIK3R1
Mairin	AKR1C3
Mairin	GSK3B
Mairin	DHFR
Mairin	TGM3
Mairin	DHODH
Mairin	MMP12
Mairin	NR1H2
Mairin	EPHX2
Mairin	PGR
Mairin	AKR1C1
Mairin	PDE4D
Mairin	ESR1
Mairin	HNMT
Mairin	MME
Mairin	ESRRG
Mairin	PIM1
Mairin	CYP2C8
Mairin	KIF11
Mairin	NR1H3
Mairin	KDR
Mairin	GRB2
Mairin	MAPK10
Mairin	MET
Mairin	PRKACA
Mairin	F2
Mairin	SULT2A1
Mairin	MMP13
Mairin	BACE1
Mairin	RARA
Mairin	ADK
Mairin	TRAPPC3
Mairin	BRAF
Mairin	NR3C2
Mairin	CASP1
Mairin	PPP5C
Mairin	PGF

Mairin	CTSB
Mairin	CHEK1
Mairin	DPEP1
Mairin	PARP1
Mairin	CASP3
Mairin	PTPN11
Mairin	ITGAL
Mairin	MTAP
Mairin	CCNA2
Mairin	MMP2
Mairin	SRC
Mairin	PLA2G2A
Mairin	ESR2
Mairin	LCK
Mairin	FNTA
Mairin	TGFBR1
Mairin	PPARA
Mairin	NR1I3
Mairin	IVD
Mairin	KIT
Mairin	ITK
Mairin	PSAP
Mairin	F10
Mairin	ADH1C
Mairin	MAPK8
Mairin	FGFR1
Mairin	ADH
Mairin	ACADM
Mairin	S100A9
Mairin	CALM1
Mairin	CALM2
Mairin	CALM3
Mairin	INSR
Mairin	STS
Mairin	DTYMK
Mairin	PRKCQ
Mairin	MTHFD1
Mairin	PPARG
Mairin	AKR1B1
Mairin	ADAM17
Malkangunin	HSD17B1
Malkangunin	RBP4
Malkangunin	F2
Malkangunin	VDR
Malkangunin	METAP2
Malkangunin	NR1I2
Malkangunin	AR
Malkangunin	PTPN1

Malkangunin	MMP3
Malkangunin	TTPA
Malkangunin	RXRB
Malkangunin	BACE1
Malkangunin	SHBG
Malkangunin	RARG
Malkangunin	MAP2K1
Malkangunin	HSP90AA1
Malkangunin	CRABP2
Malkangunin	MAOB
Malkangunin	CYP2C9
Malkangunin	SULT2B1
Malkangunin	MMP13
Malkangunin	AKR1C2
Malkangunin	MME
Malkangunin	DHODH
Malkangunin	RXRA
Malkangunin	PCTP
Malkangunin	THRB
Malkangunin	GSTA1
Malkangunin	FKBP1A
Malkangunin	MMP8
Malkangunin	RARB
Malkangunin	RORA
Malkangunin	LCK
Malkangunin	TTR
Malkangunin	DPP4
Malkangunin	ALB
Malkangunin	MMP12
Malkangunin	CA2
Malkangunin	LTA4H
Malkangunin	GSTP1
Malkangunin	HSD11B1
Malkangunin	AKR1C3
Malkangunin	CHEK1
Malkangunin	PDE4D
Malkangunin	MAPK14
Malkangunin	KDR
Malkangunin	JAK2
Malkangunin	CTSS
Malkangunin	CDK2
Malkangunin	HMGCR
Malkangunin	TEK
Malkangunin	ELANE
Malkangunin	PPP1CC
Malkangunin	NR1I3
Malkangunin	INSR
Malkangunin	BRAF

Malkangunin	FECH
Malkangunin	CTSK
Malkangunin	ABO
Malkangunin	DPEP1
Malkangunin	NR3C1
Malkangunin	PPARG
Malkangunin	AKR1B1
Malkangunin	F10
Malkangunin	PLA2G2A
Malkangunin	PGR
Malkangunin	MET
Malkangunin	ABL1
Malkangunin	ESR1
Malkangunin	GCK
Malkangunin	PRKACA
Malkangunin	PROCR
Malkangunin	FABP7
Malkangunin	HDAC8
Malkangunin	SULT2A1
Malkangunin	GM2A
Malkangunin	F11
Malkangunin	TGM3
Malkangunin	CCNA2
Malkangunin	HNMT
Malkangunin	FABP3
Malkangunin	MMP2
Malkangunin	NR1H2
Malkangunin	ADAM17
Malkangunin	BLVRB
Malkangunin	FABP6
Malkangunin	NQO1
Malkangunin	TGFB2
Malkangunin	PIK3R1
Malkangunin	ACADM
Malkangunin	REN
Malkangunin	PIM1
Malkangunin	GC
Malkangunin	KIT
Malkangunin	XIAP
Malkangunin	CASP3
Malkangunin	WAS
Malkangunin	RARA
Malkangunin	BIRC7
Malkangunin	CYP2C8
Malkangunin	MMP9
Malkangunin	TRAPPC3
Malkangunin	CASP1
Malkangunin	ADH

Malkangunin	IL2
Malkangunin	HNF4G
Malkangunin	CTSB
Malkangunin	PDE4B
Malkangunin	MAPK8
Malkangunin	GSK3B
Malkangunin	FABP4
Malkangunin	PAK7
Malkangunin	SULT1E1
Malkangunin	LSS
Malkangunin	TYMS
Malkangunin	ADK
Malkangunin	GRB2
Malkangunin	ZAP70
Malkangunin	JAK3
Malkangunin	CFD
Malkangunin	BCL2L1
Malkangunin	NR1H4
Malkangunin	MAPKAPK2
Malkangunin	FDPS
Malkangunin	PDPK1
Malkangunin	ESR2
Malkangunin	EIF4E
Malkangunin	SRC
Malkangunin	FNTA
Malkangunin	PIK3CG
Mandenol	TTR
Mandenol	CRABP2
Mandenol	RBP4
Mandenol	TTPA
Mandenol	VDR
Mandenol	MAOB
Mandenol	SEC14L2
Mandenol	BACE1
Mandenol	THRB
Mandenol	TRAPPC3
Mandenol	RXRA
Mandenol	RORA
Mandenol	PROCR
Mandenol	MAP2K1
Mandenol	RARB
Mandenol	PPP1CC
Mandenol	GSTP1
Mandenol	AKR1B1
Mandenol	FABP3
Mandenol	HSD11B1
Mandenol	DHODH
Mandenol	LSS

Mandenol	AR
Mandenol	ALB
Mandenol	PCTP
Mandenol	FABP7
Mandenol	CA2
Mandenol	IL2
Mandenol	HNF4G
Mandenol	HSD17B1
Mandenol	NR3C1
Mandenol	RARG
Mandenol	CYP2C8
Mandenol	PLA2G2A
Mandenol	ESR2
Mandenol	HPGDS
Mandenol	CASP3
Mandenol	CYP2C9
Mandenol	PTPN1
Mandenol	CDK2
Mandenol	PTPN11
Mandenol	METAP2
Mandenol	ADH
Mandenol	MDM2
Mandenol	MAPK14
Mandenol	RXRB
Mandenol	FKBP1A
Mandenol	GC
Mandenol	HSP90AA1
Mandenol	TGFBR1
Mandenol	PCK1
Mandenol	F2
Mandenol	FECH
Mandenol	PPARA
Mandenol	NR1I2
Mandenol	NR1H2
Mandenol	FGFR1
Mandenol	PDE4B
Mandenol	SULT1E1
Mandenol	HMGCR
Mandenol	SHBG
Mandenol	LCK
Mandenol	HNMT
Mandenol	DPP4
Mandenol	PPARD
Mandenol	TEK
Mandenol	F10
Mandenol	CALM1
Mandenol	CALM2
Mandenol	CALM3

Mandenol	CASP1
Mandenol	ADK
Mandenol	SULT2B1
Mandenol	KIF11
Mandenol	INSR
Mandenol	MMP3
Mandenol	KDR
Mandenol	PRKCQ
Mandenol	LCN2
Mandenol	ADAM17
Mandenol	FKBP3
Mandenol	PRKACA
Mandenol	MMP12
Mandenol	REN
Mandenol	ITK
Mandenol	GLO1
Mandenol	CHEK1
Mandenol	PDPK1
Mandenol	ZAP70
Mandenol	MAPKAPK2
Mandenol	PDE4D
Mandenol	AKR1C2
Mandenol	ITGAL
Mandenol	MMP2
Mandenol	NR1H3
Mandenol	HCK
Mandenol	PGR
Mandenol	PDE5A
Mandenol	BRAF
Mandenol	DPEP1
Mandenol	KIT
Mandenol	MET
Mandenol	GRB2
Mandenol	GSK3B
Mandenol	BLVRB
Mandenol	GSTA1
Mandenol	JAK3
Mandenol	THRA
Mandenol	FABP6
Mandenol	NR1H4
Mandenol	ABO
Mandenol	MAPK10
Mandenol	RARA
Mandenol	TGM3
Mandenol	ERRA
Mandenol	AKR1C3
Mandenol	MAPK1
Mandenol	ESR1

Mandenol	EPHX2
Mandenol	PSAP
Mandenol	HDAC8
Mandenol	CTSB
Mandenol	RFK
Mandenol	CCNA2
Mandenol	NQO1
Mandenol	BCAT2
Mandenol	ESRRG
Mandenol	NR3C2
Mandenol	SRC
Mandenol	MMP13
Mandenol	GM2A
Mandenol	AGXT
Mandenol	SULT2A1
Mandenol	CTSK
Mandenol	PIK3R1
Methylcimicifugoside	HSD17B1
Methylcimicifugoside	TTR
Methylcimicifugoside	PDE4B
Methylcimicifugoside	VDR
Methylcimicifugoside	RBP4
Methylcimicifugoside	CRABP2
Methylcimicifugoside	AR
Methylcimicifugoside	METAP2
Methylcimicifugoside	HSP90AA1
Methylcimicifugoside	SULT2B1
Methylcimicifugoside	HSD11B1
Methylcimicifugoside	MAP2K1
Methylcimicifugoside	CHEK1
Methylcimicifugoside	RARG
Methylcimicifugoside	SRC
Methylcimicifugoside	TTPA
Methylcimicifugoside	NR3C2
Methylcimicifugoside	HMGCR
Methylcimicifugoside	ALB
Methylcimicifugoside	MMP3
Methylcimicifugoside	CA2
Methylcimicifugoside	CYP2C9
Methylcimicifugoside	RORA
Methylcimicifugoside	GSTA1
Methylcimicifugoside	RXRA
Methylcimicifugoside	THRB
Methylcimicifugoside	BACE1
Methylcimicifugoside	NR1H2
Methylcimicifugoside	FABP3
Methylcimicifugoside	FKBP1A
Methylcimicifugoside	PTPN1

Methylcimicifugoside GSTP1
Methylcimicifugoside CALM1
Methylcimicifugoside CALM2
Methylcimicifugoside CALM3
Methylcimicifugoside PPP1CC
Methylcimicifugoside FECH
Methylcimicifugoside MAOB
Methylcimicifugoside F2
Methylcimicifugoside HCK
Methylcimicifugoside FABP6
Methylcimicifugoside PGR
Methylcimicifugoside NR1I2
Methylcimicifugoside RARB
Methylcimicifugoside FGFR1
Methylcimicifugoside SEC14L2
Methylcimicifugoside AKR1C2
Methylcimicifugoside MMP12
Methylcimicifugoside RXRB
Methylcimicifugoside MAPK14
Methylcimicifugoside HNF4G
Methylcimicifugoside REN
Methylcimicifugoside IL2
Methylcimicifugoside SHBG
Methylcimicifugoside CDK2
Methylcimicifugoside ABO
Methylcimicifugoside ADK
Methylcimicifugoside TGM3
Methylcimicifugoside AKR1C3
Methylcimicifugoside PDE4D
Methylcimicifugoside DHODH
Methylcimicifugoside ELANE
Methylcimicifugoside FABP7
Methylcimicifugoside SULT2A1
Methylcimicifugoside CASP1
Methylcimicifugoside PPARG
Methylcimicifugoside LCK
Methylcimicifugoside MMP2
Methylcimicifugoside ADH
Methylcimicifugoside MET
Methylcimicifugoside RARA
Methylcimicifugoside PROCR
Methylcimicifugoside NQO1
Methylcimicifugoside ITGAL
Methylcimicifugoside HNMT
Methylcimicifugoside CCNA2
Methylcimicifugoside NR1H4
Methylcimicifugoside HDAC8
Methylcimicifugoside TEK

Methylcimicifugoside PPARA
Methylcimicifugoside MDM2
Methylcimicifugoside GLO1
Methylcimicifugoside AKR1B1
Methylcimicifugoside NR3C1
Methylcimicifugoside TRAPPC3
Methylcimicifugoside GSTA3
Methylcimicifugoside INSR
Methylcimicifugoside ESR1
Methylcimicifugoside PCTP
Methylcimicifugoside GRB2
Methylcimicifugoside GC
Methylcimicifugoside PLA2G2A
Methylcimicifugoside PIM1
Methylcimicifugoside BRAF
Methylcimicifugoside PDPK1
Methylcimicifugoside CTSK
Methylcimicifugoside CYP2C8
Methylcimicifugoside JAK3
Methylcimicifugoside MAPKAPK2
Methylcimicifugoside NR1I3
Methylcimicifugoside ZAP70
Methylcimicifugoside PRKACA
Methylcimicifugoside ITK
Methylcimicifugoside F10
Methylcimicifugoside ESRRG
Methylcimicifugoside THRA
Methylcimicifugoside GSK3B
Methylcimicifugoside BST1
Methylcimicifugoside MMP8
Methylcimicifugoside ABL1
Methylcimicifugoside DPEP1
Methylcimicifugoside DTYMK
Methylcimicifugoside TGFB2
Methylcimicifugoside ANXA5
Methylcimicifugoside KIT
Methylcimicifugoside MMP13
Methylcimicifugoside GSTT2
Methylcimicifugoside FGG
Methylcimicifugoside ESR2
Methylcimicifugoside PTPN11
Methylcimicifugoside AKR1C1
Methylcimicifugoside AMD1
Methylcimicifugoside MAPK10
Methylcimicifugoside BLVRB
Methylcimicifugoside PIK3R1
Methylcimicifugoside LCN2
Methylcimicifugoside KDR

Methylcimicifugoside	SYK
Methylcimicifugoside	HSP90AB1
Methylcimicifugoside	NR1H3
Methylcimicifugoside	PAK7
Methylcimicifugoside	HMOX1
MOL000273	HSD17B1
MOL000273	CRABP2
MOL000273	MAOB
MOL000273	MET
MOL000273	TTR
MOL000273	TTPA
MOL000273	HSD11B1
MOL000273	LSS
MOL000273	RARB
MOL000273	VDR
MOL000273	MAPK10
MOL000273	F2
MOL000273	LTA4H
MOL000273	RBP4
MOL000273	CA2
MOL000273	TRAPPC3
MOL000273	PPP1CC
MOL000273	DHODH
MOL000273	PTPN1
MOL000273	RORA
MOL000273	IL2
MOL000273	AKR1C2
MOL000273	MAP2K1
MOL000273	CASP3
MOL000273	FKBP1A
MOL000273	REN
MOL000273	SEC14L2
MOL000273	GSK3B
MOL000273	NR1I2
MOL000273	RXRA
MOL000273	SHBG
MOL000273	ABL1
MOL000273	GSTA1
MOL000273	RARA
MOL000273	AKR1C3
MOL000273	CHEK1
MOL000273	PDPK1
MOL000273	MMP3
MOL000273	NR3C2
MOL000273	DPEP1
MOL000273	CYP2C9
MOL000273	BACE1
MOL000273	KDR

MOL000273	HNMT
MOL000273	F10
MOL000273	MAPK14
MOL000273	HSP90AA1
MOL000273	PROCR
MOL000273	ALB
MOL000273	TEK
MOL000273	LCK
MOL000273	AKR1B1
MOL000273	METAP2
MOL000273	THRB
MOL000273	FABP3
MOL000273	PRKACA
MOL000273	SRC
MOL000273	CASP1
MOL000273	BRAF
MOL000273	GSTP1
MOL000273	NR1H4
MOL000273	RARG
MOL000273	PDE4D
MOL000273	ADAM17
MOL000273	PDE4B
MOL000273	MMP2
MOL000273	ESR1
MOL000273	NR3C1
MOL000273	CDK2
MOL000273	NR1H2
MOL000273	HMGCR
MOL000273	HDAC8
MOL000273	ABO
MOL000273	AR
MOL000273	MAPKAPK2
MOL000273	FECH
MOL000273	AMD1
MOL000273	RXRB
MOL000273	PRKCQ
MOL000273	ITK
MOL000273	SULT2B1
MOL000273	PGR
MOL000273	MTAP
MOL000273	GRB2
MOL000273	JAK2
MOL000273	CYP2C8
MOL000273	MMP12
MOL000273	ZAP70
MOL000273	FABP7
MOL000273	SULT1E1
MOL000273	SULT2A1

MOL000273	MAPK1
MOL000273	NR1H3
MOL000273	ESR2
MOL000273	MAPK8
MOL000273	JAK3
MOL000273	INSR
MOL000273	GC
MOL000273	KIT
MOL000273	PCTP
MOL000273	PPARD
MOL000273	MDM2
MOL000273	HADH
MOL000273	FABP6
MOL000273	CCNA2
MOL000273	TGM3
MOL000273	PPARA
MOL000273	FGFR1
MOL000273	S100A9
MOL000273	CTSK
MOL000273	THRA
MOL000273	BLVRB
MOL000273	EPHB4
MOL000273	PLA2G2A
MOL000273	SORD
MOL000273	HNF4G
MOL000273	NQO1
MOL000273	MMP8
MOL000273	NR1I3
MOL000273	ADAM33
MOL000273	AURKA
MOL000273	GART
MOL000273	TGFBR1
MOL000273	CALM1
MOL000273	CALM2
MOL000273	CALM3
MOL000273	ANXA5
MOL000273	ERBB4
MOL000280	METAP2
MOL000280	SHBG
MOL000280	TTR
MOL000280	VDR
MOL000280	RBP4
MOL000280	MMP3
MOL000280	HSD17B1
MOL000280	TTPA
MOL000280	CASP3
MOL000280	CRABP2
MOL000280	MAP2K1

MOL000280	BACE1
MOL000280	HSD11B1
MOL000280	RORA
MOL000280	NR1I2
MOL000280	AKR1C1
MOL000280	RARG
MOL000280	HMGCR
MOL000280	PDE4B
MOL000280	MAPK14
MOL000280	CCNA2
MOL000280	F2
MOL000280	CA2
MOL000280	PPP1CC
MOL000280	AKR1C3
MOL000280	PTPN1
MOL000280	MAPK10
MOL000280	AKR1C2
MOL000280	THRA
MOL000280	PRKACA
MOL000280	IL2
MOL000280	AR
MOL000280	CDK2
MOL000280	DHODH
MOL000280	ADK
MOL000280	RXRA
MOL000280	SULT2B1
MOL000280	FABP3
MOL000280	MAOB
MOL000280	PCTP
MOL000280	PDE4D
MOL000280	PDPK1
MOL000280	MMP12
MOL000280	PROCR
MOL000280	SEC14L2
MOL000280	REN
MOL000280	S100A9
MOL000280	FABP6
MOL000280	GSK3B
MOL000280	GART
MOL000280	FECH
MOL000280	NR3C2
MOL000280	RARB
MOL000280	FKBP1A
MOL000280	GSTA1
MOL000280	TGFBR1
MOL000280	PGR
MOL000280	TRAPPC3
MOL000280	ABL1

MOL000280	NR1H2
MOL000280	PLA2G2A
MOL000280	INSR
MOL000280	CYP2C8
MOL000280	ALB
MOL000280	NR1H4
MOL000280	GSTP1
MOL000280	ABO
MOL000280	HSP90AA1
MOL000280	PPARD
MOL000280	HDAC8
MOL000280	LSS
MOL000280	PPARG
MOL000280	HPGDS
MOL000280	GC
MOL000280	GCK
MOL000280	CASP1
MOL000280	AKR1B1
MOL000280	FABP7
MOL000280	ADH
MOL000280	CTSS
MOL000280	NR3C1
MOL000280	HNMT
MOL000280	MAPK8
MOL000280	MAPKAPK2
MOL000280	LTA4H
MOL000280	MMP13
MOL000280	CHEK1
MOL000280	BRAF
MOL000280	MET
MOL000280	LCK
MOL000280	RXRB
MOL000280	KDR
MOL000280	MME
MOL000280	TEK
MOL000280	ITK
MOL000280	MAPK1
MOL000280	BIRC7
MOL000280	ESR2
MOL000280	FGFR1
MOL000280	JAK3
MOL000280	PRKCQ
MOL000280	SRC
MOL000280	DPP4
MOL000280	CTSB
MOL000280	MTHFD1
MOL000280	PPARA
MOL000280	ESR1

MOL000280	THRB
MOL000280	CYP2C9
MOL000280	ZAP70
MOL000280	NQO1
MOL000280	ADAM17
MOL000280	MMP8
MOL000280	BCAT2
MOL000280	F10
MOL000280	RARA
MOL000280	NR1H3
MOL000280	EGFR
MOL000280	MMP2
MOL000280	TGM3
MOL000280	GRB2
MOL000280	SULT1E1
MOL000280	CTSK
MOL000280	JAK2
MOL000280	DPEP1
MOL000280	KIT
MOL000280	EIF4E
MOL000280	EPHB4
MOL000280	PAH
MOL000280	SULT2A1
MOL000280	GM2A
MOL000280	MMP9
MOL000280	AMY2A
MOL000280	ESRRG
MOL000285	TTR
MOL000285	MAPK10
MOL000285	MAP2K1
MOL000285	VDR
MOL000285	DCK
MOL000285	HSD17B1
MOL000285	NR3C2
MOL000285	THRB
MOL000285	CASP3
MOL000285	MAOB
MOL000285	ABL1
MOL000285	RBP4
MOL000285	TTPA
MOL000285	RORA
MOL000285	LTA4H
MOL000285	CRABP2
MOL000285	HSD11B1
MOL000285	MAPK14
MOL000285	BACE1
MOL000285	REN
MOL000285	HNMT

MOL000285	PPP1CC
MOL000285	FKBP1A
MOL000285	MMP3
MOL000285	F10
MOL000285	RARB
MOL000285	SHBG
MOL000285	CA2
MOL000285	NR1H2
MOL000285	PDE4B
MOL000285	CDK2
MOL000285	RARG
MOL000285	AR
MOL000285	DHODH
MOL000285	FABP6
MOL000285	METAP2
MOL000285	FECH
MOL000285	GSK3B
MOL000285	PCTP
MOL000285	CASP1
MOL000285	TRAPPC3
MOL000285	AKR1B1
MOL000285	AKR1C3
MOL000285	CHEK1
MOL000285	BRAF
MOL000285	NQO1
MOL000285	ALB
MOL000285	PGR
MOL000285	GSTM1
MOL000285	FABP3
MOL000285	EPHB4
MOL000285	RARA
MOL000285	PROCR
MOL000285	MMP12
MOL000285	PPARD
MOL000285	NR1I2
MOL000285	RXRB
MOL000285	CTSB
MOL000285	TGFBR1
MOL000285	PTPN1
MOL000285	CYP2C8
MOL000285	KDR
MOL000285	ADH
MOL000285	S100A9
MOL000285	HSP90AA1
MOL000285	F2
MOL000285	NR3C1
MOL000285	INSR
MOL000285	MET

MOL000285	GSTP1
MOL000285	PRKACA
MOL000285	DPEP1
MOL000285	NR1H3
MOL000285	IL2
MOL000285	DPP4
MOL000285	GM2A
MOL000285	LSS
MOL000285	JAK3
MOL000285	CYP2C9
MOL000285	ESR1
MOL000285	BIRC7
MOL000285	SEC14L2
MOL000285	LCK
MOL000285	BLVRB
MOL000285	NR1H4
MOL000285	RXRA
MOL000285	PDE4D
MOL000285	CCNA2
MOL000285	ESR2
MOL000285	TEK
MOL000285	AKR1C2
MOL000285	PIM1
MOL000285	MMP2
MOL000285	HPGDS
MOL000285	GSTM2
MOL000285	THRA
MOL000285	HMGCR
MOL000285	FABP7
MOL000285	CTSK
MOL000285	ELANE
MOL000285	HDAC8
MOL000285	FNTA
MOL000285	GSTA1
MOL000285	SYK
MOL000285	HADH
MOL000285	ABO
MOL000285	SULT2B1
MOL000285	SRC
MOL000285	ADAM33
MOL000285	PDPK1
MOL000285	AURKA
MOL000285	LCN2
MOL000285	MMP8
MOL000285	KIT
MOL000285	ZAP70
MOL000287	TTR
MOL000287	RORA

MOL000287	CRABP2
MOL000287	TTPA
MOL000287	DHODH
MOL000287	HSD17B1
MOL000287	RBP4
MOL000287	VDR
MOL000287	PTPN1
MOL000287	MAPK14
MOL000287	SULT2A1
MOL000287	MAOB
MOL000287	ABL1
MOL000287	CASP3
MOL000287	NR3C2
MOL000287	FABP7
MOL000287	AR
MOL000287	PPP1CC
MOL000287	MAP2K1
MOL000287	MAPK10
MOL000287	REN
MOL000287	MMP3
MOL000287	PCTP
MOL000287	MMP12
MOL000287	RXRA
MOL000287	RARB
MOL000287	HSD11B1
MOL000287	SHBG
MOL000287	THRA
MOL000287	F10
MOL000287	NR3C1
MOL000287	RXRB
MOL000287	RARG
MOL000287	PRKACA
MOL000287	HNMT
MOL000287	RARA
MOL000287	NR1I2
MOL000287	LTA4H
MOL000287	BACE1
MOL000287	THRB
MOL000287	PDE4D
MOL000287	FKBP1A
MOL000287	PDPK1
MOL000287	AKR1C3
MOL000287	CA2
MOL000287	PROCR
MOL000287	ALB
MOL000287	ADK
MOL000287	F2
MOL000287	GRB2

MOL000287	GSTA1
MOL000287	FABP6
MOL000287	MET
MOL000287	ADH
MOL000287	HMGCR
MOL000287	IL2
MOL000287	METAP2
MOL000287	SULT2B1
MOL000287	TRAPPC3
MOL000287	CASP1
MOL000287	SEC14L2
MOL000287	HPGDS
MOL000287	AKR1C2
MOL000287	CTSK
MOL000287	FABP3
MOL000287	NR1H4
MOL000287	CCNA2
MOL000287	PGR
MOL000287	GSK3B
MOL000287	EPHB4
MOL000287	PLA2G2A
MOL000287	PPARG
MOL000287	ABO
MOL000287	TEK
MOL000287	FECH
MOL000287	PDE4B
MOL000287	ESR1
MOL000287	AKR1B1
MOL000287	GSTP1
MOL000287	CDK2
MOL000287	HSP90AA1
MOL000287	LSS
MOL000287	GART
MOL000287	CYP2C8
MOL000287	FDPS
MOL000287	PSAP
MOL000287	MMP2
MOL000287	PPARD
MOL000287	S100A9
MOL000287	SRC
MOL000287	MME
MOL000287	CTSB
MOL000287	KDR
MOL000287	ESR2
MOL000287	BRAF
MOL000287	AKR1C1
MOL000287	LCK
MOL000287	CHEK1

MOL000287	CTSS
MOL000287	KIT
MOL000287	HDAC8
MOL000287	NR1H2
MOL000287	BIRC7
MOL000287	GC
MOL000287	TGFBR1
MOL000287	CALM1
MOL000287	CALM2
MOL000287	CALM3
MOL000287	MAPK1
MOL000287	ADAM17
MOL000287	DPEP1
MOL000287	HNF4G
MOL000287	AURKA
MOL000287	PIM1
MOL000287	LCN2
MOL000287	INSR
MOL000287	NR1H3
MOL000287	MAPK8
MOL000287	ZAP70
MOL000287	GM2A
MOL000287	PTPN11
MOL000287	FGFR1
MOL005481	TTPA
MOL005481	TTR
MOL005481	ALB
MOL005481	RORA
MOL005481	VDR
MOL005481	RXRB
MOL005481	RBP4
MOL005481	MAOB
MOL005481	NR1I2
MOL005481	LSS
MOL005481	RXRA
MOL005481	RARB
MOL005481	AR
MOL005481	FABP7
MOL005481	MAP2K1
MOL005481	FABP3
MOL005481	TEK
MOL005481	HNMT
MOL005481	METAP2
MOL005481	PCTP
MOL005481	MMP3
MOL005481	CASP3
MOL005481	HSD17B1
MOL005481	PROCR

MOL005481	THRB
MOL005481	CYP2C8
MOL005481	HMGCR
MOL005481	MAPK14
MOL005481	CRABP2
MOL005481	ESR2
MOL005481	FABP6
MOL005481	FECH
MOL005481	AKR1B1
MOL005481	ADK
MOL005481	PPARD
MOL005481	PPP1CC
MOL005481	HSD11B1
MOL005481	TRAPPC3
MOL005481	HPGDS
MOL005481	SEC14L2
MOL005481	PDE4D
MOL005481	MET
MOL005481	GSTA1
MOL005481	RARG
MOL005481	CDK2
MOL005481	BLVRB
MOL005481	DPP4
MOL005481	HDAC8
MOL005481	HNF4G
MOL005481	PLA2G2A
MOL005481	SULT2B1
MOL005481	MAPK10
MOL005481	PTPN1
MOL005481	BACE1
MOL005481	PGR
MOL005481	MAPK1
MOL005481	NR1H3
MOL005481	LCK
MOL005481	F2
MOL005481	PDE4B
MOL005481	F10
MOL005481	IL2
MOL005481	PPARA
MOL005481	NR3C1
MOL005481	KIF11
MOL005481	FKBP1A
MOL005481	GSTP1
MOL005481	NR1H4
MOL005481	DHODH
MOL005481	HSP90AA1
MOL005481	REN
MOL005481	SULT1E1

MOL005481	CA2
MOL005481	SYK
MOL005481	KIT
MOL005481	FGFR1
MOL005481	BRAF
MOL005481	ESR1
MOL005481	NR1H2
MOL005481	NR1I3
MOL005481	PPARG
MOL005481	SHBG
MOL005481	WAS
MOL005481	KDR
MOL005481	CCNA2
MOL005481	PRKACA
MOL005481	PDPK1
MOL005481	CHEK1
MOL005481	ADH
MOL005481	MMP2
MOL005481	ITK
MOL005481	SULT2A1
MOL005481	FNTA
MOL005481	RARA
MOL005481	GRB2
MOL005481	ABL1
MOL005481	GC
MOL005481	TGFBR1
MOL005481	GSK3B
MOL005481	GM2A
MOL005481	MMP12
MOL005481	MDM2
MOL005481	EGFR
MOL005481	DPEP1
MOL005481	S100A9
MOL005481	PRKCQ
MOL005481	MAPKAPK2
MOL005481	ZAP70
MOL005481	PTPN11
MOL005481	ITGAL
MOL005481	BCL2L1
MOL005481	JAK3
MOL005481	PARP1
MOL005481	EPHB4
MOL005481	ERBB4
MOL005481	MAOA
MOL005481	CALM1
MOL005481	CALM2
MOL005481	CALM3
MOL005481	PIK3R1

MOL005481	ADAM17
MOL005481	AKR1C3
MOL005481	SRC
MOL005481	PDE5A
MOL005481	PSAP
MOL005481	MMP13
Mudanpioside H	HRAS
Mudanpioside H	UCK2
Mudanpioside H	DCK
Mudanpioside H	CTSK
Mudanpioside H	SIRT5
Mudanpioside H	GSK3B
Mudanpioside H	PDE4D
Mudanpioside H	BTK
Mudanpioside H	ELANE
Mudanpioside H	PCK1
Mudanpioside H	HSD17B1
Mudanpioside H	MME
Mudanpioside H	IMPDH2
Mudanpioside H	THRA
Mudanpioside H	RAP2A
Mudanpioside H	AKR1B1
Mudanpioside H	CDK2
Mudanpioside H	RAC1
Mudanpioside H	APAF1
Mudanpioside H	GSR
Mudanpioside H	PTPN1
Mudanpioside H	BHMT
Mudanpioside H	GSTM2
Mudanpioside H	INSR
Mudanpioside H	ATIC
Mudanpioside H	GSTA1
Mudanpioside H	MET
Mudanpioside H	DHFR
Mudanpioside H	NT5M
Mudanpioside H	MMP8
Mudanpioside H	GP1BA
Mudanpioside H	AR
Mudanpioside H	KDR
Mudanpioside H	LCK
Mudanpioside H	CLK1
Mudanpioside H	DCPS
Mudanpioside H	HINT1
Mudanpioside H	FDPS
Mudanpioside H	F7
Mudanpioside H	WARS
Mudanpioside H	DAPK1
Mudanpioside H	CASP1

Mudanpioside H	SPR
Mudanpioside H	EEA1
Mudanpioside H	RAB5A
Mudanpioside H	MAPK10
Mudanpioside H	SULT1A1
Mudanpioside H	PDE5A
Mudanpioside H	HPRT1
Mudanpioside H	BACE1
Mudanpioside H	ADK
Mudanpioside H	RNASE3
Mudanpioside H	MMP3
Mudanpioside H	HADH
Mudanpioside H	ARL5B
Mudanpioside H	GART
Mudanpioside H	CASP3
Mudanpioside H	DTYMK
Mudanpioside H	GSTP1
Mudanpioside H	CCNA2
Mudanpioside H	SULT2A1
Mudanpioside H	TAP1
Mudanpioside H	CA2
Mudanpioside H	PFKFB1
Mudanpioside H	GSTA3
Mudanpioside H	MAPK14
Mudanpioside H	GPI
Mudanpioside H	SULT2B1
Mudanpioside H	AKR1C3
Mudanpioside H	NMNAT1
Mudanpioside H	EIF4E
Mudanpioside H	MMP12
Mudanpioside H	PIK3R1
Mudanpioside H	FGG
Mudanpioside H	GSTT2
Mudanpioside H	RNASE2
Mudanpioside H	SRC
Mudanpioside H	IMPA1
Mudanpioside H	METAP2
Mudanpioside H	GMPR
Mudanpioside H	VDR
Mudanpioside H	PNMT
Mudanpioside H	DUT
Mudanpioside H	MMP9
Mudanpioside H	AKT1
Mudanpioside H	F2
Mudanpioside H	KIF11
Mudanpioside H	SHMT1
Mudanpioside H	HSPA8
Mudanpioside H	BST1

Mudanpioside H	MAN1B1
Mudanpioside H	IMPDH1
Mudanpioside H	PPP1CC
Mudanpioside H	ANG
Mudanpioside H	PIM1
Mudanpioside H	GSTZ1
Mudanpioside H	LTA4H
Mudanpioside H	CBS
Mudanpioside H	ARL5A
Mudanpioside H	NOS2
Mudanpioside H	DOT1L
Mudanpioside H	EPHA2
Mudanpioside H	HSP90AA1
Mudanpioside H	GSTM1
Mudanpioside H	SHBG
Mudanpioside H	GALE
Mudanpioside H	UAP1
Mudanpioside H	ABO
Mudanpioside H	OTC
Mudanpioside H	CTSF
Mudanpioside H	FKBP1A
Mudanpioside H	AMY2A
Mudanpioside H	KAT2B
Mudanpioside H	RFK
Mudanpioside H	HAGH
Mudanpioside H	CCL5
Mudanpioside H	TGM3
Mudanpioside H	RAB9A
Mudanpioside H	RAB9B
Mudanpioside H	CHIT1
Mudanpioside H	ACPP
Mudanpioside H	RAN
Mudanpioside H	RAF1
Mudanpioside H	AURKA
Mudanpioside H	CDK7
Mudanpioside H	ISG20
Mudanpioside H	FKBP1B
Mudanpioside H	REN
Mudanpioside H	FGFR1
Mudanpioside H	HSPA1A
Mudanpioside H	PDPK1
Mudanpioside H	F11
Mudanpioside H	AK1
Mudanpioside H	AHCY
Mudanpioside H	PKLR
Mudanpioside H	IVD
Mudanpioside H	ADAM33
Mudanpioside H	HPGDS

Mudanpioside H	HK1
Mudanpioside H	ESR1
Mudanpioside H	SULT1E1
Mudanpioside H	PAH
Mudanpioside H	HSD11B1
Mudanpioside H	OAT
Mudanpioside H	ITPKA
Mudanpioside H	THRB
Mudanpioside H	ESR2
Mudanpioside H	EGFR
Mudanpioside H	AGXT
Mudanpioside H	HNMT
Mudanpioside H	CHEK1
Mudanpioside H	ZAP70
Mudanpioside H	DCXR
Mudanpioside H	F10
Mudanpioside H	CBR1
Mudanpioside H	HCK
Mudanpioside H	BMP7
Mudanpioside H	ARG1
Mudanpioside H	RND3
Mudanpioside H	SELE
Pachymic acid	TTR
Pachymic acid	TTPA
Pachymic acid	CRABP2
Pachymic acid	RBP4
Pachymic acid	MAOB
Pachymic acid	VDR
Pachymic acid	DHODH
Pachymic acid	RXRA
Pachymic acid	HSD17B1
Pachymic acid	MAPK14
Pachymic acid	MAP2K1
Pachymic acid	RXRB
Pachymic acid	ADH
Pachymic acid	FABP7
Pachymic acid	RORA
Pachymic acid	DPEP1
Pachymic acid	SEC14L2
Pachymic acid	RARB
Pachymic acid	CYP2C8
Pachymic acid	LTA4H
Pachymic acid	METAP2
Pachymic acid	PPP1CC
Pachymic acid	MAPK10
Pachymic acid	PDE4D
Pachymic acid	ALB
Pachymic acid	PGR

Pachymic acid	GSTA1
Pachymic acid	MMP3
Pachymic acid	RARG
Pachymic acid	NR1I2
Pachymic acid	FECH
Pachymic acid	AR
Pachymic acid	CA2
Pachymic acid	SULT2B1
Pachymic acid	HSD11B1
Pachymic acid	ABO
Pachymic acid	GRB2
Pachymic acid	SHBG
Pachymic acid	FKBP1A
Pachymic acid	DCK
Pachymic acid	PTPN1
Pachymic acid	AKR1C2
Pachymic acid	FABP3
Pachymic acid	PDPK1
Pachymic acid	HDAC8
Pachymic acid	DPP4
Pachymic acid	SRC
Pachymic acid	GSTP1
Pachymic acid	FABP6
Pachymic acid	REN
Pachymic acid	IL2
Pachymic acid	PCTP
Pachymic acid	ADK
Pachymic acid	NR3C1
Pachymic acid	ZAP70
Pachymic acid	HNMT
Pachymic acid	RARA
Pachymic acid	CTSK
Pachymic acid	GC
Pachymic acid	MMP2
Pachymic acid	BACE1
Pachymic acid	TRAPPC3
Pachymic acid	GSK3B
Pachymic acid	NR3C2
Pachymic acid	ELANE
Pachymic acid	CASP3
Pachymic acid	LCK
Pachymic acid	NR1H4
Pachymic acid	PLA2G2A
Pachymic acid	HSP90AA1
Pachymic acid	THRB
Pachymic acid	PROCR
Pachymic acid	HPGDS
Pachymic acid	CDK2

Pachymic acid	TPSB2
Pachymic acid	MAPKAPK2
Pachymic acid	BIRC7
Pachymic acid	AKR1B1
Pachymic acid	F2
Pachymic acid	CCNA2
Pachymic acid	BCAT2
Pachymic acid	ESR2
Pachymic acid	PRKCQ
Pachymic acid	CHEK1
Pachymic acid	THRA
Pachymic acid	CTSB
Pachymic acid	HNF4G
Pachymic acid	HMGCR
Pachymic acid	CASP1
Pachymic acid	SULT2A1
Pachymic acid	MAPK12
Pachymic acid	NR1H2
Pachymic acid	MET
Pachymic acid	KDR
Pachymic acid	EGFR
Pachymic acid	ANXA5
Pachymic acid	SERPINA1
Pachymic acid	BLVRB
Pachymic acid	MMP13
Pachymic acid	MMP12
Pachymic acid	CYP2C9
Pachymic acid	PPARA
Pachymic acid	TEK
Pachymic acid	JAK3
Pachymic acid	PTPN11
Pachymic acid	MDM2
Pachymic acid	ADAM17
Pachymic acid	PPARG
Pachymic acid	TGM3
Pachymic acid	AKR1C3
Pachymic acid	GSR
Pachymic acid	MMP8
Pachymic acid	NR1H3
Pachymic acid	LSS
Pachymic acid	FGFR1
Pachymic acid	ITK
Pachymic acid	CTSS
Pachymic acid	ESR1
Pachymic acid	PDE4B
Pachymic acid	MTHFD1
Pachymic acid	GSTM2
Pachymic acid	HCK

Pachymic acid	ERBB4
Pachymic acid	DHFR
Pachymic acid	MTAP
Pachymic acid	F7
Pachymic acid	PARP1
Pachymic acid	AMD1
Pachymic acid	SULT1E1
Pachymic acid	MAPK8
Pachymic acid	KIF11
Pachymic acid	PPARD
Pachymic acid	S100A9
Paeonidanin	AKR1B1
Paeonidanin	CA2
Paeonidanin	F2
Paeonidanin	MMP3
Paeonidanin	MAPK14
Paeonidanin	METAP2
Paeonidanin	KDR
Paeonidanin	KIT
Paeonidanin	BACE1
Paeonidanin	F10
Paeonidanin	ELANE
Paeonidanin	RARA
Paeonidanin	SYK
Paeonidanin	MAP2K1
Paeonidanin	DCK
Paeonidanin	VDR
Paeonidanin	RARG
Paeonidanin	HMGCR
Paeonidanin	CYP2C9
Paeonidanin	MAOB
Paeonidanin	PPP1CC
Paeonidanin	ALB
Paeonidanin	AKR1C3
Paeonidanin	HSD17B1
Paeonidanin	TTR
Paeonidanin	PRKACA
Paeonidanin	CASP3
Paeonidanin	DPP4
Paeonidanin	CRABP2
Paeonidanin	PTPN1
Paeonidanin	FKBP1A
Paeonidanin	FGFR1
Paeonidanin	ABL1
Paeonidanin	LCK
Paeonidanin	TEK
Paeonidanin	ABO
Paeonidanin	CTSK

Paeonidanin	REN
Paeonidanin	CDK2
Paeonidanin	CTSB
Paeonidanin	HSP90AA1
Paeonidanin	CTSS
Paeonidanin	MAPK10
Paeonidanin	RXRB
Paeonidanin	AR
Paeonidanin	MME
Paeonidanin	SHBG
Paeonidanin	MAPK1
Paeonidanin	PDE4D
Paeonidanin	AKR1C2
Paeonidanin	BIRC7
Paeonidanin	RORA
Paeonidanin	TGM3
Paeonidanin	HSD11B1
Paeonidanin	MET
Paeonidanin	IL2
Paeonidanin	CASP1
Paeonidanin	LTA4H
Paeonidanin	RXRA
Paeonidanin	GPI
Paeonidanin	DHODH
Paeonidanin	BCAT2
Paeonidanin	GSK3B
Paeonidanin	CCNA2
Paeonidanin	NR1H2
Paeonidanin	SRC
Paeonidanin	MMP8
Paeonidanin	PDE4B
Paeonidanin	DPEP1
Paeonidanin	HDAC8
Paeonidanin	ADAM33
Paeonidanin	ESR2
Paeonidanin	DTYMK
Paeonidanin	GSTP1
Paeonidanin	RBP4
Paeonidanin	THRB
Paeonidanin	GSTA1
Paeonidanin	UCK2
Paeonidanin	NR1I2
Paeonidanin	RARB
Paeonidanin	MMP12
Paeonidanin	MMP9
Paeonidanin	GSR
Paeonidanin	HCK
Paeonidanin	CTNNA1

Paeonidanin	SIRT5
Paeonidanin	FDPS
Paeonidanin	PCK1
Paeonidanin	INSR
Paeonidanin	CHEK1
Paeonidanin	FABP6
Paeonidanin	HPGDS
Paeonidanin	TTPA
Paeonidanin	TGFB2
Paeonidanin	WAS
Paeonidanin	RAP2A
Paeonidanin	PYGL
Paeonidanin	GLO1
Paeonidanin	WARS
Paeonidanin	MDM2
Paeonidanin	BRAF
Paeonidanin	HNMT
Paeonidanin	PCTP
Paeonidanin	ACADM
Paeonidanin	TGFBR1
Paeonidanin	KIF11
Paeonidanin	NR1H4
Paeonidanin	PARP1
Paeonidanin	FABP3
Paeonidanin	PPARD
Paeonidanin	JAK2
Paeonidanin	PPARG
Paeonidanin	PGR
Paeonidanin	NQO1
Paeonidanin	SULT2B1
Paeonidanin	DHFR
Paeonidanin	LSS
Paeonidanin	FABP7
Paeonidanin	SULT2A1
Paeonidanin	ADAM17
Paeonidanin	GM2A
Paeonidanin	HNF4G
Paeonidanin	TRAPPC3
Paeonidanin	ESR1
Paeonidanin	PROCR
Paeoniflorin	HSP90AA1
Paeoniflorin	FKBP1A
Paeoniflorin	F10
Paeoniflorin	LCK
Paeoniflorin	GSTA1
Paeoniflorin	HCK
Paeoniflorin	PRKACA
Paeoniflorin	CDK2

Paeoniflorin	TGM3
Paeoniflorin	NR3C1
Paeoniflorin	AR
Paeoniflorin	CRABP2
Paeoniflorin	PPP1CC
Paeoniflorin	PDPK1
Paeoniflorin	BACE1
Paeoniflorin	MAOB
Paeoniflorin	PDE4D
Paeoniflorin	PIM1
Paeoniflorin	GSTP1
Paeoniflorin	TTR
Paeoniflorin	ELANE
Paeoniflorin	HSD17B1
Paeoniflorin	ESR1
Paeoniflorin	AKR1B1
Paeoniflorin	GSK3B
Paeoniflorin	THRB
Paeoniflorin	CA2
Paeoniflorin	JAK2
Paeoniflorin	MMP3
Paeoniflorin	PDE4B
Paeoniflorin	RORA
Paeoniflorin	METAP2
Paeoniflorin	AMD1
Paeoniflorin	THRA
Paeoniflorin	VDR
Paeoniflorin	CYP2C9
Paeoniflorin	CTSK
Paeoniflorin	PLA2G2A
Paeoniflorin	AKR1C3
Paeoniflorin	PTPN1
Paeoniflorin	REN
Paeoniflorin	CTNNA1
Paeoniflorin	TTPA
Paeoniflorin	APAF1
Paeoniflorin	CHEK1
Paeoniflorin	WAS
Paeoniflorin	EGFR
Paeoniflorin	NR1I2
Paeoniflorin	HSD11B1
Paeoniflorin	KDR
Paeoniflorin	MDM2
Paeoniflorin	PAK7
Paeoniflorin	MAP2K1
Paeoniflorin	MET
Paeoniflorin	HMGCR
Paeoniflorin	TGFB2

Paeoniflorin	SHBG
Paeoniflorin	FABP6
Paeoniflorin	MAPK14
Paeoniflorin	S100A9
Paeoniflorin	ESR2
Paeoniflorin	MMP13
Paeoniflorin	FABP7
Paeoniflorin	FABP3
Paeoniflorin	AKR1C2
Paeoniflorin	F11
Paeoniflorin	ESRRG
Paeoniflorin	DPEP1
Paeoniflorin	PNMT
Paeoniflorin	RBP4
Paeoniflorin	HDAC8
Paeoniflorin	TEK
Paeoniflorin	NR1H4
Paeoniflorin	CASP1
Paeoniflorin	ABO
Paeoniflorin	F2
Paeoniflorin	PGR
Paeoniflorin	NR1H2
Paeoniflorin	FKBP3
Paeoniflorin	ADK
Paeoniflorin	ADAM17
Paeoniflorin	SRC
Paeoniflorin	FECH
Paeoniflorin	PIK3R1
Paeoniflorin	BRAF
Paeoniflorin	CTSS
Paeoniflorin	CTSB
Paeoniflorin	CDK5R1
Paeoniflorin	BCAT2
Paeoniflorin	ALB
Paeoniflorin	NQO1
Paeoniflorin	MMP12
Paeoniflorin	HSP90AB1
Paeoniflorin	LTA4H
Paeoniflorin	RARG
Paeoniflorin	HNMT
Paeoniflorin	FKBP1B
Paeoniflorin	CCNA2
Paeoniflorin	SULT2B1
Paeoniflorin	UCK2
Paeoniflorin	RXRB
Paeoniflorin	FNTA
Paeoniflorin	HRAS
Paeoniflorin	FGFR1

Paeoniflorin	IL2
Paeoniflorin	GART
Paeoniflorin	MTAP
Paeoniflorin	PROCR
Paeoniflorin	BCL2L1
Paeoniflorin	PCTP
Paeoniflorin	TGFBR1
Paeoniflorin	IGF1R
Paeoniflorin	INSR
Paeoniflorin	ZAP70
Paeoniflorin	RXRA
Paeoniflorin	DAPK1
Piperlonguminine	CRABP2
Piperlonguminine	MAPK14
Piperlonguminine	CHEK1
Piperlonguminine	HSD17B1
Piperlonguminine	TTR
Piperlonguminine	MAOB
Piperlonguminine	PLA2G2A
Piperlonguminine	AKR1B1
Piperlonguminine	MMP3
Piperlonguminine	TPSB2
Piperlonguminine	RARA
Piperlonguminine	BHMT
Piperlonguminine	LCK
Piperlonguminine	THRA
Piperlonguminine	BACE1
Piperlonguminine	GSTA1
Piperlonguminine	MAPK10
Piperlonguminine	KDR
Piperlonguminine	FGFR1
Piperlonguminine	BRAF
Piperlonguminine	NQO2
Piperlonguminine	DHODH
Piperlonguminine	GSK3B
Piperlonguminine	GLO1
Piperlonguminine	PADI4
Piperlonguminine	HSD11B1
Piperlonguminine	MMP8
Piperlonguminine	PDE4D
Piperlonguminine	TEK
Piperlonguminine	INSR
Piperlonguminine	F2
Piperlonguminine	HMGCR
Piperlonguminine	MMP12
Piperlonguminine	CASP1
Piperlonguminine	ADAM33
Piperlonguminine	NR1I2

Piperlonguminine	CCNA2
Piperlonguminine	PPP1CC
Piperlonguminine	RARG
Piperlonguminine	MMP9
Piperlonguminine	AKR1C3
Piperlonguminine	HRAS
Piperlonguminine	CA2
Piperlonguminine	HSP90AA1
Piperlonguminine	ABL1
Piperlonguminine	REN
Piperlonguminine	MET
Piperlonguminine	PIK3R1
Piperlonguminine	ELANE
Piperlonguminine	DDX39B
Piperlonguminine	ESR1
Piperlonguminine	RFK
Piperlonguminine	LTA4H
Piperlonguminine	GMPR2
Piperlonguminine	METAP2
Piperlonguminine	PRKACA
Piperlonguminine	MME
Piperlonguminine	TAP1
Piperlonguminine	PCK1
Piperlonguminine	AR
Piperlonguminine	CDK2
Piperlonguminine	CTSS
Piperlonguminine	TGFB2
Piperlonguminine	PDPK1
Piperlonguminine	VDR
Piperlonguminine	RARB
Piperlonguminine	MAP2K1
Piperlonguminine	HPGDS
Piperlonguminine	DPP4
Piperlonguminine	GSTA3
Piperlonguminine	TGM3
Piperlonguminine	ADH
Piperlonguminine	SHBG
Piperlonguminine	ALB
Piperlonguminine	KAT2B
Piperlonguminine	EPHX2
Piperlonguminine	PPARD
Piperlonguminine	GSTM2
Piperlonguminine	DUT
Piperlonguminine	RBP4
Piperlonguminine	PNMT
Piperlonguminine	GRB2
Piperlonguminine	BIRC7
Piperlonguminine	HADH

Piperlonguminine	PDE4B
Piperlonguminine	JAK2
Piperlonguminine	SRC
Piperlonguminine	GSTP1
Piperlonguminine	HCK
Piperlonguminine	DPEP1
Piperlonguminine	SORD
Piperlonguminine	FKBP1A
Piperlonguminine	HNMT
Piperlonguminine	IL2
Piperlonguminine	AKR1C2
Piperlonguminine	CTSK
Piperlonguminine	TTPA
Piperlonguminine	PTPN1
Piperlonguminine	NMNAT1
Piperlonguminine	ESR2
Piperlonguminine	KIT
Piperlonguminine	THRB
Piperlonguminine	HNF4G
Piperlonguminine	ADK
Piperlonguminine	TGFBR1
Piperlonguminine	GM2A
Piperlonguminine	ERRA
Piperlonguminine	CYP2C8
Piperlonguminine	WAS
Piperlonguminine	KIF11
Piperlonguminine	LSS
Piperlonguminine	GMPR
Piperlonguminine	MDM2
Piperlonguminine	NR1H4
Piperlonguminine	ANXA5
Piperlonguminine	RORA
Piperlonguminine	TRAPPC3
Piperlonguminine	CTSB
Piperlonguminine	ADH1C
Piperlonguminine	FABP3
Piperlonguminine	PPARG
Piperlonguminine	PCTP
Piperlonguminine	NR1H2
Piperlonguminine	ABO
Piperlonguminine	MAPK1
Piperlonguminine	FNTA
Piperlonguminine	RXRB
Piperlonguminine	MMP13
Piperlonguminine	NQO1
Piperlonguminine	CASP3
Poricoic acid A	TTR
Poricoic acid A	PPP1CC

Poricoic acid A	MMP12
Poricoic acid A	MMP3
Poricoic acid A	PTPN1
Poricoic acid A	BCAT2
Poricoic acid A	HSD17B1
Poricoic acid A	RXRB
Poricoic acid A	CRABP2
Poricoic acid A	BACE1
Poricoic acid A	GSTM1
Poricoic acid A	RBP4
Poricoic acid A	GSTP1
Poricoic acid A	TTPA
Poricoic acid A	MME
Poricoic acid A	MAOB
Poricoic acid A	GSK3B
Poricoic acid A	FABP3
Poricoic acid A	HSD11B1
Poricoic acid A	AKR1C3
Poricoic acid A	PIK3R1
Poricoic acid A	F2
Poricoic acid A	RARG
Poricoic acid A	GSTM2
Poricoic acid A	INSR
Poricoic acid A	CYP2C9
Poricoic acid A	ABL1
Poricoic acid A	SRC
Poricoic acid A	PDE4B
Poricoic acid A	MET
Poricoic acid A	NR1H2
Poricoic acid A	ALB
Poricoic acid A	HSP90AA1
Poricoic acid A	MAP2K1
Poricoic acid A	PLA2G2A
Poricoic acid A	THRA
Poricoic acid A	VDR
Poricoic acid A	CCNA2
Poricoic acid A	REN
Poricoic acid A	TEK
Poricoic acid A	PDPK1
Poricoic acid A	HNMT
Poricoic acid A	RAP2A
Poricoic acid A	AKR1C2
Poricoic acid A	GSTT2
Poricoic acid A	MAPK14
Poricoic acid A	CA2
Poricoic acid A	RXRA
Poricoic acid A	FABP7
Poricoic acid A	DTYMK

Poricoic acid A	DHODH
Poricoic acid A	GSTA1
Poricoic acid A	BTK
Poricoic acid A	LTA4H
Poricoic acid A	SHBG
Poricoic acid A	METAP2
Poricoic acid A	DPEP1
Poricoic acid A	EIF4E
Poricoic acid A	MAPK10
Poricoic acid A	BRAF
Poricoic acid A	CTSK
Poricoic acid A	TPSB2
Poricoic acid A	F7
Poricoic acid A	AKR1B1
Poricoic acid A	LCN2
Poricoic acid A	FKBP1A
Poricoic acid A	ESR1
Poricoic acid A	NR1I2
Poricoic acid A	RARB
Poricoic acid A	CTSB
Poricoic acid A	CDK2
Poricoic acid A	RORA
Poricoic acid A	FABP6
Poricoic acid A	PYGL
Poricoic acid A	GSR
Poricoic acid A	ADH
Poricoic acid A	TYMS
Poricoic acid A	CASP3
Poricoic acid A	CASP1
Poricoic acid A	MTAP
Poricoic acid A	RNASEL
Poricoic acid A	ARF4
Poricoic acid A	IL2
Poricoic acid A	AR
Poricoic acid A	LCK
Poricoic acid A	PCK1
Poricoic acid A	ABO
Poricoic acid A	AKT1
Poricoic acid A	PLAU
Poricoic acid A	ME2
Poricoic acid A	HNF4G
Poricoic acid A	GSTA3
Poricoic acid A	DDX39B
Poricoic acid A	TRAPPC3
Poricoic acid A	CDK7
Poricoic acid A	RARA
Poricoic acid A	FDPS
Poricoic acid A	HMGCR

Poricoic acid A	THRB
Poricoic acid A	PIM1
Poricoic acid A	TGFBR1
Poricoic acid A	SULT2A1
Poricoic acid A	NT5M
Poricoic acid A	F10
Poricoic acid A	AGXT
Poricoic acid A	CYP2C8
Poricoic acid A	SELE
Poricoic acid A	GART
Poricoic acid A	ACPP
Poricoic acid A	RNASE3
Poricoic acid A	MMP2
Poricoic acid A	PCTP
Poricoic acid A	UCK2
Poricoic acid A	FECH
Poricoic acid A	GRB2
Poricoic acid A	NR3C1
Poricoic acid A	CTSG
Poricoic acid A	CLIC1
Poricoic acid A	B3GAT1
Poricoic acid A	DAPK1
Poricoic acid A	NR3C2
Poricoic acid A	ITK
Poricoic acid A	ANG
Poricoic acid A	DHFR
Poricoic acid A	TPI1
Poricoic acid A	CPB1
Poricoic acid A	PNPO
Poricoic acid A	HPGDS
Poricoic acid A	MAPK12
Poricoic acid A	IGF1R
Poricoic acid A	NR1H3
Poricoic acid A	PRKACA
Poricoic acid A	ESR2
Poricoic acid A	TAP1
Poricoic acid A	PARP1
Poricoic acid B	PPP1CC
Poricoic acid B	PTPN1
Poricoic acid B	SRC
Poricoic acid B	METAP2
Poricoic acid B	GSTA1
Poricoic acid B	MMP3
Poricoic acid B	HSP90AA1
Poricoic acid B	TTR
Poricoic acid B	MAOB
Poricoic acid B	AKR1C3
Poricoic acid B	ABL1

Poricoic acid B	CRABP2
Poricoic acid B	DAPK1
Poricoic acid B	RXRB
Poricoic acid B	VDR
Poricoic acid B	EIF4E
Poricoic acid B	HRAS
Poricoic acid B	FABP3
Poricoic acid B	MMP12
Poricoic acid B	PLA2G2A
Poricoic acid B	FDPS
Poricoic acid B	ALB
Poricoic acid B	F2
Poricoic acid B	BACE1
Poricoic acid B	RARA
Poricoic acid B	CDK7
Poricoic acid B	FECH
Poricoic acid B	FKBP1A
Poricoic acid B	RAP2A
Poricoic acid B	HSD17B1
Poricoic acid B	REN
Poricoic acid B	TTPA
Poricoic acid B	GSTP1
Poricoic acid B	F7
Poricoic acid B	NR1I2
Poricoic acid B	RARG
Poricoic acid B	PRKACA
Poricoic acid B	GSK3B
Poricoic acid B	FABP6
Poricoic acid B	S100A9
Poricoic acid B	CDK2
Poricoic acid B	DTYMK
Poricoic acid B	MAPK14
Poricoic acid B	NR3C1
Poricoic acid B	UCK2
Poricoic acid B	HNMT
Poricoic acid B	DPEP1
Poricoic acid B	PPARA
Poricoic acid B	CTSK
Poricoic acid B	FABP7
Poricoic acid B	BCAT2
Poricoic acid B	CHEK1
Poricoic acid B	SULT2B1
Poricoic acid B	RBP4
Poricoic acid B	HSD11B1
Poricoic acid B	GRB2
Poricoic acid B	AKR1C2
Poricoic acid B	CA2
Poricoic acid B	RXRA

Poricoic acid B	AKR1B1
Poricoic acid B	MAPK12
Poricoic acid B	MAP2K1
Poricoic acid B	CASP3
Poricoic acid B	LCK
Poricoic acid B	MME
Poricoic acid B	BLVRB
Poricoic acid B	PGR
Poricoic acid B	PIK3R1
Poricoic acid B	HDAC8
Poricoic acid B	HNF4G
Poricoic acid B	PDE4B
Poricoic acid B	ABO
Poricoic acid B	ACPP
Poricoic acid B	PCK1
Poricoic acid B	TYMS
Poricoic acid B	HMGCR
Poricoic acid B	SHBG
Poricoic acid B	LCN2
Poricoic acid B	ADH
Poricoic acid B	PIM1
Poricoic acid B	WARS
Poricoic acid B	CCNA2
Poricoic acid B	EGFR
Poricoic acid B	DHFR
Poricoic acid B	PDE4D
Poricoic acid B	RNASE2
Poricoic acid B	AURKA
Poricoic acid B	MTAP
Poricoic acid B	CASP1
Poricoic acid B	DHODH
Poricoic acid B	PPARD
Poricoic acid B	GSTT2
Poricoic acid B	TRAPPC3
Poricoic acid B	MIF
Poricoic acid B	INSR
Poricoic acid B	ITPKA
Poricoic acid B	ME2
Poricoic acid B	GSTM1
Poricoic acid B	IL2
Poricoic acid B	RAC1
Poricoic acid B	PCTP
Poricoic acid B	PYGL
Poricoic acid B	GSR
Poricoic acid B	CYP2C8
Poricoic acid B	THRA
Poricoic acid B	MAPK10
Poricoic acid B	GART

Poricoic acid B	OAT
Poricoic acid B	NME2
Poricoic acid B	BTK
Poricoic acid B	SELE
Poricoic acid B	CSK
Poricoic acid B	CPB1
Poricoic acid B	SEC14L2
Poricoic acid B	EPHA2
Poricoic acid B	DDX39B
Poricoic acid B	RARB
Poricoic acid B	NR1H2
Poricoic acid B	CYP2C9
Poricoic acid B	TAP1
Poricoic acid B	GSTO1
Poricoic acid B	BRAF
Poricoic acid B	ADK
Poricoic acid B	ANG
Poricoic acid B	ALAD
Poricoic acid B	LYZ
Poricoic acid B	DCK
Poricoic acid B	ITK
Poricoic acid B	MAPKAPK2
Poricoic acid B	GATM
Poricoic acid B	PDPK1
Poricoic acid B	ARG1
Poricoic acid B	THRB
Poricoic acid B	RNASEL
Poricoic acid B	FKBP3
Poricoic acid B	NMNAT1
Poricoic acid B	LTA4H
Poricoic acid B	LDHB
Poricoic acid B	F10
Poricoic acid B	ZAP70
Poricoic acid C	PPP1CC
Poricoic acid C	TTR
Poricoic acid C	PLA2G2A
Poricoic acid C	MMP3
Poricoic acid C	GSTP1
Poricoic acid C	F2
Poricoic acid C	VDR
Poricoic acid C	TTPA
Poricoic acid C	RXRB
Poricoic acid C	NR3C1
Poricoic acid C	MAP2K1
Poricoic acid C	RBP4
Poricoic acid C	DTYMK
Poricoic acid C	BCAT2
Poricoic acid C	BACE1

Poricoic acid C	FABP7
Poricoic acid C	PTPN1
Poricoic acid C	CRABP2
Poricoic acid C	HSD17B1
Poricoic acid C	EIF4E
Poricoic acid C	RORA
Poricoic acid C	AKR1C3
Poricoic acid C	GSK3B
Poricoic acid C	ALB
Poricoic acid C	MAPK10
Poricoic acid C	HSD11B1
Poricoic acid C	PIK3R1
Poricoic acid C	ABL1
Poricoic acid C	FABP3
Poricoic acid C	HNMT
Poricoic acid C	MAPK14
Poricoic acid C	RXRA
Poricoic acid C	HPGDS
Poricoic acid C	RARG
Poricoic acid C	BRAF
Poricoic acid C	MME
Poricoic acid C	GSTA1
Poricoic acid C	NR3C2
Poricoic acid C	PPARD
Poricoic acid C	AKR1B1
Poricoic acid C	LTA4H
Poricoic acid C	SRC
Poricoic acid C	DPEP1
Poricoic acid C	NR1H2
Poricoic acid C	METAP2
Poricoic acid C	REN
Poricoic acid C	HDAC8
Poricoic acid C	MAOB
Poricoic acid C	PDE4B
Poricoic acid C	HSP90AA1
Poricoic acid C	DHODH
Poricoic acid C	IL2
Poricoic acid C	CYP2C8
Poricoic acid C	HMGCR
Poricoic acid C	CDK2
Poricoic acid C	AR
Poricoic acid C	GSR
Poricoic acid C	GSTT2
Poricoic acid C	TYMS
Poricoic acid C	CHEK1
Poricoic acid C	CASP1
Poricoic acid C	GART
Poricoic acid C	LCN2

Poricoic acid C	CASP3
Poricoic acid C	ITK
Poricoic acid C	ELANE
Poricoic acid C	AKT1
Poricoic acid C	INSR
Poricoic acid C	ACPP
Poricoic acid C	MMP12
Poricoic acid C	FECH
Poricoic acid C	SHBG
Poricoic acid C	PROCR
Poricoic acid C	F7
Poricoic acid C	TRAPPC3
Poricoic acid C	CDK7
Poricoic acid C	PCTP
Poricoic acid C	TAP1
Poricoic acid C	FDPS
Poricoic acid C	PCK1
Poricoic acid C	HNF4G
Poricoic acid C	WARS
Poricoic acid C	GRB2
Poricoic acid C	PDE4D
Poricoic acid C	RAP2A
Poricoic acid C	NR1I2
Poricoic acid C	SEC14L2
Poricoic acid C	S100A9
Poricoic acid C	TPSB2
Poricoic acid C	CTSK
Poricoic acid C	GSTA3
Poricoic acid C	RNASE2
Poricoic acid C	ANG
Poricoic acid C	PARP1
Poricoic acid C	FABP5
Poricoic acid C	PYGL
Poricoic acid C	NMNAT1
Poricoic acid C	PGR
Poricoic acid C	CA2
Poricoic acid C	NME2
Poricoic acid C	GSTM1
Poricoic acid C	TGFBR1
Poricoic acid C	PDPK1
Poricoic acid C	CMA1
Poricoic acid C	ABO
Poricoic acid C	LCK
Poricoic acid C	ADH
Poricoic acid C	RARA
Poricoic acid C	BTK
Poricoic acid C	BIRC7
Poricoic acid C	THRB

Poricoic acid C	SELE
Poricoic acid C	CPB1
Poricoic acid C	SULT2B1
Poricoic acid C	MTAP
Poricoic acid C	RFK
Poricoic acid C	PRKACA
Poricoic acid C	PIM1
Poricoic acid C	MET
Poricoic acid C	EGFR
Poricoic acid C	PITPNA
Poricoic acid C	AGXT
Poricoic acid C	FABP6
Poricoic acid C	PPARG
Poricoic acid C	FKBP1A
Poricoic acid C	PDE5A
Poricoic acid C	MAPKAPK2
Poricoic acid C	HRAS
Poricoic acid C	GSTO1
Poricoic acid C	JAK3
Poriferast-5-en-3beta-	RORA
Poriferast-5-en-3beta-	TTR
Poriferast-5-en-3beta-	CRABP2
Poriferast-5-en-3beta-	MAOB
Poriferast-5-en-3beta-	RBP4
Poriferast-5-en-3beta-	HSD17B1
Poriferast-5-en-3beta-	TTPA
Poriferast-5-en-3beta-	MAPK10
Poriferast-5-en-3beta-	BACE1
Poriferast-5-en-3beta-	MAP2K1
Poriferast-5-en-3beta-	FGFR1
Poriferast-5-en-3beta-	PPP1CC
Poriferast-5-en-3beta-	SULT2B1
Poriferast-5-en-3beta-	SEC14L2
Poriferast-5-en-3beta-	RARG
Poriferast-5-en-3beta-	METAP2
Poriferast-5-en-3beta-	VDR
Poriferast-5-en-3beta-	MAPK14
Poriferast-5-en-3beta-	NR3C2
Poriferast-5-en-3beta-	ALB
Poriferast-5-en-3beta-	CASP3
Poriferast-5-en-3beta-	RXRA
Poriferast-5-en-3beta-	SHBG
Poriferast-5-en-3beta-	TRAPPC3
Poriferast-5-en-3beta-	FABP6
Poriferast-5-en-3beta-	FECH
Poriferast-5-en-3beta-	RXRB
Poriferast-5-en-3beta-	PCTP
Poriferast-5-en-3beta-	FKBP1A

Poriferast-5-en-3beta-	MET
Poriferast-5-en-3beta-	KDR
Poriferast-5-en-3beta-	NR3C1
Poriferast-5-en-3beta-	RARB
Poriferast-5-en-3beta-	PROCR
Poriferast-5-en-3beta-	AKR1C2
Poriferast-5-en-3beta-	NR1H4
Poriferast-5-en-3beta-	MAPK1
Poriferast-5-en-3beta-	CYP2C8
Poriferast-5-en-3beta-	CA2
Poriferast-5-en-3beta-	MMP12
Poriferast-5-en-3beta-	THRB
Poriferast-5-en-3beta-	F2
Poriferast-5-en-3beta-	HSD11B1
Poriferast-5-en-3beta-	NR1I2
Poriferast-5-en-3beta-	FABP7
Poriferast-5-en-3beta-	RARA
Poriferast-5-en-3beta-	PPARG
Poriferast-5-en-3beta-	MMP3
Poriferast-5-en-3beta-	CDK2
Poriferast-5-en-3beta-	HNMT
Poriferast-5-en-3beta-	PTPN1
Poriferast-5-en-3beta-	HDAC8
Poriferast-5-en-3beta-	PDE4D
Poriferast-5-en-3beta-	TEK
Poriferast-5-en-3beta-	HNF4G
Poriferast-5-en-3beta-	NR1H2
Poriferast-5-en-3beta-	PPARD
Poriferast-5-en-3beta-	PRKACA
Poriferast-5-en-3beta-	LCK
Poriferast-5-en-3beta-	IL2
Poriferast-5-en-3beta-	CALM1
Poriferast-5-en-3beta-	CALM2
Poriferast-5-en-3beta-	CALM3
Poriferast-5-en-3beta-	GSTA1
Poriferast-5-en-3beta-	HSP90AA1
Poriferast-5-en-3beta-	ADH
Poriferast-5-en-3beta-	GRB2
Poriferast-5-en-3beta-	PDE4B
Poriferast-5-en-3beta-	AKR1B1
Poriferast-5-en-3beta-	PIK3R1
Poriferast-5-en-3beta-	PLA2G2A
Poriferast-5-en-3beta-	DHODH
Poriferast-5-en-3beta-	ESR2
Poriferast-5-en-3beta-	AR
Poriferast-5-en-3beta-	MMP2
Poriferast-5-en-3beta-	CHEK1
Poriferast-5-en-3beta-	FABP3

Poriferast-5-en-3beta-	PTPN11
Poriferast-5-en-3beta-	ABL1
Poriferast-5-en-3beta-	NR1I3
Poriferast-5-en-3beta-	LSS
Poriferast-5-en-3beta-	PDE5A
Poriferast-5-en-3beta-	ADK
Poriferast-5-en-3beta-	EPHB4
Poriferast-5-en-3beta-	ADAM17
Poriferast-5-en-3beta-	MTAP
Poriferast-5-en-3beta-	GC
Poriferast-5-en-3beta-	REN
Poriferast-5-en-3beta-	SULT1E1
Poriferast-5-en-3beta-	KIF11
Poriferast-5-en-3beta-	ERBB4
Poriferast-5-en-3beta-	HSP90AB1
Poriferast-5-en-3beta-	ESR1
Poriferast-5-en-3beta-	GSTP1
Poriferast-5-en-3beta-	SYK
Poriferast-5-en-3beta-	MDM2
Poriferast-5-en-3beta-	BRAF
Poriferast-5-en-3beta-	CYP2C9
Poriferast-5-en-3beta-	HPGDS
Poriferast-5-en-3beta-	NR1H3
Poriferast-5-en-3beta-	PGR
Poriferast-5-en-3beta-	TGM3
Poriferast-5-en-3beta-	BLVRB
Poriferast-5-en-3beta-	KIT
Poriferast-5-en-3beta-	AKR1C1
Poriferast-5-en-3beta-	TGFBR1
Poriferast-5-en-3beta-	ITGAL
Poriferast-5-en-3beta-	SERPINA1
Poriferast-5-en-3beta-	DPP4
Poriferast-5-en-3beta-	HMGCR
Poriferast-5-en-3beta-	SULT2A1
Poriferast-5-en-3beta-	CCNA2
Poriferast-5-en-3beta-	F10
Poriferast-5-en-3beta-	FNTA
Poriferast-5-en-3beta-	EGFR
Poriferast-5-en-3beta-	WAS
Poriferast-5-en-3beta-	CTSB
Poriferast-5-en-3beta-	ITK
Poriferast-5-en-3beta-	DPEP1
Poriferast-5-en-3beta-	INSR
Poriferast-5-en-3beta-	NQO1
Poriferast-5-en-3beta-	CASP1
Poriferast-5-en-3beta-	PRKCQ
Poriferast-5-en-3beta-	MAPKAPK2
Poriferast-5-en-3beta-	GSK3B

Poriferast-5-en-3beta-	MME
Quercetin	HRAS
Quercetin	UCK2
Quercetin	PDE4D
Quercetin	GSTP1
Quercetin	PNMT
Quercetin	APAF1
Quercetin	GSTA1
Quercetin	ELANE
Quercetin	HSD17B1
Quercetin	MME
Quercetin	RFK
Quercetin	HEXB
Quercetin	AKR1B1
Quercetin	SULT2B1
Quercetin	SPR
Quercetin	HCK
Quercetin	PDPK1
Quercetin	HSP90AA1
Quercetin	BACE1
Quercetin	INSR
Quercetin	GART
Quercetin	CCNA2
Quercetin	GPI
Quercetin	SORD
Quercetin	HPRT1
Quercetin	TEK
Quercetin	GSTT2
Quercetin	HDAC8
Quercetin	DCK
Quercetin	CTSK
Quercetin	CASP1
Quercetin	LCK
Quercetin	PDE4B
Quercetin	CA2
Quercetin	PTPN11
Quercetin	UAP1
Quercetin	GSTA3
Quercetin	HSD11B1
Quercetin	GSTM2
Quercetin	DHFR
Quercetin	MTAP
Quercetin	CASP3
Quercetin	BHMT
Quercetin	DCXR
Quercetin	SULT1A1
Quercetin	WARS
Quercetin	DTYMK

Quercetin	PRKACA
Quercetin	DUT
Quercetin	GMPR
Quercetin	SRC
Quercetin	LYZ
Quercetin	HPGDS
Quercetin	NOS2
Quercetin	ABO
Quercetin	ADK
Quercetin	AR
Quercetin	MAPK14
Quercetin	RAC1
Quercetin	CBR1
Quercetin	GSR
Quercetin	SULT2A1
Quercetin	TYMS
Quercetin	PAH
Quercetin	DHODH
Quercetin	VDR
Quercetin	PCK1
Quercetin	IMPA1
Quercetin	RAB5A
Quercetin	GSK3B
Quercetin	TPH1
Quercetin	EIF4E
Quercetin	JAK2
Quercetin	HK1
Quercetin	DAPK1
Quercetin	HNMT
Quercetin	TPI1
Quercetin	ESR1
Quercetin	FGG
Quercetin	GMPR2
Quercetin	GALK1
Quercetin	CTSS
Quercetin	PYGL
Quercetin	EEA1
Quercetin	GALE
Quercetin	MMP3
Quercetin	AKR1C2
Quercetin	RAB11A
Quercetin	CHEK1
Quercetin	ATIC
Quercetin	CHIT1
Quercetin	AHCY
Quercetin	UMPS
Quercetin	CD209
Quercetin	RAB9A

Quercetin	RAB9B
Quercetin	AURKA
Quercetin	ZAP70
Quercetin	ACAT1
Quercetin	ADH
Quercetin	KIT
Quercetin	NR3C1
Quercetin	CES1
Quercetin	AKR1C1
Quercetin	NR3C2
Quercetin	DCPS
Quercetin	SERPINA1
Quercetin	PDHB
Quercetin	NR1H4
Quercetin	FABP6
Quercetin	XIAP
Quercetin	THRB
Quercetin	PFKFB1
Quercetin	HADH
Quercetin	BST1
Quercetin	TGFB2
Quercetin	NQO1
Quercetin	MAN1B1
Quercetin	KDR
Quercetin	SULT1E1
Quercetin	BCAT2
Quercetin	PIM1
Quercetin	STAT1
Quercetin	CYP2C9
Quercetin	AKR1C3
Quercetin	MAP2K1
Quercetin	BIRC7
Quercetin	HAGH
Quercetin	ADAM33
Quercetin	ARL5B
Quercetin	FGFR1
Quercetin	CDK2
Quercetin	FKBP3
Quercetin	SSE1
Quercetin	HINT1
Quercetin	MMP12
Quercetin	METAP2
Quercetin	SIRT5
Quercetin	LCN2
Quercetin	NR1I2
Quercetin	FKBP1A
Quercetin	F2
Quercetin	CRYZ

Quercetin	RAP2A
Quercetin	ARHGAP1
Quercetin	ALB
Quercetin	TTR
Rehmannioside A	HRAS
Rehmannioside A	UCK2
Rehmannioside A	NT5M
Rehmannioside A	MAPK12
Rehmannioside A	HNMT
Rehmannioside A	MAN1B1
Rehmannioside A	DAPK1
Rehmannioside A	BACE1
Rehmannioside A	KAT2B
Rehmannioside A	DCPS
Rehmannioside A	IMPA1
Rehmannioside A	ADAM33
Rehmannioside A	HPRT1
Rehmannioside A	ADK
Rehmannioside A	CDK2
Rehmannioside A	PNP
Rehmannioside A	UAP1
Rehmannioside A	RAP2A
Rehmannioside A	GSTP1
Rehmannioside A	ABO
Rehmannioside A	HINT1
Rehmannioside A	PFKFB1
Rehmannioside A	RFK
Rehmannioside A	TAP1
Rehmannioside A	RAC1
Rehmannioside A	MAPK10
Rehmannioside A	PDPK1
Rehmannioside A	APAF1
Rehmannioside A	ARL5B
Rehmannioside A	EEA1
Rehmannioside A	PDE4D
Rehmannioside A	AKR1B1
Rehmannioside A	CHIT1
Rehmannioside A	CA2
Rehmannioside A	GSTM2
Rehmannioside A	SULT1A1
Rehmannioside A	GLTP
Rehmannioside A	GSTM1
Rehmannioside A	TGM3
Rehmannioside A	GMPR
Rehmannioside A	LCK
Rehmannioside A	SRC
Rehmannioside A	PAH
Rehmannioside A	GSK3B

Rehmannioside A	BST1
Rehmannioside A	RAB5A
Rehmannioside A	GPI
Rehmannioside A	EIF4E
Rehmannioside A	MTHFD1
Rehmannioside A	SIRT5
Rehmannioside A	LGALS2
Rehmannioside A	PIM1
Rehmannioside A	PYGL
Rehmannioside A	DUT
Rehmannioside A	ATIC
Rehmannioside A	MAPKAPK2
Rehmannioside A	PCK1
Rehmannioside A	SPR
Rehmannioside A	BTK
Rehmannioside A	VDR
Rehmannioside A	F7
Rehmannioside A	AURKA
Rehmannioside A	AMY2A
Rehmannioside A	DHFR
Rehmannioside A	MME
Rehmannioside A	GALK1
Rehmannioside A	INSR
Rehmannioside A	AKT1
Rehmannioside A	SULT2B1
Rehmannioside A	RND3
Rehmannioside A	ZAP70
Rehmannioside A	LSS
Rehmannioside A	SELE
Rehmannioside A	PDE5A
Rehmannioside A	GSTT2
Rehmannioside A	FDPS
Rehmannioside A	GSTA3
Rehmannioside A	ARL5A
Rehmannioside A	PDE4B
Rehmannioside A	FGG
Rehmannioside A	ITPKA
Rehmannioside A	B3GAT1
Rehmannioside A	GMPR2
Rehmannioside A	GART
Rehmannioside A	PTPN1
Rehmannioside A	RNASE3
Rehmannioside A	PNMT
Rehmannioside A	DTYMK
Rehmannioside A	ARF4
Rehmannioside A	CDC42
Rehmannioside A	CTSK
Rehmannioside A	EPHA2

Rehmannioside A	PKLR
Rehmannioside A	ELANE
Rehmannioside A	HSD11B1
Rehmannioside A	SULT2A1
Rehmannioside A	ARHGAP1
Rehmannioside A	F2
Rehmannioside A	PLAU
Rehmannioside A	ANG
Rehmannioside A	GSR
Rehmannioside A	AMD1
Rehmannioside A	MMP9
Rehmannioside A	NOS2
Rehmannioside A	CASP3
Rehmannioside A	WARS
Rehmannioside A	AKR1C2
Rehmannioside A	HSP90AA1
Rehmannioside A	RAB11A
Rehmannioside A	RNASE2
Rehmannioside A	CCL5
Rehmannioside A	GSTA1
Rehmannioside A	HMGCR
Rehmannioside A	METAP2
Rehmannioside A	AMY1A
Rehmannioside A	AMY1B
Rehmannioside A	AMY1C
Rehmannioside A	BMP7
Rehmannioside A	BHMT
Rehmannioside A	BRAF
Rehmannioside A	MMP8
Rehmannioside A	GALE
Rehmannioside A	PAPSS1
Rehmannioside A	LGALS3
Rehmannioside A	IL2
Rehmannioside A	HK1
Rehmannioside A	DOT1L
Rehmannioside A	GNPDA1
Rehmannioside A	GNPDA2
Rehmannioside A	PMS2
Rehmannioside A	CD209
Rehmannioside A	STAT1
Rehmannioside A	LYZ
Rehmannioside A	GCK
Rehmannioside A	SRM
Rehmannioside A	IMPDH2
Rehmannioside A	ADH
Rehmannioside A	NMNAT1
Rehmannioside A	CASP1
Rehmannioside A	DPP4

Rehmannioside A	NCS1
Rehmannioside A	CANT1
Rehmannioside A	REG1A
Rehmannioside A	CDK7
Rehmannioside A	DDX39B
Rehmannioside A	CCNA2
Rehmannioside A	FECH
Rehmannioside A	NME2
Rehmannioside A	HPGDS
Rehmannioside A	TPH1
Rehmannioside A	AHCY
Rehmannioside A	CMA1
Rehmannioside A	GSTZ1
Rehmannioside A	HCK
Rehmannioside A	MTAP
Rehmannioside A	SHMT1
Rehmannioside A	HADH
Rehmannioside A	TNK2
Rehmannioside A	HSPA8
Rehmannioside A	ISG20
Rehmannioside A	DCXR
Rehmannioside A	CTSB
Rehmannioside A	ATOX1
Rehmannioside A	AK1
Rehmannioside A	HSD17B1
Rehmannioside A	KIT
Rehmannioside A	LTA4H
Rehmannioside A	MMP16
Rehmannioside D	HRAS
Rehmannioside D	UCK2
Rehmannioside D	PNMT
Rehmannioside D	DCK
Rehmannioside D	HINT1
Rehmannioside D	GSTP1
Rehmannioside D	UAP1
Rehmannioside D	SULT2B1
Rehmannioside D	SIRT5
Rehmannioside D	EEA1
Rehmannioside D	LCK
Rehmannioside D	CHIT1
Rehmannioside D	RAP2A
Rehmannioside D	PFKFB1
Rehmannioside D	DTYMK
Rehmannioside D	CDK2
Rehmannioside D	IMPA1
Rehmannioside D	RAC1
Rehmannioside D	TGM3
Rehmannioside D	SPR

Rehmannioside D	PIM1
Rehmannioside D	RAB5A
Rehmannioside D	AKR1B1
Rehmannioside D	BACE1
Rehmannioside D	PCK1
Rehmannioside D	GSTM2
Rehmannioside D	RFK
Rehmannioside D	AKT1
Rehmannioside D	HNMT
Rehmannioside D	HADH
Rehmannioside D	GSTA1
Rehmannioside D	BIRC7
Rehmannioside D	DAPK1
Rehmannioside D	INSR
Rehmannioside D	PDE4D
Rehmannioside D	HPRT1
Rehmannioside D	CTSK
Rehmannioside D	APAF1
Rehmannioside D	BTK
Rehmannioside D	ABO
Rehmannioside D	GSK3B
Rehmannioside D	JAK2
Rehmannioside D	TAP1
Rehmannioside D	RAB11A
Rehmannioside D	ADAM33
Rehmannioside D	FKBP3
Rehmannioside D	SULT1A1
Rehmannioside D	F2
Rehmannioside D	DHFR
Rehmannioside D	AURKA
Rehmannioside D	FKBP1B
Rehmannioside D	IMPDH2
Rehmannioside D	GSTM1
Rehmannioside D	EIF4E
Rehmannioside D	PYGL
Rehmannioside D	CHEK1
Rehmannioside D	HK1
Rehmannioside D	ADK
Rehmannioside D	NOS2
Rehmannioside D	ACAT1
Rehmannioside D	LSS
Rehmannioside D	LGALS3
Rehmannioside D	BST1
Rehmannioside D	PKLR
Rehmannioside D	BHMT
Rehmannioside D	GP1BA
Rehmannioside D	HSPA1A
Rehmannioside D	AMY2A

Rehmannioside D	FGG
Rehmannioside D	MME
Rehmannioside D	AK1
Rehmannioside D	FKBP1A
Rehmannioside D	DCPS
Rehmannioside D	PTPN1
Rehmannioside D	HSP90AA1
Rehmannioside D	GMPR2
Rehmannioside D	CCL5
Rehmannioside D	RAN
Rehmannioside D	CA2
Rehmannioside D	WARS
Rehmannioside D	MAPK10
Rehmannioside D	MMP3
Rehmannioside D	SULT2A1
Rehmannioside D	TGFB2
Rehmannioside D	GSTA3
Rehmannioside D	CLK1
Rehmannioside D	SORD
Rehmannioside D	ARL5B
Rehmannioside D	MAN1B1
Rehmannioside D	RNASE2
Rehmannioside D	PDE4B
Rehmannioside D	PAPSS1
Rehmannioside D	PDPK1
Rehmannioside D	IGF1R
Rehmannioside D	CTSB
Rehmannioside D	BCAT2
Rehmannioside D	AMD1
Rehmannioside D	KIT
Rehmannioside D	ARL5A
Rehmannioside D	GMPR
Rehmannioside D	MMP9
Rehmannioside D	CASP3
Rehmannioside D	GNPDA1
Rehmannioside D	GNPDA2
Rehmannioside D	KAT2B
Rehmannioside D	CD209
Rehmannioside D	LGALS2
Rehmannioside D	NMNAT1
Rehmannioside D	CCNA2
Rehmannioside D	ANG
Rehmannioside D	DUT
Rehmannioside D	SELE
Rehmannioside D	FDPS
Rehmannioside D	CDC42
Rehmannioside D	KDR
Rehmannioside D	IMPDH1

Rehmannioside D	SRC
Rehmannioside D	NME2
Rehmannioside D	LTA4H
Rehmannioside D	GSTZ1
Rehmannioside D	GALE
Rehmannioside D	DDX39B
Rehmannioside D	GART
Rehmannioside D	MAPK12
Rehmannioside D	PAK6
Rehmannioside D	PAH
Rehmannioside D	RAB9A
Rehmannioside D	RAB9B
Rehmannioside D	DOT1L
Rehmannioside D	ARF4
Rehmannioside D	ATIC
Rehmannioside D	RAC2
Rehmannioside D	NT5M
Rehmannioside D	TNK2
Rehmannioside D	RNASE3
Rehmannioside D	ISG20
Rehmannioside D	F7
Rehmannioside D	METAP2
Rehmannioside D	GPI
Rehmannioside D	B3GAT1
Rehmannioside D	AMY1A
Rehmannioside D	AMY1B
Rehmannioside D	AMY1C
Rehmannioside D	GSTT2
Rehmannioside D	KIF11
Rehmannioside D	SRM
Rehmannioside D	HPGDS
Rehmannioside D	OTC
Rehmannioside D	CTSS
Rehmannioside D	HSD11B1
Rehmannioside D	STAT1
Rehmannioside D	LYZ
Rehmannioside D	ME2
Sitosterol	RORA
Sitosterol	RBP4
Sitosterol	TTR
Sitosterol	MAOB
Sitosterol	TTPA
Sitosterol	CRABP2
Sitosterol	HSD17B1
Sitosterol	MAPK14
Sitosterol	NR3C2
Sitosterol	MAP2K1
Sitosterol	SULT2B1

Sitosterol	GRB2
Sitosterol	RARG
Sitosterol	VDR
Sitosterol	SHBG
Sitosterol	HDAC8
Sitosterol	PPP1CC
Sitosterol	ALB
Sitosterol	SEC14L2
Sitosterol	TRAPPC3
Sitosterol	NR1H2
Sitosterol	METAP2
Sitosterol	FABP6
Sitosterol	RXRB
Sitosterol	FECH
Sitosterol	NR1H3
Sitosterol	GSTA1
Sitosterol	MAPK1
Sitosterol	THRB
Sitosterol	PROCR
Sitosterol	CASP3
Sitosterol	CA2
Sitosterol	KDR
Sitosterol	MAPK10
Sitosterol	PPARG
Sitosterol	FABP7
Sitosterol	AR
Sitosterol	NR1H4
Sitosterol	MET
Sitosterol	ADK
Sitosterol	RXRA
Sitosterol	CYP2C8
Sitosterol	TEK
Sitosterol	HNF4G
Sitosterol	GLO1
Sitosterol	F2
Sitosterol	LSS
Sitosterol	MMP3
Sitosterol	RARB
Sitosterol	PTPN1
Sitosterol	HSD11B1
Sitosterol	PGR
Sitosterol	PCTP
Sitosterol	HNMT
Sitosterol	EPHB4
Sitosterol	PPARD
Sitosterol	ESR2
Sitosterol	IL2
Sitosterol	AKR1B1

Sitosterol	FABP3
Sitosterol	SULT2A1
Sitosterol	CDK2
Sitosterol	PIK3R1
Sitosterol	GSTP1
Sitosterol	HSP90AA1
Sitosterol	NR3C1
Sitosterol	ABL1
Sitosterol	PDE4D
Sitosterol	LCK
Sitosterol	PLA2G2A
Sitosterol	PRKACA
Sitosterol	BACE1
Sitosterol	BRAF
Sitosterol	REN
Sitosterol	RARA
Sitosterol	CHEK1
Sitosterol	CALM1
Sitosterol	CALM2
Sitosterol	CALM3
Sitosterol	DPP4
Sitosterol	ADH
Sitosterol	NR1I2
Sitosterol	MMP2
Sitosterol	PTPN11
Sitosterol	SYK
Sitosterol	GART
Sitosterol	DHODH
Sitosterol	MMP12
Sitosterol	MMP13
Sitosterol	CTSB
Sitosterol	KIT
Sitosterol	HPGDS
Sitosterol	ESR1
Sitosterol	ADAM17
Sitosterol	MDM2
Sitosterol	AKR1C1
Sitosterol	WAS
Sitosterol	PDE4B
Sitosterol	BLVRB
Sitosterol	GC
Sitosterol	KIF11
Sitosterol	DPEP1
Sitosterol	HMGCR
Sitosterol	HSP90AB1
Sitosterol	LCN2
Sitosterol	EGFR
Sitosterol	SERPINA1

Sitosterol	SULT1E1
Sitosterol	CCNA2
Sitosterol	NR1I3
Sitosterol	ITGAL
Sitosterol	FNTA
Sitosterol	GSK3B
Sitosterol	TGFBR1
Sitosterol	PDPK1
Sitosterol	F10
Sitosterol	ZAP70
Sitosterol	ERBB4
Sitosterol	FKBP1A
Sitosterol	FGFR1
Sitosterol	ITK
Sitosterol	CYP2C9
Sitosterol	ERRA
Sitosterol	PRKCQ
Sitosterol	PPARA
Sitosterol	MAPKAPK2
Sitosterol	JAK3
Sitosterol	AKR1C3
Stachyose	UCK2
Stachyose	HRAS
Stachyose	GSK3B
Stachyose	APAF1
Stachyose	CHIT1
Stachyose	RAN
Stachyose	DCK
Stachyose	HPRT1
Stachyose	UAP1
Stachyose	LCK
Stachyose	MAPK10
Stachyose	HINT1
Stachyose	RAP2A
Stachyose	GNPDA1
Stachyose	GNPDA2
Stachyose	AURKA
Stachyose	WARS
Stachyose	SIRT5
Stachyose	DCPS
Stachyose	GSTZ1
Stachyose	DHFR
Stachyose	GSTP1
Stachyose	DAPK1
Stachyose	PIM1
Stachyose	ABO
Stachyose	CDK2
Stachyose	ARL5B

Stachyose	PCK1
Stachyose	SPR
Stachyose	DOT1L
Stachyose	NT5M
Stachyose	RFK
Stachyose	RAB5A
Stachyose	DTYMK
Stachyose	GMPR2
Stachyose	HNMT
Stachyose	SULT1A1
Stachyose	EEA1
Stachyose	PDE4B
Stachyose	ADK
Stachyose	CDC42
Stachyose	AKR1B1
Stachyose	MAPK12
Stachyose	PYGL
Stachyose	PKLR
Stachyose	ITPKA
Stachyose	OTC
Stachyose	F7
Stachyose	DUT
Stachyose	HADH
Stachyose	RAC1
Stachyose	EIF4E
Stachyose	TPH1
Stachyose	LGALS3
Stachyose	TAP1
Stachyose	BTK
Stachyose	RAB11A
Stachyose	IMPDH2
Stachyose	GMPR
Stachyose	CCL5
Stachyose	ELANE
Stachyose	AHCY
Stachyose	BACE1
Stachyose	AK1
Stachyose	GP1BA
Stachyose	ATIC
Stachyose	TGM3
Stachyose	BST1
Stachyose	HK1
Stachyose	AMY1A
Stachyose	AMY1B
Stachyose	AMY1C
Stachyose	TNK2
Stachyose	AKT1
Stachyose	MAN1B1

Stachyose	CD209
Stachyose	PDE4D
Stachyose	HSP90AA1
Stachyose	NMNAT1
Stachyose	RNASE2
Stachyose	LGALS2
Stachyose	AMY2A
Stachyose	PAPSS1
Stachyose	SULT2B1
Stachyose	GSTM2
Stachyose	PFKFB1
Stachyose	CLK1
Stachyose	F2
Stachyose	INSR
Stachyose	GART
Stachyose	SELE
Stachyose	RAB9A
Stachyose	RAB9B
Stachyose	KIF11
Stachyose	MME
Stachyose	KAT2B
Stachyose	NME2
Stachyose	ADAM33
Stachyose	AMD1
Stachyose	FKBP3
Stachyose	EPHA2
Stachyose	PAK6
Stachyose	CA2
Stachyose	PTPN1
Stachyose	ARL5A
Stachyose	PNMT
Stachyose	BCAT2
Stachyose	RNASE3
Stachyose	FDPS
Stachyose	ANG
Stachyose	LYZ
Stachyose	GSTA1
Stachyose	CTSK
Stachyose	MMP9
Stachyose	SDS
Stachyose	SULT2A1
Stachyose	SRC
Stachyose	IVD
Stachyose	NCS1
Stachyose	GSTT2
Stachyose	AKT2
Stachyose	SELP
Stachyose	PNP

Stachyose	KIT
Stachyose	B3GAT1
Stachyose	GSTM1
Stachyose	BMP7
Stachyose	MMP1
Stachyose	FGG
Stachyose	PLAU
Stachyose	PDE5A
Stachyose	ATOX1
Stachyose	SHMT1
Stachyose	HAGH
Stachyose	SRM
Stachyose	ARG2
Stachyose	GSTA3
Stachyose	CLEC4M
Stachyose	DDX39B
Stachyose	RAF1
Stachyose	LDHB
Stachyose	HSPA8
Stachyose	IMPDH1
Stachyose	MMP8
Stachyose	GPI
Stachyose	IMPA1
Stachyose	APRT
Stachyose	STAT1
Stachyose	CDA
Stachyose	IGF1R
Stachyose	ME2
Stachyose	PDPK1
Stachyose	HEXB
Stachyose	CANT1
Stachyose	CASP3
Stachyose	NOS2
Stachyose	ISG20
Stachyose	REG1A
Stachyose	MTAP
Stachyose	RHEB
Stachyose	RND3
Stachyose	ALDOA
Stachyose	PMS2
Stachyose	PAH
Stachyose	PTK2
Stachyose	CDK7
Stachyose	FKBP1A
Stachyose	GSR
Stachyose	BIRC7
Stachyose	ACAT1
Stachyose	RAC2

Stachyose	HCK
Stachyose	MMP3
Stachyose	CCNA2
Stigmasterol	RORA
Stigmasterol	TTR
Stigmasterol	CRABP2
Stigmasterol	MAOB
Stigmasterol	VDR
Stigmasterol	MAP2K1
Stigmasterol	RBP4
Stigmasterol	TTPA
Stigmasterol	PCTP
Stigmasterol	HSD17B1
Stigmasterol	KDR
Stigmasterol	PPP1CC
Stigmasterol	CYP2C8
Stigmasterol	ADK
Stigmasterol	DHODH
Stigmasterol	MET
Stigmasterol	RARG
Stigmasterol	PDPK1
Stigmasterol	PRKACA
Stigmasterol	PROCR
Stigmasterol	CA2
Stigmasterol	RARB
Stigmasterol	RXRB
Stigmasterol	HSD11B1
Stigmasterol	THRB
Stigmasterol	NR1H2
Stigmasterol	TEK
Stigmasterol	NR1I2
Stigmasterol	HNF4G
Stigmasterol	RXRA
Stigmasterol	HPGDS
Stigmasterol	CASP3
Stigmasterol	GSTP1
Stigmasterol	ESR1
Stigmasterol	CALM1
Stigmasterol	CALM2
Stigmasterol	CALM3
Stigmasterol	IL2
Stigmasterol	NR3C1
Stigmasterol	HNMT
Stigmasterol	SULT2B1
Stigmasterol	LSS
Stigmasterol	MMP2
Stigmasterol	SULT2A1
Stigmasterol	ALB

Stigmasterol	AKR1C2
Stigmasterol	MAPK1
Stigmasterol	NR1H3
Stigmasterol	METAP2
Stigmasterol	FABP6
Stigmasterol	NR1I3
Stigmasterol	GSTA1
Stigmasterol	BRAF
Stigmasterol	MAPK14
Stigmasterol	MMP13
Stigmasterol	FABP7
Stigmasterol	DPP4
Stigmasterol	SEC14L2
Stigmasterol	MAPK10
Stigmasterol	FECH
Stigmasterol	ESR2
Stigmasterol	BACE1
Stigmasterol	RARA
Stigmasterol	AR
Stigmasterol	TRAPPC3
Stigmasterol	PPARD
Stigmasterol	FGFR1
Stigmasterol	CHEK1
Stigmasterol	MMP3
Stigmasterol	BLVRB
Stigmasterol	GLO1
Stigmasterol	PLA2G2A
Stigmasterol	ITGAL
Stigmasterol	GC
Stigmasterol	KIF11
Stigmasterol	PTPN1
Stigmasterol	MDM2
Stigmasterol	PDE4B
Stigmasterol	AKR1B1
Stigmasterol	HDAC8
Stigmasterol	GRB2
Stigmasterol	CDK2
Stigmasterol	WAS
Stigmasterol	FABP3
Stigmasterol	LCK
Stigmasterol	NR1H4
Stigmasterol	F10
Stigmasterol	REN
Stigmasterol	HSP90AA1
Stigmasterol	SHBG
Stigmasterol	ERBB4
Stigmasterol	PPARG
Stigmasterol	NQO1

Stigmasterol	F2
Stigmasterol	KIT
Stigmasterol	PDE4D
Stigmasterol	PPARA
Stigmasterol	ADH
Stigmasterol	SYK
Stigmasterol	AKR1C3
Stigmasterol	FKBP1A
Stigmasterol	DPEP1
Stigmasterol	S100A9
Stigmasterol	PGR
Stigmasterol	ADAM17
Stigmasterol	TGFBR1
Stigmasterol	PTPN11
Stigmasterol	MMP12
Stigmasterol	HMGCR
Stigmasterol	ELANE
Stigmasterol	EGFR
Stigmasterol	TGM3
Stigmasterol	GART
Stigmasterol	PIK3R1
Stigmasterol	ERRA
Stigmasterol	ABO
Stigmasterol	HSP90AB1
Stigmasterol	CYP2C9
Stigmasterol	SRC
Stigmasterol	NR3C2
Stigmasterol	CCNA2
Stigmasterol	FNTA
Stigmasterol	CASP1
Stigmasterol	BCL2L1
Telocinobufagin	VDR
Telocinobufagin	SHBG
Telocinobufagin	RXRB
Telocinobufagin	HSD17B1
Telocinobufagin	MAP2K1
Telocinobufagin	BACE1
Telocinobufagin	F2
Telocinobufagin	HRAS
Telocinobufagin	TTPA
Telocinobufagin	CRABP2
Telocinobufagin	MAPK14
Telocinobufagin	RARG
Telocinobufagin	GSTA1
Telocinobufagin	SULT2B1
Telocinobufagin	FABP6
Telocinobufagin	FABP3
Telocinobufagin	GC

Telocinobufagin	CA2
Telocinobufagin	TTR
Telocinobufagin	FGFR1
Telocinobufagin	NR3C1
Telocinobufagin	AKR1C2
Telocinobufagin	NR1H4
Telocinobufagin	THRB
Telocinobufagin	PLA2G2A
Telocinobufagin	AR
Telocinobufagin	PTPN1
Telocinobufagin	RXRA
Telocinobufagin	WARS
Telocinobufagin	TGM3
Telocinobufagin	HSP90AA1
Telocinobufagin	ALB
Telocinobufagin	GSK3B
Telocinobufagin	FECH
Telocinobufagin	HSD11B1
Telocinobufagin	HNMT
Telocinobufagin	GSTP1
Telocinobufagin	AKR1C3
Telocinobufagin	ADK
Telocinobufagin	FKBP1A
Telocinobufagin	TGFBR1
Telocinobufagin	NR1I2
Telocinobufagin	GART
Telocinobufagin	PPP1CC
Telocinobufagin	METAP2
Telocinobufagin	PDE4D
Telocinobufagin	ADH1C
Telocinobufagin	PGR
Telocinobufagin	PROCR
Telocinobufagin	THRA
Telocinobufagin	FABP7
Telocinobufagin	CDK2
Telocinobufagin	MMP3
Telocinobufagin	RBP4
Telocinobufagin	TGFB2
Telocinobufagin	EPHA2
Telocinobufagin	MAOB
Telocinobufagin	MMP12
Telocinobufagin	CYP2C9
Telocinobufagin	RARB
Telocinobufagin	CTSK
Telocinobufagin	ESR1
Telocinobufagin	MET
Telocinobufagin	EGFR
Telocinobufagin	MTAP

Telocinobufagin	LCK
Telocinobufagin	DCK
Telocinobufagin	SULT2A1
Telocinobufagin	GSTA3
Telocinobufagin	DHFR
Telocinobufagin	PDE4B
Telocinobufagin	MME
Telocinobufagin	ABL1
Telocinobufagin	HMGCR
Telocinobufagin	GSTM1
Telocinobufagin	HDAC8
Telocinobufagin	DHODH
Telocinobufagin	ESR2
Telocinobufagin	SEC14L2
Telocinobufagin	PIK3R1
Telocinobufagin	PCTP
Telocinobufagin	REN
Telocinobufagin	RARA
Telocinobufagin	CCNA2
Telocinobufagin	ADH
Telocinobufagin	AKR1B1
Telocinobufagin	NR3C2
Telocinobufagin	GSTT2
Telocinobufagin	MMP13
Telocinobufagin	NR1H2
Telocinobufagin	GSR
Telocinobufagin	SOD2
Telocinobufagin	IL2
Telocinobufagin	PNMT
Telocinobufagin	KDR
Telocinobufagin	MAPK1
Telocinobufagin	DPP4
Telocinobufagin	ESRRG
Telocinobufagin	GCK
Telocinobufagin	KIF11
Telocinobufagin	INSR
Telocinobufagin	SERPINA1
Telocinobufagin	PIM1
Telocinobufagin	ELANE
Telocinobufagin	DTYMK
Telocinobufagin	RORA
Telocinobufagin	NR1I3
Telocinobufagin	ADAM17
Telocinobufagin	BHMT
Telocinobufagin	BIRC7
Telocinobufagin	PRKACA
Telocinobufagin	PPARG
Telocinobufagin	JAK3

Telocinobufagin	MAPKAPK2
Telocinobufagin	BRAF
Telocinobufagin	HNF4G
Telocinobufagin	PDPK1
Telocinobufagin	CTSF
Telocinobufagin	LSS
Telocinobufagin	CHEK1
Telocinobufagin	HPGDS
Telocinobufagin	EIF4E
Telocinobufagin	ADAM33
Telocinobufagin	PPARA
Telocinobufagin	CTSS
Telocinobufagin	CANT1
Telocinobufagin	DPEP1
Telocinobufagin	TYMS
Telocinobufagin	F10
Telocinobufagin	BST1
Telocinobufagin	EPHB4
Telocinobufagin	JAK2
Telocinobufagin	FNTA
Telocinobufagin	HSPA8
Tetrahydroalstonine	MAPK1
Tetrahydroalstonine	MMP12
Tetrahydroalstonine	FGFR1
Tetrahydroalstonine	PIM1
Tetrahydroalstonine	THRB
Tetrahydroalstonine	HNMT
Tetrahydroalstonine	HSD17B1
Tetrahydroalstonine	PDPK1
Tetrahydroalstonine	TTR
Tetrahydroalstonine	HSP90AA1
Tetrahydroalstonine	RARG
Tetrahydroalstonine	CRABP2
Tetrahydroalstonine	VDR
Tetrahydroalstonine	FKBP1A
Tetrahydroalstonine	BACE1
Tetrahydroalstonine	METAP2
Tetrahydroalstonine	RBP4
Tetrahydroalstonine	MAP2K1
Tetrahydroalstonine	PTPN1
Tetrahydroalstonine	CYP2C8
Tetrahydroalstonine	FNTA
Tetrahydroalstonine	PPP1CC
Tetrahydroalstonine	CHEK1
Tetrahydroalstonine	ALB
Tetrahydroalstonine	MAOB
Tetrahydroalstonine	SHBG
Tetrahydroalstonine	AKR1C3

Tetrahydroalstonine	CCNA2
Tetrahydroalstonine	TRAPPC3
Tetrahydroalstonine	GSTP1
Tetrahydroalstonine	TEK
Tetrahydroalstonine	FABP3
Tetrahydroalstonine	HMGCR
Tetrahydroalstonine	AR
Tetrahydroalstonine	PDE4B
Tetrahydroalstonine	CDK2
Tetrahydroalstonine	AKR1C2
Tetrahydroalstonine	ERRA
Tetrahydroalstonine	AKR1B1
Tetrahydroalstonine	HSD11B1
Tetrahydroalstonine	PDE4D
Tetrahydroalstonine	RXRA
Tetrahydroalstonine	PGR
Tetrahydroalstonine	MAPKAPK2
Tetrahydroalstonine	MAPK14
Tetrahydroalstonine	MTAP
Tetrahydroalstonine	NR1H2
Tetrahydroalstonine	PTPN11
Tetrahydroalstonine	ESR2
Tetrahydroalstonine	LCK
Tetrahydroalstonine	NR1H4
Tetrahydroalstonine	HPGDS
Tetrahydroalstonine	IL2
Tetrahydroalstonine	NR1H3
Tetrahydroalstonine	TTPA
Tetrahydroalstonine	GSK3B
Tetrahydroalstonine	SULT1E1
Tetrahydroalstonine	ITK
Tetrahydroalstonine	ADH
Tetrahydroalstonine	ABO
Tetrahydroalstonine	PPARD
Tetrahydroalstonine	JAK3
Tetrahydroalstonine	MMP3
Tetrahydroalstonine	MME
Tetrahydroalstonine	ITGAL
Tetrahydroalstonine	CALM1
Tetrahydroalstonine	CALM2
Tetrahydroalstonine	CALM3
Tetrahydroalstonine	PGF
Tetrahydroalstonine	BLVRB
Tetrahydroalstonine	ABL1
Tetrahydroalstonine	ADK
Tetrahydroalstonine	LTA4H
Tetrahydroalstonine	THRA
Tetrahydroalstonine	GSTA1

Tetrahydroalstonine	HDAC8
Tetrahydroalstonine	FABP6
Tetrahydroalstonine	PRKACA
Tetrahydroalstonine	ZAP70
Tetrahydroalstonine	NR1I2
Tetrahydroalstonine	SULT2B1
Tetrahydroalstonine	HCK
Tetrahydroalstonine	KIF11
Tetrahydroalstonine	SRC
Tetrahydroalstonine	NR3C1
Tetrahydroalstonine	ESR1
Tetrahydroalstonine	ESRRG
Tetrahydroalstonine	MET
Tetrahydroalstonine	TGFBR1
Tetrahydroalstonine	WAS
Tetrahydroalstonine	F2
Tetrahydroalstonine	SULT2A1
Tetrahydroalstonine	KDR
Tetrahydroalstonine	MAPK10
Tetrahydroalstonine	RORA
Tetrahydroalstonine	HSP90AB1
Tetrahydroalstonine	MAPK8
Tetrahydroalstonine	BRAF
Tetrahydroalstonine	CA2
Tetrahydroalstonine	SEC14L2
Tetrahydroalstonine	LSS
Tetrahydroalstonine	PRKCQ
Tetrahydroalstonine	CASP1
Tetrahydroalstonine	DHODH
Tetrahydroalstonine	PCTP
Tetrahydroalstonine	PARP1
Tetrahydroalstonine	PROCR
Tetrahydroalstonine	HNF4G
Tetrahydroalstonine	CTSK
Tetrahydroalstonine	ELANE
Tetrahydroalstonine	RXRB
Tetrahydroalstonine	PPARG
Tetrahydroalstonine	PPARA
Tetrahydroalstonine	FABP7
Tetrahydroalstonine	GC
Tetrahydroalstonine	CYP2C9
Tetrahydroalstonine	CTSB
Tetrahydroalstonine	PLA2G2A
Tetrahydroalstonine	NR3C2
Tetrahydroalstonine	MDM2
Tetrahydroalstonine	GM2A
Tetrahydroalstonine	FECH
Tetrahydroalstonine	REN

Tetrahydroalstonine	GRB2
Tetrahydroalstonine	GLO1
Trametenolic acid	HSD17B1
Trametenolic acid	TTPA
Trametenolic acid	RXRA
Trametenolic acid	RBP4
Trametenolic acid	NR3C1
Trametenolic acid	SULT2B1
Trametenolic acid	TTR
Trametenolic acid	CRABP2
Trametenolic acid	RXRB
Trametenolic acid	SULT1E1
Trametenolic acid	MAP2K1
Trametenolic acid	FABP7
Trametenolic acid	RARB
Trametenolic acid	FABP3
Trametenolic acid	NR1I2
Trametenolic acid	GSTA1
Trametenolic acid	HMGCR
Trametenolic acid	AR
Trametenolic acid	HSP90AA1
Trametenolic acid	PROCR
Trametenolic acid	PCTP
Trametenolic acid	RARG
Trametenolic acid	PRKCQ
Trametenolic acid	VDR
Trametenolic acid	CCNA2
Trametenolic acid	NR1I3
Trametenolic acid	GSTP1
Trametenolic acid	SHBG
Trametenolic acid	ZAP70
Trametenolic acid	CHEK1
Trametenolic acid	CDK2
Trametenolic acid	ESR1
Trametenolic acid	THRB
Trametenolic acid	ALB
Trametenolic acid	PDE4B
Trametenolic acid	SULT2A1
Trametenolic acid	THRA
Trametenolic acid	PPP1CC
Trametenolic acid	HSD11B1
Trametenolic acid	FABP6
Trametenolic acid	DPEP1
Trametenolic acid	LTA4H
Trametenolic acid	METAP2
Trametenolic acid	NR1H2
Trametenolic acid	MMP12
Trametenolic acid	PTPN11

Trametenolic acid	PIM1
Trametenolic acid	GSK3B
Trametenolic acid	MET
Trametenolic acid	HCK
Trametenolic acid	CTSB
Trametenolic acid	SORD
Trametenolic acid	MME
Trametenolic acid	PDE4D
Trametenolic acid	MAPK14
Trametenolic acid	MMP3
Trametenolic acid	PDPK1
Trametenolic acid	ERBB4
Trametenolic acid	HNMT
Trametenolic acid	LCN2
Trametenolic acid	KDR
Trametenolic acid	RARA
Trametenolic acid	F2
Trametenolic acid	FKBP1A
Trametenolic acid	PLA2G2A
Trametenolic acid	CA2
Trametenolic acid	HDAC8
Trametenolic acid	MAOB
Trametenolic acid	AKR1C3
Trametenolic acid	RORA
Trametenolic acid	BACE1
Trametenolic acid	SEC14L2
Trametenolic acid	ABO
Trametenolic acid	LCK
Trametenolic acid	PPARG
Trametenolic acid	ADK
Trametenolic acid	MMP13
Trametenolic acid	DPP4
Trametenolic acid	BRAF
Trametenolic acid	CALM1
Trametenolic acid	CALM2
Trametenolic acid	CALM3
Trametenolic acid	ITK
Trametenolic acid	IL2
Trametenolic acid	PGR
Trametenolic acid	MAPK10
Trametenolic acid	MAPKAPK2
Trametenolic acid	NR3C2
Trametenolic acid	GC
Trametenolic acid	SRC
Trametenolic acid	HPGDS
Trametenolic acid	FGFR1
Trametenolic acid	CASP3
Trametenolic acid	AKR1B1

Trametenolic acid	SYK
Trametenolic acid	JAK3
Trametenolic acid	DHODH
Trametenolic acid	S100A9
Trametenolic acid	TEK
Trametenolic acid	WAS
Trametenolic acid	PRKACA
Trametenolic acid	FNTA
Trametenolic acid	MTAP
Trametenolic acid	EIF4E
Trametenolic acid	PPARA
Trametenolic acid	ESR2
Trametenolic acid	FECH
Trametenolic acid	CSK
Trametenolic acid	ADH
Trametenolic acid	KIF11
Trametenolic acid	ESRRG
Trametenolic acid	MDM2
Trametenolic acid	MAPK8
Trametenolic acid	REN
Trametenolic acid	BIRC7
Trametenolic acid	ADAM17
Trametenolic acid	ITGAL
Trametenolic acid	MMP2
Trametenolic acid	PSAP
Trametenolic acid	NQO1
Trametenolic acid	GSR
ZINC02816192	AKR1B1
ZINC02816192	RFK
ZINC02816192	MAOB
ZINC02816192	PDPK1
ZINC02816192	HRAS
ZINC02816192	DUT
ZINC02816192	EIF4E
ZINC02816192	NQO2
ZINC02816192	INSR
ZINC02816192	PNMT
ZINC02816192	PTPN1
ZINC02816192	LTA4H
ZINC02816192	CHEK1
ZINC02816192	HSD11B1
ZINC02816192	VDR
ZINC02816192	MME
ZINC02816192	LSS
ZINC02816192	GSTP1
ZINC02816192	TK1
ZINC02816192	MMP3
ZINC02816192	EGFR

ZINC02816192	HCK
ZINC02816192	FGFR1
ZINC02816192	DAPK1
ZINC02816192	ARL5A
ZINC02816192	ESRRG
ZINC02816192	CDK2
ZINC02816192	FKBP3
ZINC02816192	ESR1
ZINC02816192	KDR
ZINC02816192	GSTM2
ZINC02816192	TTR
ZINC02816192	RAP2A
ZINC02816192	ARL5B
ZINC02816192	PDE4D
ZINC02816192	CCNA2
ZINC02816192	CTSK
ZINC02816192	UAP1
ZINC02816192	HSP90AA1
ZINC02816192	HSP90AB1
ZINC02816192	PRKACA
ZINC02816192	F10
ZINC02816192	HNMT
ZINC02816192	GSTA1
ZINC02816192	DTYMK
ZINC02816192	GSTT2
ZINC02816192	CTSS
ZINC02816192	ABO
ZINC02816192	HMGCR
ZINC02816192	HSD17B1
ZINC02816192	ITK
ZINC02816192	THRB
ZINC02816192	GMPR
ZINC02816192	HPGDS
ZINC02816192	ZAP70
ZINC02816192	PPP1CC
ZINC02816192	MTAP
ZINC02816192	MMP8
ZINC02816192	BACE1
ZINC02816192	AURKA
ZINC02816192	PRKCQ
ZINC02816192	FKBP1A
ZINC02816192	F2
ZINC02816192	HADH
ZINC02816192	AR
ZINC02816192	LCK
ZINC02816192	MET
ZINC02816192	MMP12
ZINC02816192	CRABP2

ZINC02816192	BST1
ZINC02816192	PARP1
ZINC02816192	SULT2B1
ZINC02816192	PLA2G2A
ZINC02816192	CHIT1
ZINC02816192	PDE4B
ZINC02816192	MAOA
ZINC02816192	BRAF
ZINC02816192	ABL1
ZINC02816192	WARS
ZINC02816192	HNF4G
ZINC02816192	FABP6
ZINC02816192	DCK
ZINC02816192	CA2
ZINC02816192	SHBG
ZINC02816192	CSK
ZINC02816192	CTSB
ZINC02816192	CBS
ZINC02816192	MAPK14
ZINC02816192	AKR1C3
ZINC02816192	AKR1C2
ZINC02816192	METAP2
ZINC02816192	DHFR
ZINC02816192	TGM3
ZINC02816192	AMD1
ZINC02816192	PGR
ZINC02816192	AMY2A
ZINC02816192	SRC
ZINC02816192	AKT1
ZINC02816192	HINT1
ZINC02816192	AKT2
ZINC02816192	HSPA8
ZINC02816192	GSK3B
ZINC02816192	GSR
ZINC02816192	TYMS
ZINC02816192	UCK2
ZINC02816192	IL2
ZINC02816192	TGFB2
ZINC02816192	CASP1
ZINC02816192	NOS3
ZINC02816192	BCAT2
ZINC02816192	CDA
ZINC02816192	ACAT1
ZINC02816192	CYP2C9
ZINC02816192	CDK7
ZINC02816192	PCK1
ZINC02816192	UMPS
ZINC02816192	DCXR

ZINC02816192	GSTM1
ZINC02816192	PIM1
ZINC02816192	LCN2
ZINC02816192	MAP2K1
ZINC02816192	DPP4
ZINC02816192	REN
ZINC02816192	NOS2
ZINC02816192	OTC
ZINC02816192	MAPK8
ZINC02816192	CTSF
ZINC02816192	ADAM33
ZINC02816192	DHODH
ZINC02816192	PDE3B
ZINC02816192	NNT
ZINC02816192	MAPK12
ZINC02816192	PAH
ZINC02816192	CANT1
