# Supplementary material for: Exploring the Pharmacological Mechanism of Liuwei Dihuang Decoction for Diabetic Retinopathy: A Systematic Biological Strategy-Based Research
Source: Evid Based Complement Alternat Med. 2021 Aug 2;2021:5544518. doi: 10.1155/2021/5544518 (PMC8356007; doi:10.1155/2021/5544518)
Supplement: Supplementary Materials — Table S1: compound targets for each compounds. Table S2: known targets for each compounds. Table S3: DR genes. Table S4: enrichment analysis of clusters based on Gene Ontology (GO) annotation of DR PPI network. Table S5: pathway enrichment analysis of DR PPI network. Table S6: enrichment analysis of clusters based on Gene Ontology (GO) annotation of LDD-DR PPI network. Table S7: pathway enrichment analysis of LDD-DR PPI network. Table S8: enrichment analysis of clusters based on Gene Ontology (GO) annotation of LDD known target-DR network. Table S9: pathway enrichment analysis of LDD known target-DR network. [file 5544518.f1.zip › 5544518.f1/Table S2 .pdf]

Table S2 Known targets for each compounds

Compounds	Compounds Targets
(-)-taxifolin	PTGS1
(-)-taxifolin	PTGS2
(-)-taxifolin	PIK3CG
(+)-catechin	PTGS1
(+)-catechin	ESR1
(+)-catechin	PTGS2
(+)-catechin	HSP90AA1
(+)-catechin	NCOA2
(+)-catechin	CALM1
(+)-catechin	CALM2
(+)-catechin	CALM3
(+)-catechin	RXRA
(+)-catechin	CAT
(+)-catechin	HAS2
Acteoside	ICAM1
AIDS180907	NOS2
AIDS180907	ESR1
AIDS180907	AR
AIDS180907	F10
AIDS180907	PTGS2
AIDS180907	F7
AIDS180907	TOP2A
AIDS180907	GSK3B
AIDS180907	PIM1
AIDS180907	CCNA2
AIDS180907	NCOA2
AIDS180907	NCOA1
AIDS180907	CALM1
AIDS180907	CALM2
AIDS180907	CALM3
Beta-sitosterol	PGR
Beta-sitosterol	NCOA2
Beta-sitosterol	NR3C2
Campesterol	PGR
Campesterol	PTGS1
Campesterol	PTGS2
Campesterol	PIK3CG
Campesterol	NCOA2
Catalpol	DPP4
Catalpol	BCL2
Catalpol	CASP3
Catalpol	SOD1
Cerevisterol	NR3C2
CLR	PGR
CLR	NR3C2
CLR	NCOA2

Cornudentanone	F2
Cornudentanone	PTGS2
Cornudentanone	NCOA2
Diop	SCN5A
Diop	ADRB2
Diop	CHRM3
Dioscoreside C	NR3C1
Dioscoreside C	NCOA2
Diosgenin	PGR
Diosgenin	NR3C2
Diosgenin	RELA
Diosgenin	AKT1
Diosgenin	VEGFA
Diosgenin	CDKN1A
Diosgenin	TP53
Diosgenin	PTGS2
Diosgenin	FASN
Diosgenin	SOD1
Diosgenin	CAT
Diosgenin	HIF1A
Diosgenin	NR1I2
Diosgenin	PLA2G4A
Diosgenin	ABCC2
Diosgenin	MTOR
Ergosta-7,22E-dien-3be	PGR
Ergosterol peroxide	PGR
Ethyl linolenate	PTGS1
Ethyl linolenate	NCOA2
Ethyl oleate (NF)	NCOA2
Hancinone C	NOS2
Hancinone C	CHRM3
Hancinone C	F2
Hancinone C	KCNH2
Hancinone C	CHRM1
Hancinone C	ESR1
Hancinone C	SCN5A
Hancinone C	F10
Hancinone C	PTGS2
Hancinone C	F7
Hancinone C	PDE3A
Hancinone C	ADRA1B
Hancinone C	ADRA1D
Hancinone C	TOP2A
Hancinone C	ESR2
Hancinone C	DPP4
Hancinone C	PRSS1
Hancinone C	NCOA2
Hancinone C	NCOA1

Hancinone C	KCNMA1
Hancinone C	CALM1
Hancinone C	CALM2
Hancinone C	CALM3
Hederagenin	PGR
Hederagenin	NCOA2
Hederagenin	CHRM3
Hederagenin	CHRM1
Hederagenin	GABRA2
Hederagenin	GABRA3
Hederagenin	CHRM2
Hederagenin	ADRA1B
Hederagenin	GABRA1
Hederagenin	GRIA2
Hederagenin	GABRA6
Hederagenin	GABRA5
Hederagenin	IGHG1
Hederagenin	ADH1B
Hederagenin	ADH1C
Hederagenin	PTGS1
Hederagenin	SCN5A
Hederagenin	PTGS2
Hederagenin	RXRA
Hederagenin	PDE3A
Hederagenin	SLC6A2
Hydroxygenkwanin	NOS2
Hydroxygenkwanin	PTGS1
Hydroxygenkwanin	PTGS2
Hydroxygenkwanin	DPP4
Hydroxygenkwanin	PRSS1
Hydroxygenkwanin	NCOA2
Hydroxygenkwanin	CALM1
Hydroxygenkwanin	CALM2
Hydroxygenkwanin	CALM3
Hydroxygenkwanin	PIK3CG
Isofucosterol	PGR
Isofucosterol	NR3C2
Isofucosterol	ABAT
Isofucosterol	GABRA1
Isofucosterol	ADH1B
Isofucosterol	ADH1C
Isofucosterol	ADH1A
Isofucosterol	NCOA2
Kadsurenone	PTGS1
Kadsurenone	CHRM3
Kadsurenone	F2
Kadsurenone	KCNH2
Kadsurenone	CHRM1

Kadsurenone	SCN5A
Kadsurenone	F10
Kadsurenone	CHRM5
Kadsurenone	PTGS2
Kadsurenone	CA2
Kadsurenone	ACHE
Kadsurenone	CHRM2
Kadsurenone	ADRA1B
Kadsurenone	ADRB2
Kadsurenone	ADRA1D
Kadsurenone	TOP2A
Kadsurenone	OPRM1
Kadsurenone	DPP4
Kadsurenone	PRSS1
Kadsurenone	NCOA2
Kadsurenone	NCOA1
Kadsurenone	KCNMA1
Kadsurenone	CALM1
Kadsurenone	CALM2
Kadsurenone	CALM3
Kadsurenone	RXRA
Kadsurenone	PDE3A
Kaempferol	NOS2
Kaempferol	PTGS1
Kaempferol	AR
Kaempferol	PPARG
Kaempferol	PTGS2
Kaempferol	HSP90AA1
Kaempferol	PIK3CG
Kaempferol	NCOA2
Kaempferol	DPP4
Kaempferol	PRSS1
Kaempferol	PGR
Kaempferol	F2
Kaempferol	CHRM1
Kaempferol	NOS3
Kaempferol	GABRA2
Kaempferol	ACHE
Kaempferol	SLC6A2
Kaempferol	CHRM2
Kaempferol	ADRA1B
Kaempferol	GABRA1
Kaempferol	TOP2A
Kaempferol	F7
Kaempferol	CALM1
Kaempferol	CALM2
Kaempferol	CALM3
Kaempferol	RELA

Kaempferol	IKBKB
Kaempferol	AKT1
Kaempferol	BCL2
Kaempferol	BAX
Kaempferol	TNF
Kaempferol	JUN
Kaempferol	AHSA1
Kaempferol	CASP3
Kaempferol	MAPK8
Kaempferol	XDH
Kaempferol	MMP1
Kaempferol	STAT1
Kaempferol	CDK1
Kaempferol	HMOX1
Kaempferol	CYP3A4
Kaempferol	CYP1A2
Kaempferol	CYP1A1
Kaempferol	ICAM1
Kaempferol	SELE
Kaempferol	VCAM1
Kaempferol	NR1I2
Kaempferol	CYP1B1
Kaempferol	ALOX5
Kaempferol	HAS2
Kaempferol	GSTP1
Kaempferol	AHR
Kaempferol	PSMD3
Kaempferol	SLC2A4
Kaempferol	NR1I3
Kaempferol	INSR
Kaempferol	DIO1
Kaempferol	PPP3CA
Kaempferol	GSTM1
Kaempferol	GSTM2
Kaempferol	AKR1C3
Kaempferol	SLPI
Mairin	PGR
Mandenol	PTGS1
Mandenol	PTGS2
Mandenol	NCOA2
MOL000273	NR3C2
MOL000273	NCOA2
MOL005435	PGR
MOL005481	PTGS2
Piperlonguminine	CHRM1
Piperlonguminine	NOS3
Piperlonguminine	RXRA
Piperlonguminine	PDE3A

Piperlonguminine	ADRA1B
Piperlonguminine	SLC6A3
Piperlonguminine	ADRB2
Piperlonguminine	SLC6A4
Piperlonguminine	LTA4H
Piperlonguminine	MAOB
Poriferast-5-en-3beta-o	PGR
Poriferast-5-en-3beta-o	NCOA2
Quercetin	PTGS1
Quercetin	AR
Quercetin	PPARG
Quercetin	PTGS2
Quercetin	HSP90AA1
Quercetin	PIK3CG
Quercetin	NCOA2
Quercetin	DPP4
Quercetin	AKR1B1
Quercetin	PRSS1
Quercetin	TOP2A
Quercetin	F2
Quercetin	KCNH2
Quercetin	SCN5A
Quercetin	F10
Quercetin	ADRB2
Quercetin	MMP3
Quercetin	F7
Quercetin	NOS3
Quercetin	RXRA
Quercetin	ACHE
Quercetin	GABRA1
Quercetin	MAOB
Quercetin	RELA
Quercetin	EGFR
Quercetin	AKT1
Quercetin	VEGFA
Quercetin	CCND1
Quercetin	BCL2
Quercetin	BCL2L1
Quercetin	FOS
Quercetin	CDKN1A
Quercetin	EIF6
Quercetin	BAX
Quercetin	CASP9
Quercetin	PLAU
Quercetin	MMP2
Quercetin	MAPK1
Quercetin	IL10
Quercetin	EGF

Quercetin	RB1
Quercetin	TNF
Quercetin	JUN
Quercetin	IL6
Quercetin	AHSA1
Quercetin	CASP3
Quercetin	TP53
Quercetin	ELK1
Quercetin	NFKBIA
Quercetin	POR
Quercetin	ODC1
Quercetin	XDH
Quercetin	CASP8
Quercetin	TOP1
Quercetin	RAF1
Quercetin	SOD1
Quercetin	PRKCA
Quercetin	MMP1
Quercetin	HIF1A
Quercetin	STAT1
Quercetin	RUNX1T1
Quercetin	CDK1
Quercetin	HSPA5
Quercetin	ERBB2
Quercetin	ACACA
Quercetin	HMOX1
Quercetin	CYP3A4
Quercetin	CYP1A2
Quercetin	CAV1
Quercetin	MYC
Quercetin	F3
Quercetin	GJA1
Quercetin	CYP1A1
Quercetin	ICAM1
Quercetin	IL1B
Quercetin	CCL2
Quercetin	SELE
Quercetin	VCAM1
Quercetin	PTGER3
Quercetin	IL8
Quercetin	PRKCB
Quercetin	BIRC5
Quercetin	DUOX2
Quercetin	HSPB1
Quercetin	TGFB1
Quercetin	SULT1E1
Quercetin	MGAM
Quercetin	IL2

Quercetin	NR1I2
Quercetin	CYP1B1
Quercetin	CCNB1
Quercetin	PLAT
Quercetin	THBD
Quercetin	SERPINE1
Quercetin	COL1A1
Quercetin	IFNG
Quercetin	ALOX5
Quercetin	PTEN
Quercetin	IL1A
Quercetin	MPO
Quercetin	NCF1
Quercetin	ABCG2
Quercetin	HAS2
Quercetin	GSTP1
Quercetin	NFE2L2
Quercetin	NQO1
Quercetin	PARP1
Quercetin	AHR
Quercetin	PSMD3
Quercetin	SLC2A4
Quercetin	COL3A1
Quercetin	CXCL11
Quercetin	CXCL2
Quercetin	DCAF5
Quercetin	NR1I3
Quercetin	CHEK2
Quercetin	INSR
Quercetin	CLDN4
Quercetin	PPARA
Quercetin	PPARD
Quercetin	HSF1
Quercetin	CRP
Quercetin	CXCL10
Quercetin	CHUK
Quercetin	SPP1
Quercetin	RUNX2
Quercetin	RASSF1
Quercetin	E2F1
Quercetin	E2F2
Quercetin	ACPP
Quercetin	CTSD
Quercetin	IGFBP3
Quercetin	IGF2
Quercetin	CD40LG
Quercetin	IRF1
Quercetin	ERBB3

Quercetin	PON1
Quercetin	DIO1
Quercetin	PCOLCE
Quercetin	NPEPPS
Quercetin	HK2
Quercetin	NKX3-1
Quercetin	RASA1
Quercetin	GSTM1
Quercetin	GSTM2
Sitosterol	PGR
Sitosterol	NCOA2
Sitosterol	NR3C2
Stachyose	PTGS1
Stachyose	PTGS2
Stachyose	GABRA1
Stachyose	MAN2A1
Stigmasterol	PGR
Stigmasterol	NR3C2
Stigmasterol	NCOA2
Stigmasterol	ADH1C
Stigmasterol	IGHG1
Stigmasterol	RXRA
Stigmasterol	NCOA1
Stigmasterol	PTGS1
Stigmasterol	PTGS2
Stigmasterol	ADRA2A
Stigmasterol	SLC6A2
Stigmasterol	SLC6A3
Stigmasterol	ADRB2
Stigmasterol	AKR1B1
Stigmasterol	PLAU
Stigmasterol	LTA4H
Stigmasterol	MAOB
Stigmasterol	MAOA
Stigmasterol	CTRB1
Stigmasterol	CHRM3
Stigmasterol	CHRM1
Stigmasterol	ADRB1
Stigmasterol	SCN5A
Stigmasterol	HTR2A
Stigmasterol	ADRA1A
Stigmasterol	GABRA3
Stigmasterol	CHRM2
Stigmasterol	ADRA1B
Stigmasterol	GABRA1
Stigmasterol	CHRNA7
Telocinobufagin	NR3C2
Telocinobufagin	NR3C1

Telocinobufagin	NOS2
Telocinobufagin	PTGS1
Telocinobufagin	DRD1
Telocinobufagin	CHRM3
Telocinobufagin	F2
Telocinobufagin	KCNH2
Telocinobufagin	CHRM1
Telocinobufagin	AR
Telocinobufagin	SCN5A
Telocinobufagin	PPARG
Telocinobufagin	F10
Telocinobufagin	CHRM5
Tetrahydroalstonine	PTGS2
Tetrahydroalstonine	ADRA2C
Tetrahydroalstonine	CHRM4
Tetrahydroalstonine	OPRD1
Tetrahydroalstonine	ACHE
Tetrahydroalstonine	HTR2A
Tetrahydroalstonine	ADRA1B
Tetrahydroalstonine	ADRB2
Tetrahydroalstonine	ADRA1D
Tetrahydroalstonine	SLC6A4
Tetrahydroalstonine	OPRM1
Tetrahydroalstonine	DPP4
Tetrahydroalstonine	PRSS1
Tetrahydroalstonine	CALM1
Tetrahydroalstonine	CALM2
Tetrahydroalstonine	CALM3
Trametenolic acid	NR3C2
ZINC02816192	ESR1
ZINC02816192	GSK3B
ZINC02816192	CDK2
