# Supplementary material for: Exploring the Pharmacological Mechanism of Liuwei Dihuang Decoction for Diabetic Retinopathy: A Systematic Biological Strategy-Based Research
Source: Evid Based Complement Alternat Med. 2021 Aug 2;2021:5544518. doi: 10.1155/2021/5544518 (PMC8356007; doi:10.1155/2021/5544518)
Supplement: Supplementary Materials — Table S1: compound targets for each compounds. Table S2: known targets for each compounds. Table S3: DR genes. Table S4: enrichment analysis of clusters based on Gene Ontology (GO) annotation of DR PPI network. Table S5: pathway enrichment analysis of DR PPI network. Table S6: enrichment analysis of clusters based on Gene Ontology (GO) annotation of LDD-DR PPI network. Table S7: pathway enrichment analysis of LDD-DR PPI network. Table S8: enrichment analysis of clusters based on Gene Ontology (GO) annotation of LDD known target-DR network. Table S9: pathway enrichment analysis of LDD known target-DR network. [file 5544518.f1.zip › 5544518.f1/Table S3.pdf]

Table S3 DR Ggenes

Gene Symbol	Description	Relevance score
VEGFA	Vascular Endothelial Growth Fa	60.4
ACE	Angiotensin I Converting Enzym	36.58
PON1	Paraoxonase 1	32.31
EPO	Erythropoietin	30.32
AKR1B1	Aldo-Keto Reductase Family 1 M	26.82
SERPINF1	Serpin Family F Member 1	23.91
SOD2	Superoxide Dismutase 2	23.6
AGER	Advanced Glycosylation End-Pr	22.59
ICAM1	Intercellular Adhesion Molecule	21.98
HFE	Homeostatic Iron Regulator	21.62
ALB	Albumin	20.92
IGF1	Insulin Like Growth Factor 1	20.9
CCL2	C-C Motif Chemokine Ligand 2	20.66
INS	Insulin	20.61
ANGPT2	Angiopoietin 2	19.5
FGF2	Fibroblast Growth Factor 2	19.2
NOS3	Nitric Oxide Synthase 3	19.15
VCAM1	Vascular Cell Adhesion Molecu	18.55
IL1RN	Interleukin 1 Receptor Antagoni	17.61
SERPINE1	Serpin Family E Member 1	17.05
HGF	Hepatocyte Growth Factor	16.08
PRKCB	Protein Kinase C Beta	16.05
FLT1	Fms Related Tyrosine Kinase 1	15.86
CTGF	Connective Tissue Growth Facto	15.39
CXCL12	C-X-C Motif Chemokine Ligand	15.35
MMP9	Matrix Metallopeptidase 9	14.59
CXCL8	C-X-C Motif Chemokine Ligand	14.47
SST	Somatostatin	14.45
CAPN5	Calpain 5	14.03
KDR	Kinase Insert Domain Receptor	13.67
MIR21	MicroRNA 21	13.42
AOC3	Amine Oxidase, Copper Contain	13.18
SORD	Sorbitol Dehydrogenase	13.16
TNF	Tumor Necrosis Factor	12.63
TEK	TEK Receptor Tyrosine Kinase	12.49
PGF	Placental Growth Factor	12.03
TGFB2	Transforming Growth Factor Be	12.01
TGFB1	Transforming Growth Factor Be	11.63
AGT	Angiotensinogen	11.46
PLXDC2	Plexin Domain Containing 2	10.95
AGTR1	Angiotensin II Receptor Type 1	10.91
CHN2	Chimerin 2	10.67
BMP6	Bone Morphogenetic Protein 6	10.09
EDN1	Endothelin 1	10.08
MIR192	MicroRNA 192	10.05
ADM	Adrenomedullin	9.97

COLEC12	Collectin Subfamily Member 12	9.91
MTHFR	Methylenetetrahydrofolate Redu	9.67
PPARG	Peroxisome Proliferator Activate	9.65
MIR377	MicroRNA 377	9.62
IL6	Interleukin 6	9.49
REN	Renin	9.01
MIAT	Myocardial Infarction Associate	8.86
GFAP	Glial Fibrillary Acidic Protein	8.46
VTN	Vitronectin	8.25
SEMA3E	Semaphorin 3E	7.67
ENSG00000285441		7.63
TIMP1	TIMP Metallopeptidase Inhibito	7.58
FGF1	Fibroblast Growth Factor 1	7.45
SPARC	Secreted Protein Acidic And Cy	7.4
IGFBP3	Insulin Like Growth Factor Binc	7.32
OPN4	Opsin 4	7.3
VDR	Vitamin D Receptor	6.81
TLR9	Toll Like Receptor 9	6.8
KCNH3	Potassium Voltage-Gated Chann	6.8
ITIH2	Inter-Alpha-Trypsin Inhibitor He	6.8
MALAT1	Metastasis Associated Lung Ade	6.8
NAGLU	N-Acetyl-Alpha-Glucosaminidas	6.74
ITGA2	Integrin Subunit Alpha 2	6.66
SOD1	Superoxide Dismutase 1	6.64
TCF7L2	Transcription Factor 7 Like 2	6.56
SELE	Selectin E	6.36
CRP	C-Reactive Protein	6.16
NAA15	N(Alpha)-Acetyltransferase 15,	6.09
ADIPOQ	Adiponectin, C1Q And Collager	6.05
PLXDC1	Plexin Domain Containing 1	5.97
BDNF	Brain Derived Neurotrophic Fac	5.84
HMGB1	High Mobility Group Box 1	5.83
NOS2	Nitric Oxide Synthase 2	5.8
APOE	Apolipoprotein E	5.79
VWF	Von Willebrand Factor	5.73
HIF1A	Hypoxia Inducible Factor 1 Sub	5.7
LTA	Lymphotoxin Alpha	5.61
PVT1	Pvt1 Oncogene	5.48
CCNL1	Cyclin L1	5.43
IGSF21	Immunoglobin Superfamily Mer	5.43
LEKR1	Leucine, Glutamate And Lysine	5.43
KLHDC7A	Kelch Domain Containing 7A	5.43
HLA-DQB1	Major Histocompatibility Comp	5.43
INSR	Insulin Receptor	5.38
TLR4	Toll Like Receptor 4	5.38
IL17A	Interleukin 17A	5.28
SELP	Selectin P	5.23
SLC2A1	Solute Carrier Family 2 Member	5.18

ARHGAP22	Rho GTPase Activating Protein	5.12
CXCL10	C-X-C Motif Chemokine Ligand	5.08
NPY	Neuropeptide Y	5.07
ANGPT1	Angiopoietin 1	4.88
NPHS1	NPHS1, Nephrin	4.81
CNDP1	Carnosine Dipeptidase 1	4.81
STK38L	Serine/Threonine Kinase 38 Lik	4.81
MIR216A	MicroRNA 216a	4.81
MIR217	MicroRNA 217	4.81
MEG8	Maternally Expressed 8, Small N	4.81
SNHG6	Small Nucleolar RNA Host Gen	4.81
SNHG18	Small Nucleolar RNA Host Gen	4.81
IL10	Interleukin 10	4.8
TIMP3	TIMP Metallopeptidase Inhibito	4.75
IGFBP1	Insulin Like Growth Factor Binc	4.7
CYR61	Cysteine Rich Angiogenic Induc	4.6
MIR200B	MicroRNA 200b	4.58
TAB2	TGF-Beta Activated Kinase 1 (M	4.52
TCF4	Transcription Factor 4	4.52
KCNK1	Potassium Two Pore Domain Ch	4.52
CREB5	CAMP Responsive Element Bin	4.52
RBFOX1	RNA Binding Fox-1 Homolog 1	4.52
MPRIP	Myosin Phosphatase Rho Interac	4.52
VSTM2B	V-Set And Transmembrane Don	4.52
LINC00917	Long Intergenic Non-Protein Co	4.52
LOC339529	Uncharacterized LOC339529	4.52
KRT18P34	Keratin 18 Pseudogene 34	4.52
LOC100506023	Uncharacterized LOC10050602	4.52
LOC729200	Small Nuclear Ribonucleoprotei	4.52
LOC101928236	Uncharacterized LOC10192823	4.52
ADRB3	Adrenoceptor Beta 3	4.51
MMP2	Matrix Metallopeptidase 2	4.49
JUN	Jun Proto-Oncogene, AP-1 Tran	4.49
UCP2	Uncoupling Protein 2	4.49
APLN	Apelin	4.49
THBS1	Thrombospondin 1	4.47
IFNG	Interferon Gamma	4.37
NFKB1	Nuclear Factor Kappa B Subunit	4.35
APLNR	Apelin Receptor	4.31
ANGPTL4	Angiopoietin Like 4	4.31
IL1R1	Interleukin 1 Receptor Type 1	4.31
HLA-DRB1	Major Histocompatibility Comp	4.3
HS6ST3	Heparan Sulfate 6-O-Sulfotransf	4.27
TIMP2	TIMP Metallopeptidase Inhibito	4.21
HP	Haptoglobin	4.21
IL1B	Interleukin 1 Beta	4.21
MAPK8	Mitogen-Activated Protein Kina	4.2
PLAT	Plasminogen Activator, Tissue T	4.2

IGF2	Insulin Like Growth Factor 2	4.17
PF4	Platelet Factor 4	4.16
UCHL3	Ubiquitin C-Terminal Hydrolase	4.14
MYSM1	Myb Like, SWIRM And MPN D	4.14
CNR1	Cannabinoid Receptor 1	4.08
TNFRSF11B	TNF Receptor Superfamily Men	4.04
FNDC5	Fibronectin Type III Domain Co	4.03
ROBO1	Roundabout Guidance Receptor	3.98
PON2	Paraoxonase 2	3.98
THBD	Thrombomodulin	3.93
GHR	Growth Hormone Receptor	3.93
GSTM1	Glutathione S-Transferase Mu 1	3.93
PTGS2	Prostaglandin-Endoperoxide Syn	3.91
ITGB3	Integrin Subunit Beta 3	3.87
PPARGC1A	PPARG Coactivator 1 Alpha	3.87
CYBB	Cytochrome B-245 Beta Chain	3.87
LOX	Lysyl Oxidase	3.87
GSTT1	Glutathione S-Transferase Theta	3.87
NGF	Nerve Growth Factor	3.83
ALDH2	Aldehyde Dehydrogenase 2 Fam	3.83
SDHB	Succinate Dehydrogenase Comp	3.77
SLIT2	Slit Guidance Ligand 2	3.77
FGF21	Fibroblast Growth Factor 21	3.77
ADAMTSL1	ADAMTS Like 1	3.74
RBP4	Retinol Binding Protein 4	3.73
F3	Coagulation Factor III, Tissue Fa	3.73
RHO	Rhodopsin	3.73
POSTN	Periostin	3.73
NID1	Nidogen 1	3.73
AKT3	AKT Serine/Threonine Kinase 3	3.71
CTSD	Cathepsin D	3.71
PTX3	Pentraxin 3	3.65
TTR	Transthyretin	3.65
HMGCR	3-Hydroxy-3-Methylglutaryl-Co	3.64
AKR1B10	Aldo-Keto Reductase Family 1 M	3.64
STAT3	Signal Transducer And Activato	3.59
CD40LG	CD40 Ligand	3.59
UTS2	Urotensin 2	3.59
HLA-DQA1	Major Histocompatibility Compl	3.55
EGF	Epidermal Growth Factor	3.55
SIRT1	Sirtuin 1	3.52
CCL5	C-C Motif Chemokine Ligand 5	3.51
BAX	BCL2 Associated X, Apoptosis R	3.46
ITGB2	Integrin Subunit Beta 2	3.44
HNF1A	HNF1 Homeobox A	3.4
CDH5	Cadherin 5	3.35
TJP1	Tight Junction Protein 1	3.35
PLG	Plasminogen	3.34

IL1A	Interleukin 1 Alpha	3.34
ELN	Elastin	3.34
IFNA2	Interferon Alpha 2	3.34
CXCL9	C-X-C Motif Chemokine Ligand	3.34
TF	Transferrin	3.33
IGF1R	Insulin Like Growth Factor 1 Re	3.29
ANG	Angiogenin	3.23
PIK3CG	Phosphatidylinositol-4,5-Bispho	3.19
MIF	Macrophage Migration Inhibitor	3.19
KITLG	KIT Ligand	3.15
ROBO4	Roundabout Guidance Receptor	3.15
NRP1	Neuropilin 1	3.14
PTGDS	Prostaglandin D2 Synthase	3.14
CCL4	C-C Motif Chemokine Ligand 4	3.14
PSMD9	Proteasome 26S Subunit, Non-A	3.11
ZNRF1	Zinc And Ring Finger 1	3.11
GORAB	Golgin, RAB6 Interacting	3.11
API5	Apoptosis Inhibitor 5	3.11
CCL3	C-C Motif Chemokine Ligand 3	3.08
PLVAP	Plasmalemma Vesicle Associate	3.07
PRDX1	Peroxiredoxin 1	3.06
SERPINI1	Serpin Family I Member 1	3.06
CFI	Complement Factor I	3.06
CLSTN1	Calsyntenin 1	3.06
ST3GAL4	ST3 Beta-Galactoside Alpha-2,3	3.03
AR	Androgen Receptor	3.01
GJA1	Gap Junction Protein Alpha 1	3.01
CASP1	Caspase 1	3.01
FN1	Fibronectin 1	3.01
GH1	Growth Hormone 1	3.01
CA1	Carbonic Anhydrase 1	3.01
SETD2	SET Domain Containing 2	3.01
CHI3L1	Chitinase 3 Like 1	3.01
ITGA2B	Integrin Subunit Alpha 2b	2.97
CPVL	Carboxypeptidase, Vitellogenic	2.97
FLT4	Fms Related Tyrosine Kinase 4	2.94
EDN3	Endothelin 3	2.94
SELL	Selectin L	2.94
MIR146A	MicroRNA 146a	2.94
LOC111365141	NOS2 5' Regulatory Region	2.94
PPARA	Peroxisome Proliferator Activat	2.92
KLKB1	Kallikrein B1	2.86
NPPA	Natriuretic Peptide A	2.86
VEGFC	Vascular Endothelial Growth Fa	2.86
CASP14	Caspase 14	2.86
RPE65	RPE65, Retinoid Isomerohydrol	2.86
CSF1	Colony Stimulating Factor 1	2.86
PIGF	Phosphatidylinositol Glycan An	2.86

MIR195	MicroRNA 195	2.86
PRL	Prolactin	2.85
AKT1	AKT Serine/Threonine Kinase 1	2.8
SP1	Sp1 Transcription Factor	2.8
APOA4	Apolipoprotein A4	2.8
RXRG	Retinoid X Receptor Gamma	2.8
SPTBN5	Spectrin Beta, Non-Erythrocytic	2.8
MMP14	Matrix Metallopeptidase 14	2.76
MMP1	Matrix Metallopeptidase 1	2.76
TLR2	Toll Like Receptor 2	2.76
VIM	Vimentin	2.76
EPOR	Erythropoietin Receptor	2.76
F2	Coagulation Factor II, Thrombin	2.76
GNB3	G Protein Subunit Beta 3	2.76
LRP6	LDL Receptor Related Protein 6	2.76
TKT	Transketolase	2.76
UCP1	Uncoupling Protein 1	2.76
AASS	Aminoadipate-Semialdehyde Sy	2.76
KNG1	Kininogen 1	2.76
SHBG	Sex Hormone Binding Globulin	2.76
TMSB4X	Thymosin Beta 4 X-Linked	2.76
CORT	Cortistatin	2.76
GRB2	Growth Factor Receptor Bound	2.74
CASP9	Caspase 9	2.74
CD59	CD59 Molecule (CD59 Blood G	2.74
TNFRSF13B	TNF Receptor Superfamily Men	2.74
IRF8	Interferon Regulatory Factor 8	2.74
TNFSF4	TNF Superfamily Member 4	2.74
MYT1L	Myelin Transcription Factor 1 L	2.74
PECAM1	Platelet And Endothelial Cell Ad	2.74
MRPS15	Mitochondrial Ribosomal Protei	2.74
OSCP1	Organic Solute Carrier Partner 1	2.74
CCDC68	Coiled-Coil Domain Containing	2.74
VEPH1	Ventricular Zone Expressed PH	2.74
TVP23B	Trans-Golgi Network Vesicle Pr	2.74
GRAMD2B	GRAM Domain Containing 2B	2.74
MAP3K21	Mitogen-Activated Protein Kina	2.74
MIR126	MicroRNA 126	2.74
LOC100131080	Ribosomal Protein S14 Pseudog	2.74
PRKCD	Protein Kinase C Delta	2.66
RAC1	Rac Family Small GTPase 1	2.66
MPO	Myeloperoxidase	2.66
FASN	Fatty Acid Synthase	2.66
ITGA4	Integrin Subunit Alpha 4	2.66
CD79A	CD79a Molecule	2.66
NFE2L2	Nuclear Factor, Erythroid 2 Like	2.66
ROCK2	Rho Associated Coiled-Coil Cor	2.66
ENG	Endoglin	2.66

ATP6AP2	ATPase H+ Transporting Access	2.66
COL11A2	Collagen Type XI Alpha 2 Chain	2.66
KLK1	Kallikrein 1	2.66
RHD	Rh Blood Group D Antigen	2.66
EDN2	Endothelin 2	2.66
HIST1H1C	Histone Cluster 1 H1 Family Me	2.66
PTPN1	Protein Tyrosine Phosphatase, N	2.64
GCK	Glucokinase	2.64
TNFRSF1A	TNF Receptor Superfamily Men	2.64
IL6R	Interleukin 6 Receptor	2.64
TNC	Tenascin C	2.64
ITGAM	Integrin Subunit Alpha M	2.64
HLA-B	Major Histocompatibility Comp	2.64
BSG	Basigin (Ok Blood Group)	2.64
ADA2	Adenosine Deaminase 2	2.64
MBL2	Mannose Binding Lectin 2	2.58
CTSH	Cathepsin H	2.58
CARM1	Coactivator Associated Arginine	2.58
HPSE	Heparanase	2.58
IL13	Interleukin 13	2.58
TIAM1	T Cell Lymphoma Invasion And	2.58
HTRA1	HtrA Serine Peptidase 1	2.58
FBLN1	Fibulin 1	2.58
ENPP2	Ectonucleotide Pyrophosphatase	2.58
OSM	Oncostatin M	2.58
BGLAP	Bone Gamma-Carboxyglutamate	2.58
CXCL5	C-X-C Motif Chemokine Ligand	2.58
SDS	Serine Dehydratase	2.58
B4GALT2	Beta-1,4-Galactosyltransferase 2	2.58
CCL1	C-C Motif Chemokine Ligand 1	2.58
TNFSF12	TNF Superfamily Member 12	2.58
SUMO4	Small Ubiquitin-Like Modifier 4	2.58
MIR27B	MicroRNA 27b	2.58
MIR320A	MicroRNA 320a	2.58
MET	MET Proto-Oncogene, Receptor	2.49
EGFR	Epidermal Growth Factor Recep	2.49
RARA	Retinoic Acid Receptor Alpha	2.49
MMP3	Matrix Metallopeptidase 3	2.49
GDNF	Glial Cell Derived Neurotrophic	2.49
C5	Complement C5	2.49
KCNJ11	Potassium Voltage-Gated Chann	2.49
NAMPT	Nicotinamide Phosphoribosyltra	2.49
TGFA	Transforming Growth Factor Al	2.49
PDCD1	Programmed Cell Death 1	2.49
HMGA1	High Mobility Group AT-Hook	2.49
OCLN	Occludin	2.49
AGTR2	Angiotensin II Receptor Type 2	2.49
NPY2R	Neuropeptide Y Receptor Y2	2.49

SSTR3	Somatostatin Receptor 3	2.49
ALOX12	Arachidonate 12-Lipoxygenase,	2.49
AGK	Acylglycerol Kinase	2.49
SERPINA4	Serpin Family A Member 4	2.49
RBP3	Retinol Binding Protein 3	2.49
C1QTNF3	C1q And TNF Related 3	2.49
MMRN1	Multimerin 1	2.49
TXNIP	Thioredoxin Interacting Protein	2.49
PAFAH2	Platelet Activating Factor Acety	2.49
TUBD1	Tubulin Delta 1	2.49
NUDT6	Nudix Hydrolase 6	2.49
CDKN2B-AS1	CDKN2B Antisense RNA 1	2.49
MIR20B	MicroRNA 20b	2.49
LOC109113863	FGF2 Promoter Region	2.49
RETN	Resistin	2.45
ATM	ATM Serine/Threonine Kinase	2.36
GAD1	Glutamate Decarboxylase 1	2.36
MME	Membrane Metalloendopeptidas	2.36
EDNRA	Endothelin Receptor Type A	2.36
EDNRB	Endothelin Receptor Type B	2.36
G6PD	Glucose-6-Phosphate Dehydroge	2.36
GLUL	Glutamate-Ammonia Ligase	2.36
ITGB1	Integrin Subunit Beta 1	2.36
HLA-A	Major Histocompatibility Comp	2.36
GRIK2	Glutamate Ionotropic Receptor I	2.36
CYP2C19	Cytochrome P450 Family 2 Sub	2.36
PML	Promyelocytic Leukemia	2.36
IL2	Interleukin 2	2.36
ADORA2A	Adenosine A2a Receptor	2.36
IL6ST	Interleukin 6 Signal Transducer	2.36
THBS2	Thrombospondin 2	2.36
CXCR3	C-X-C Motif Chemokine Recept	2.36
LBP	Lipopolysaccharide Binding Pro	2.36
S100A4	S100 Calcium Binding Protein A	2.36
ECM1	Extracellular Matrix Protein 1	2.36
TBC1D4	TBC1 Domain Family Member 4	2.36
SORL1	Sortilin Related Receptor 1	2.36
NTN1	Netrin 1	2.36
CD163	CD163 Molecule	2.36
FCN3	Ficolin 3	2.36
ST6GAL1	ST6 Beta-Galactoside Alpha-2,6	2.36
LPA	Lipoprotein(A)	2.36
RS1	Retinoschisin 1	2.36
GCNT1	Glucosaminyl (N-Acetyl) Transf	2.36
AZU1	Azurocidin 1	2.36
TBX18	T-Box 18	2.36
SOD3	Superoxide Dismutase 3	2.36
ESM1	Endothelial Cell Specific Molec	2.36

HLTF	Helicase Like Transcription Fact	2.36
NUTF2	Nuclear Transport Factor 2	2.36
SLMAP	Sarcolemma Associated Protein	2.36
LECT2	Leukocyte Cell Derived Chemot	2.36
MOK	MOK Protein Kinase	2.36
CNKSR3	CNKSR Family Member 3	2.36
COMMD6	COMM Domain Containing 6	2.36
ROMO1	Reactive Oxygen Species Modul	2.36
SCAF8	SR-Related CTD Associated Fac	2.36
KIAA0825	KIAA0825	2.36
CEP162	Centrosomal Protein 162	2.36
ANGPTL8	Angiopoietin Like 8	2.36
LINC01611	Long Intergenic Non-Protein Co	2.36
APOA1	Apolipoprotein A1	2.24
CAT	Catalase	2.19
ELAVL1	ELAV Like RNA Binding Prote	2.08
MECP2	Methyl-CpG Binding Protein 2	2.06
TXNRD2	Thioredoxin Reductase 2	2.06
HBEGF	Heparin Binding EGF Like Grov	2.06
FABP2	Fatty Acid Binding Protein 2	2.06
TXN2	Thioredoxin 2	2.06
G6PC3	Glucose-6-Phosphatase Catalytic	2.06
FN3K	Fructosamine 3 Kinase	2.06
FN3KRP	Fructosamine 3 Kinase Related I	2.06
VASH1	Vasohibin 1	2.06
CFH	Complement Factor H	2.01
AHSG	Alpha 2-HS Glycoprotein	2
NPPB	Natriuretic Peptide B	1.94
CST3	Cystatin C	1.84
NLRP3	NLR Family Pyrin Domain Cont	1.82
MAP2K7	Mitogen-Activated Protein Kina	1.78
ARHGEF18	Rho/Rac Guanine Nucleotide Ex	1.78
KHSRP	KH-Type Splicing Regulatory Pr	1.78
FLCN	Folliculin	1.78
SORBS1	Sorbin And SH3 Domain Contai	1.78
PSPN	Persephin	1.78
GTF2F1	General Transcription Factor IIF	1.78
XAB2	XPA Binding Protein 2	1.78
POP4	POP4 Homolog, Ribonuclease P	1.78
GPR108	G Protein-Coupled Receptor 108	1.78
FSTL5	Follistatin Like 5	1.78
TIPARP	TCDD Inducible Poly(ADP-Ribo	1.78
SSR3	Signal Sequence Receptor Subur	1.78
ZNF557	Zinc Finger Protein 557	1.78
WDFY4	WDFY Family Member 4	1.78
TENM2	Teneurin Transmembrane Protei	1.78
PLD6	Phospholipase D Family Membe	1.78
CAMSAP3	Calmodulin Regulated Spectrin .	1.78

TMEM94	Transmembrane Protein 94	1.78
LINC00880	Long Intergenic Non-Protein Co	1.78
TIPARP-AS1	TIPARP Antisense RNA 1	1.78
ARHGAP22-IT1	ARHGAP22 Intronic Transcript	1.78
RNU4-77P	RNA, U4 Small Nuclear 77, Pse	1.78
RNU6-938P	RNA, U6 Small Nuclear 938, Ps	1.78
RPL26P11	Ribosomal Protein L26 Pseudog	1.78
AMD1P1	Adenosylmethionine Decarboxy	1.78
RNA5SP459	RNA, 5S Ribosomal Pseudogen	1.78
LINC01947	Long Intergenic Non-Protein Co	1.78
ENSG00000243176		1.78
ENSG00000241770		1.78
LOC100420048	Poly(RC) Binding Protein 1 Pse	1.78
ENSG00000260328		1.78
ENSG00000279822		1.78
ENSG00000238246		1.78
GC16P007369		1.78
GC16M007351		1.78
LOC105373389	Uncharacterized LOC10537338	1.78
LOC105378314	Uncharacterized LOC10537831	1.78
PIR43800		1.78
GC10M095525		1.78
GC19P007151		1.78
GC19P007214		1.78
GC03M157131		1.78
LOC105374179	Uncharacterized LOC10537417	1.78
LOC105378313	Uncharacterized LOC10537831	1.78
LOC107985839	Uncharacterized LOC10798583	1.78
GC01M173270		1.78
LOC105377515	Uncharacterized LOC10537751	1.78
PARP1	Poly(ADP-Ribose) Polymerase	1.76
CASP3	Caspase 3	1.75
UBE2I	Ubiquitin Conjugating Enzyme	1.69
SUMO1	Small Ubiquitin-Like Modifier 1	1.69
NOX1	NADPH Oxidase 1	1.69
GGT1	Gamma-Glutamyltransferase 1	1.68
MMP10	Matrix Metallopeptidase 10	1.68
MAPK1	Mitogen-Activated Protein Kina	1.62
IGFBP2	Insulin Like Growth Factor Binc	1.62
IL18	Interleukin 18	1.59
HNF4A	Hepatocyte Nuclear Factor 4 Al	1.55
LAMP1	Lysosomal Associated Membra	1.53
TGFBR2	Transforming Growth Factor Be	1.47
SLC12A3	Solute Carrier Family 12 Memb	1.47
CYBA	Cytochrome B-245 Alpha Chain	1.47
LIPG	Lipase G, Endothelial Type	1.47
HBA1	Hemoglobin Subunit Alpha 1	1.47
ARMS2	Age-Related Maculopathy Susce	1.47

PDGFB	Platelet Derived Growth Factor S	1.37
GSR	Glutathione-Disulfide Reductase	1.37
TNFRSF1B	TNF Receptor Superfamily Men	1.37
CTLA4	Cytotoxic T-Lymphocyte Associ	1.37
PDGFA	Platelet Derived Growth Factor S	1.37
IGFBP4	Insulin Like Growth Factor Binc	1.37
CAPN10	Calpain 10	1.37
MT-TL1	Mitochondrially Encoded TRNA	1.37
BCL2	BCL2, Apoptosis Regulator	1.35
LEP	Leptin	1.35
GLP1R	Glucagon Like Peptide 1 Recept	1.35
GAS6	Growth Arrest Specific 6	1.35
PRKCA	Protein Kinase C Alpha	1.27
NEUROD1	Neuronal Differentiation 1	1.27
NOX4	NADPH Oxidase 4	1.27
PLAU	Plasminogen Activator, Urokina	1.25
AIFM1	Apoptosis Inducing Factor Mito	1.25
IL2RB	Interleukin 2 Receptor Subunit E	1.25
IGFBP7	Insulin Like Growth Factor Binc	1.25
MAPK10	Mitogen-Activated Protein Kina	1.19
ETS1	ETS Proto-Oncogene 1, Transcr	1.19
RPS6KB1	Ribosomal Protein S6 Kinase B1	1.19
GLO1	Glyoxalase I	1.19
FGF5	Fibroblast Growth Factor 5	1.19
HSP90B1	Heat Shock Protein 90 Beta Fam	1.19
SIRT6	Sirtuin 6	1.19
ARNT	Aryl Hydrocarbon Receptor Nuc	1.19
CMA1	Chymase 1	1.19
LTF	Lactotransferrin	1.19
MFAP4	Microfibril Associated Protein 4	1.19
NFAT5	Nuclear Factor Of Activated T C	1.19
RAF1	Raf-1 Proto-Oncogene, Serine/T	1.1
FGFR1	Fibroblast Growth Factor Recep	1.1
MTOR	Mechanistic Target Of Rapamyc	1.1
KIT	KIT Proto-Oncogene Receptor T	1.1
HRAS	HRas Proto-Oncogene, GTPase	1.1
NTRK2	Neurotrophic Receptor Tyrosine	1.1
CSF1R	Colony Stimulating Factor 1 Rec	1.1
TGFBR1	Transforming Growth Factor Be	1.1
CAPN1	Calpain 1	1.1
SERPINC1	Serpin Family C Member 1	1.1
EGLN1	Egl-9 Family Hypoxia Inducible	1.1
FASLG	Fas Ligand	1.1
FOXO1	Forkhead Box O1	1.1
ITGAV	Integrin Subunit Alpha V	1.1
ANXA5	Annexin A5	1.1
CD28	CD28 Molecule	1.1
CCR5	C-C Motif Chemokine Receptor	1.1

RHOA	Ras Homolog Family Member A	1.1
SPP1	Secreted Phosphoprotein 1	1.1
SLC19A3	Solute Carrier Family 19 Membe	1.1
TGFBI	Transforming Growth Factor Be	1.1
APOH	Apolipoprotein H	1.1
ACP1	Acid Phosphatase 1	1.1
SETD7	SET Domain Containing Lysine	1.1
MKI67	Marker Of Proliferation Ki-67	1.1
NPPC	Natriuretic Peptide C	1.1
SUV39H1	Suppressor Of Variegation 3-9 H	1.1
SUV39H2	Suppressor Of Variegation 3-9 H	1.1
SCG2	Secretogranin II	1.1
OPTC	Opticin	1.1
CDKAL1	CDK5 Regulatory Subunit Asso	1.1
CASP12	Caspase 12 (Gene/Pseudogene)	1.1
MIR15A	MicroRNA 15a	1.1
TP53	Tumor Protein P53	0.97
IL2RA	Interleukin 2 Receptor Subunit A	0.97
GSTP1	Glutathione S-Transferase Pi 1	0.97
EPHB4	EPH Receptor B4	0.97
EPHB2	EPH Receptor B2	0.97
FAS	Fas Cell Surface Death Receptor	0.97
GRIA1	Glutamate Ionotropic Receptor A	0.97
GRIA2	Glutamate Ionotropic Receptor A	0.97
RUNX1	Runt Related Transcription Fact	0.97
NR1H2	Nuclear Receptor Subfamily 1 G	0.97
TBXAS1	Thromboxane A Synthase 1	0.97
SMAD3	SMAD Family Member 3	0.97
PTGER3	Prostaglandin E Receptor 3	0.97
GRIA4	Glutamate Ionotropic Receptor A	0.97
ACE2	Angiotensin I Converting Enzym	0.97
PPM1D	Protein Phosphatase, Mg2+/Mn2	0.97
ENO2	Enolase 2	0.97
SGK1	Serum/Glucocorticoid Regulated	0.97
ALDH1A1	Aldehyde Dehydrogenase 1 Fam	0.97
COL4A1	Collagen Type IV Alpha 1 Chain	0.97
ALPP	Alkaline Phosphatase, Placental	0.97
GAD2	Glutamate Decarboxylase 2	0.97
KL	Klotho	0.97
FGF3	Fibroblast Growth Factor 3	0.97
CFB	Complement Factor B	0.97
ADAMTS13	ADAM Metallopeptidase With T	0.97
ITGAL	Integrin Subunit Alpha L	0.97
CTSF	Cathepsin F	0.97
ELOVL4	ELOVL Fatty Acid Elongase 4	0.97
EFNB2	Ephrin B2	0.97
CX3CR1	C-X3-C Motif Chemokine Recep	0.97
EPHB3	EPH Receptor B3	0.97

PON3	Paraoxonase 3	0.97
SERPINF2	Serpin Family F Member 2	0.97
HNF1B	HNF1 Homeobox B	0.97
C5AR1	Complement C5a Receptor 1	0.97
IGF2BP2	Insulin Like Growth Factor 2 MI	0.97
SMAD1	SMAD Family Member 1	0.97
SOCS1	Suppressor Of Cytokine Signalir	0.97
PTGES3	Prostaglandin E Synthase 3	0.97
HGS	Hepatocyte Growth Factor-Regu	0.97
IL18R1	Interleukin 18 Receptor 1	0.97
IGFBP5	Insulin Like Growth Factor Binc	0.97
PER2	Period Circadian Regulator 2	0.97
SRF	Serum Response Factor	0.97
CX3CL1	C-X3-C Motif Chemokine Ligar	0.97
PTGES	Prostaglandin E Synthase	0.97
GDF15	Growth Differentiation Factor 1!	0.97
ENTPD5	Ectonucleoside Triphosphate Di	0.97
TSC22D1	TSC22 Domain Family Member	0.97
TIMP4	TIMP Metallopeptidase Inhibito	0.97
PAPPA	Pappalysin 1	0.97
SLPI	Secretory Leukocyte Peptidase I	0.97
MMP25	Matrix Metallopeptidase 25	0.97
MIOX	Myo-Inositol Oxygenase	0.97
8-Sep	Septin 8	0.97
HCAR2	Hydroxycarboxylic Acid Recept	0.97
PF4V1	Platelet Factor 4 Variant 1	0.97
5-Sep	Septin 5	0.97
11-Sep	Septin 11	0.97
VASH2	Vasohibin 2	0.97
VEGFD	Vascular Endothelial Growth Fa	0.97
MIR200C	MicroRNA 200c	0.97
MIR92A1	MicroRNA 92a-1	0.97
CP	Ceruloplasmin	0.95
APOB	Apolipoprotein B	0.95
SLC6A6	Solute Carrier Family 6 Member	0.95
LEPQTL1	Leptin, Serum Levels Of	0.95
JAK2	Janus Kinase 2	0.67
SLC9A1	Solute Carrier Family 9 Member	0.67
PIK3CA	Phosphatidylinositol-4,5-Bispho	0.67
FOS	Fos Proto-Oncogene, AP-1 Tran	0.67
SMAD4	SMAD Family Member 4	0.67
LPL	Lipoprotein Lipase	0.67
CD4	CD4 Molecule	0.67
CYCS	Cytochrome C, Somatic	0.67
CD36	CD36 Molecule	0.67
MCL1	MCL1, BCL2 Family Apoptosis	0.67
BAD	BCL2 Associated Agonist Of Ce	0.67
BIRC5	Baculoviral IAP Repeat Contain	0.67

FADD	Fas Associated Via Death Doma	0.67
OGG1	8-Oxoguanine DNA Glycosylase	0.67
SMAD2	SMAD Family Member 2	0.67
F8	Coagulation Factor VIII	0.67
PROS1	Protein S	0.67
ACACB	Acetyl-CoA Carboxylase Beta	0.67
FGB	Fibrinogen Beta Chain	0.67
A2M	Alpha-2-Macroglobulin	0.67
FCER2	Fc Fragment Of IgE Receptor II	0.67
BTC	Betacellulin	0.67
DLC1	DLC1 Rho GTPase Activating P	0.67
SEMA3A	Semaphorin 3A	0.67
SHC1	SHC Adaptor Protein 1	0.67
XBP1	X-Box Binding Protein 1	0.67
BACE1	Beta-Secretase 1	0.67
GREM1	Gremlin 1, DAN Family BMP A	0.67
ADH1B	Alcohol Dehydrogenase 1B (Cla	0.67
FTO	FTO, Alpha-Ketoglutarate Depe	0.67
CD47	CD47 Molecule	0.67
SIRPA	Signal Regulatory Protein Alpha	0.67
P2RX7	Purinergic Receptor P2X 7	0.67
MUTYH	MutY DNA Glycosylase	0.67
NTF3	Neurotrophin 3	0.67
NTF4	Neurotrophin 4	0.67
APOC3	Apolipoprotein C3	0.67
AMBP	Alpha-1-Microglobulin/Bikunin	0.67
CNTF	Ciliary Neurotrophic Factor	0.67
LAP3	Leucine Aminopeptidase 3	0.67
MTRR	5-Methyltetrahydrofolate-Homo	0.67
TRADD	TNFRSF1A Associated Via Dea	0.67
H6PD	Hexose-6-Phosphate Dehydroge	0.67
LYVE1	Lymphatic Vessel Endothelial H	0.67
NCALD	Neurocalcin Delta	0.67
UMOD	Uromodulin	0.67
XRCC1	X-Ray Repair Cross Complemen	0.67
BACE2	Beta-Secretase 2	0.67
MIP	Major Intrinsic Protein Of Lens	0.67
FCGRT	Fc Fragment Of IgG Receptor A	0.67
SGCB	Sarcoglycan Beta	0.67
ELMO1	Engulfment And Cell Motility 1	0.67
ALMS1	ALMS1, Centrosome And Basal	0.67
ITLN1	Intelectin 1	0.67
SERPINA12	Serpin Family A Member 12	0.67
LCN1	Lipocalin 1	0.67
HPX	Hemopexin	0.67
DGKQ	Diacylglycerol Kinase Theta	0.67
PIP5KL1	Phosphatidylinositol-4-Phosphat	0.67
SCAF4	SR-Related CTD Associated Fac	0.67

MT-CO2	Mitochondrially Encoded Cytoc	0.67
MT-ND5	Mitochondrially Encoded NADH	0.67
SOX2-OT	SOX2 Overlapping Transcript	0.67
SSTR2	Somatostatin Receptor 2	0.61
MIR29A	MicroRNA 29a	0.61
HSPB1	Heat Shock Protein Family B (S	0.52
ADRB1	Adrenoceptor Beta 1	0.52
ATF4	Activating Transcription Factor	0.52
FAAH	Fatty Acid Amide Hydrolase	0.52
EIF2S1	Eukaryotic Translation Initiation	0.52
BDKRB2	Bradykinin Receptor B2	0.52
SRR	Serine Racemase	0.52
PTEN	Phosphatase And Tensin Homol	0.43
HMOX1	Heme Oxygenase 1	0.43
CALR	Calreticulin	0.43
MAPK14	Mitogen-Activated Protein Kina	0.43
UCHL1	Ubiquitin C-Terminal Hydrolase	0.43
ABCB1	ATP Binding Cassette Subfamily	0.43
ACTB	Actin Beta	0.43
PIK3R1	Phosphoinositide-3-Kinase Regu	0.43
P4HB	Prolyl 4-Hydroxylase Subunit B	0.43
EPAS1	Endothelial PAS Domain Protein	0.43
GPX1	Glutathione Peroxidase 1	0.43
GAPDH	Glyceraldehyde-3-Phosphate De	0.43
IL4	Interleukin 4	0.43
ENPP1	Ectonucleotide Pyrophosphatase	0.43
PCSK1	Proprotein Convertase Subtilisin	0.43
VHL	Von Hippel-Lindau Tumor Supp	0.43
TXN	Thioredoxin	0.43
SLC1A6	Solute Carrier Family 1 Member	0.43
PDIA3	Protein Disulfide Isomerase Fam	0.43
SOAT1	Sterol O-Acyltransferase 1	0.43
BEST1	Bestrophin 1	0.43
PRDX4	Peroxiredoxin 4	0.43
HSPA4	Heat Shock Protein Family A (H	0.43
SSTR1	Somatostatin Receptor 1	0.43
BPI	Bactericidal Permeability Increa	0.43
ADCYAP1	Adenylate Cyclase Activating Po	0.43
ANXA3	Annexin A3	0.43
CLDN5	Claudin 5	0.43
BDKRB1	Bradykinin Receptor B1	0.43
COTL1	Coactosin Like F-Actin Binding	0.43
CAPZB	Capping Actin Protein Of Muscl	0.43
SP3	Sp3 Transcription Factor	0.43
SERPINB9	Serpin Family B Member 9	0.43
GDE1	Glycerophosphodiester Phospho	0.43
MIR34A	MicroRNA 34a	0.43
MIR16-1	MicroRNA 16-1	0.43

MIR15B	MicroRNA 15b	0.43
MIR29C	MicroRNA 29c	0.43
FGFR2	Fibroblast Growth Factor Recep	0.3
MAP2K1	Mitogen-Activated Protein Kina	0.3
PDGFRB	Platelet Derived Growth Factor I	0.3
AKT2	AKT Serine/Threonine Kinase 2	0.3
CTNNB1	Catenin Beta 1	0.3
CXCR4	C-X-C Motif Chemokine Recept	0.3
MYLK	Myosin Light Chain Kinase	0.3
COL1A1	Collagen Type I Alpha 1 Chain	0.3
PCNA	Proliferating Cell Nuclear Antig	0.3
EP300	E1A Binding Protein P300	0.3
ACVRL1	Activin A Receptor Like Type 1	0.3
ADAM17	ADAM Metallopeptidase Domai	0.3
PRKAA2	Protein Kinase AMP-Activated (	0.3
PLCG2	Phospholipase C Gamma 2	0.3
CASP7	Caspase 7	0.3
CA2	Carbonic Anhydrase 2	0.3
JAG1	Jagged 1	0.3
BMP4	Bone Morphogenetic Protein 4	0.3
POMC	Proopiomelanocortin	0.3
MAPK3	Mitogen-Activated Protein Kina	0.3
MYH9	Myosin Heavy Chain 9	0.3
MAPK9	Mitogen-Activated Protein Kina	0.3
HSP90AA1	Heat Shock Protein 90 Alpha Fa	0.3
CD40	CD40 Molecule	0.3
ROCK1	Rho Associated Coiled-Coil Con	0.3
SMPD1	Sphingomyelin Phosphodiesteras	0.3
CREB1	CAMP Responsive Element Bin	0.3
SLC16A1	Solute Carrier Family 16 Membe	0.3
F2R	Coagulation Factor II Thrombin	0.3
AQP1	Aquaporin 1 (Colton Blood Grou	0.3
HSPA5	Heat Shock Protein Family A (H	0.3
CA4	Carbonic Anhydrase 4	0.3
MAP3K5	Mitogen-Activated Protein Kina	0.3
MMP7	Matrix Metallopeptidase 7	0.3
IRS1	Insulin Receptor Substrate 1	0.3
MAOA	Monoamine Oxidase A	0.3
LMNA	Lamin A/C	0.3
NPR2	Natriuretic Peptide Receptor 2	0.3
NCF2	Neutrophil Cytosolic Factor 2	0.3
EIF4EBP1	Eukaryotic Translation Initiation	0.3
SIRT3	Sirtuin 3	0.3
YY1	YY1 Transcription Factor	0.3
F2RL1	F2R Like Trypsin Receptor 1	0.3
GLI1	GLI Family Zinc Finger 1	0.3
COL4A3	Collagen Type IV Alpha 3 Chain	0.3
IFNAR1	Interferon Alpha And Beta Rece	0.3

S100B	S100 Calcium Binding Protein E	0.3
TNFSF10	TNF Superfamily Member 10	0.3
PDK1	Pyruvate Dehydrogenase Kinase	0.3
PXN	Paxillin	0.3
COL18A1	Collagen Type XVIII Alpha 1 C	0.3
IDO1	Indoleamine 2,3-Dioxygenase 1	0.3
HSP90AB1	Heat Shock Protein 90 Alpha Fa	0.3
JAM3	Junctional Adhesion Molecule 3	0.3
ITGA3	Integrin Subunit Alpha 3	0.3
DKK1	Dickkopf WNT Signaling Pathw	0.3
NPR1	Natriuretic Peptide Receptor 1	0.3
SIAH1	Siah E3 Ubiquitin Protein Ligas	0.3
VEGFB	Vascular Endothelial Growth Fa	0.3
AOX1	Aldehyde Oxidase 1	0.3
CCR2	C-C Motif Chemokine Receptor	0.3
PTPRB	Protein Tyrosine Phosphatase, R	0.3
S1PR1	Sphingosine-1-Phosphate Recep	0.3
NPR3	Natriuretic Peptide Receptor 3	0.3
SOCS3	Suppressor Of Cytokine Signalir	0.3
ST3GAL5	ST3 Beta-Galactoside Alpha-2,3	0.3
GHRL	Ghrelin And Obestatin Prepropе	0.3
ACAA1	Acetyl-CoA Acyltransferase 1	0.3
ABCA4	ATP Binding Cassette Subfamily	0.3
COL4A2	Collagen Type IV Alpha 2 Chair	0.3
DDAH1	Dimethylarginine Dimethylamin	0.3
SLC2A10	Solute Carrier Family 2 Member	0.3
SERPINB5	Serpin Family B Member 5	0.3
EGR1	Early Growth Response 1	0.3
LIF	LIF, Interleukin 6 Family Cytoki	0.3
CRYAA	Crystallin Alpha A	0.3
SYP	Synaptophysin	0.3
VIP	Vasoactive Intestinal Peptide	0.3
HEY1	Hes Related Family BHLH Tran	0.3
HHEX	Hematopoietically Expressed Hc	0.3
LAMA5	Laminin Subunit Alpha 5	0.3
FBLN2	Fibulin 2	0.3
MMP12	Matrix Metallopeptidase 12	0.3
CYP51A1	Cytochrome P450 Family 51 Sul	0.3
DKK2	Dickkopf WNT Signaling Pathw	0.3
CTTN	Cortactin	0.3
DDAH2	Dimethylarginine Dimethylamin	0.3
TNFRSF12A	TNF Receptor Superfamily Men	0.3
UGCG	UDP-Glucose Ceramide Glucosy	0.3
HRG	Histidine Rich Glycoprotein	0.3
GAP43	Growth Associated Protein 43	0.3
MAOB	Monoamine Oxidase B	0.3
TSPO	Translocator Protein	0.3
TKTL1	Transketolase Like 1	0.3

SLC30A8	Solute Carrier Family 30 Membe	0.3
GCG	Glucagon	0.3
AKAP12	A-Kinase Anchoring Protein 12	0.3
CDKN3	Cyclin Dependent Kinase Inhibi	0.3
IAPP	Islet Amyloid Polypeptide	0.3
HPS1	HPS1, Biogenesis Of Lysosomal	0.3
GAST	Gastrin	0.3
SMPD2	Sphingomyelin Phosphodiestera	0.3
F2RL2	Coagulation Factor II Thrombin	0.3
CA7	Carbonic Anhydrase 7	0.3
RGS5	Regulator Of G Protein Signalin	0.3
RHOD	Ras Homolog Family Member D	0.3
MSRB1	Methionine Sulfoxide Reductase	0.3
LGR4	Leucine Rich Repeat Containing	0.3
SPG11	SPG11, Spatacsin Vesicle Traffi	0.3
HIF3A	Hypoxia Inducible Factor 3 Subu	0.3
RP1L1	RP1 Like 1	0.3
MOAP1	Modulator Of Apoptosis 1	0.3
GCM1	Glial Cells Missing Homolog 1	0.3
MLN	Motilin	0.3
SCT	Secretin	0.3
PLPP3	Phospholipid Phosphatase 3	0.3
MT-ND4	Mitochondrially Encoded NADH	0.3
OR52B4	Olfactory Receptor Family 52 Su	0.3
HSP90AA2P	Heat Shock Protein 90 Alpha Fa	0.3
MIR152	MicroRNA 152	0.3
BDNF-AS	BDNF Antisense RNA	0.3
MIR18B	MicroRNA 18b	0.3
