# Supplementary material for: Exploring the Pharmacological Mechanism of Liuwei Dihuang Decoction for Diabetic Retinopathy: A Systematic Biological Strategy-Based Research
Source: Evid Based Complement Alternat Med. 2021 Aug 2;2021:5544518. doi: 10.1155/2021/5544518 (PMC8356007; doi:10.1155/2021/5544518)
Supplement: Supplementary Materials — Table S1: compound targets for each compounds. Table S2: known targets for each compounds. Table S3: DR genes. Table S4: enrichment analysis of clusters based on Gene Ontology (GO) annotation of DR PPI network. Table S5: pathway enrichment analysis of DR PPI network. Table S6: enrichment analysis of clusters based on Gene Ontology (GO) annotation of LDD-DR PPI network. Table S7: pathway enrichment analysis of LDD-DR PPI network. Table S8: enrichment analysis of clusters based on Gene Ontology (GO) annotation of LDD known target-DR network. Table S9: pathway enrichment analysis of LDD known target-DR network. [file 5544518.f1.zip › 5544518.f1/Table S4.pdf]

Table S4 Enrichment analysis of clusters based on Gene Ontology (GO) annotation

Cluster	Term	Pathway	Count	%	Pvalue
	GO:0006954	inflammatory response	24	0.162448	4.43E-18
	GO:0045429	positive regulation of nitric oxide bios	13	0.087992	4.59E-18
	GO:0043066	negative regulation of apoptotic proce	24	0.162448	2.42E-16
	GO:0043406	positive regulation of MAP kinase act	13	0.087992	3.10E-16
	GO:0001666	response to hypoxia	17	0.115067	1.11E-15
	GO:0070374	positive regulation of ERK1 and ERK	16	0.108298	3.31E-14
	GO:0000165	MAPK cascade	17	0.115067	8.54E-13
	GO:0051092	positive regulation of NF-kappaB tran	13	0.087992	7.80E-12
	GO:0000187	activation of MAPK activity	12	0.081224	1.46E-11
	GO:0014068	positive regulation of phosphatidylino	10	0.067686	7.41E-11
	GO:0001525	angiogenesis	14	0.094761	2.49E-10
	GO:0032868	response to insulin	8	0.054149	7.82E-08
	GO:0035924	cellular response to vascular endotheli	6	0.040612	1.38E-07
	GO:0051770	positive regulation of nitric-oxide syn	5	0.033843	5.81E-07
	GO:0071456	cellular response to hypoxia	8	0.054149	9.43E-07
	GO:0006006	glucose metabolic process	7	0.047381	1.75E-06
	GO:0048010	vascular endothelial growth factor rec	7	0.047381	2.68E-06
	GO:0043536	positive regulation of blood vessel enc	5	0.033843	3.07E-06
	GO:0045766	positive regulation of angiogenesis	8	0.054149	3.19E-06
	GO:0042346	positive regulation of NF-kappaB imp	5	0.033843	4.70E-06
	GO:0070371	ERK1 and ERK2 cascade	5	0.033843	8.24E-06
	GO:0032735	positive regulation of interleukin-12 p	5	0.033843	9.77E-06
	GO:0050999	regulation of nitric-oxide synthase act	5	0.033843	1.15E-05
	GO:0032757	positive regulation of interleukin-8 pro	5	0.033843	1.15E-05
	GO:0050796	regulation of insulin secretion	6	0.040612	3.27E-05
	GO:0071347	cellular response to interleukin-1	6	0.040612	4.33E-05
	GO:0038128	ERBB2 signaling pathway	5	0.033843	5.40E-05
	GO:1904707	positive regulation of vascular smooth	4	0.027075	6.90E-05
	GO:0032755	positive regulation of interleukin-6 pro	5	0.033843	1.06E-04
1	GO:0007179	transforming growth factor beta recep	6	0.040612	1.49E-04
	GO:1900015	regulation of cytokine production invo	3	0.020306	1.77E-04
	GO:0042593	glucose homeostasis	6	0.040612	2.32E-04
	GO:0043123	positive regulation of I-kappaB kinase	7	0.047381	2.56E-04
	GO:0032733	positive regulation of interleukin-10 p	4	0.027075	2.60E-04
	GO:2000379	positive regulation of reactive oxygen	4	0.027075	5.80E-04
	GO:0046326	positive regulation of glucose import	4	0.027075	5.80E-04
	GO:0045765	regulation of angiogenesis	4	0.027075	6.40E-04
	GO:0043552	positive regulation of phosphatidylino	4	0.027075	6.40E-04
	GO:0045907	positive regulation of vasoconstriction	4	0.027075	7.04E-04
	GO:0032700	negative regulation of interleukin-17 p	3	0.020306	0.001581
	GO:0048009	insulin-like growth factor receptor sig	3	0.020306	0.002589
	GO:2000353	positive regulation of endothelial cell	3	0.020306	0.002589
	GO:0007249	I-kappaB kinase/NF-kappaB signaling	4	0.027075	0.004346
	GO:0007263	nitric oxide mediated signal transducti	3	0.020306	0.004779

	GO:0050727	regulation of inflammatory response	4	0.027075	0.004985
	GO:0051000	positive regulation of nitric-oxide syn	3	0.020306	0.006387
	GO:0010575	positive regulation of vascular endoth	3	0.020306	0.009534
	GO:0032715	negative regulation of interleukin-6 pr	3	0.020306	0.010231
	GO:0046666	retinal cell programmed cell death	2	0.013537	0.010928
	GO:0000302	response to reactive oxygen species	3	0.020306	0.019292
	GO:0001895	retina homeostasis	3	0.020306	0.020235
	GO:0072606	interleukin-8 secretion	2	0.013537	0.021738
	GO:0006979	response to oxidative stress	4	0.027075	0.022546
	GO:0032611	interleukin-1 beta production	2	0.013537	0.027099
	GO:0038033	positive regulation of endothelial cell	2	0.013537	0.027099
	GO:0045084	positive regulation of interleukin-12 b	2	0.013537	0.032431
	GO:0038084	vascular endothelial growth factor sig	2	0.013537	0.032431
	GO:0045410	positive regulation of interleukin-6 bio	2	0.013537	0.043008
	GO:0008203	cholesterol metabolic process	4	0.027075	0.006168
	GO:0090370	negative regulation of cholesterol efflu	2	0.013537	0.027099
	GO:0006954	inflammatory response	27	0.148278	1.69E-18
	GO:0001666	response to hypoxia	17	0.09336	1.02E-13
	GO:0001525	angiogenesis	18	0.098852	4.40E-13
	GO:0070374	positive regulation of ERK1 and ERK	14	0.076885	4.04E-10
	GO:0014068	positive regulation of phosphatidylino	9	0.049426	2.06E-08
	GO:0048010	vascular endothelial growth factor rec	8	0.043934	8.47E-07
	GO:0032909	regulation of transforming growth fac	4	0.021967	1.45E-06
	GO:0008217	regulation of blood pressure	7	0.038443	7.21E-06
	GO:0008286	insulin receptor signaling pathway	7	0.038443	2.08E-05
	GO:0009749	response to glucose	6	0.032951	1.30E-04
	GO:0032869	cellular response to insulin stimulus	6	0.032951	2.33E-04
	GO:0050728	negative regulation of inflammatory re	6	0.032951	2.63E-04
	GO:0043410	positive regulation of MAPK cascade	6	0.032951	2.96E-04
	GO:0043065	positive regulation of apoptotic proces	10	0.054918	3.23E-04
	GO:0070373	negative regulation of ERK1 and ERK	5	0.027459	8.06E-04
	GO:0042593	glucose homeostasis	6	0.032951	8.16E-04
**2**	GO:0010575	positive regulation of vascular endoth	4	0.021967	9.41E-04
	GO:0043123	positive regulation of I-kappaB kinase	7	0.038443	0.001098
	GO:0046326	positive regulation of glucose import	4	0.021967	0.001286
	GO:0071347	cellular response to interleukin-1	5	0.027459	0.001718
	GO:0038128	ERBB2 signaling pathway	4	0.021967	0.002562
	GO:0046628	positive regulation of insulin receptor	3	0.016475	0.003813
	GO:0032755	positive regulation of interleukin-6 pro	4	0.021967	0.004154
	GO:1902176	negative regulation of oxidative stress	3	0.016475	0.004428
	GO:0030949	positive regulation of vascular endoth	3	0.016475	0.005785
	GO:0017015	regulation of transforming growth fac	3	0.016475	0.008989
	GO:0045777	positive regulation of blood pressure	3	0.016475	0.008989
	GO:0042346	positive regulation of NF-kappaB imp	3	0.016475	0.009888
	GO:0050727	regulation of inflammatory response	4	0.021967	0.010587

	GO:0046330	positive regulation of JNK cascade	4	0.021967	0.011525
	GO:0032715	negative regulation of interleukin-6 pr	3	0.016475	0.017225
	GO:0032717	negative regulation of interleukin-8 pr	2	0.010984	0.049372
	GO:0070723	response to cholesterol	3	0.016475	0.001402
	GO:0032930	positive regulation of superoxide anio	4	0.114384	1.63E-07
	GO:0042310	vasoconstriction	4	0.114384	1.31E-06
	GO:0008217	regulation of blood pressure	5	0.14298	1.42E-06
	GO:0006954	inflammatory response	7	0.200172	6.98E-06
	GO:0014824	artery smooth muscle contraction	3	0.085788	5.87E-05
	GO:0042554	superoxide anion generation	3	0.085788	1.48E-04
	GO:0006801	superoxide metabolic process	3	0.085788	3.07E-04
**3**	GO:0000302	response to reactive oxygen species	3	0.085788	0.001179
	GO:0086100	endothelin receptor signaling pathway	2	0.057192	0.002619
	GO:0048010	vascular endothelial growth factor rec	3	0.085788	0.003962
	GO:1902177	positive regulation of oxidative stress-	2	0.057192	0.005231
	GO:0003100	regulation of systemic arterial blood p	2	0.057192	0.006534
	GO:0045766	positive regulation of angiogenesis	3	0.085788	0.009821
	GO:0030168	platelet activation	3	0.085788	0.009821
	GO:0019229	regulation of vasoconstriction	2	0.057192	0.025894
	GO:0000302	response to reactive oxygen species	3	0.148002	2.85E-04
**4**	GO:0055114	oxidation-reduction process	5	0.24667	4.14E-04
	GO:0045454	cell redox homeostasis	3	0.148002	0.001111
	GO:0009749	response to glucose	2	0.098668	0.043667
**5**	GO:0071560	cellular response to transforming grov	2	0.2457	0.011622
	GO:0016525	negative regulation of angiogenesis	2	0.2457	0.014689
**6**	GO:0045454	cell redox homeostasis	4	0.221484	1.98E-05
	GO:0000302	response to reactive oxygen species	3	0.166113	3.42E-04
	GO:0014066	regulation of phosphatidylinositol 3-k	3	0.110988	0.002151
	GO:0043410	positive regulation of MAPK cascade	3	0.110988	0.002317
	GO:0001666	response to hypoxia	3	0.110988	0.010034
**7**	GO:0070374	positive regulation of ERK1 and ERK	3	0.110988	0.010372
	GO:0001525	angiogenesis	3	0.110988	0.016452
	GO:0000165	MAPK cascade	3	0.110988	0.022275
	GO:0046627	negative regulation of insulin receptor	2	0.073992	0.025605
	GO:0071333	cellular response to glucose stimulus	2	0.169062	0.02148
**8**	GO:0032869	cellular response to insulin stimulus	2	0.169062	0.031666
	GO:0000187	activation of MAPK activity	2	0.169062	0.043769
	GO:0043410	positive regulation of MAPK cascade	4	0.104548	1.56E-04
	GO:0000165	MAPK cascade	5	0.130685	3.40E-04

9	GO:0045766	positive regulation of angiogenesis	4	0.104548	4.38E-04
	GO:0070374	positive regulation of ERK1 and ERK	4	0.104548	0.001481
	GO:0036092	phosphatidylinositol-3-phosphate bios	3	0.078411	0.001856
	GO:0001525	angiogenesis	4	0.104548	0.002953
	GO:0014066	regulation of phosphatidylinositol 3-k	3	0.078411	0.004633
	GO:0042981	regulation of apoptotic process	3	0.078411	0.031317

ı

Genes	Fold Enrichment	Bonferroni
PIK3CG, IL6, TNF, CCL2, PTGS2, CSF1, TLR2, IL13, NFK	11.43382416	9.11E-15
AKT1, EGFR, ICAM1, IL6, TNF, PTGS2, INS, EDN1, IFNG	54.58764691	9.43E-15
IL4, EGFR, IL6, MMP9, TP53, IGF1, NFKB1, ANXA5, IL10	9.523998582	4.56E-13
EGFR, PIK3CG, HRAS, TNF, FLT1, EDN1, KITLG, CD40,	39.78421724	6.84E-13
EGR1, CCL2, CREB1, TLR2, ITGA2, CXCL12, MMP2, TGI	17.84596149	2.28E-12
EGFR, ICAM1, HRAS, IL6, TNF, CCL2, MAP2K1, TLR4, C	16.50826421	6.80E-11
EGFR, HRAS, TNF, CCL2, MAP2K1, KITLG, KIT, CCL5, T	11.71566938	1.75E-09
ICAM1, AR, IL6, TNF, CD40LG, INS, TLR2, IL1B, NFKB1,	17.64863772	1.60E-08
MAPK1, TNF, MAP2K1, CXCR4, MAPK14, MAPK3, IGF1,	20.24962315	2.99E-08
LEP, FLT1, INS, F2, IGF1, JAK2, CAT, KIT, CCL5, KDR	27.7783292	1.52E-07
PIK3CG, FLT1, CCL2, PTGS2, MMP2, KDR, LEP, MAPK1	11.33555138	5.12E-07
LEP, EGR1, IL6, TNFSF10, TLR2, CAT, MTOR, IL10	21.55930027	1.61E-04
VCAM1, AKT1, FLT1, MAPK14, VEGFA, KDR	47.10238429	2.83E-04
CCL2, TLR2, TLR4, JAK2, KDR	69.44582299	0.001192443
AKT1, ICAM1, PTGS2, BCL2, EDN1, VEGFA, TP53, MTO	15.04659498	0.00193515
LEP, AKT1, TNF, INS, MAPK14, PIK3CA, GAPDH	18.86438774	0.003586926
ACTB, CCL2, FLT1, MAPK14, VEGFA, PIK3CA, KDR	17.55436081	0.005488511
AKT1, MAPK14, VEGFA, FGF2, TGFB1	47.5155631	0.006287414
FLT1, F3, VEGFA, SERPINE1, IL1B, NOS3, FGF2, KDR	12.56063581	0.006523956
TNF, PTGS2, TLR2, IL1B, TLR4	42.99027138	0.009611328
MAPK1, MAP2K1, MAPK3, IGF1, EGF	37.61648746	0.016790017
CD40LG, IFNG, TLR2, TLR4, CD40	36.11182796	0.019873386
LEP, AKT1, EGFR, IL1B, NOS3	34.7229115	0.02334719
TNF, SERPINE1, TLR2, IL1B, TLR4	34.7229115	0.02334719
LEP, TNF, IFNG, IL1B, NOS2, CCL5	16.1694752	0.064923657
ICAM1, IL6, CCL2, EDN1, NFKB1, CCL5	15.25851886	0.085110367
AKT1, EGFR, HRAS, PIK3CA, EGF	23.75778155	0.104997524
MMP9, JAK2, MMP2, IL10	48.14910394	0.13222117
IL6, TNF, TLR2, IL1B, TLR4	20.06212664	0.195435926
FOS, CCL2, JUN, CREB1, PARP1, TGFB1	11.77559607	0.264326551
LEP, MAPK14, NOS2	135.4193548	0.304660638
LEP, AKT1, IL6, INS, PPARG, STAT3	10.72628553	0.378546001
TNFRSF1A, TNFSF10, TNF, FASLG, CD40, CASP1, CTNN	7.850397382	0.408579904
IL4, CD40LG, TLR2, TLR4	31.40158953	0.414210967
LEP, MAPK14, F2, TP53	24.07455197	0.696586259
AKT1, INS, MAPK14, IGF1	24.07455197	0.696586259
LEP, IL6, FGF2, CTNNB1	23.29795352	0.731596445
FLT1, KIT, FGF2, TGFB1	23.29795352	0.731596445
AKT1, EGFR, ICAM1, PTGS2	22.56989247	0.764404047
IFNG, TLR4, TGFB1	49.24340176	0.961255821
AKT1, IGF1R, IGF1	38.69124424	0.995126857
CD40LG, FASLG, CD40	38.69124424	0.995126857
TNFRSF1A, TNF, NFKB1, TLR4	12.03727599	0.999869856
APOE, NOS3, NOS2	28.50933786	0.999946658

PTGS2, JAK2, CASP1, SELE	11.46407237	0.999965136
AKT1, APOE, INS	24.62170088	0.999998075
PTGS2, IL1B, TGFB1	20.06212664	0.999999997
TNF, TLR4, IL10	19.34562212	0.999999999
BAX, FASLG	180.5591398	1
APOE, CYCS, CAT	13.8891646	1
ACTB, TF, ALB	13.54193548	1
LEP, NOS2	90.27956989	1
AKT1, EGFR, PTGS2, APOE	6.565786901	1
IL1B, CASP1	72.22365591	1
VEGFA, KDR	72.22365591	1
IFNG, TLR4	60.18637993	1
FLT1, VEGFA	60.18637993	1
IFNG, IL1B	45.13978495	1
LEP, IL4, APOE, CAT	10.62112587	0.999996976
APOE, EGF	72.22365591	1
CCL3, CXCL5, IL18, C5, CRP, CXCL9, BDKRB1, PF4, BDI	9.805441412	2.85E-15
PLAT, PGF, SMAD4, RAF1, SMAD3, MMP14, ADIPOQ, A'	13.60388868	1.73E-10
PGF, IL18, CXCR3, MMP14, ECM1, SIRT1, PTEN, TGFB2,	11.10990223	7.43E-10
CCL3, C5AR1, CCL4, PTEN, GAS6, VEGFB, CD36, FGB, C	11.01114754	6.82E-07
SELP, AGT, IL18, TEK, PDGFRB, ANGPT1, HGF, SIRT1, T	19.05775536	3.48E-05
VEGFB, VEGFC, CYBB, HSP90AA1, PGF, RHOA, HSPB1,	15.29326047	0.001430061
HIF1A, SMAD4, SMAD3, TGFB2	137.6393443	0.002451335
AGTR2, NPY, REN, AGT, HMOX1, GNB3, SOD2	14.82269861	0.012118412
GRB2, FOXO1, IGF2, SHC1, IRS1, INSR, AKT2	12.35224884	0.034523046
HNF4A, CTGF, SMAD2, RPS6KB1, PTEN, ADIPOQ	12.14464802	0.196846549
SP1, FOXO1, IRS1, ADIPOQ, INSR, AKT2	10.72514371	0.326072945
TNFRSF1B, SOCS3, TEK, SMAD3, HGF, ADIPOQ	10.45362108	0.359199432
LIF, TNFRSF1B, IGF2, FAS, IL6R, INSR	10.19550698	0.393583426
KNG1, HMOX1, CNR1, CTLA4, PDGFRB, FOXO1, HRG, F	4.587978142	0.421048418
LIF, SMAD4, PTEN, TIMP3, ADIPOQ	11.86546071	0.744020672
HIF1A, HNF4A, CNR1, IRS1, ADIPOQ, INSR	8.176594709	0.748348355
HIF1A, C5AR1, C5, IL1A	20.39101396	0.796232414
CD36, HMOX1, RHOA, ECM1, ADIPOQ, IL1A, TLR9	5.984319316	0.843726465
IRS1, ADIPOQ, INSR, AKT2	18.35191257	0.886273678
IL17A, CCL3, HIF1A, FGB, CCL4	9.692911568	0.94528885
HSP90AA1, GRB2, HBEGF, SHC1	14.48835203	0.98689336
IGF2, IRS1, SIRT1	31.7629256	0.998430594
CD36, IL6R, IL1A, TLR9	12.23460838	0.999119574
HSPB1, SIRT1, SOD2	29.4941452	0.999447009
VEGFB, HIF1A, VTN	25.80737705	0.999944792
SMAD4, SMAD3, SMAD2	20.64590164	0.999999764
AGT, CNR1, ADIPOQ	20.64590164	0.999999764
IL18, RHOA, TLR9	19.66276347	0.999999949
AGTR1, IL1R1, GGT1, AHSG	8.739005985	0.999999985

SERPINF2, CTGF, IL1A, TLR9	8.470113493	0.999999997
HGF, GAS6, TLR9	14.7470726	1
IL6R, TLR9	39.32552693	1
CCL3, CCR5, SMAD2	51.6147541	0.906551936
CYBA, F2RL1, SOD1, PRKCD	324.4830918	8.25E-05
EDNRA, EDNRB, EDN3, EDN2	171.7851662	6.63E-04
EDNRA, EDNRB, UTS2, NOX1, SOD1	56.16053512	7.18E-04
CYBA, ITGAL, NOX1, RAC1, F2RL1, ITGB2, NFE2L2	13.48445566	0.003525943
EDNRA, EDN3, EDN2	243.3623188	0.029247198
CYBA, NOX1, SOD1	156.447205	0.072014451
CYBA, NOX1, SOD1	109.5130435	0.143858497
CYBA, GSR, SOD1	56.16053512	0.44950952
EDNRA, EDNRB	730.0869565	0.734669297
CYBA, ITGAV, RAC1	30.42028986	0.865846844
NOX1, SOD1	365.0434783	0.929610751
EDN3, EDN2	292.0347826	0.963747332
UTS2, ITGB2, NFE2L2	19.04574669	0.993222589
F2RL2, RAC1, PRKCD	19.04574669	0.993222589
EDN3, EDN2	73.00869565	0.999998283
TXN2, TXNRD2, PRDX1	107.6410256	0.031451954
H6PD, TXN2, AKR1B1, TXNRD2, PRDX1	11.81869369	0.045322981
TXN2, TXNRD2, PRDX1	54.51948052	0.11709625
GLUL, TXN2	41.15686275	0.993266523
COL4A2, COL1A1	137.077551	0.569024507
COL4A3, COL4A2	108.3354839	0.655419243
GPX1, P4HB, PDIA3, PRDX4	67.1008991	0.003912405
GPX1, P4HB, SOD3	99.36094675	0.065456714
FGFR2, PDGFB, PDGFA	40.36538462	0.61636502
FGFR2, PDGFB, PDGFA	38.87037037	0.643764674
PPARA, THBS1, PRKCB	18.30523256	0.9887543
FGFR2, PDGFB, PDGFA	17.99142857	0.990340146
FGFR2, PDGFA, FN1	14.11883408	0.999377763
FGFR2, PDGFB, PDGFA	12.01717557	0.999955691
PTPN1, PRKCB	72.37931034	0.999990292
UCP2, PPARGC1A	80.73076923	0.987280463
UCP2, GHR	54.51948052	0.998447391
GHRL, GHR	39.23364486	0.999876073
OSM, FGFR1, TIMP2, IGFBP3	36.05367687	0.080726755
FGFR1, MAP3K5, IL2RA, FGF1, GDNF	13.93295719	0.167541031

PRKCA, ETS1, FGF1, ANGPT2	25.39432892	0.210764489
PRKCA, BMP4, GCG, FGF1	16.68770186	0.550816688
FGFR1, FGF1, PIK3R1	44.69920142	0.633367555
PRKCA, FGFR1, FGF1, ANGPT2	13.09573016	0.797522815
FGFR1, FGF1, PIK3R1	28.08026756	0.918534049
ETS1, FADD, IGFBP3	10.28291488	0.999999965
