# Supplementary material for: Exploring the Pharmacological Mechanism of Liuwei Dihuang Decoction for Diabetic Retinopathy: A Systematic Biological Strategy-Based Research
Source: Evid Based Complement Alternat Med. 2021 Aug 2;2021:5544518. doi: 10.1155/2021/5544518 (PMC8356007; doi:10.1155/2021/5544518)
Supplement: Supplementary Materials — Table S1: compound targets for each compounds. Table S2: known targets for each compounds. Table S3: DR genes. Table S4: enrichment analysis of clusters based on Gene Ontology (GO) annotation of DR PPI network. Table S5: pathway enrichment analysis of DR PPI network. Table S6: enrichment analysis of clusters based on Gene Ontology (GO) annotation of LDD-DR PPI network. Table S7: pathway enrichment analysis of LDD-DR PPI network. Table S8: enrichment analysis of clusters based on Gene Ontology (GO) annotation of LDD known target-DR network. Table S9: pathway enrichment analysis of LDD known target-DR network. [file 5544518.f1.zip › 5544518.f1/Table S5.pdf]

Table S5 Pathway enrichment analysis

Term	Pathway	Count	%	Pvalue
hsa04151	PI3K-Akt signaling pathway	109	0.093291	2.05E-38
hsa04066	HIF-1 signaling pathway	51	0.04365	5.93E-30
hsa04668	TNF signaling pathway	50	0.042794	4.49E-26
hsa04068	FoxO signaling pathway	47	0.040226	2.24E-18
hsa04620	Toll-like receptor signaling pathway	37	0.031668	2.12E-14
hsa04931	Insulin resistance	36	0.030812	2.44E-13
hsa04012	ErbB signaling pathway	30	0.025676	1.31E-11
hsa04010	MAPK signaling pathway	55	0.047073	1.61E-11
hsa04370	VEGF signaling pathway	24	0.020541	1.04E-10
hsa04910	Insulin signaling pathway	34	0.0291	8.79E-09
hsa04930	Type II diabetes mellitus	19	0.016262	1.29E-08
hsa04940	Type I diabetes mellitus	17	0.01455	6.46E-08
hsa04150	mTOR signaling pathway	19	0.016262	3.66E-07
hsa04064	NF-kappa B signaling pathway	23	0.019685	9.97E-07
hsa04350	TGF-beta signaling pathway	20	0.017118	3.06E-05
hsa03320	PPAR signaling pathway	14	0.011982	0.002446
hsa04310	Wnt signaling pathway	19	0.016262	0.029437

Genes	Fold Enrichment	Bonferroni
HRAS, PDGFB, PDGFA, PGF, TLR2, VTN, TLR4, RPS6KB	3.887952088	5.53E-36
EDN1, NFKB1, TLR4, RPS6KB1, AKT1, EIF4EBP1, HMOX	6.537511181	1.6015E-27
PTGS2, MMP9, EDN1, NFKB1, JAG1, MMP3, CXCL10, AK	5.750422149	1.21104E-23
HRAS, GRB2, FASLG, FOXO1, PTEN, IL10, G6PC3, TGFB	4.3162497	6.04731E-16
CCL3, TNF, TLR2, CXCL9, CXCL8, NFKB1, TLR4, CCL5,	4.295456847	5.72542E-12
PPARA, TNF, FOXO1, NFKB1, RPS6KB1, PTEN, G6PC3, A	4.1019678	6.57974E-11
HRAS, GRB2, BTC, RPS6KB1, AKT1, EIF4EBP1, TGFA, P	4.243414965	3.54071E-09
FGF5, HRAS, PDGFB, PDGFA, FASLG, NFKB1, TGFB1, T	2.675196391	4.33E-09
PRKCA, PIK3CG, HRAS, MAP2K1, PTGS2, RAF1, BAD, P	4.841666911	2.81398E-08
HRAS, GRB2, FOXO1, RPS6KB1, G6PC3, AKT1, EIF4EBP	3.031889243	2.37262E-06
PIK3CG, TNF, SOCS3, SOCS1, MAPK10, PRKCD, ADIPOQ	4.871086762	3.47161E-06
HLA-DQB1, TNF, HLA-DRB1, HLA-A, FASLG, HLA-B, HI	4.9809609	1.74408E-05
PRKCA, PIK3CG, TNF, IGF1, RPS6KB1, IRS1, PTEN, PRK	4.031244217	9.87862E-05
ICAM1, IL1R1, TNF, PTGS2, CXCL8, NFKB1, TLR4, UBE2	3.253284807	0.000269134
BMP4, TNF, ROCK1, TGFBR1, TGFBR2, SMAD4, SMAD3	2.929977	0.008229038
PPARA, LPL, PPARG, RXRG, UCP1, ADIPOQ, MMP1, APO	2.5713828	0.483797689
PRKCA, ROCK2, TP53, SMAD4, MMP7, MAPK10, TCF7L	1.694291048	0.999686353
