# Supplementary material for: Exploring the Pharmacological Mechanism of Liuwei Dihuang Decoction for Diabetic Retinopathy: A Systematic Biological Strategy-Based Research
Source: Evid Based Complement Alternat Med. 2021 Aug 2;2021:5544518. doi: 10.1155/2021/5544518 (PMC8356007; doi:10.1155/2021/5544518)
Supplement: Supplementary Materials — Table S1: compound targets for each compounds. Table S2: known targets for each compounds. Table S3: DR genes. Table S4: enrichment analysis of clusters based on Gene Ontology (GO) annotation of DR PPI network. Table S5: pathway enrichment analysis of DR PPI network. Table S6: enrichment analysis of clusters based on Gene Ontology (GO) annotation of LDD-DR PPI network. Table S7: pathway enrichment analysis of LDD-DR PPI network. Table S8: enrichment analysis of clusters based on Gene Ontology (GO) annotation of LDD known target-DR network. Table S9: pathway enrichment analysis of LDD known target-DR network. [file 5544518.f1.zip › 5544518.f1/Table S6.pdf]

Table S6 Enrichment analysis of clusters based on Gene Ontology (GO) annotation

Cluster	Term	Pathway	Count	%	Pvalue
	GO:0045429	positive regulation of nitric oxide bios	15	0.084684	6.59E-21
	GO:0043066	negative regulation of apoptotic proce	28	0.158076	7.55E-19
	GO:0000165	MAPK cascade	23	0.129848	1.26E-18
	GO:0006954	inflammatory response	24	0.135494	2.52E-16
	GO:0070374	positive regulation of ERK1 and ERK	18	0.10162	1.35E-15
	GO:0043406	positive regulation of MAP kinase act	11	0.062101	4.20E-12
	GO:0000187	activation of MAPK activity	13	0.073392	4.47E-12
	GO:0001666	response to hypoxia	15	0.084684	5.66E-12
	GO:0014066	regulation of phosphatidylinositol 3-k	11	0.062101	7.60E-11
	GO:0038128	ERBB2 signaling pathway	9	0.05081	1.01E-10
	GO:0001525	angiogenesis	15	0.084684	1.86E-10
	GO:0051092	positive regulation of NF-kappaB tran	12	0.067747	1.01E-09
	GO:0014068	positive regulation of phosphatidylino	9	0.05081	9.03E-09
	GO:0032868	response to insulin	9	0.05081	1.15E-08
	GO:0048010	vascular endothelial growth factor rec	9	0.05081	2.06E-08
	GO:0050729	positive regulation of inflammatory re	9	0.05081	2.30E-08
	GO:0043410	positive regulation of MAPK cascade	9	0.05081	5.28E-08
	GO:0042346	positive regulation of NF-kappaB imp	6	0.033873	1.97E-07
	GO:0008217	regulation of blood pressure	8	0.045165	2.04E-07
	GO:1904707	positive regulation of vascular smooth	5	0.028228	2.17E-06
	GO:0007179	transforming growth factor beta recep	8	0.045165	2.23E-06
	GO:0006006	glucose metabolic process	7	0.039519	4.71E-06
	GO:0071347	cellular response to interleukin-1	7	0.039519	6.62E-06
**1**	GO:0032757	positive regulation of interleukin-8 pr	5	0.028228	2.25E-05
	GO:0046326	positive regulation of glucose import	5	0.028228	4.04E-05
	GO:0045765	regulation of angiogenesis	5	0.028228	4.62E-05
	GO:0042593	glucose homeostasis	7	0.039519	5.01E-05
	GO:0045907	positive regulation of vasoconstrictior	5	0.028228	5.25E-05
	GO:0046628	positive regulation of insulin receptor	4	0.022582	7.26E-05
	GO:0048009	insulin-like growth factor receptor sig	4	0.022582	9.19E-05
	GO:0045766	positive regulation of angiogenesis	7	0.039519	1.03E-04
	GO:0032869	cellular response to insulin stimulus	6	0.033873	1.43E-04
	GO:0008286	insulin receptor signaling pathway	6	0.033873	1.52E-04
	GO:0032755	positive regulation of interleukin-6 pr	5	0.028228	2.04E-04
	GO:0035924	cellular response to vascular endothel	4	0.022582	4.29E-04
	GO:0045909	positive regulation of vasodilation	4	0.022582	8.60E-04
	GO:0050796	regulation of insulin secretion	5	0.028228	9.40E-04
	GO:0007223	Wnt signaling pathway, calcium modu	4	0.022582	0.002051
	GO:0007249	I-kappaB kinase/NF-kappaB signaling	4	0.022582	0.006961
	GO:0009749	response to glucose	4	0.022582	0.009822
	GO:0046666	retinal cell programmed cell death	2	0.011291	0.012941
	GO:0032715	negative regulation of interleukin-6 pr	3	0.016937	0.014138
	GO:0010574	regulation of vascular endothelial grov	2	0.011291	0.025715
	GO:1990314	cellular response to insulin-like growt	2	0.011291	0.032041

	GO:0071560	cellular response to transforming grov	3	0.016937	0.040287
	GO:0008203	cholesterol metabolic process	4	0.022582	0.009822
	GO:0090370	negative regulation of cholesterol efflu	2	0.011291	0.032041
	GO:0006954	inflammatory response	27	0.153601	8.65E-19
	GO:0045766	positive regulation of angiogenesis	13	0.073956	2.75E-11
	GO:0070374	positive regulation of ERK1 and ERK	14	0.079645	2.94E-10
	GO:0001525	angiogenesis	14	0.079645	5.73E-09
	GO:0016525	negative regulation of angiogenesis	9	0.0512	1.15E-08
	GO:0014068	positive regulation of phosphatidylino	9	0.0512	1.69E-08
	GO:0032909	regulation of transforming growth fac	4	0.022756	1.35E-06
	GO:0048010	vascular endothelial growth factor rec	7	0.039823	1.13E-05
	GO:0007179	transforming growth factor beta recep	7	0.039823	4.62E-05
	GO:0050729	positive regulation of inflammatory re	6	0.034134	1.61E-04
	GO:0032869	cellular response to insulin stimulus	6	0.034134	2.08E-04
	GO:0014066	regulation of phosphatidylinositol 3-k	6	0.034134	2.21E-04
	GO:0050728	negative regulation of inflammatory re	6	0.034134	2.34E-04
2	GO:0043410	positive regulation of MAPK cascade	6	0.034134	2.63E-04
	GO:0043536	positive regulation of blood vessel enc	4	0.022756	3.02E-04
	GO:0000165	MAPK cascade	9	0.0512	5.27E-04
	GO:0010575	positive regulation of vascular endoth	4	0.022756	8.75E-04
	GO:0050727	regulation of inflammatory response	5	0.028445	0.001003
	GO:0043552	positive regulation of phosphatidylino	4	0.022756	0.001317
	GO:0008286	insulin receptor signaling pathway	5	0.028445	0.002215
	GO:2001275	positive regulation of glucose import i	3	0.017067	0.003631
	GO:0001678	cellular glucose homeostasis	3	0.017067	0.004216
	GO:0030949	positive regulation of vascular endoth	3	0.017067	0.005509
	GO:0000187	activation of MAPK activity	5	0.028445	0.006861
	GO:0030512	negative regulation of transforming gr	4	0.022756	0.010324
	GO:0050796	regulation of insulin secretion	4	0.022756	0.011692
	GO:0045909	positive regulation of vasodilation	3	0.017067	0.017566
	GO:0046326	positive regulation of glucose import	3	0.017067	0.018735
	GO:0001666	response to hypoxia	12	0.142281	3.96E-12
	GO:0070374	positive regulation of ERK1 and ERK	9	0.106711	6.04E-08
	GO:0043410	positive regulation of MAPK cascade	7	0.082997	1.78E-07
	GO:0001525	angiogenesis	9	0.106711	3.88E-07
	GO:0043406	positive regulation of MAP kinase act	6	0.071141	1.03E-06
	GO:0000165	MAPK cascade	9	0.106711	1.31E-06
	GO:0045766	positive regulation of angiogenesis	7	0.082997	1.42E-06
	GO:0014068	positive regulation of phosphatidylino	6	0.071141	1.68E-06
	GO:0002040	sprouting angiogenesis	4	0.047427	6.14E-05
	GO:0043123	positive regulation of I-kappaB kinase	5	0.059284	0.001541
3	GO:0014066	regulation of phosphatidylinositol 3-k	4	0.047427	0.001809
	GO:0046627	negative regulation of insulin receptor	3	0.03557	0.00362
	GO:0043407	negative regulation of MAP kinase ac	3	0.03557	0.00554

	GO:0070373	negative regulation of ERK1 and ERK	3	0.03557	0.013919
	GO:0048010	vascular endothelial growth factor rec	3	0.03557	0.02094
	GO:0043116	negative regulation of vascular perme	2	0.023714	0.036546
	GO:0000187	activation of MAPK activity	3	0.03557	0.043393
	GO:1904707	positive regulation of vascular smooth	2	0.023714	0.045476
	GO:0008631	intrinsic apoptotic signaling pathway	2	0.023714	0.045476
	GO:1900745	positive regulation of p38MAPK casc	2	0.023714	0.048434
	GO:0030949	positive regulation of vascular endoth	2	0.023714	0.048434
5	GO:0051156	glucose 6-phosphate metabolic proces	2	0.090785	0.007125
	GO:0034599	cellular response to oxidative stress	2	0.090785	0.044804
6	GO:0006954	inflammatory response	10	0.140706	5.97E-07
	GO:0032930	positive regulation of superoxide anio	4	0.056283	1.49E-06
	GO:0008284	positive regulation of cell proliferatio	10	0.140706	3.29E-06
	GO:0042554	superoxide anion generation	4	0.056283	6.41E-06
	GO:0042310	vasoconstriction	4	0.056283	1.19E-05
	GO:0008217	regulation of blood pressure	5	0.070353	2.70E-05
	GO:0001666	response to hypoxia	6	0.084424	9.33E-05
	GO:0000302	response to reactive oxygen species	4	0.056283	1.54E-04
	GO:0048010	vascular endothelial growth factor rec	4	0.056283	9.43E-04
	GO:0043065	positive regulation of apoptotic proce	6	0.084424	0.001198
	GO:0043410	positive regulation of MAPK cascade	4	0.056283	0.001326
	GO:1902177	positive regulation of oxidative stress-	2	0.028141	0.010677
	GO:0070374	positive regulation of ERK1 and ERK	4	0.056283	0.011457
	GO:0003100	regulation of systemic arterial blood p	2	0.028141	0.013329
	GO:0000187	activation of MAPK activity	3	0.042212	0.033337
	GO:0043537	negative regulation of blood vessel en	2	0.028141	0.036885
7	GO:2000378	negative regulation of reactive oxyger	2	0.088652	0.018603
	GO:0019933	cAMP-mediated signaling	2	0.088652	0.033426
	GO:0007190	activation of adenylate cyclase activit	2	0.088652	0.035156
8	GO:1901687	glutathione derivative biosynthetic pr	5	0.281849	2.19E-09
	GO:0042178	xenobiotic catabolic process	4	0.225479	1.61E-08
	GO:0006749	glutathione metabolic process	5	0.281849	1.08E-07
	GO:0098869	cellular oxidant detoxification	3	0.169109	0.001509
10	GO:0045454	cell redox homeostasis	4	0.120048	9.99E-05
	GO:0034097	response to cytokine	3	0.090036	0.001724
	GO:0019430	removal of superoxide radicals	2	0.060024	0.014204
	GO:0008631	intrinsic apoptotic signaling pathway	2	0.060024	0.017725
13	GO:0055114	oxidation-reduction process	4	0.888889	4.36E-05
	GO:0006695	cholesterol biosynthetic process	3	0.098619	5.87E-04

14	GO:0010903	negative regulation of very-low-densit	2	0.065746	0.002856
	GO:0034375	high-density lipoprotein particle remo	2	0.065746	0.014203
	GO:0043691	reverse cholesterol transport	2	0.065746	0.017021
	GO:0019433	triglyceride catabolic process	2	0.065746	0.023567
	GO:0033344	cholesterol efflux	2	0.065746	0.023567
	GO:0070328	triglyceride homeostasis	2	0.065746	0.024499
	GO:0042157	lipoprotein metabolic process	2	0.065746	0.035615
15	GO:0061621	canonical glycolysis	2	0.43956	0.003094
	GO:0032869	cellular response to insulin stimulus	2	0.43956	0.00915
16	GO:0006635	fatty acid beta-oxidation	3	0.544465	6.71E-06
17	GO:0070059	intrinsic apoptotic signaling pathway	3	0.142315	2.44E-04
	GO:0030512	negative regulation of transforming gr	3	0.142315	9.21E-04
	GO:0048010	vascular endothelial growth factor rec	3	0.142315	0.001164
	GO:2000353	positive regulation of endothelial cell	2	0.094877	0.009962
	GO:0001525	angiogenesis	3	0.142315	0.010615
	GO:0035924	cellular response to vascular endothel	2	0.094877	0.016318
	GO:0071353	cellular response to interleukin-4	2	0.094877	0.017022
	GO:0042149	cellular response to glucose starvation	2	0.094877	0.020535
18	GO:0044255	cellular lipid metabolic process	3	0.22338	3.15E-04
	GO:0007179	transforming growth factor beta recep	3	0.22338	0.001581

Genes	Fold Enrichment	Bonferroni
EGFR, ICAM1, IL6, TNF, HSP90AA1, PTGS2, EDN1, ESR1	53.25158562	1.52E-17
MMP9, NFKB1, BCL2L1, PTEN, IL10, SRC, TIMP1, AKT1,	9.394125874	1.75E-15
EGFR, HRAS, TNF, CCL2, MAP2K1, GRB2, KITLG, KIT, (	13.40097155	2.92E-15
PIK3CG, IL6, TNF, CCL2, PTGS2, CSF1, TLR2, IL13, NFK	9.666778604	5.13E-13
EGFR, ICAM1, HRAS, IL6, TNF, CCL2, MAP2K1, TLR4, C	15.70161039	3.08E-12
PIK3CG, EGFR, HRAS, TNF, EDN1, KITLG, KIT, EGF, FG	28.46101695	9.71E-09
MAPK1, TNF, MAP2K1, CXCR4, MAPK14, MAPK3, IGF1,	18.54681393	1.03E-08
EGR1, CCL2, CREB1, TLR2, CXCL12, MMP2, TGFB1, LEI	13.31289641	1.31E-08
AKT1, EGFR, MAPK1, GRB2, MAPK3, PIK3CA, KITLG, K	21.52820513	1.76E-07
AKT1, EGFR, HRAS, HSP90AA1, GRB2, PIK3CA, SHC1, E	36.15502392	2.34E-07
PIK3CG, CCL2, PTGS2, PTEN, MMP2, KDR, LEP, JUN, HI	10.26824297	4.29E-07
ICAM1, AR, IL6, TNF, CD40LG, INS, TLR2, IL1B, NFKB1,	13.77334245	2.33E-06
LEP, INS, F2, IGF1, JAK2, CAT, KIT, CCL5, KDR	21.13678322	2.09E-05
LEP, EGR1, IL6, TNFSF10, TLR2, CAT, MTOR, IRS1, IL10	20.50583446	2.67E-05
ACTB, CDC42, HSP90AA1, CCL2, MAPK14, RHOA, PIK3(	19.08181818	4.77E-05
EGFR, TNFRSF1A, CCL2, SERPINE1, TLR2, TLR4, JAK2,	18.82042341	5.32E-05
LEP, AR, IL6, HRAS, INS, IGF1, KIT, KDR, CTNNB1	16.96161616	1.22E-04
TNF, PTGS2, RHOA, TLR2, IL1B, TLR4	43.61558442	4.55E-04
LEP, ACE, PTGS2, REN, HMOX1, EDN1, PPARG, NOS3	18.78825175	4.72E-04
MMP9, MDM2, JAK2, MMP2, IL10	50.88484848	0.005004646
FOS, CCL2, JUN, CREB1, RHOA, PARP1, TGFB1, SRC	13.2743083	0.005132232
LEP, AKT1, TNF, INS, MAPK14, PIK3CA, GAPDH	15.94898236	0.010834942
ICAM1, IL17A, IL6, CCL2, EDN1, NFKB1, CCL5	15.05044814	0.015192671
TNF, SERPINE1, TLR2, IL1B, TLR4	29.35664336	0.050679207
AKT1, INS, MAPK14, IGF1, IRS1	25.44242424	0.089217841
LEP, IL6, HMOX1, FGF2, CTNNB1	24.62170088	0.101260388
LEP, AKT1, IL6, INS, PPARG, IRS1, STAT3	10.580018	0.109296729
AKT1, EGFR, ICAM1, PTGS2, ABL1	23.85227273	0.114325882
LEP, INS, IRS1, SRC	46.97062937	0.154474934
AKT1, IGF1R, IGF1, IRS1	43.61558442	0.191483306
F3, HMOX1, SERPINE1, IL1B, NOS3, FGF2, KDR	9.29201581	0.212433769
AKT1, CCL2, PPARG, PARP1, IRS1, SRC	11.89515939	0.281871036
AKT1, IGF1R, GRB2, INS, SHC1, IRS1	11.74265734	0.29670117
IL6, TNF, TLR2, IL1B, TLR4	16.96161616	0.375922827
VCAM1, AKT1, MAPK14, KDR	26.5486166	0.628887445
EGFR, INS, HMOX1, NOS3	21.05579937	0.863105255
LEP, TNF, IFNG, IL1B, CCL5	11.39213026	0.886381125
CALM3, CALM2, CTNNB1, CALM1	15.65687646	0.991328468
TNFRSF1A, TNF, NFKB1, TLR4	10.1769697	0.999999903
EGR1, CASP3, PTEN, TGFB1	8.979679144	1
BAX, FASLG	152.6545455	1
TNF, TLR4, IL10	16.35584416	1
IL6, CCL2	76.32727273	1
CREB1, TGFB1	61.06181818	1

EDN1, ABL1, TGFB1	9.34619666	1
LEP, IL4, APOE, CAT	8.979679144	1
APOE, EGF	61.06181818	1
CXCL5, IL18, C5, CRP, CXCL9, BDKRB1, PF4, BDKRB2,	10.05263741	1.38E-15
PRKCA, C5AR1, C5, HGF, CXCR3, SIRT1, ECM1, VEGFC	15.95147972	4.40E-08
PRKCA, C5AR1, PDGFB, CCL4, GAS6, PTPN11, VEGFB,	11.2887395	4.69E-07
PRKCA, IL18, CXCR3, ECM1, SIRT1, TGFB2, VEGFB, VE	8.858876286	9.15E-06
CCR2, TEK, APOH, HRG, PF4, CXCR3, SPARC, STAT1, C	20.48359989	1.84E-05
SELP, PTK2, PDGFB, IL18, TEK, PDGFRB, HGF, SIRT1, T	19.53820297	2.70E-05
HIF1A, SMAD4, SMAD3, TGFB2	141.1092437	0.002146278
VEGFB, VEGFC, CYBB, PTK2, HSPB1, PIK3R1, PXN	13.71895425	0.017941655
PTK2, PDGFB, SMAD4, SMAD3, SMAD2, PXN, TGFB2	10.73657289	0.071070251
AGTR1, IL18, CCR2, ITGA2, CCL4, TLR9	11.59802003	0.227184091
SP1, FOXO1, STAT1, INSR, PIK3R1, AKT2	10.99552548	0.282171762
PDGFB, LCK, PDGFRB, HBEGF, PIK3R1, PTPN11	10.85455721	0.296905719
TNFRSF1B, IL2RA, SOCS3, TEK, SMAD3, HGF	10.71715775	0.312007364
LIF, TNFRSF1B, PDGFB, IGF2, FAS, INSR	10.45253657	0.343245199
PRKCA, VEGFC, PDGFB, HSPB1	29.7072092	0.382427055
PTK2, IL2RA, PDGFB, CCR5, IL18, TEK, PDGFRB, RAF1,	4.847264096	0.568516751
HIF1A, C5AR1, C5, IL1A	20.90507314	0.752622338
AGTR1, IL1R1, XIAP, GGT1, AHSG	11.19914633	0.798305927
PTK2, PDGFB, TEK, PDGFRB	18.20764435	0.877986575
FOXO1, IGF2, INSR, PIK3R1, AKT2	9.04546434	0.970952763
PIK3R1, AKT2, PTPN11	32.56367162	0.99698789
FOXO1, SIRT1, PIK3R1	30.23769508	0.998821608
VEGFB, HIF1A, VTN	26.45798319	0.999851838
C5AR1, C5, HGF, INSR, PTPN11	6.593889892	0.999983082
SMAD3, SMAD2, HSPA5, SIRT1	8.819327731	0.999999936
PRKCA, HNF4A, CNR1, NOS2	8.42443246	0.999999993
AGTR2, NOS2, APLN	14.59750797	1
INSR, PIK3R1, AKT2	14.11092437	1
PLAT, BMP2, ETS1, PGF, APAF1, THBS1, MMP14, ADIP	22.10443177	4.81E-09
BMP4, GCG, BMP2, CCL3, CD36, ERBB4, PDGFA, ANGP	16.29412399	7.32E-05
FGFR1, BMP2, FLT1, PDGFA, IL6R, TIMP2, IGFBP3	27.38038668	2.16E-04
FGFR1, FLT1, PDGFA, PGF, ANGPT1, MMP14, FGF1, FN	12.7868686	4.71E-04
FGFR1, FLT1, PDGFA, ELANE, FGF1, CSK	32.22001919	0.001250502
FGFR1, MAP3K5, CCL3, MAPK12, ERBB4, PDGFA, ANG	10.88347976	0.001589965
FLT1, ETS1, PGF, THBS1, FGF1, ENG, PRKCB	19.28531583	0.001726972
FGFR1, FLT1, ERBB4, PDGFA, AGT, ANGPT1	29.24586357	0.002033682
PGF, ANGPT1, THBS1, ENG	50.69283019	0.071767747
CD36, GJA1, FADD, ADIPOQ, PRKCB	9.839446853	0.845936503
FGFR1, ERBB4, PDGFA, FGF1	16.24770198	0.888794453
SOCS1, PTPN1, PRKCB	32.77553676	0.98770715
BMP4, PTPN1, ADIPOQ	26.40251572	0.998815463

PTPN1, CSK, ADIPOQ	16.38776838	0.999999959
FLT1, MAPK12, PGF	13.20125786	1
TJP1, ANGPT1	52.80503145	1
BMP2, THBS1, FGF1	8.883089402	1
MAP3K5, AGT	42.24402516	1
MAP3K5, SOD2	42.24402516	1
MAP3K5, BMP2	39.60377358	1
FLT1, PRKCB	39.60377358	1
GPI, G6PD	258.3384615	0.674588557
G6PD, TXN2	40.36538462	0.999250904
NOX4, CYBA, HMGB1, PRKCQ, TNFRSF11B, NOX1, RA(	9.631754044	4.92E-04
CYBA, F2RL1, SOD1, PRKCD	162.2415459	0.001228638
OSM, EDNRB, EDN3, CNTF, S100B, ITGAV, EDN2, FLT4,	7.833551036	0.002707554
NOX4, CYBA, NOX1, SOD1	104.2981366	0.005263722
EDNRA, EDNRB, EDN3, EDN2	85.89258312	0.009756083
EDNRA, EDNRB, UTS2, NOX1, SOD1	28.08026756	0.022007285
EDNRA, NOX4, CYBA, PPARA, UTS2, ANGPT2	12.73407482	0.073907626
NOX4, CYBA, GSR, SOD1	37.44035674	0.118780707
CYBA, ITGAV, FLT4, RAC1	20.28019324	0.539806141
NOX4, HMGB1, S100B, RAC1, SOD1, ITGB1	7.300869565	0.627158195
OSM, HMGB1, TNFRSF11B, FLT4	18.02683843	0.664454025
NOX1, SOD1	182.5217391	0.999854406
NOX4, HMGB1, FLT4, F2RL1	8.343850932	0.999923936
EDN3, EDN2	146.0173913	0.999984012
MAPK10, SOD1, GHR	10.23486388	1
HMGB1, ANGPT2	52.14906832	1
SIRT5, SIRT3	99.95238095	0.940201579
GLP1R, ADCYAP1	55.23684211	0.993900868
GLP1R, ADCYAP1	52.475	0.995338089
GSTM1, GSTA1, GSTM2, GSTA3, GSTO1	254.4242424	2.74E-07
GSTM1, GSTM2, GSTO1, CRYZ	639.6952381	2.01E-06
GSTM1, GSTA1, GSTM2, GSTA3, GSTO1	99.95238095	1.35E-05
GSTA1, GSTM2, GSTO1	47.97714286	0.172040026
GPX1, PDIA3, PRDX4, PRDX1	41.53865182	0.038411252
TYMS, PML, RARA	46.13186813	0.491632884
NQO1, PRDX1	133.2698413	0.996331033
GPX1, PML	106.615873	0.999097583
MAOA, MAOB, TPH1, AOC3	28.36486486	0.001307309
APOA1, HMGCR, PRKAA2	77.98142415	0.163359314

APOA1, APOC3	658.5098039	0.580818508
APOA1, APOC3	131.7019608	0.987077838
APOA1, APOC3	109.751634	0.99458748
APOA1, APOC3	79.02117647	0.999289954
APOA1, APOC3	79.02117647	0.999289954
APOA1, APOC3	75.98190045	0.999468822
APOA1, APOC3	51.9876161	0.999983703
GCK, PKLR	430.5641026	0.094415384
GCK, PKLR	145.3852814	0.25484035
ACADM, ACAT1, ACAA1	381.6363636	2.42E-04
ATF4, XBP1, CASP12	117.4265734	0.083709261
PDPK1, XBP1, TGFBR1	60.54807692	0.280946704
NRP1, XBP1, ITGB3	53.82051282	0.340905979
COL18A1, XBP1	184.5274725	0.972245968
COL18A1, NRP1, XBP1	17.37702656	0.978084679
NRP1, XBP1	112.3210702	0.997233563
HSP90AB1, XBP1	107.6410256	0.997858982
ATF4, XBP1	89.08222812	0.999405682
NR1H2, RXRA, CARM1	102.3902439	0.06352316
ACVRL1, TGFBR2, GDF15	45.63043478	0.280495415
