# Supplementary material for: Exploring the Pharmacological Mechanism of Liuwei Dihuang Decoction for Diabetic Retinopathy: A Systematic Biological Strategy-Based Research
Source: Evid Based Complement Alternat Med. 2021 Aug 2;2021:5544518. doi: 10.1155/2021/5544518 (PMC8356007; doi:10.1155/2021/5544518)
Supplement: Supplementary Materials — Table S1: compound targets for each compounds. Table S2: known targets for each compounds. Table S3: DR genes. Table S4: enrichment analysis of clusters based on Gene Ontology (GO) annotation of DR PPI network. Table S5: pathway enrichment analysis of DR PPI network. Table S6: enrichment analysis of clusters based on Gene Ontology (GO) annotation of LDD-DR PPI network. Table S7: pathway enrichment analysis of LDD-DR PPI network. Table S8: enrichment analysis of clusters based on Gene Ontology (GO) annotation of LDD known target-DR network. Table S9: pathway enrichment analysis of LDD known target-DR network. [file 5544518.f1.zip › 5544518.f1/Table S7.pdf]

Table S7 Pathway enrichment analysis

Term	Pathway	Count	%	Pvalue
hsa04151	PI3K-Akt signaling pathway	38	0.068771	4.18E-07
hsa04910	Insulin signaling pathway	29	0.052483	7.18E-12
hsa04068	FoxO signaling pathway	25	0.045244	3.52E-09
hsa04010	MAPK signaling pathway	25	0.045244	3.41E-04
hsa04722	Neurotrophin signaling pathway	24	0.043434	1.91E-09
hsa04012	ErbB signaling pathway	19	0.034385	3.34E-08
hsa04931	Insulin resistance	19	0.034385	1.03E-06
hsa04370	VEGF signaling pathway	18	0.032576	6.10E-10
hsa04066	HIF-1 signaling pathway	16	0.028956	1.87E-05
hsa03320	PPAR signaling pathway	15	0.027146	1.02E-06
hsa04668	TNF signaling pathway	15	0.027146	2.54E-04
hsa04922	Glucagon signaling pathway	14	0.025337	4.05E-04
hsa04620	Toll-like receptor signaling pathway	14	0.025337	7.89E-04
hsa04152	AMPK signaling pathway	13	0.023527	0.008325
hsa04930	Type II diabetes mellitus	9	0.016288	0.001114
hsa04150	mTOR signaling pathway	9	0.016288	0.003849
hsa04210	Apoptosis	8	0.014478	0.019426

Genes	Fold Enrichment	Bonferroni
HSP90AB1, FGFR1, HRAS, GRB2, PGF, BCL2L1, KIT, AK	2.468035689	1.12E-04
HRAS, GRB2, PDE3B, HK1, AKT1, PDPK1, PRKACA, INS	4.708752301	1.93E-09
HRAS, GRB2, TGFB2, AKT1, IGF1R, PDPK1, INSR, PIK3F	4.180441441	9.42E-07
FGFR1, HRAS, GRB2, HSPA1A, MAPKAPK2, TGFB2, AK	2.21414685	0.087447821
PIK3CG, HRAS, BRAF, MAP2K1, GRB2, RAF1, MAPK10,	4.481433225	5.12E-07
PIK3CG, EGFR, HRAS, ERBB4, BRAF, MAP2K1, GRB2, R	4.893519039	8.95E-06
PIK3CG, PPARA, MAPK10, PPP1CC, PTPN11, PCK1, AKT	3.942001448	2.76E-04
PIK3CG, HRAS, MAP2K1, RAF1, MAPKAPK2, SRC, KDR	6.611950659	1.64E-07
EGFR, PIK3CG, MAP2K1, HK1, PDHB, AKT1, IGF1R, MA	3.734527687	0.004988985
PPARA, PPARD, ACADM, RXRB, RXRA, PPARG, MMP1,	5.016529729	2.74E-04
PIK3CG, MAP2K1, MMP9, MAPK10, MMP3, CCL5, AKT1	3.141191513	0.065777151
LDHB, PPARA, PFKFB1, PDE3B, PDHB, PCK1, AKT1, GC	3.168690159	0.102981338
PIK3CG, AKT1, MAPK1, CTSK, MAP2K1, MAPK12, MAP	2.959437035	0.190580928
PIK3CG, AKT1, IGF1R, PDPK1, HMGCR, PFKFB1, PPAR	2.36823707	0.893597383
PIK3CG, MAPK1, GCK, PKLR, HK1, MAPK8, MAPK10, I	4.201343648	0.258154697
PIK3CG, AKT1, MAPK1, PDPK1, EIF4E, BRAF, RHEB, PI	3.476974054	0.64426038
PIK3CG, AKT1, CASP3, XIAP, APAF1, BCL2L1, PIK3R1,	2.891247242	0.994791391
