# Supplementary material for: Exploring the Pharmacological Mechanism of Liuwei Dihuang Decoction for Diabetic Retinopathy: A Systematic Biological Strategy-Based Research
Source: Evid Based Complement Alternat Med. 2021 Aug 2;2021:5544518. doi: 10.1155/2021/5544518 (PMC8356007; doi:10.1155/2021/5544518)
Supplement: Supplementary Materials — Table S1: compound targets for each compounds. Table S2: known targets for each compounds. Table S3: DR genes. Table S4: enrichment analysis of clusters based on Gene Ontology (GO) annotation of DR PPI network. Table S5: pathway enrichment analysis of DR PPI network. Table S6: enrichment analysis of clusters based on Gene Ontology (GO) annotation of LDD-DR PPI network. Table S7: pathway enrichment analysis of LDD-DR PPI network. Table S8: enrichment analysis of clusters based on Gene Ontology (GO) annotation of LDD known target-DR network. Table S9: pathway enrichment analysis of LDD known target-DR network. [file 5544518.f1.zip › 5544518.f1/Table S8.pdf]

Table S8 Enrichment analysis of clusters based on Gene Ontology (GO) annotation

Cluster	Term	Pathway	Count	%	Pvalue
1	GO:0006954	inflammatory response	24	0.136364	9.84E-16
	GO:0045766	positive regulation of angiogenesis	13	0.073864	2.03E-11
	GO:0001525	angiogenesis	16	0.090909	3.26E-11
	GO:0001666	response to hypoxia	13	0.073864	2.24E-09
	GO:0008217	regulation of blood pressure	9	0.051136	1.38E-08
	GO:0043066	negative regulation of apoptotic proce	17	0.096591	8.30E-08
	GO:0014068	positive regulation of phosphatidylino	8	0.045455	2.95E-07
	GO:0043950	positive regulation of cAMP-mediatec	5	0.028409	9.90E-07
	GO:0032909	regulation of transforming growth fac	4	0.022727	1.25E-06
	GO:0070374	positive regulation of ERK1 and ERK	10	0.056818	3.22E-06
	GO:0016525	negative regulation of angiogenesis	7	0.039773	4.07E-06
	GO:0042593	glucose homeostasis	8	0.045455	5.95E-06
	GO:0030816	positive regulation of cAMP metaboli	4	0.022727	6.17E-06
	GO:0000165	MAPK cascade	11	0.0625	1.28E-05
	GO:0071363	cellular response to growth factor stin	6	0.034091	1.36E-05
	GO:0008286	insulin receptor signaling pathway	7	0.039773	1.56E-05
	GO:0032869	cellular response to insulin stimulus	6	0.034091	1.84E-04
	GO:0019229	regulation of vasoconstriction	4	0.022727	3.28E-04
	GO:0010575	positive regulation of vascular endoth	4	0.022727	8.12E-04
	GO:0045909	positive regulation of vasodilation	4	0.022727	0.001004
	GO:0046627	negative regulation of insulin receptoı	4	0.022727	0.001004
	GO:0050796	regulation of insulin secretion	5	0.028409	0.001148
	GO:0043552	positive regulation of phosphatidylino	4	0.022727	0.001223
	GO:0001974	blood vessel remodeling	4	0.022727	0.001343
	GO:0014066	regulation of phosphatidylinositol 3-k	5	0.028409	0.002016
	GO:0038128	ERBB2 signaling pathway	4	0.022727	0.002217
	GO:0046628	positive regulation of insulin receptor	3	0.017045	0.003452
	GO:0007179	transforming growth factor beta recep	5	0.028409	0.003671
	GO:0036092	phosphatidylinositol-3-phosphate bios	4	0.022727	0.004585
	GO:0030949	positive regulation of vascular endoth	3	0.017045	0.00524
	GO:0000187	activation of MAPK activity	5	0.028409	0.006271
	GO:0007173	epidermal growth factor receptor sign	4	0.022727	0.006663
	GO:0017015	regulation of transforming growth fac	3	0.017045	0.00815
	GO:0050727	regulation of inflammatory response	4	0.022727	0.009222
	GO:0030512	negative regulation of transforming gr	4	0.022727	0.009628
	GO:0009749	response to glucose	4	0.022727	0.011355
	GO:0048010	vascular endothelial growth factor rec	4	0.022727	0.01325
	GO:0050729	positive regulation of inflammatory re	4	0.022727	0.01375
	GO:0043410	positive regulation of MAPK cascade	4	0.022727	0.018136
	GO:0045907	positive regulation of vasoconstrictioı	3	0.017045	0.020173
	GO:0070374	positive regulation of ERK1 and ERK	14	0.13428	1.70E-13
	GO:0043410	positive regulation of MAPK cascade	11	0.105505	7.63E-13
	GO:0008284	positive regulation of cell proliferatioı	18	0.172645	2.42E-12

	GO:0045907	positive regulation of vasoconstriction	6	0.057548	1.61E-07
	GO:0006954	inflammatory response	12	0.115097	2.77E-07
	GO:0045766	positive regulation of angiogenesis	8	0.076731	3.64E-07
	GO:0001666	response to hypoxia	9	0.086323	4.01E-07
	GO:0043123	positive regulation of I-kappaB kinase	8	0.076731	3.51E-06
	GO:0008283	cell proliferation	10	0.095914	1.41E-05
	GO:0042310	vasoconstriction	4	0.038366	3.97E-05
	GO:0043536	positive regulation of blood vessel end	4	0.038366	5.62E-05
	GO:0019229	regulation of vasoconstriction	4	0.038366	6.59E-05
	GO:0043406	positive regulation of MAP kinase act	5	0.047957	8.93E-05
	GO:0001994	norepinephrine-epinephrine vasoconst	3	0.028774	9.36E-05
	GO:0014068	positive regulation of phosphatidylino	5	0.047957	1.30E-04
**2**	GO:0001525	angiogenesis	7	0.06714	2.60E-04
	GO:2000778	positive regulation of interleukin-6 se	3	0.028774	0.002052
	GO:0048010	vascular endothelial growth factor rec	4	0.038366	0.002975
	GO:0050729	positive regulation of inflammatory re	4	0.038366	0.003094
	GO:0014066	regulation of phosphatidylinositol 3-k	4	0.038366	0.003731
	GO:0050728	negative regulation of inflammatory re	4	0.038366	0.003867
	GO:0000165	MAPK cascade	6	0.057548	0.003911
	GO:0001937	negative regulation of endothelial cell	3	0.028774	0.00594
	GO:0045909	positive regulation of vasodilation	3	0.028774	0.00594
	GO:0003056	regulation of vascular smooth muscle	2	0.019183	0.015866
	GO:0009749	response to glucose	3	0.028774	0.030172
	GO:0050702	interleukin-1 beta secretion	2	0.019183	0.031484
	GO:0002544	chronic inflammatory response	2	0.019183	0.03535
	GO:0032869	cellular response to insulin stimulus	3	0.028774	0.037881
	GO:0007252	I-kappaB phosphorylation	2	0.019183	0.046858
	GO:0042312	regulation of vasodilation	2	0.019183	0.046858
**3**	GO:0043116	negative regulation of vascular perme	2	0.197824	0.004281
	GO:0042311	vasodilation	2	0.197824	0.004993
	GO:0000302	response to reactive oxygen species	3	0.108225	6.18E-04
**5**	GO:0042593	glucose homeostasis	3	0.108225	0.004069
	GO:0001678	cellular glucose homeostasis	2	0.07215	0.013262
	GO:0015758	glucose transport	2	0.07215	0.030998
**6**	GO:1901687	glutathione derivative biosynthetic pro	4	0.600601	3.89E-08
	GO:0006749	glutathione metabolic process	4	0.600601	6.98E-07
	GO:0008284	positive regulation of cell proliferation	9	0.17192	1.49E-06
	GO:0043410	positive regulation of MAPK cascade	5	0.095511	1.43E-05
	GO:0045766	positive regulation of angiogenesis	5	0.095511	5.69E-05
	GO:0016525	negative regulation of angiogenesis	4	0.076409	2.00E-04
	GO:0001666	response to hypoxia	5	0.095511	2.69E-04
**7**	GO:0001525	angiogenesis	5	0.095511	7.19E-04

	GO:0046627	negative regulation of insulin receptor	3	0.057307	0.001298
	GO:0070374	positive regulation of ERK1 and ERK	4	0.076409	0.004036
	GO:0050729	positive regulation of inflammatory re	3	0.057307	0.00799
	GO:0032930	positive regulation of superoxide anio	2	0.038204	0.016497
	GO:0010575	positive regulation of vascular endoth	2	0.038204	0.048704
	GO:0042554	superoxide anion generation	4	0.126823	4.44E-07
	GO:0048010	vascular endothelial growth factor rec	5	0.158529	1.15E-06
	GO:0050900	leukocyte migration	5	0.158529	9.45E-06
	GO:0000302	response to reactive oxygen species	4	0.126823	1.09E-05
	GO:0071333	cellular response to glucose stimulus	4	0.126823	2.62E-05
	GO:0006954	inflammatory response	6	0.190235	5.11E-05
**9**	GO:0001938	positive regulation of endothelial cell	4	0.126823	6.14E-05
	GO:0001666	response to hypoxia	4	0.126823	9.07E-04
	GO:0071560	cellular response to transforming grov	3	0.095117	0.001382
	GO:0043065	positive regulation of apoptotic proces	4	0.126823	0.004425
	GO:1902177	positive regulation of oxidative stress-	2	0.063412	0.004519
	GO:0001525	angiogenesis	3	0.095117	0.02588
	GO:0070371	ERK1 and ERK2 cascade	2	0.063412	0.026823
	GO:0072593	reactive oxygen species metabolic pro	2	0.063412	0.038888
**10**	GO:0010906	regulation of glucose metabolic proce	2	0.072727	0.020767
	GO:0000302	response to reactive oxygen species	2	0.072727	0.036536
	GO:0038061	NIK/NF-kappaB signaling	2	0.414938	0.007846
**11**	GO:0090263	positive regulation of canonical Wnt s	2	0.414938	0.014242
	GO:0090090	negative regulation of canonical Wnt s	2	0.414938	0.01932
	GO:0000165	MAPK cascade	2	0.414938	0.030963
**12**	GO:0007190	activation of adenylate cyclase activity	3	0.623701	5.53E-06
	GO:0019933	cAMP-mediated signaling	2	0.4158	0.004521
	GO:0008217	regulation of blood pressure	3	0.525394	1.46E-04
**13**	GO:0043114	regulation of vascular permeability	2	0.350263	0.002974
	GO:0042312	regulation of vasodilation	2	0.350263	0.003568
	GO:0016525	negative regulation of angiogenesis	2	0.350263	0.018328

l

Genes	Fold Enrichment	Bonferroni
KNG1, SELP, C5AR1, PTGER3, CXCL5, IL18, C5, CRP, CX	9.166772814	1.63E-12
C5AR1, FLT1, C5, CXCR3, SIRT1, ECM1, HIF1A, HMOX1	16.36401799	3.30E-08
CAV1, FLT1, IL18, CXCR3, ECM1, SIRT1, TGFB2, VEGFI	10.38626875	5.31E-08
VEGFB, CAV1, HIF1A, EP300, HMOX1, TEK, SMAD4, SM	10.94105854	3.65E-06
ACE, AGTR2, NPY, REN, HMOX1, PPARG, GNB3, POMC	20.04350133	2.25E-05
CDK1, MCL1, SOCS3, SMAD3, FOXO1, RPS6KB1, SIRT1,	5.40856385	1.35E-04
SELP, FLT1, ERBB3, IL18, TEK, PDGFRB, SIRT1, TGFB2	17.81644562	4.80E-04
OPRM1, CXCL9, PF4, CXCR3, CXCL11	60.31609195	0.001611162
HIF1A, SMAD4, SMAD3, TGFB2	144.7586207	0.002025571
OPRM1, VEGFB, C5AR1, FGB, SERPINF2, CTGF, TEK, PI	8.271921182	0.005228827
CCR2, TEK, APOH, HRG, PF4, CXCR3, SPARC	16.34371524	0.00660404
HIF1A, HNF4A, CNR1, PPARG, ADRA2A, POMC, IRS1, IN	11.46602936	0.009635779
CXCL9, PF4, CXCR3, CXCL11	96.50574713	0.0099868
MAP3K5, CAV1, CCR5, GRB2, ERBB3, IL18, TEK, PDGFI	6.077652014	0.02054475
SHC1, RPS6KB1, SPARC, INSR, GAS6, OPRD1	19.30114943	0.021822835
GRB2, FOXO1, IGF2, SHC1, IRS1, INSR, AKT2	12.99115827	0.025011299
SP1, PPARG, FOXO1, IRS1, INSR, AKT2	11.27989252	0.259049037
ACE, ADRA2A, ADRA2C, BDKRB2	28.95172414	0.413452702
HIF1A, C5AR1, C5, IL1A	21.44572158	0.733456543
AGTR2, HMOX1, NOS2, APLN	19.9667063	0.805153606
SOCS3, RPS6KB1, IRS1, AHSG	19.9667063	0.805153606
HNF4A, CNR1, ADRA2A, ADRA2C, NOS2	10.80288214	0.845812645
FLT1, TEK, PDGFRB, IRS1	18.6785317	0.863614494
ACE, AGTR2, CCR2, TGFB2	18.09482759	0.887813128
GRB2, ERBB3, PDGFRB, HBEGF, IRS1	9.279398762	0.962595112
GRB2, ERBB3, HBEGF, SHC1	15.23774955	0.9730279
IGF2, IRS1, SIRT1	33.40583554	0.996410882
SMAD4, SMAD3, SMAD2, PXN, TGFB2	7.867316342	0.997490466
GRB2, IRS1, ATM, TLR9	11.81703026	0.999436196
VEGFB, HIF1A, FLT1	27.14224138	0.999807158
CDK1, C5AR1, C5, SHC1, INSR	6.764421528	0.99996432
GRB2, HBEGF, SHC1, PXN	10.33990148	0.999981243
SMAD4, SMAD3, SMAD2	21.7137931	0.999998364
IL1R1, GGT1, SELE, AHSG	9.191023536	0.999999718
CAV1, SMAD3, SMAD2, SIRT1	9.047413793	0.999999855
HNF4A, CTGF, SMAD2, RPS6KB1	8.515212982	0.999999992
VEGFB, FLT1, HSPB1, PXN	8.042145594	1
IL18, CCR2, SERPINE1, TLR9	7.931979216	1
FLT1, IGF2, FAS, INSR	7.148573861	1
CAV1, FGB, ADRA2C	13.57112069	1
PRKCA, BMP4, HMGB1, CCL3, PDGFB, F2RL1, CCL4, GC	19.75529412	2.03E-10
LIF, FGFR1, HMGB1, TNFRSF1B, PDGFB, ADRA1B, ADF	33.5352215	9.12E-10
FGFR1, EDN3, PDGFB, PGF, TNC, EDN2, IL6R, LIF, EDNI	9.538500379	2.89E-09

ADRA1B, ADRA1A, GJA1, ADRA1D, HTR2A, F2R	46.30147059	1.92E-04
IFNA2, HMGB1, CYBB, TNFRSF1B, CCL3, IL2RA, F2RL1	7.818717989	3.31E-04
PRKCA, CYBB, UTS2, ETS1, PGF, THBS1, FGF1, ENG	17.17851662	4.35E-04
EDNRA, PLAT, UTS2, ETS1, PGF, THBS1, MMP14, ADIP(	12.92134063	4.80E-04
CD36, F2RL1, GJA1, FADD, IKBKB, ADIPOQ, CHUK, F2F	12.27036902	0.00418259
EDNRA, GCG, CHRM5, IL2RA, CHRM3, CHRM1, TXN, A	6.74702668	0.016721949
EDNRA, EDNRB, EDN3, EDN2	58.10380623	0.046296012
PRKCA, PDGFB, ANGPT1, THBS1	51.9876161	0.064957635
EDN3, AGTR1, EDN2, ADRA1A	49.38823529	0.075766757
EDN3, FGFR1, PDGFB, FGF1, HTR2A	20.92721834	0.101236857
ADRA1B, ADRA1A, ADRA1D	185.2058824	0.105844409
FGFR1, PDGFB, F2RL1, ANGPT1, F2R	18.99547511	0.144403286
PRKCA, FGFR1, PGF, ANGPT1, MMP14, FGF1, FN1	7.75151675	0.267521979
HMGB1, F2RL1, F2R	43.57785467	0.914107317
CYBB, NCF1, PGF, PIK3R1	13.71895425	0.971570003
AGTR1, CCL3, ETS1, CCL4	13.53102337	0.975340906
FGFR1, PDGFB, FGF1, PIK3R1	12.66365008	0.988514242
TNFRSF1B, IL2RA, ETS1, ADIPOQ	12.50335071	0.99024674
FGFR1, CCL3, IL2RA, PDGFB, ANGPT1, FGF1	5.655141446	0.990750979
GJA1, THBS1, ENG	25.54563895	0.999190634
UTS2, F2RL1, GJA1	25.54563895	0.999190634
CHRM3, CHRM1	123.4705882	0.999999995
GJA1, THBS1, ADIPOQ	10.89446367	1
CD36, F2RL1	61.73529412	1
GJA1, THBS1	54.87581699	1
SLC2A4, ADIPOQ, PIK3R1	9.621084798	1
IKBKB, CHUK	41.15686275	1
AGTR1, UTS2	41.15686275	1
ADM, ADORA2A	399.8095238	0.496609191
DRD1, ADORA2A	342.6938776	0.551045735
GPX1, P4HB, TXN2	75.98190045	0.160994803
HNF1A, GCK, PRKAA2	29.33954572	0.685836946
GCK, HK2	141.1092437	0.977443802
GCK, HK2	59.86452763	0.999869319
GSTM1, GSTM2, GSTT1, GSTP1	436.1558442	3.08E-06
GSTM1, GSTM2, GSTT1, GSTP1	171.3469388	5.51E-05
OSM, FGFR2, COL18A1, VEGFC, PDGFA, FLT4, ADAM1'	10.13465665	0.001091654
OSM, FGFR2, TNFRSF11B, PDGFA, FLT4	32.39197531	0.01043889
VEGFC, CX3CR1, CX3CL1, ANGPT2, PRKCB	22.81521739	0.040879849
AGT, CX3CR1, ANGPT2, CXCL10	33.85483871	0.136462719
VEGFC, ADAM17, ANGPT2, SRF, PRKCB	15.25436047	0.179270597
FGFR2, COL18A1, VEGFC, PDGFA, ANGPT2	11.76569507	0.409713844

PTPN1, PRKCD, PRKCB	54.28448276	0.614105014
FGFR2, PDGFA, FLT4, CX3CL1	11.99428571	0.948405052
LPL, AGT, CX3CL1	21.56506849	0.997205061
AGT, PRKCD	116.6111111	0.999994934
ATF4, FLT4	38.87037037	1
NOX4, CYBA, NOX1, SOD1	239.8857143	2.47E-04
CYBA, NRP1, ITGAV, RAC1, ITGB3	58.30555556	6.39E-04
ITGAL, THBD, ITGAV, ITGB2, ITGB3	34.40983607	0.005248458
NOX4, CYBA, SOD1, PPARGC1A	86.11282051	0.006074358
NOX4, CYBA, UCP2, PPARGC1A	64.58461538	0.014491619
NOX4, CYBA, ITGAL, NOX1, RAC1, ITGB2	13.29182058	0.028059001
CYBA, NRP1, TGFBR1, ITGB3	48.67246377	0.033612274
NOX4, CYBA, PPARA, UCP2	19.5255814	0.396742536
NOX4, TGFBR1, PPARGC1A	51.40408163	0.537109378
NOX4, NCOA1, RAC1, SOD1	11.19466667	0.915423814
NOX1, SOD1	419.8	0.919750532
NRP1, ITGAV, NOX1	11.29506726	0.999999546
APOA1, ITGAV	69.96666667	0.999999735
NOX4, SOD1	47.97714286	1
NCOA2, ACACB	89.79679144	0.99308153
TXNRD2, SOD3	50.6546003	0.999852435
PSMD3, PSMD9	169.6161616	0.240945238
PSMD3, PSMD9	93.28888889	0.394710964
PSMD3, PSMD9	68.67893661	0.494814743
PSMD3, PSMD9	42.72773537	0.66740508
ADRB2, GLP1R, ADCYAP1	419.8	3.93E-04
GLP1R, ADCYAP1	294.5964912	0.275096861
NPPB, NPR1, NPPA	129.1692308	0.015547814
NPPB, NPR1	559.7333333	0.272933242
NPPB, NPR1	466.4444444	0.317850999
NPPB, NPR1	90.27956989	0.861825418
