# Supplementary material for: Exploring the Pharmacological Mechanism of Liuwei Dihuang Decoction for Diabetic Retinopathy: A Systematic Biological Strategy-Based Research
Source: Evid Based Complement Alternat Med. 2021 Aug 2;2021:5544518. doi: 10.1155/2021/5544518 (PMC8356007; doi:10.1155/2021/5544518)
Supplement: Supplementary Materials — Table S1: compound targets for each compounds. Table S2: known targets for each compounds. Table S3: DR genes. Table S4: enrichment analysis of clusters based on Gene Ontology (GO) annotation of DR PPI network. Table S5: pathway enrichment analysis of DR PPI network. Table S6: enrichment analysis of clusters based on Gene Ontology (GO) annotation of LDD-DR PPI network. Table S7: pathway enrichment analysis of LDD-DR PPI network. Table S8: enrichment analysis of clusters based on Gene Ontology (GO) annotation of LDD known target-DR network. Table S9: pathway enrichment analysis of LDD known target-DR network. [file 5544518.f1.zip › 5544518.f1/Table S9.pdf]

Table S9 Pathway enrichment analysis

Term	Pathway	Count	%	Pvalue
hsa04066	HIF-1 signaling pathway	22	0.071806	3.69E-14
hsa04151	PI3K-Akt signaling pathway	33	0.107709	3.38E-10
hsa04010	MAPK signaling pathway	25	0.081598	4.56E-08
hsa04370	VEGF signaling pathway	13	0.042431	4.89E-08
hsa04910	Insulin signaling pathway	17	0.055487	6.41E-07
hsa04931	Insulin resistance	15	0.048959	8.49E-07
hsa04064	NF-kappa B signaling pathway	13	0.042431	2.72E-06
hsa04930	Type II diabetes mellitus	9	0.029375	3.27E-05
hsa04150	mTOR signaling pathway	9	0.029375	1.32E-04

Genes	Fold Enrichment	Bonferroni
PRKCA, EGFR, PIK3CG, IL6, ERBB2, RELA, HK2, PRKC[illegible]	8.567595109	8.32E-12
COL3A1, BCL2L1, PTEN, AKT1, CASP9, BCL2, NOS3, E[illegible]	3.576039698	7.61E-08
PRKCA, EGFR, TNF, RELA, TP53, RAF1, ELK1, TGFB1, [illegible]	3.694255886	1.02524E-05
PRKCA, PIK3CG, AKT1, MAPK1, PLA2G4A, CASP9, PTG[illegible]	7.967480399	1.10101E-05
PIK3CG, ACACA, HK2, RAF1, ELK1, AKT1, MAPK1, SL[illegible]	4.605505671	0.00014426
PIK3CG, PPARA, IL6, TNF, RELA, NFKBIA, PTEN, AKT1[illegible]	5.192481884	0.000190914
VCAM1, ICAM1, TNF, PTGS2, CD40LG, BCL2, RELA, N[illegible]	5.586394303	0.000612678
PIK3CG, MAPK1, TNF, SLC2A4, HK2, MAPK8, MTOR, I[illegible]	7.009850543	0.007323257
PRKCA, PIK3CG, AKT1, MAPK1, TNF, MTOR, IKBKB, P[illegible]	5.801255622	0.029315561
